# Extracellular vesicles of human glial cells exert neuroprotective effects via brain miRNA modulation in a rat model of traumatic brain injury

**DOI:** 10.1038/s41598-023-47627-2

**Published:** 2023-11-21

**Authors:** Diana I. Salikhova, Angelika V. Timofeeva, Victoria V. Golovicheva, Timur Kh. Fatkhudinov, Yulia A. Shevtsova, Anna G. Soboleva, Ivan S. Fedorov, Kirill V. Goryunov, Alexander S. Dyakonov, Victoria O. Mokrousova, Margarita O. Shedenkova, Andrey V. Elchaninov, Oleg V. Makhnach, Sergey I. Kutsev, Vladimir P. Chekhonin, Denis N. Silachev, Dmitry V. Goldshtein

**Affiliations:** 1 Institute of Molecular and Cellular Medicine, Medical Institute, RUDN University, Moscow, Russian Federation 117198; 2 Research Centre for Medical Genetics, Moscow, Russian Federation 115522; 3 V.I. Kulakov National Medical Research Center of Obstetrics, Gynecology and Perinatology, Moscow, Russian Federation 117997; 4 A.N. Belozersky Institute of Physico-Chemical Biology, Lomonosov Moscow State University, Moscow, Russian Federation 119992; 5 Faculty of Bioengineering and Bioinformatics, Lomonosov Moscow State University, Moscow, Russian Federation 119234; 6 Avtsyn Research Institute of Human Morphology of Federal State Budgetary Scientific Institution “Petrovsky National Research Centre of Surgery”, Moscow, Russian Federation 117418; 7 The Serbsky State Scientific Center for Social and Forensic Psychiatry, Moscow, Russian Federation 119034

**Keywords:** Stem cells, Regeneration and repair in the nervous system

## Abstract

Stem cell-based therapeutic approaches for neurological disorders are widely studied. Paracrine factors secreted by stem cells in vitro and delivered intranasally might allow bypassing the disadvantages associated with a surgical cell delivery procedure with likely immune rejection of a transplant. In this study, we investigated the therapeutic effect of the extracellular vesicles secreted by glial progenitor cells (GPC-EV) derived from human induced pluripotent stem cell in a traumatic brain injury model. Intranasal administration of GPC-EV to Wistar rats for 6 days improved sensorimotor functions assessed over a 14-day observation period. Beside, deep sequencing of microRNA transcriptome of GPC-EV was estimate, and was revealed 203 microRNA species that might be implicated in prevention of various brain pathologies. Modulation of microRNA pools might contribute to the observed decrease in the number of astrocytes that inhibit neurorecovery processes while enhancing neuroplasticity by decreasing phosphorylated Tau forms, preventing inflammation and apoptosis associated with secondary damage to brain tissue. The course of GPC-EV administration was promoted the increasing protein levels of NF-κB in studied areas of the rat brain, indicating NF-κB dependent mechanisms as a plausible route of neuroprotection within the damaged area. This investigation showed that GPC-EV may be representing a therapeutic approach in traumatic brain injury, though its translation into the clinic would require an additional research and development.

## Introduction

Traumatic brain injury (TBI) is a prominent medical and social problem associated with high risks of disability and lethality. The condition annually affects over 10 million people worldwide^1^. TBI is accompanied by profound damage to brain tissues. Specifically, the traumatizing event causes primary damage (acute phase) through mechanical disintegration of the brain parenchyma. The damaged cells produce high amounts of signaling molecules that can initiate inflammation, apoptosis and oxidative stress in the surrounding tissues^2^. The acute phase, which lasts about 24 h, is followed by chronic neuroinflammatory reaction triggered by pro-inflammatory (M1) microglia, reactive astrocytes crosstalk, disruption of the blood–brain barrier and recruitment of peripheral immune cells. This chronic reaction lasts long and can spread to the initially intact brain areas, leading to secondary post-TBI functional impairments including behavioral and cognitive^3,4^. Ultimately, the disruption of the brain tissue integrity can promote neurodegenerative processes, and the probability has been shown to correlate with TBI severity^5–7^. For example, lateral contusion damage to the cerebral cortex has been shown to extend considerably beyond the original deformity area, with neurodegenerative processes affecting the ipsilateral hippocampus, the ipsilateral and contralateral hemisphere, the dorsolateral nuclei of the thalamus, the dorsal part of the striatum and the corpus callosum^5^.

Stem cell transplantations are considered a promising tool for the treatment of neurodegenerative disorders, ischemic stroke and TBI. Importantly, their benefits are attributed not so much to the ability of the transplant to differentiate and replace damaged and dead recipient cells as to the stem cells capacity of producing biologically active substances (the paracrine effect)^8^. The advent of the induced pluripotent stem cells (iPSCs) technologies made it possible to obtain autologous neuronal and glial cells at different stages of differentiation in vitro. Glial cells are known to provide the trophic support to neurons by releasing a diverse spectrum of physiologically active substances including those incorporated in extracellular vesicles. Directed differentiation of iPSCs into glia cells can provide a source of biologically active molecules of developing human brain, including those expressed at embryonic stages, as possible contributors to the metabolic regulation, neuroprotection, functional neuromodulation and neurotrophic activity of the brain during post-TBI recovery. This study investigates the possibility of using the human pluripotent stem cell-derived glial cell extracellular vesicles (GPC-EV) for neuroprotection in a TBI model.

## Methods

### Extracellular vesicle isolation

Glial progenitor cells (GPCs) were previously obtained from three healthy donors (male and 2 females) by stepwise differentiation of human iPSCs in the glial direction^9–11^. Donor skin biopsy collection was approved by the Institutional Ethics Committee of the Research Centre for Medical Genetics (Protocol No. 2019-2/3 from October 13, 2020). CTS CytoTune-iPS 2.1 Sendai reprogramming kit (Invitrogen, USA) were used for fibroblast reprogramming. Obtained iPSCs were cultivated in the Essential 8 medium (Gibco, USA), and the pluripotent status of the iPSCs was confirmed, as previously described^9^. First stage of iPSCs differentiation was addition mix of 10 μM SB431542 (Tocris, USA), 2 μM Dorsomorphine (Sigma Algrich, USA), and 200 nM LDN193189 (Sigma Algrich, USA). Futher cultivation was carry out in DMEM/F12 medium (PanEco, Russia) supplemented with 1% N2 (PanEco), 1 mM glutamine (PanEco), 50 U/ml of penicillin–streptomycin (PanEco), 20 ng/ml of EGF (Peprotech, USA), and 20 ng/ml of CNTF (Peprotech) on a Matrigel (Corning, USA) coated dishes. For obtaining conditioned medium, the GPCs were cultured to confluency, washed with Hanks’ Balanced Salt Solution (PanEco), and incubated for 24 h in maintained medium. The collected conditioned medium was spun by centrifugation at 3000 rpm for 5 min, and repeatedly at 10,000 g for 30 min. Extracellular vesicles (EV) were spun at 108,000 g for 1.5 h using an Avanti JXN-30 ultracentrifuge equipped with JA-30.50 Ti rotor (Beckman Coulter Inc., USA). The pellet was washed with phosphate-buffered saline (PBS; PanEco) and the ultracentrifugation was repeated. The obtained EV isolates were stored in PBS at − 80 °C, and used within 2 months.

### Nanoparticle tracking assay

Particle size distribution analysis and concentration measurements for GPC-EV isolates were carried out by nanoparticle tracking assay using a Nanosight LM10 HS (NanoSight Ltd., UK) according to the ASTM E2834-12 standard^12^. Briefly, the samples were diluted with PBS (Gibco, USA) to about 1.5 × 10^8^ particles/ml. Videos of the Brownian particle motion were recorded at room temperature with a passive temperature readout and the following camera settings: camera shutter 850, camera gain 450, lower threshold 715 and upper threshold 10,725. The records were processed using Nanoparticle Tracking Analysis version 2.3 build 0033 analytical software (NanoSight Ltd.) at a 9% sensitivity threshold. A total of 12 separate video clips were processed, 60 s each, amounting to over 5000 tracks. Data from multiple videos were combined to obtain a histogram of particle size, and mean total concentration corrected for the dilution factor. A sample obtained by identical ultracentrifugation of non-conditioned medium was used as a reference (blank).

### Transmission electron microscopy

GPC-EV samples were dispensed in 10 µl aliquots on nitrocellulose carbon coated PELCO® Cu grids (Ted Pella Inc., USA) and incubated for 1 min. Then 10 µl of 2% uranyl acetate were applied, the grids were incubated for 15 s and excess liquid was removed with nitrocellulose paper. The samples were examined at 80 kV using a JEM-1011 transmission electron microscope (JEOL, Akishima, Japan) equipped with an Orius™ SC1000 W camera (Gatan + Inc., Pleasanton, CA, USA).

### Deep sequencing of miRNA from extracellular vesicles

cDNA libraries were synthesized using 7 µl of the 14 µl total RNA column eluate (miRNeasy Micro kit, Qiagen, Hilden, Germany), extracted from GPC-EV using NEBNext Multiplex Small RNA Library Prep Set for Illumina (Set11 and Set2, New England Biolab, Frankfurt am Main, Germany), amplified for 23 PCR cycles, purified by QIAQuick PCR Purification Kit (Qiagen, Hilden, Germany), and 6% polyacrylamide gel electrophoresis for isolation of 136–150 bp bands corresponding to the adapter-ligated miRNAs. The obtained cDNA libraries were analyzed using a Qubit 2.0 fluorimeter (Qubit DS HS assay, Invitrogen, USA), Agilent 2100 Bioanalyzer (Agilent HS DNA Analysis Kit, Agilent Technologies), and were sequenced on the NextSeq 500 platform (Illumina, San Diego, CA, USA) with NextSeq 500/550 High Output Kit v2 (75 cycles, Illumina, San Diego, CA, USA). Obtained FASTQ files containing the sequence data, were processed as following: the adapters were removed with Cutadapt; all trimmed reads shorter than 16 bp and longer than 30 bp were filtered; the remaining reads were mapped to the GRCh38.p15 human genomes miRBase v21 with the bowtie aligner^13^; aligned reads were counted with the featureCount tool from the Subread package^14^ and with the fracOverlap 0.9 option, so the whole read was forced to have a 90% intersection with miRNA features; comparison analysis of the miRNA count data was performed with the DESeq2 package^15^.

### Animals

The experiments involved mature male Wistar rats, 250–300 g body weight, n = 30. All manipulations with animals were approved by the Ethical Committee at the Avtsyn Research Institute of Human Morphology of Federal state budgetary scientific institution “Petrovsky National Research Centre of Surgery” (Protocol No. 38(14)), carried out in accordance with Directive 2010/63/EU of the European Parliament and of the Council and ARRIVE guidelines. Upon the arrival from breeding facilities, the animals housed 5 animals per cage, at a 12 h/12 h light–dark cycle and 24 °C air temperature, were quarantined for 14 days prior to TBI modeling. After the operation, the animals were housed individually, 1 rat per cage, for 2 weeks to avoid trauma and facilitate the recovery.

### Traumatic brain injury model

All surgical interventions were performed under inhalation anesthesia with isoflurane (Aerrane, Baxter HealthCare Corporation, USA) air mixture at concentrations of 5% at commencement and 1.5–2.5% for maintenance; additionally, the surgical area on the head was subject to preoperative infiltration anesthesia with lidocaine. The injury was modeled using the dosed concussion to the open brain method^16^. The animal, scalp-shaven, was fixed in a stereotaxic frame, and a longitudinal median incision was made to the scalp. A cutter 5 mm in diameter was used to drill a hole in the skull above the left hemisphere 2.5 mm lateral and 1.5 mm caudal to the bregma, corresponding to sensorimotor cortex. The mechanical impact device was inserted, comprising a cylindrical striker at a depth of 3 mm below the skull bones above the dura mater. The injury was inflicted by dropping a 50 g load from a height of 10 cm. The wound was sutured with a simple nodal seam and treated with antiseptic, and the animal was transferred to a warmed-up cage for awaking. The body temperature of the animals was maintained at a physiological level using an infrared lamp and a thermostat.

### Study design

**Figure 1:**
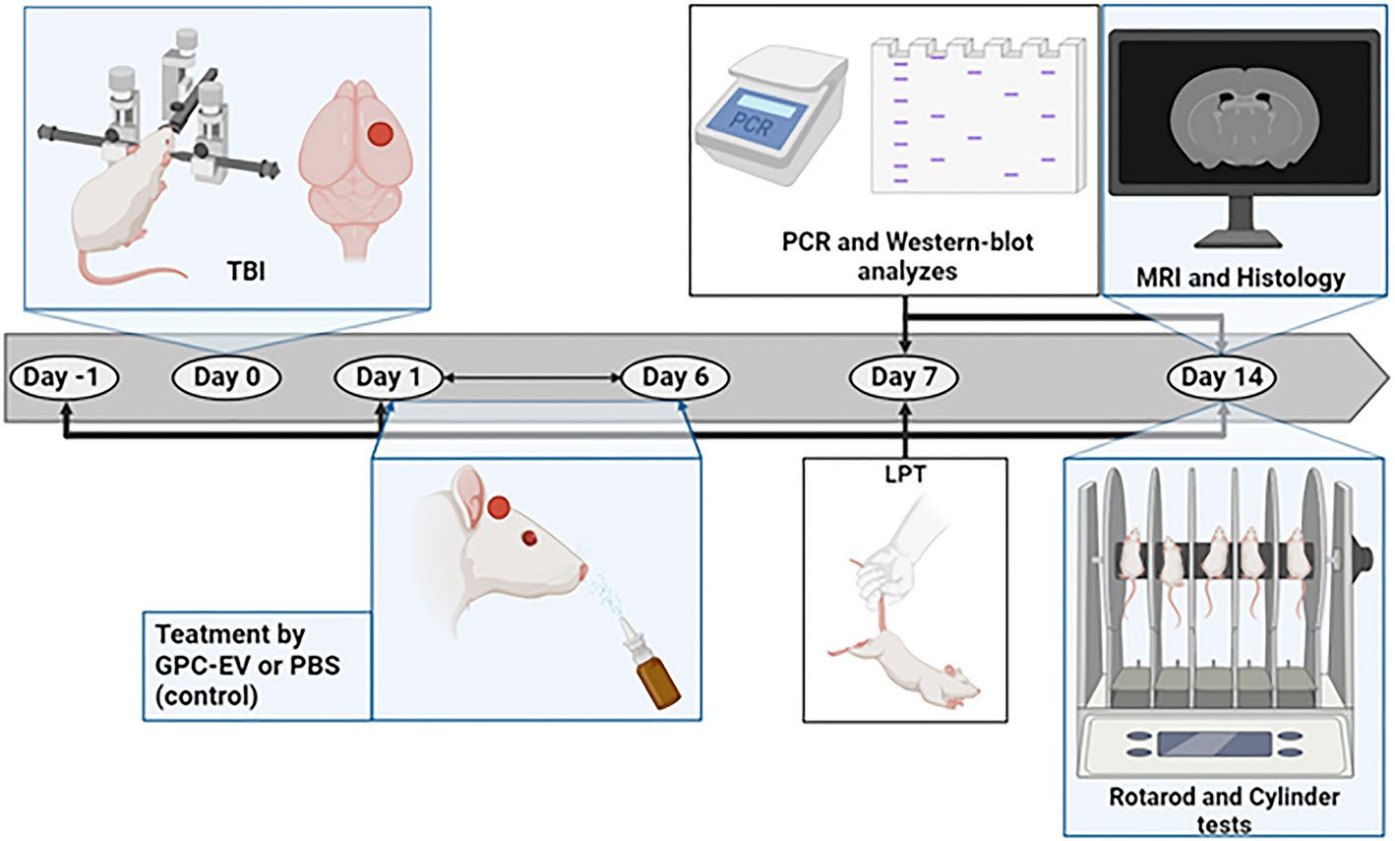
Study design. A scheme of the experiment on neuroprotective properties of GPC-EV in TBI model, created using https://biorender.com/. *TBI* traumatic brain injury, *LPT* limb-placing test, *MRI* magnetic resonance imaging, *PCR* polymerase chain reaction.

The animals were randomly divided into 3 groups: a sham-operated group (animals that underwent trepanation without TBI, n = 6); TBI (animals that underwent TBI with receiving 30 µl PBS intranasally, n = 15), and TBI + GPC-EV (animals with TBI, and intranasal administration of GPC-EV in PBS at dos 2 × 10^11^ particles/ml with total protein concentration 12.7 ± 0.6 µg/ml, n = 15). The administration was carried out for 6 days and the observation lasted 14 days. Neurological status of the animals was assessed before surgery and on postoperative days 1, 7 and 14 (n = 10). Analysis of the volume of brain damage was performed by magnetic resonance imaging (MRI) on postoperative day 14 (n = 10) and the animals were withdrawn from the experiment for histological examination (n = 5). Western blotting and PCR analysis of brain tissues were performed on postoperative day 7 and 14 (n = 4–6). miRNA expression level was assessment on day 7 (n = 3). All methods were performed in accordance with the relevant guidelines and regulations (Fig. 1).

### Behavioral tests

All behavioral tests were performed on equipment manufactured by Open Science LLC (Russia). The test cylinder was used to analyze the asymmetry in the use of the forelimbs during spontaneous exploration of cylindrical walls from inside^17^. The test was carried out on post-TBI day 14. The rat was placed in a transparent cylinder 30 cm high and 20 cm in diameter, and its motions were recorded for 5–8 min with a video camera located under the cylinder. The independent use of the contra- and ipsilateral forelimbs during cylinder wall exploration in a rear posture, and their simultaneous (combined) use were counted. The frequency of using the forelimbs was calculated by the formula: (contr + 1/2 × sim)/(ipsi + sim + contr) × 100, where contr stands for contralateral forelimb (affected), sim—simultaneous use of both forelimbs, ipsi—ipsilateral forelimb.

The limb-placing test was carried out before the injury (the rats were handled 3 days in advance) and repeatedly on post-TBI days 1, 7 and 14 as a measure of sensorimotor impairment. The test was based on the published protocol^18^, and included 7 tasks on sensorimotor performance of fore- and hindlimbs in response to tactile and proprioceptive stimulation. The results were scored in points: normal performance (prompt and complete), 2 points; delayed (> 2 s) and/or incompletely performance, 1 point; failed test, 0 points.

In the rotarod test, the animals were placed on a rotating rod, and the time the animals remained on the rod was recorded. The sequence ramping was gradually raised from 4 to 40 rpm in the course of 5 min. The session was finished when the animal fell, and the time was recorded. An arbitrary time limit of 500 s was fixed during training and testing procedures. The training was carried out once a day on post-TBI days 11, 12 and 13, and the test was carried out on post-TBI day 14 as described^19^.

### MRI morphometry of brain damage

MRI was performed in a BioSpec 70/30 MRI scanner (Bruker, Germany) with a 7 T magnetic field induction and a 105 mT/m gradient system. To produce high-resolution T2-weighted images of the brain, the animals were anesthetized with isoflurane air mixture at concentrations of 5% at commencement and 1.5–2.5% for maintenance, and transferred to a positioning device equipped with stereotaxis and thermoregulation systems.

The brain volume damage was determined with ImageJ software package (Wayne Rasband, National Institute of Mental Health, Bethesda, Maryland, USA) using T2-weighted images on post-TBI day 14. The damage area was outlined manually. The infarction volume was calculated as V = (S1 + ⋯ + Sn) × (h + d), where S is the affected area, mm^2^, summed up for slices 1 to n; h—slice thickness, mm; d—interslice space, mm.

### Histology and immunohistochemistry

The animals were withdrawn from the experiment on post-TBI day 14. After euthanasia with a lethal dose of isoflurane, the animals were decapitated. The brain was removed from the skull, and gradually cooled to − 80 °C, or placed in 10% neutral buffered formalin to prepare paraffin sections. The formalin-fixed tissues were dehydrated and embedded in paraffin. The 5–7 µm thick sections were placed on gelatin-coated slides, and dried at 37 °C for 24 h. The sections were deparaffinized in xylene, and rehydrated in 100°–70° ethanol series, stained with hematoxylin and eosin (H&E), dehydrated and mounted. The frozen tissues were embedded in Tissue-Tek OCT Compound (Sakura Finetek), and sectioned at 4–5 µm in a Leica CM1950 cryostat (Leica Microsystems, Germany). The slides were blocked in PBS with 0.3% Triton X-100 and 2% bovine serum albumin for 1 h, and treated with primary antibodies to CD68 (1:100, ab125212), iba1 (1:200, ab178847), and GFAP (1:600, ab7260) (Abcam, UK) at 4 °C overnight. The slides were washed; then secondary antibodies Goat anti-Rabbit IgG (H+L) Alexa Fluor™ 488 (1:600, A-11008) (TermoFisher, USA) were added, and the slides were incubated for 1 h in the dark. The nuclei were counterstained with DAPI, 1 µg/ml in PBS. At least three series were photographed an Axio Observer.D1 luminescent inverted microscope equipped with AxioCam HRc camera (Carl Zeiss, Germany). Positively stained cells were counted using the ImageJ software.

### mRNA quantitation by real-time PCR coupled with reverse transcription

Tissue fragments of the brain (cortex at the site of injury, hippocampus and striatum from the same hemisphere) were placed in RNAlater reagent (Thermo Fisher, USA) for stabilization. Total RNA was isolated using RNeasy Mini Kit (Qiagen, Netherlands) according to the manual, and cDNA was synthesized using RevertAid First Strand cDNA Synthesis Kit (TermoFisher) by corresponding standard protocol. The PCRs set with qPCRmix-HS SYBR master mixes (Evrogen, Russia) were carried out in a BioRad iQ cycler (BioRad, USA) in the following mode: primary denaturation 95 °C for 5 min; denaturation 95 °C 20 s, annealing 56–63 °C 20 s, elongation 72 °C 20 s (40 cycles). Relative mRNA levels for the analyzed genes of interest were calculated as threshold cycle values normalized by corresponding data for reference housekeeping genes *Gapdh* (glyceraldehyde-3-phosphate dehydrogenase) and *Actb* (β-actin) using ΔC(T) approach. The amplification primers designed with NCBI Primer-Blast tool are given in Table S1.

### miRNA quantitation by real-time PCR coupled with reverse transcription

cDNA was synthesized with miScript II RT Kit (Qiagen, Hilden, Germany) using 250 ng of total RNA isolated from brain tissue samples as described in the previous section. Synthesized cDNA was quantified using miScript SYBR Green PCR Kit (Qiagen, Hillen, Germany), and a custom miScript miRNA PCR Array (Cat. No./ID: 331231, Qiagen, Hilden, Germany) in the 96-well format for analysis of 45 miRNAs, SNORD61, SNORD72 (small nucleolar RNA, C/D box 61/72) in 2 samples per plate, and PPC (PCR positive control) offered by the manufacturer. The real-time PCR was carried out on a CFX96 system (BioRad, USA) according to the program: 95 °C—15 min, 45 cycles: 94 °C—15 s, 55 °C—30 s, 70 °C—30 s, followed by analysis of the melting curves of the obtained PCR-products plotted in the range of 55 °C—95 °C. The relative amount of miRNA was estimated by the equation ΔCt = Ct(miRNA) − Ct(SNORD), where Ct is the value of the amplification cycle at the point of intersection of the kinetic curve of the accumulation of the amplification product with the line of the threshold fluorescence level, which is determined automatically by the software of the CFX96 system.

### Western blot analysis

Tissue fragments of the brain (cortex at the site of injury, hippocampus and striatum) were lysed using Protein Solubilization Buffer Kit (BioRad) supplemented with Halt Protease and Phosphatase Inhibitor Cocktail (Thermo Fisher) as recommended, 4 µl of complete lysis buffer per 1 mg tissue. After thorough mechanical grinding to homogeneous suspension the samples were centrifuged for 30 min at 20,000×*g* and 4 °C. The supernatants were transferred to fresh tubes and kept at 4 °C, whereas the pellets underwent another round of the procedure. The supernatants from the two rounds were combined, and aliquots were mixed with equal volume of 2× Laemmli Sample Buffer (BioRad). The GPC-EV preparation aliquots (see corresponding section above) were mixed with 4× Laemmli Sample Buffer (BioRad). Prior to gel loading, all samples were heated at 99 °C for 10 min. The gels were prepared using TGX Stain-Free FastCast Acrylamide Starter Kit, 10% (BioRad) in accordance with the manual. The protein transfer to nitrocellulose membranes was performed electrically using a Trans-Blot Turbo Transfer System (BioRad). The membranes were blocked in PBS with 3% BSA overnight. Next day, the membranes were incubated with primary antibodies diluted in 0.1% Tween-20 and 1% BSA. The targets included p-Tau Ser396 (1:1000, ab32057, Abcam, UK), p-Tau Thr205 (1:1000, ab254410, Abcam, UK), Tau (1:1000, 46687, Cell Signaling, UK), NF-κB (1:500, 8242, Cell Signaling, UK), β-actin (1:1000, 8457, Cell Signaling, UK) for neurodegeneration markers analysis; β-actin (1:2000, A1978, Sigma-Aldrich, USA), cleaved CASP-9 (1:500, CSB-PA000010, Cusabio, China) for caspase-9 analysis; and CD63 (1:500, AF5117, Affinity Biosciences, China), CD81 (1:500, DF2306, Affinity Biosciences, China), CD9 (1:500, DF6565, Affinity Biosciences, China) for GPC-EV characterization. The membranes were washed from primary antibodies and exposed to secondary antibodies anti-Rabbit IgG conjugated Alexa Fluor 488 (1:1000, A-11008) or anti-Mouse IgG conjugated Alexa Fluor 555 (1:1000, A-21422) (Thermo Fisher, USA) for 60 min in the dark. The immunoblots were documented in a BioRad Chemidoc Imaging System (BioRad). The phospho-Tau (Ser356) control lysate (64TS356TDA), phospho-Tau (Ser202/Th205) control lysate (64TS2TDA), total Tau control lysate (64NTAUTDA), total NFkB control lysate (64NFTTDA) (all CISBIO BIOASSAYS, France) were used for positive controls; NFkB Knockout Cell Lysate from HeLa (# DAG-KO066, Creative Diagnostics, USA), lysate from rat liver tissue for Tau and their phosphorylated forms were used for negative controls, and mouse L929 Apoptosis Cell Lysates (#9503, cell signaling, UK) were used for both controls (Supplementary Fig. S4). Semi-quantitative calculations of specific protein content as normalized to β-actin were performed in ImageLab Software (BioRad). The levels of active caspase-9 was calculated by the formula: active caspase-9/(active caspase-9 + procaspase-9) × 100%.

### Statistics

Statistical analysis was carried out in SigmaPlot 12.5 (Systat Software, USA). Normality of the distributions was estimated using Shapiro–Wilk test. The MRI, miRNA PCR assay, Western blot, and immunohistochemistry analysis were analyzed using *t*-test (for normal distributions) or nonparametric Mann–Whitney test (for distributions differing from normal). The mRNA PCR quantiation, cylinder and rotarod tests data were performed with the Kruskal–Wallis test and post hoc Dunn’s test. The limb-placing test data were analyzed by two-way ANOVA with post hoc Holm–Sidak correction for multiple comparison. The data are presented as means and standard deviations or medians with range. The differences were considered statistically significant at p ≤ 0.05.

### Ethics approval and consent to participate

The Research Centre for Medical Genetics approved the collection of human glial progenitor cells derived from human induced pluripotent cells used in the study (Protocol No. 2019-2/3 from 13 October 2020). Patients provided informed written consent for the use of tissue for research purposes.

All manipulations with animals were approved by the Ethical Committee at the Avtsyn Research Institute of Human Morphology of Federal state budgetary scientific institution “Petrovsky National Research Centre of Surgery” (Protocol No. 38(14)), carried out in accordance with Directive 2010/63/EU of the European Parliament and of the Council and ARRIVE guidelines.

## Results

### GPC-EV characterization

**Figure 2:**
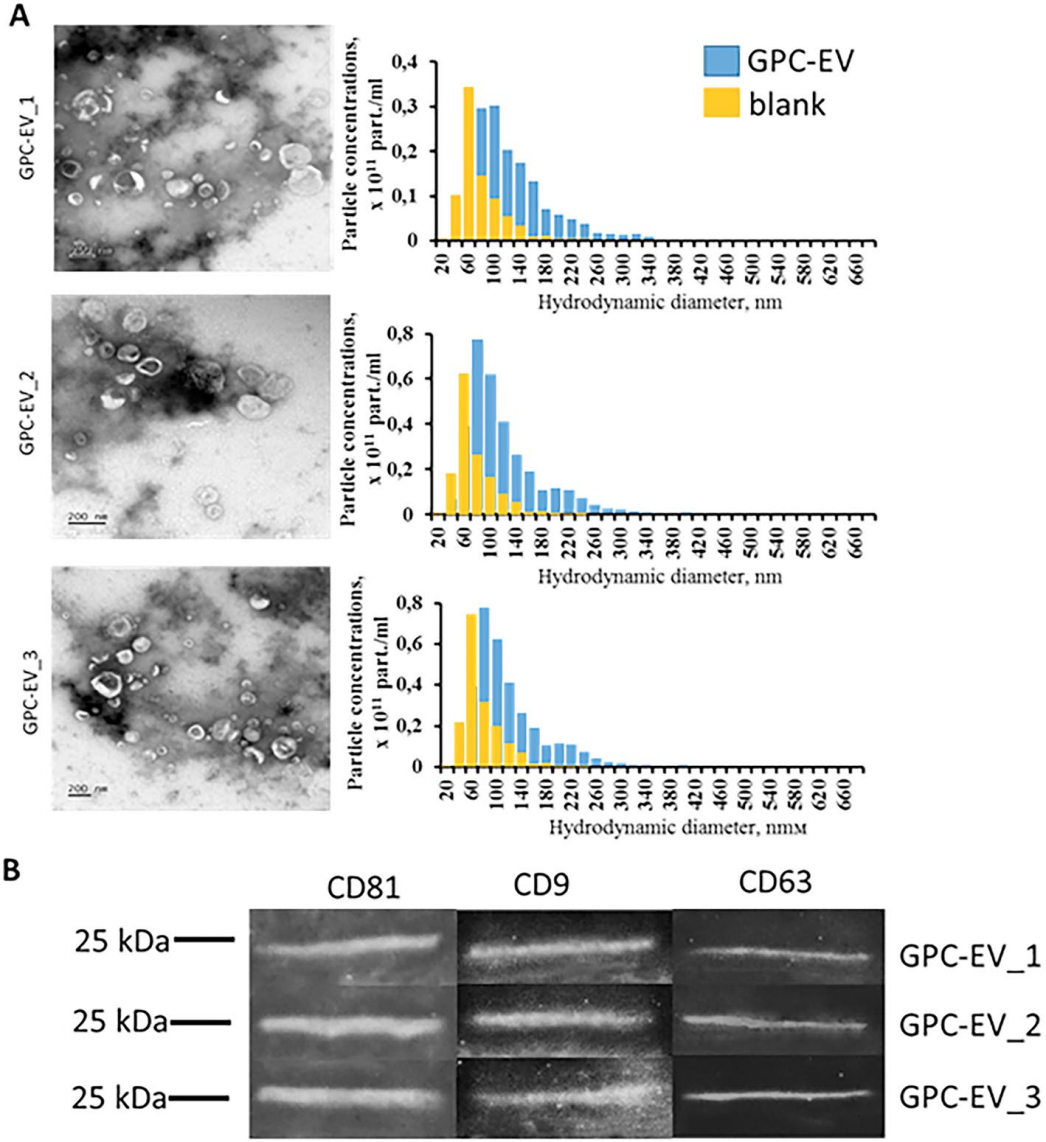
Characterization of extracellular vesicles released by human GPCs obtained from three independent donors. (**A**) Transmission electron microscopy images and size distribution charts for three isolates (co-plotted with the non-conditioned medium control data, blank). (**B**) Representative immunoblots showing the presence of tetraspanins CD81, CD9, and CD63 in three samples.

The nanoparticle tracking assay for concentration and size distribution measurements, and transmission electron microscopy for morphological assessment of GPC-EV were used. Most of the particles in three independent samples fell in a range of 60–300 nm with a peak at 80–100 nm, which indicated predominance of the exosomal fraction in the isolates (Fig. 2A). Concentrations of particles produced by GPCs from three independent donors varied slightly—from 1.58 × 10^11^ to 3.28 × 10^11^ particles per ml isolate. By contrast, the blank sample (obtained by ultracentrifugation of non-conditioned medium and used as a reference) contained 2.08 ± 0.69 × 10^8^ particles per ml, with sizes peaking at 60 nm. As revealed by transmission electron microscopy, most particles in the isolates had cup-shaped morphology characteristic of EV, opposed to a minor portion of smooth-surfaced, smaller-sized objects of spherical shape (Fig. 2A). All three samples contained tetraspanins CD81, CD9, and CD63, which are specific for extracellular vesicles. (Fig. 2B).

### GPC-EV miRNA profiling

**Figure 3:**
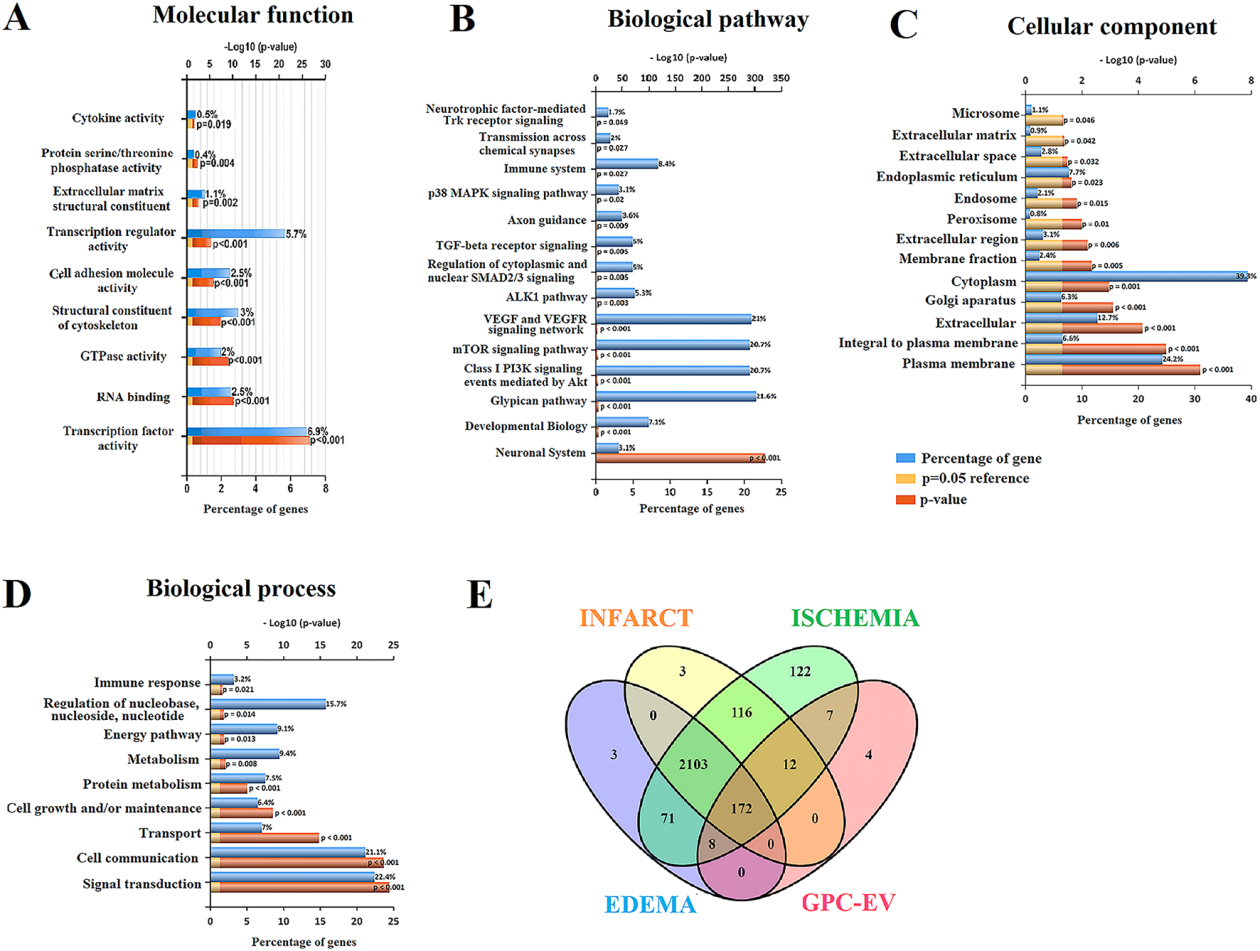
Human GPC-EV miRNA profiling. Identified miRNAs were classified by molecular functions (**A**), biological pathways (**B**), cellular components (**C**), and biological processes (**D**) using Funrich software. (**E**) Venn diagram of GPC-EV miRNA functional significance according to MirWalk 2.0.

The miRNA profiles in GPC-EV isolates obtained from three independent donors were studied by deep sequencing with the reads counted for each identified molecule from a total of 203 different miRNAs. The coefficients of read number variation for each miRNA in GPC-EV isolates (calculated as a standard deviation-to-median ratio, n = 3), not exceeding 0.5 for 77.4% of the identified miRNA species, are given in Table S2. The functional significance of GPC-EV miRNA target genes as analyzed by Funrich software (http://funrich.org/), and results are given in Supplementary Tables S6–S9. The identified miRNAs were implicated in specific molecular functionalities including transcription factor activity, transcription regulator activity, structural constituent of cytoskeleton, protein serine/threonine kinase activity, receptor activity, GTPase activity, extracellular matrix structural constituent, and etc. (Fig. 3A, all molecular functions in Table S7). The GPC-EV miRNAs were implicated in several signaling pathways through their targets (see all biological pathways data in Table S9). Specifically, over 20% of GPC-EV miRNA-regulated genes belong to VEGF and VEGFR signaling network, Glypican pathway, mTOR signaling pathway and/or Class I PI3K signaling events mediated by Akt, involved in key cellular functionalities including energy metabolism, proliferation and survival. Several genes involved in axon guidance and transmission across chemical synapses also identified among the targets may represent a link to neuronal plasticity (Fig. 3B). In addition, the identified GPC-EV miRNAs can influence on various cellular components (see all cellular components data in Table S6) including the microsome, the cytoplasm, the Golgi apparatus, the extracellular matrix/space, and others, participating in the regulation of such main biological processes (see all biological processes data in Table S8) as immune response, metabolism, transport, cell communication, signal transduction, cell growth and maintenance, energy pathway, etc., thereby maintaining the integrity and functional activity of nervous tissue (Fig. 3C,D).

The MirWalk 2.0 electronic database (http://mirwalk.umm.uni-heidelberg.de/diseases/) was addressed to assess the identified GPC-EV miRNAs for potential therapeutic relevance in various pathological conditions of the brain including brain edema (180/203 GPC-EV miRNAs), brain infarct (184/203 GPC-EV miRNAs), brain ischemia (199/203 GPC-EV miRNAs), as shown in Fig. 3E plotted using Venny 2.1 online tool (https://bioinfogp.cnb.csic.es/tools/venny/).

### GPC-EV therapeutic effect evaluation by MRI and behavioral tests

**Figure 4:**
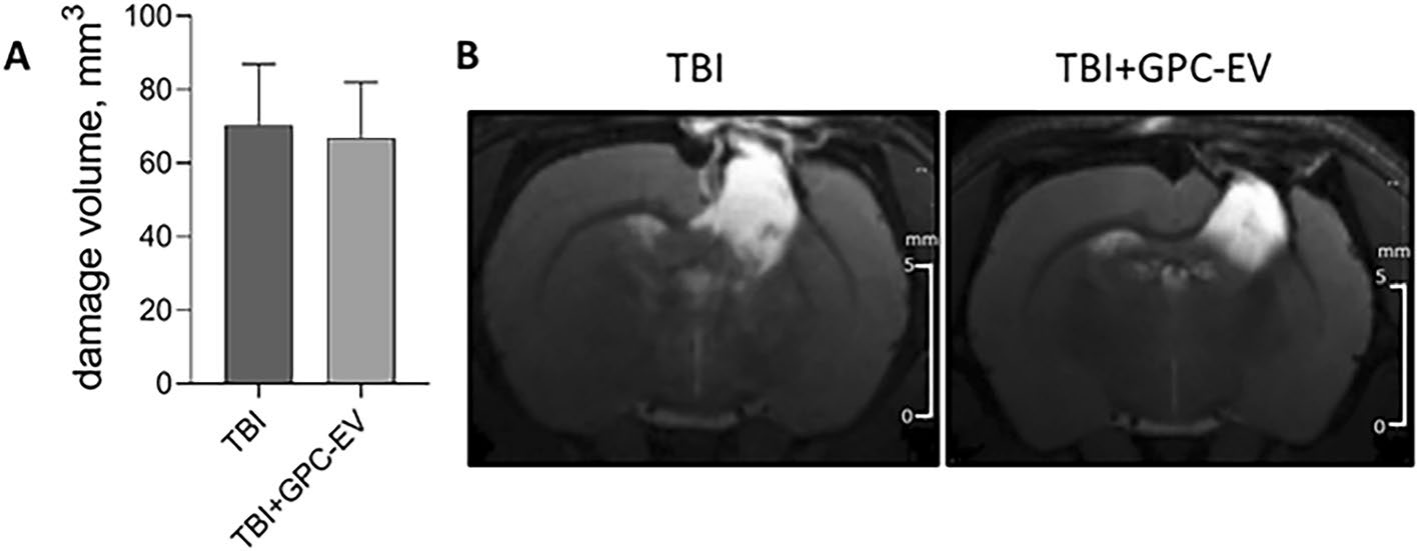
The assessment of damage volume by MRI on day 14. (**A**) Comparison of the quantitation data presented as means ± SD; (**B**) representative T2-weighted images, the hyperintense (light) zones correspond to damage area, scale bars, 5 mm. TBI group—brain traumatized rats that received 30 µl PBS intranasally (n = 10), TBI + GPC-EV group—rats with brain injury, and intranasal administration of GPC-EV (n = 10).

The modeled open TBI caused substantial damage to the corresponding sensorimotor cortical area (Fig. 4A). By the end of observation (day 14) the infarction volume constituted 70.4 ± 16.5 mm^3^ in TBI group. The intranasal GPC-EV administration had no significant effect on the infarction volume (66.9 ± 15.1 mm^3^, Fig. 4).

**Figure 5:**
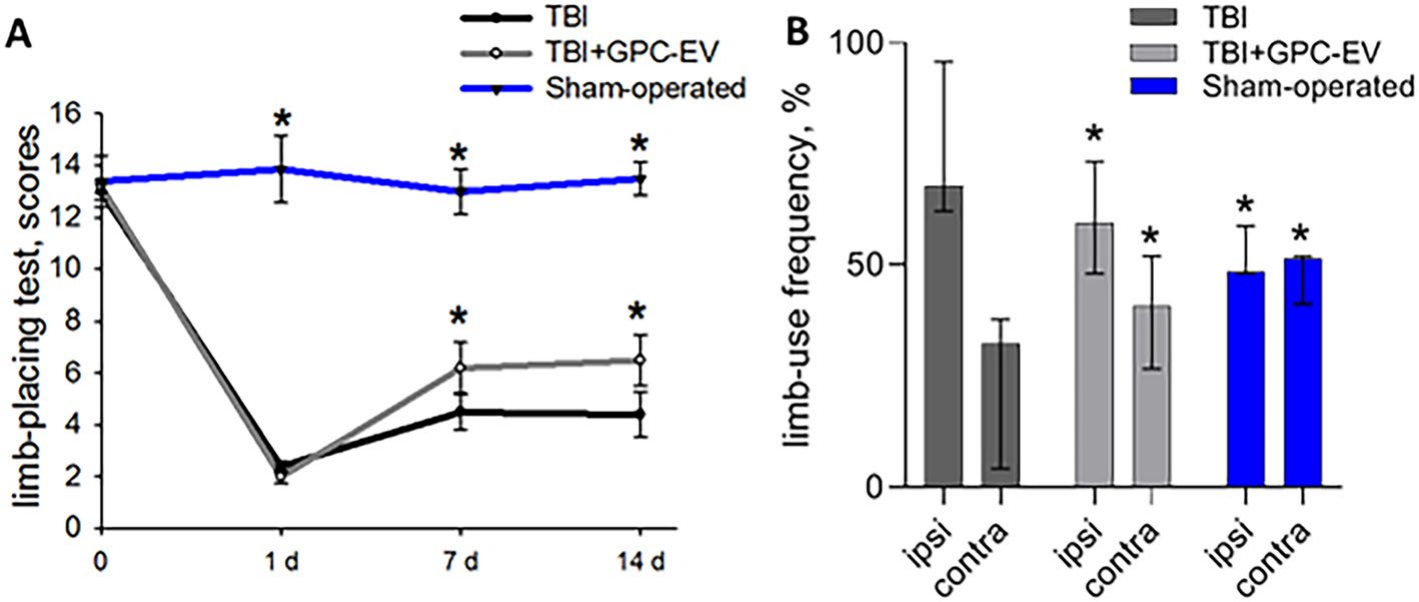
Neurological outcomes of animals during post-TBI recovery. (**A**) Limb-placing test data analyzed by two-way ANOVA with Holm–Sidak correction for multiple comparison; the data are presented as mean ± SD (*p ≤ 0.05 compared with the TBI group). (**B**) Cylinder test exposing the use of ipsi- and contralateral forelimbs. The data analyzed by Kruskal–Wallis test with post hoc Dunn’s test are presented as median with range (*p ≤ 0.05 compared with the TBI group). TBI group—brain traumatized rats that received 30 µl PBS intranasally (n = 10), TBI + GPC-EV group—rats with brain injury, and intranasal administration of GPC-EV (n = 10). Sham-operated group—rats that underwent trepanation without TBI (n = 6).

The TBI induction caused significant fore- and hindlimb sensorimotor deficits, with limb-placing score of 13.2 ± 0.43 as measured before the brain trauma decreased to 2.2 ± 0.7 score on post-TBI day 1 (Fig. 5A). Sham-operated rats during the experiment showed the same score as animals before TBI. In saline threated group, the score remained low until the end of observation period, constituting 4.5 ± 2.1 and 4.4 ± 1.5 on post-TBI days 7 and 14, respectively (Fig. 5A). Intranasal administration of GPC-EV afforded significant improvement in neurological status, as indicated in limb-placing test scores of 6.2 ± 0.9 and 6.5 ± 2.2 on post-TBI days 7 and 14, respectively.

The cylinder test was applied to assess the degree of asymmetry in the use of forelimbs (Fig. 5B). Normally, a rat explores the walls of a closed compartment with both forelimbs in the same proportion. In TBI, the unilateral damage to sensorimotor cortex leads to asymmetry in the use of forelimbs and a corresponding adaptive strategy with predominant use of ipsilateral limb controlled by contralateral hemisphere (as related to the site of injury). Accordingly, sham-operated rats used contra- and ipsilateral limbs equally, whereas on day 14 animals with TBI used contra- and ipsilateral forelimbs with 32 [4; 38]% and 68 [62; 96]% frequencies, respectively. The symmetry was partially restored by the intranasal GPC-EV administration, with forelimb usage frequencies of 40 [27; 52]% and 60 [48; 73]% for contra- and ipsilateral limbs, respectively (Fig. 5B). In the rotarod test applied on post-TBI day 14, the median value was 140 [73; 323] s for the GPC-EV treatment group and 215 [27; 317] s—for TBI group, without statistically significant difference between the groups. Sham-operated rats lasted for the entirety of the allotted time, 298 [285; 300] s (Fig. S1).

### Comparative immunohistochemistry

**Figure 6:**
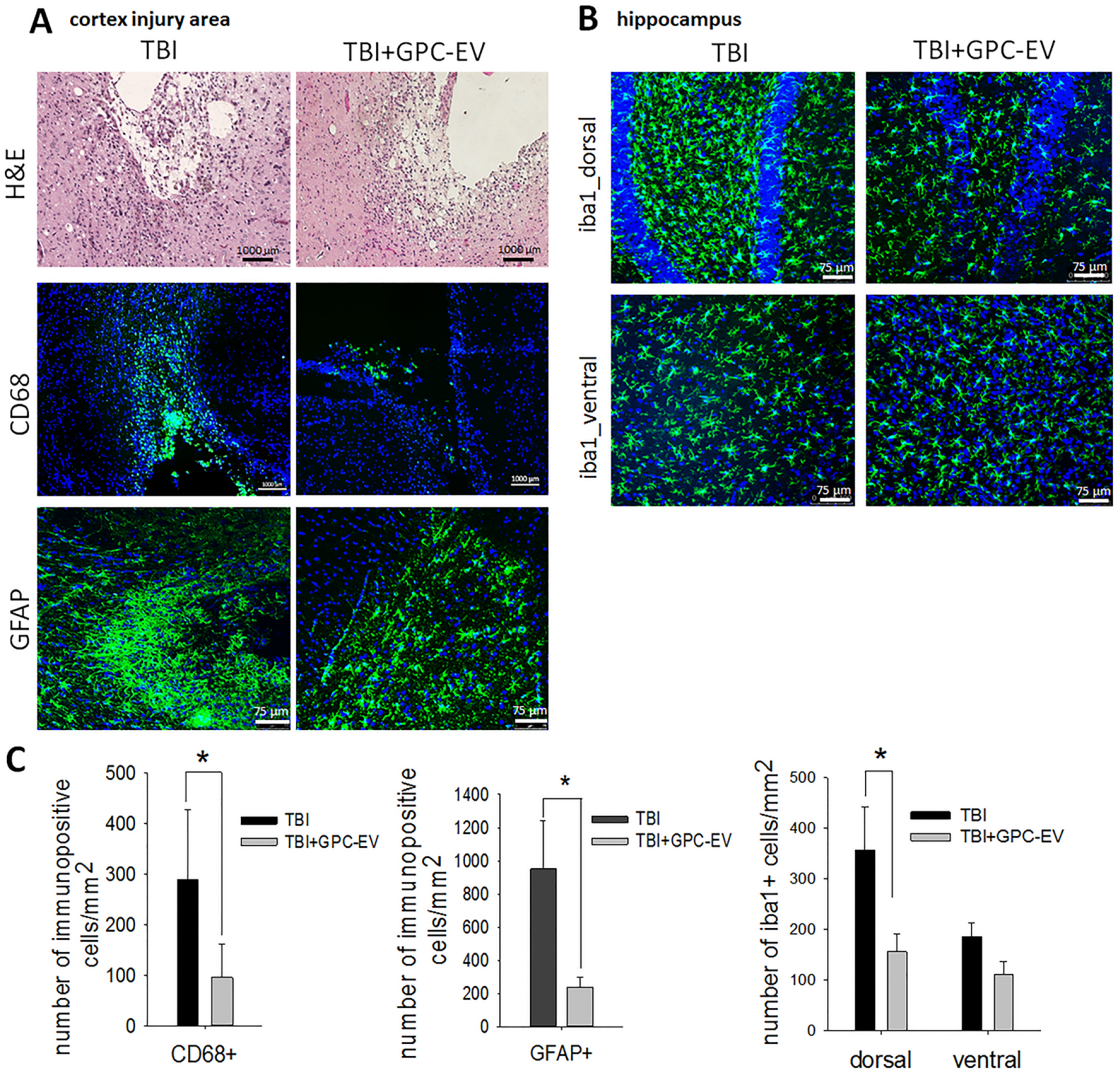
Immunohistochemical study of brain tissues on post-TBI day 14. (**A**) Representative histology images with hematoxylin and eosin stain (H&E), and visualization of monocytic macrophages (CD68^+^, green fluorescence), and astrocytes (GFAP^+^, green fluorescence) at the site of traumatic injury within the cortex. (**B**) Visualization of microglia (iba1^+^, green fluorescence) in hippocampus. (**C**) Counts of number monocytic macrophages, astrocytes, and microglia cells. The data analyzed by *t*-test are presented as means ± standard deviations (*p ≤ 0.05 compared with the TBI group). Scale bars, 1000 µm or 75 µm. Cell nuclei counterstained with DAPI (blue fluorescence). TBI group—brain traumatized rats that received 30 µL PBS intranasally (n = 5), TBI + GPC-EV group—rats with brain injury, and intranasal administration of GPC-EV (n = 5).

In both groups, pericellular edema was observed at the site of injury. The pia mater in this zone was damaged. Infiltration with lymphocytes and macrophages was detected in the remaining areas (Fig. 6A, H&E). Immunohistochemical study conducted on post-TBI day 14 revealed immunopositive cells identified as monocytic CD68^+^ macrophages at the site of injury (Fig. 6A). Amount of CD68^+^ cells was three times lower in the TBI + GPC-EV group compared with the TBI group (Fig. 6C).

Both groups of the study presented with glial scars at the damage area. In TBI group, a glial scar was detected in the cerebral cortex, represented by astroglial large cells with rounded light nuclei, and microglia—cells with small hyperchromic nuclei. Microglial cells predominate among glial cells. Neurons in the scar area are not detected. In experimental group (TBI + GPC-EV), the area of damage was represented by a small glial scar, in which microglial cells, astroglia, and fibrous structures are determined (Fig. 6A, H&E). By immunohistochemical analysis, it was found that in the GPC-EV treatment group GFAP ^+^ cell (astroglia) were 4 times lower compared with the TBI group on day 14 (Fig. 6C). But statistically significant differences between amounts of microglia cells (defined as iba1 ^+^ cells) were not detected in the area of damage (Fig. S3A). The neuroinflammatory brain damage could spread to other regions of the ipsilateral brain hemisphere. Significant differences in iba1 ^+^ microglial cells counts (lower in the GPC-EV receiving animals) were confined in dorsal hippocampus (Fig. 6B,C), and no significant differences were observed for astroglia (GFAP^+^ cells, Fig. S3C). In striatum, astroglial and microglial cell counts between two groups were similar (Fig. S3B).

### Comparative transcriptomic study

Representation of transcripts encoding matrix metalloproteinases and their inhibitors, as well as transcriptomic markers of apoptosis/inflammation and miRNAs in different brain regions including the cortex, the striatum and the hippocampus was studied to investigate molecular-genetic mechanisms of the observed therapeutic effects of GPC-EV.

**Figure 7:**
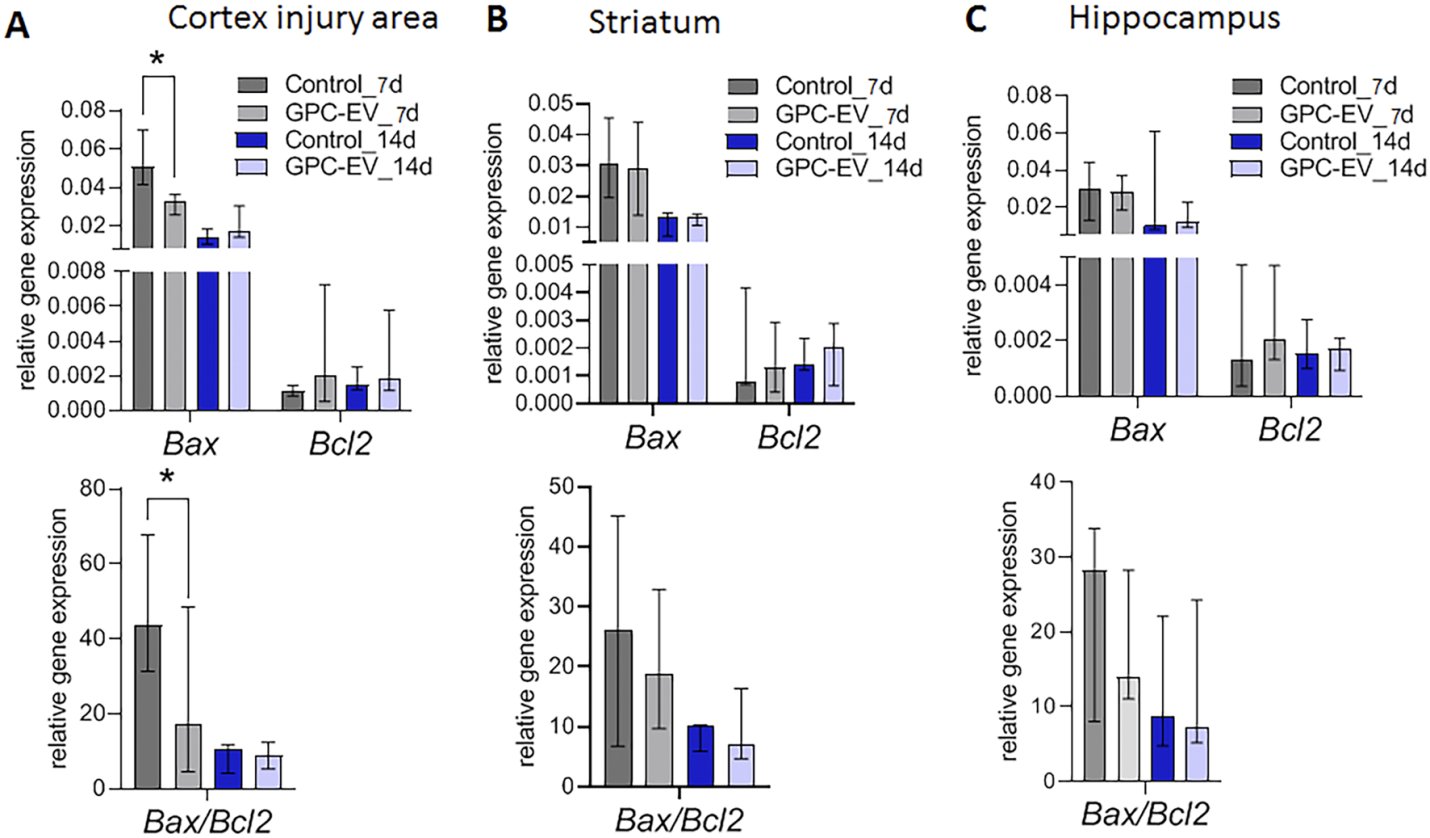
mRNA expression levels of markers of apoptosis in different brain regions: (**A**) cortex (traumatic lesion); (**B**) striatum; (**C**) hippocampus. Reverse transcription PCR data for mRNA analyzed by Kruskal–Wallis test with post hoc Dunn’s test are presented as median with range histograms (*p ≤ 0.05 compared with the TBI group). TBI group—brain traumatized rats that received 30 µl PBS intranasally (n = 5), TBI + GPC-EV group—rats with brain injury, and intranasal administration of GPC-EV (n = 5).

Differential expression of apoptosis-related genes *Bax* and *Bcl2* was observed on post-TBI day 7. Bcl2, a factor of survival, protects the cell from programmed death, whereas Bax is known to induce apoptosis. The intracellular *Bax/Bcl2* expression ratio can modulate the cell’s capacity to react to apoptotic stimuli. According to this concept, a cell with higher *Bax/Bcl2* ratio will be more sensitive to pro-apoptotic stimuli compared to similar cells with lower *Bax/Bcl2* ratio*.* The GPC-EV administration significantly reduced *Bax* mRNA levels in the affected cortical area as compared with the TBI group, whereas corresponding *Bcl2* mRNA levels were similar; at that, the *Bax*/*Bcl2* expression ratio for the treatment group was 2.4 times lower compared with the TBI group (Fig. 7A, p < 0.05). In the striatum and hippocampus, *Bax* and *Bcl2* expression levels, as well as their ratio, showed no significant differences with regard to the treatment (Fig. 7B,C).

**Figure 8:**
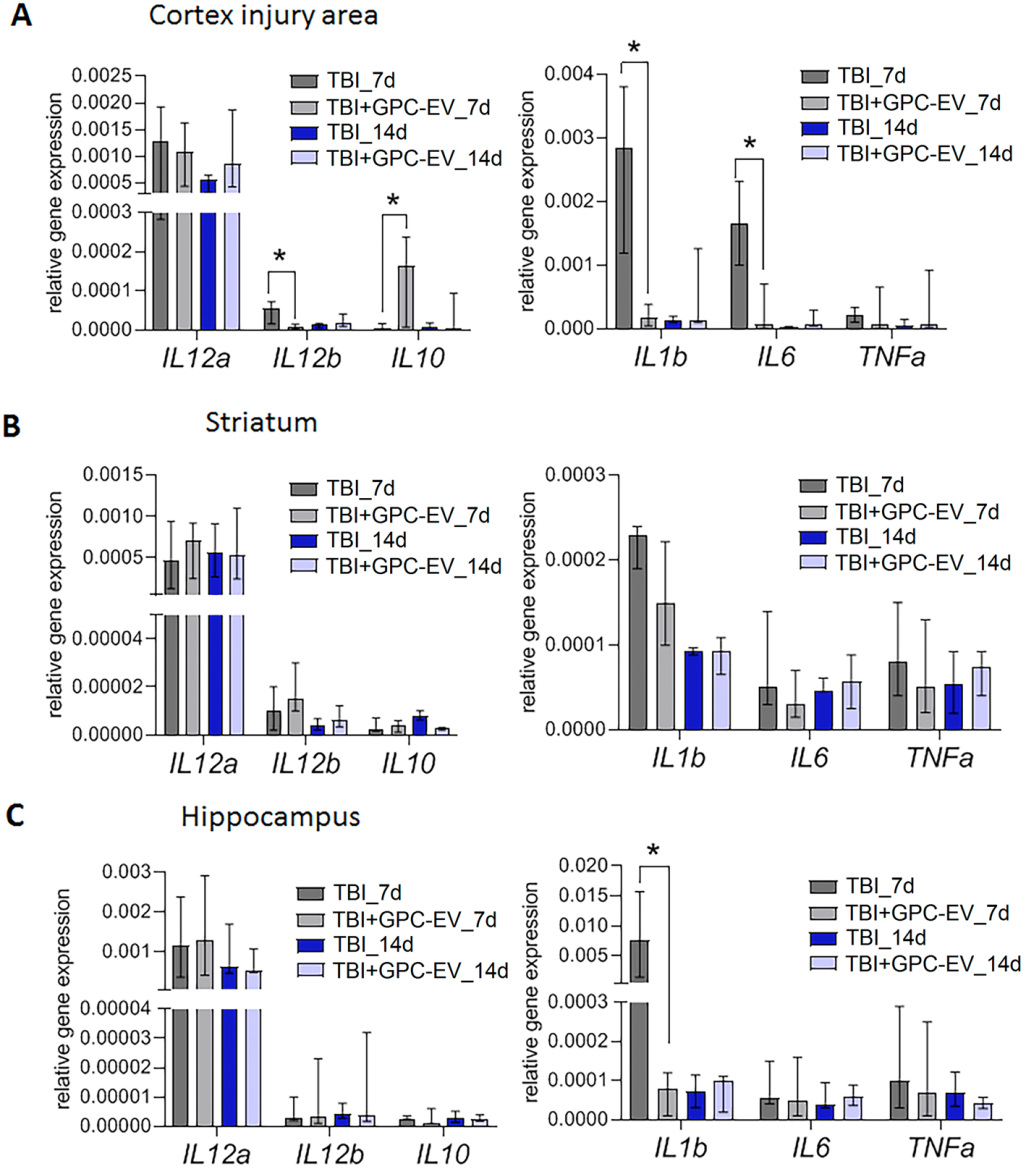
mRNA expression levels of markers of inflammation in different brain regions: (**A**) cortex (traumatic lesion); (**B**) striatum; (**C**) hippocampus. Reverse transcription PCR data for mRNA analyzed by Kruskal–Wallis test with post hoc Dunn’s test are presented as median with range histograms (*p ≤ 0.05 compared with the TBI group). TBI group—brain traumatized rats that received 30 µl PBS intranasally (n = 5), TBI + GPC-EV group—rats with brain injury, and intranasal administration of GPC-EV (n = 5).

Brain traumas inflict secondary damage by triggering neuroinflammation accompanied by massive production of hazardous pro-inflammatory signaling molecules, notably cytokines IL-6, IL-1b, IL-12, TNF-⍺, etc., which can spread to other brain regions and promote neuroinflammatory responses outside the primary lesion. The GPC-EV administration significantly reduced expression levels of *Il6* (24-fold), *Il12b* (ninefold) and *Il1b* (16-fold) in the affected cortical area as measured on post-TBI day 7 (Fig. 8A, p < 0.05). At that, expression of the anti-inflammatory cytokine *Il10* was increased 26-fold compared with the TBI group (Fig. 8A, p < 0.05). Reduced expression of pro-inflammatory cytokines was observed in other brain region as well, including a 34-fold decrease for *Il1b* in the hippocampus on post-TBI day 7 (Fig. 8C; p < 0.05). No significant changes were observed in striatum (Fig. 8B).

**Figure 9:**
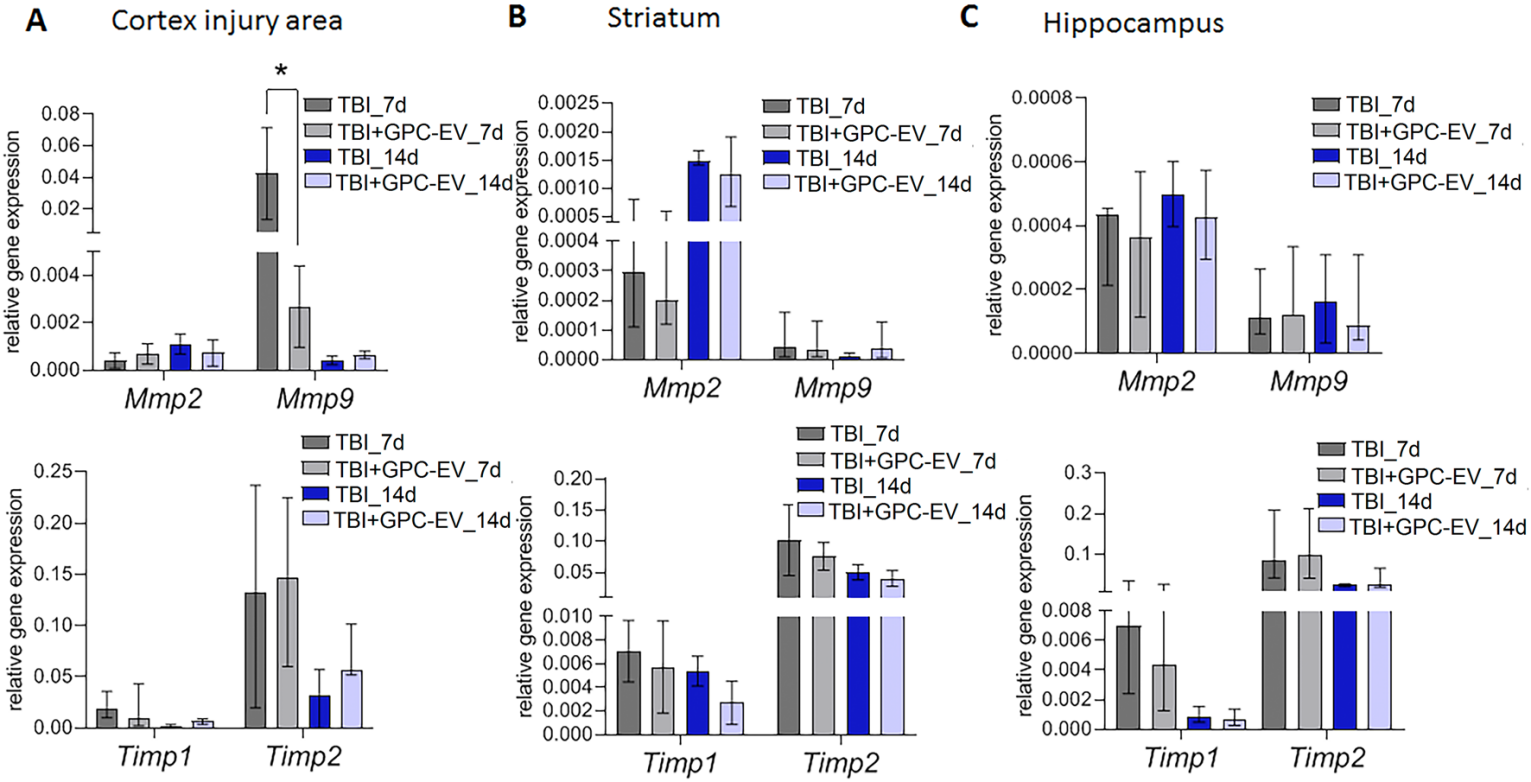
mRNA expression levels of extracellular matrix remodeling markers in different brain regions: (**A**) cortex (traumatic lesion); (**B**) striatum; (**C**) hippocampus. Reverse transcription PCR data for mRNA analyzed by Kruskal–Wallis test with post hoc Dunn’s test are presented as median with range histograms (*p ≤ 0.05 compared with the TBI group). TBI group—brain traumatized rats that received 30 µl PBS intranasally (n = 5), TBI + GPC-EV group—rats with brain injury, and intranasal administration of GPC-EV (n = 5).

Elevated expression of matrix metalloproteinases (MMPs) by neurons, astrocytes, microglia and endothelial cells of the brain is a typical response to TBI, whereas the baseline MMPs expression in the brain is known to be very low. The GPC-EV administration significantly mitigated such response from *Mmp9* (a 16-fold reduction on post-TBI day 7) within the affected cortical area (Fig. 9A, p < 0.05). No significant differences were observed in other brain regions (Fig. 9B,C).

**Figure 10:**
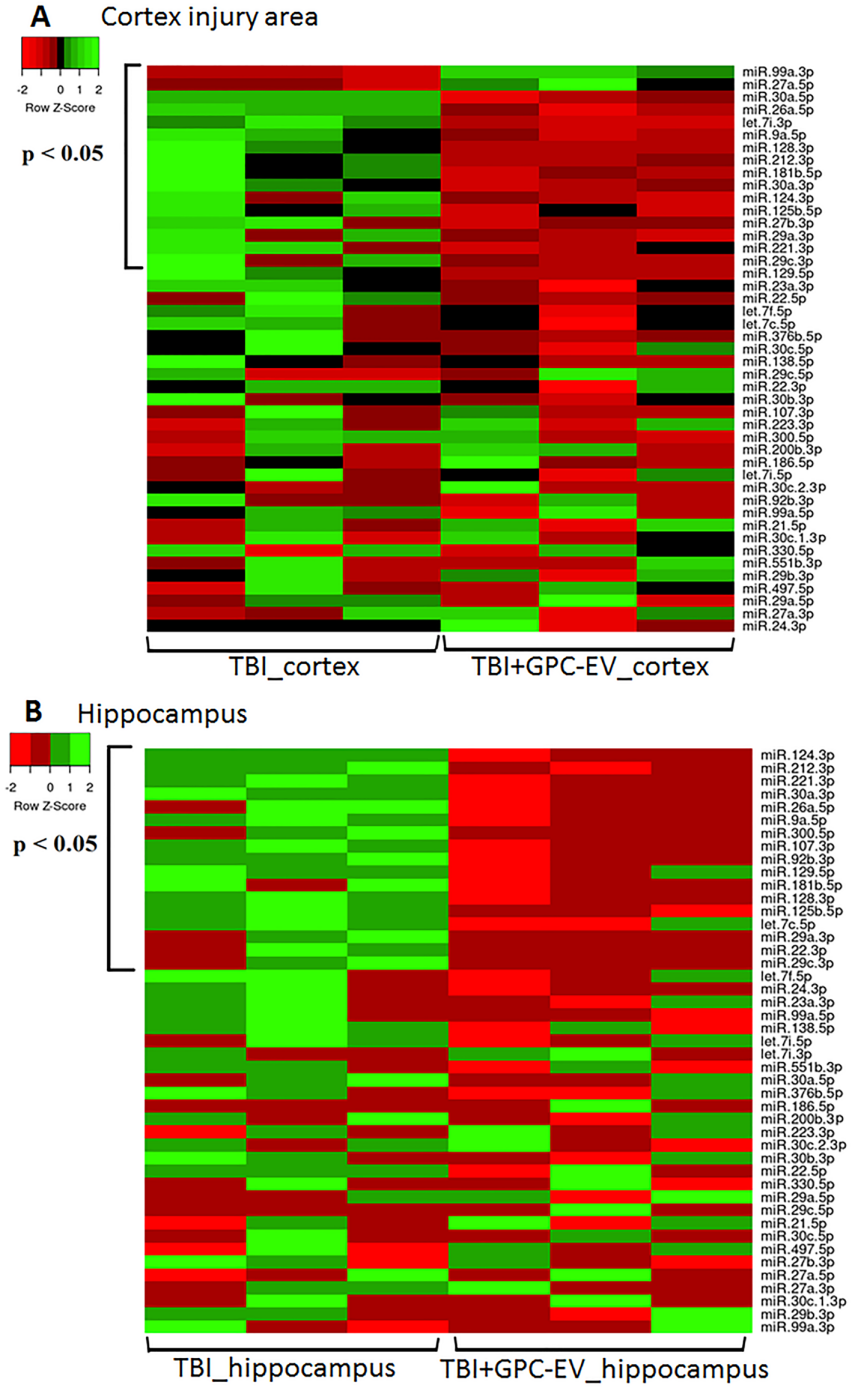
miRNA expression level in different brain regions: (**A**) cortex (traumatic lesion), (**B**) striatum. Real-time PCR data for miRNA on post-TBI day 7 analyzed by *t*-test are presented as heatmaps (*p ≤ 0.05 compared with the TBI group). TBI group—brain traumatized rats that received 30 µl PBS intranasally (n = 3), TBI + GPC-EV group—rats with brain injury, and intranasal administration of GPC-EV (n = 3).

The influence of intranasal GPC-EV administration post-TBI on miRNA signatures in the affected cortical area, striatum and hippocampus were analyzed. By accessing the miRWalk 2.1 was selected 45 miRNAs implicated in the regulation of apoptosis, inflammation and neurogenesis in brain tissues (see “Methods” section) to analyze their expression on post-TBI day 7. This time point was chosen for the analysis as post-TBI day 7, not day 14, presented with the most profound alterations in protein-coding gene expression associated with the treatment. The analysis revealed significant differences in miRNA expression levels between the groups of the study within the affected cortical area (Table S3, Fig. 10A) and the hippocampus (Table S4, Fig. 10B). Striatum tissues presented with borderline significance of differences for several miRNAs (Table S5, Fig. S2). It is important to note that the higher ∆Ct for particular miRNA, the lower its expression level.

The use of MiRTargetLink database (https://ccb-compute.cs.uni-saarland.de/mirtargetlink2/bidirectional_search/) to reveal potential transcriptomic links between miRNA and their target genes at the cortical site of injury revealed several inversely correlated pairs, notably miR-26a-5p/*IL6*, miR-128-3p/*IL6*, miR-128-3p/*Bax*, miR-29a-3p/*Bax*, miR-29c-3p/*Bax* and miR-129-5p/*IL12b*, indicating direct anti-inflammatory and anti-apoptotic roles of corresponding miRNAs. However, indirect effects of miRNA on protein-coding gene expression should be considered as well. For example, an increase in miR-30a-5p expression may reduce the amounts of transcription factors KLF9 and ATF1 binding the enhancer regions of *MMP9* and *IL1B* according to the GeneCards database (https://www.genecards.org/). This regulatory chain of events may explain the observed decrease in the expression levels of *MMP9* and *IL1B* within the affected cortical area of the treatment group animals as compared with the TBI group. In a similar manner, the reduced expression of miR-99a-3p could indirectly facilitate *IL10* through a reduction targeting of *BCL11A*, *TBL1XR1*, *MEIS2* (https://www.genecards.org/), which bind to enhancer regions of *IL10* promoter. In the present study significant increase of *IL10* expression level was observed within the affected cortical area in response to the treatment. Similarly, elevated levels of miR-221-3p could inhibit *CDKN1B*, the cyclin-dependent kinase inhibitor 1B, thereby promoting cell survival through increased expression of *Bcl2*^20^.

Analysis of target genes for 17 miRNAs with significantly elevated hippocampal expression levels (Table S4) using the target gene overrepresentation analysis algorithm in MiRTargetLink database (BioCarta Pathways) revealed their participation in regulation of p53 Signaling Pathway (*APAF1*, *ATM*, *BAX*, *BCL2*, *CCND1*, *CCNE1*, *CDK2*, *CDK4*, *CDKN1A*, *E2F1*, *MDM2*, *PCNA*, *RB1*, *TIMP3*, *TP53*); Cytokine Network (*CXCL8*, *IFNB1*, *IL10*, *IL1b*, *IL6*, *TNF*, *TXLNA*); and Cytokines and Inflammatory Response (*CSF1*, *CXCL8*, *HLA-DRA*, *IFNB1*, *IL1b, IL10*, *IL11*, *IL1A*, *IL6*, *IL7*, *PDGFA*, *TGFB3*, *TNF*) modules.

### Levels of active caspase-9

**Figure 11:**
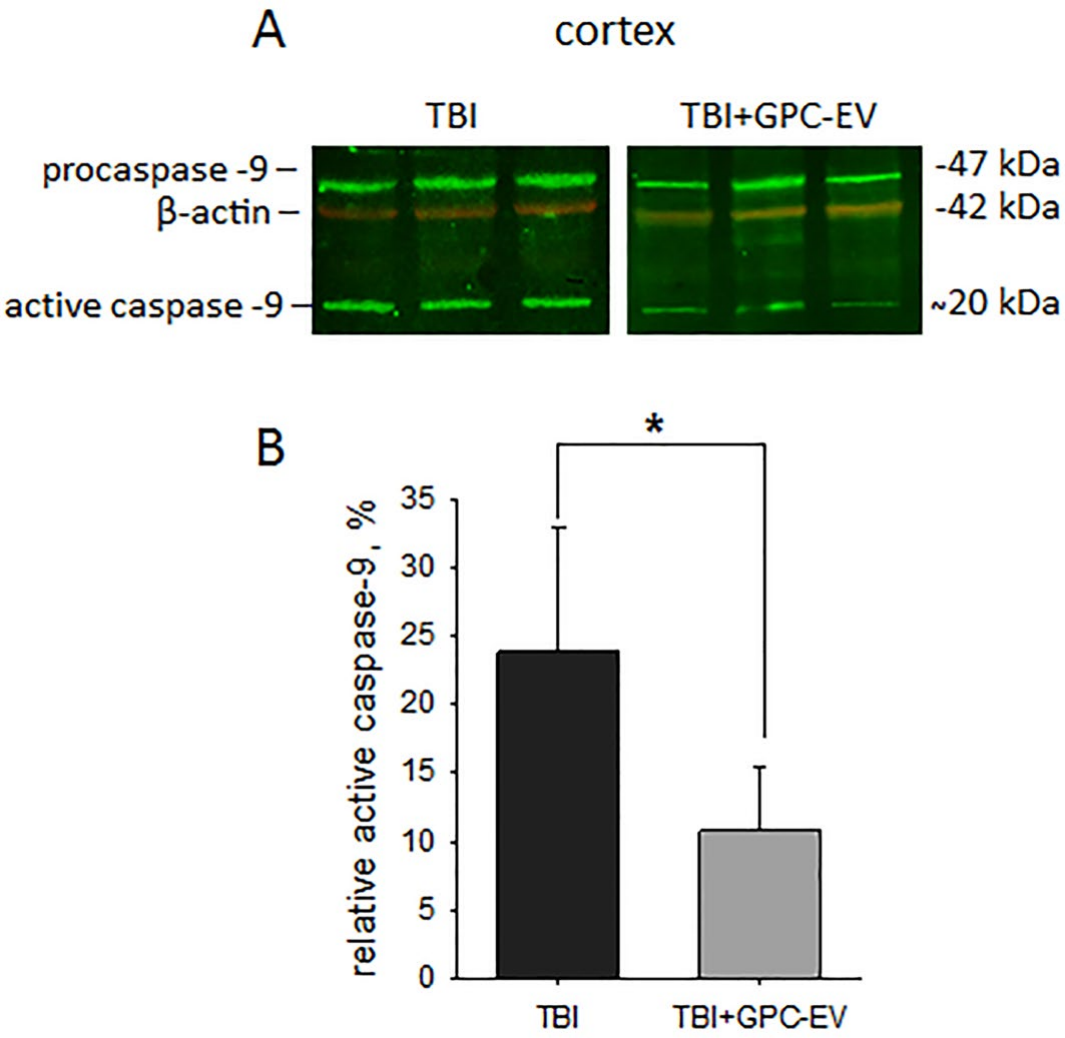
Immunoblotting for caspase-9 in the site of cortical damage: (**A**) semi-quantitation data presented as means ± SD; *p ≤ 0.05 compared with the TBI group (t-test); (**B**) representative blots. TBI group—brain traumatized rats that received 30 µl PBS intranasally (n = 4), TBI + GPC-EV group—rats with brain injury, and intranasal administration of GPC-EV (n = 6).

Injuries after TBI lead to the death of neurons. Caspases activated by proteolytic cleavage are the main regulators of apoptotic cell death. Caspase-9 is a main effector enzyme of neuronal apoptosis. According to PCR analysis, the level of active caspase-9 in the site of cortical damage was assessed. The level of active caspase-9 was twofold lower in the experimental group compared with the TBI group (p < 0.05; Fig. 11).

### Comparative study of NF-κB and neurodegeneration marker protein levels

**Figure 12:**
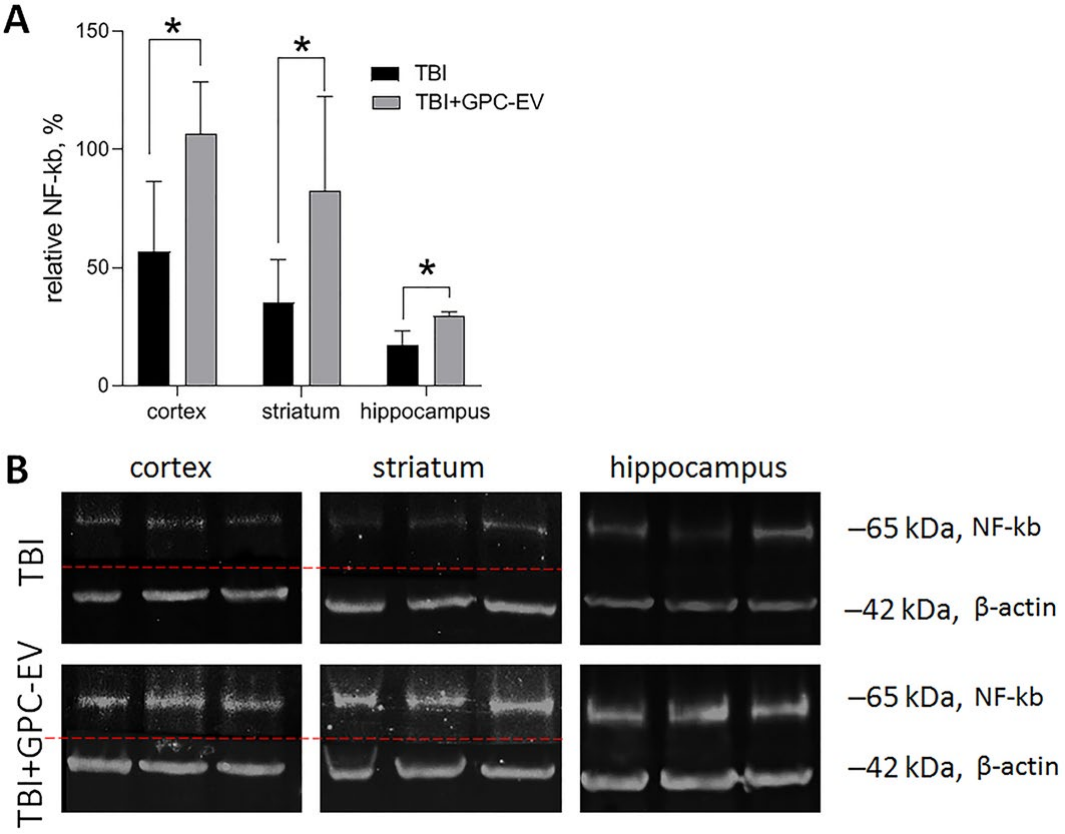
Analysis of the NF-κB levels on post-TBI day 7. (**A**) Semiquantitative assessment of protein levels for NF-κB. (**B**) Representative blots. Lines divided grouping blots from two parts of the same gel. Original blots are presented in Supplementary Fig. S5. The data are presented as mean ± standard deviation; *p ≤ 0.05 compared with the TBI group (*t*-test). TBI group—brain traumatized rats that received 30 µl PBS intranasally (n = 5), TBI + GPC-EV group—rats with brain injury, and intranasal administration of GPC-EV (n = 6).

NF-κB expression has been demonstrated in all cell types of the brain. This transcription factor participates in multiple signaling cascades and has been shown to play major roles in the pathogenesis of various inflammatory conditions, as well as in neuronal plasticity, neuronal differentiation and neurodegeneration. Baseline NF-κB expression in neurons is essential for their normal functioning involving synaptogenesis and synaptic plasticity, whereas its elevated levels can ensure neuroprotection in certain pathologies^21^. In this study, NF-κB expression levels on post-TBI day 7 were assessed. The GPC-EV administration promoted a significant increase in the NF-κB protein content for all studied brain regions, as compared with the TBI group (Fig. 12). These data suggest that GPC-EV effects are NF-κB-dependent.

**Figure 13:**
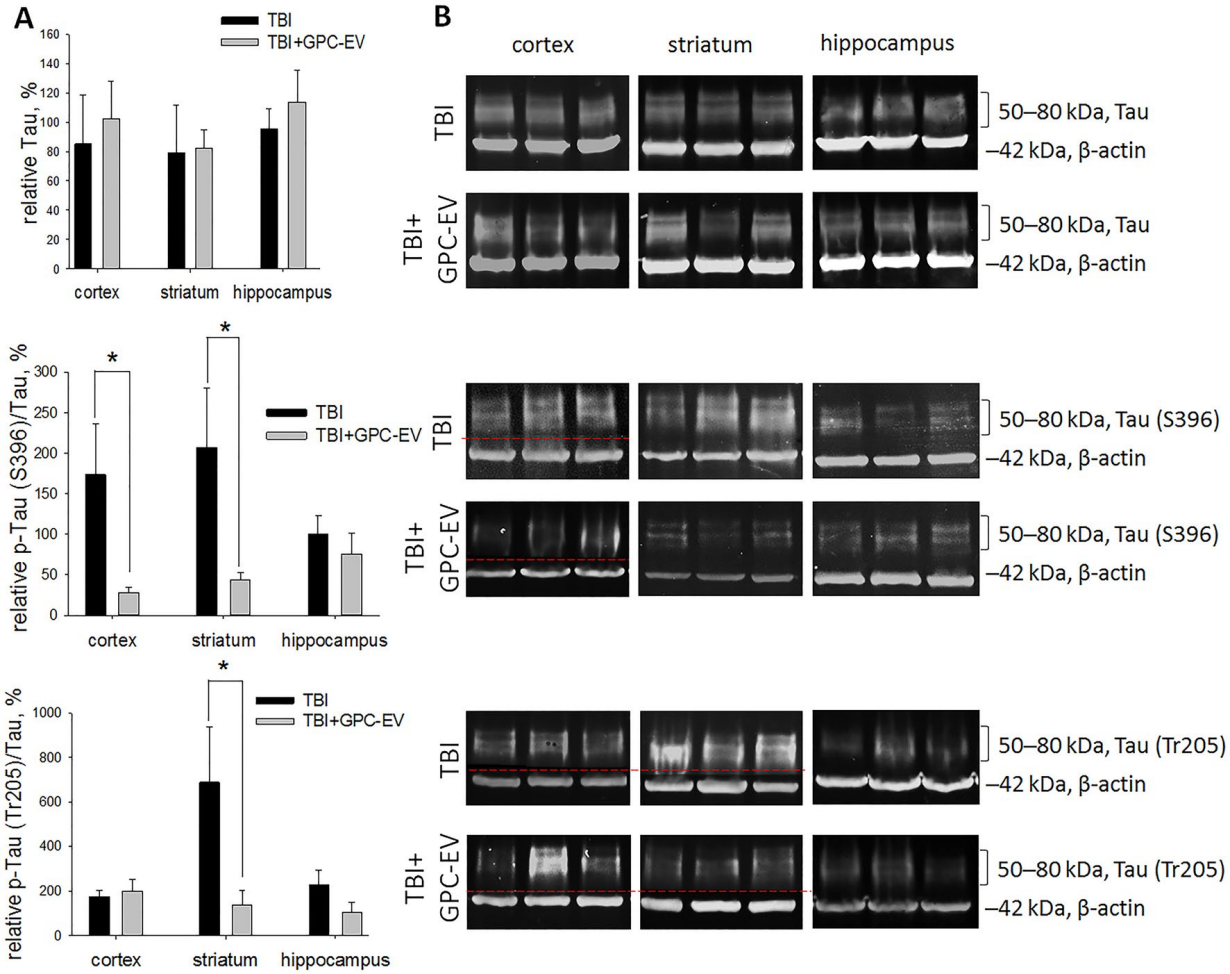
Analysis of the neurodegeneration marker levels on post-TBI day 14. (**A**) Semiquantitative assessment of protein levels for Tau, p-Tau (Ser396) and p-Tau (Thr205) on post-TBI day 14. (**B**) Representative blots. Lines divided grouping blots from two parts of the same gel. Original blots are presented in Supplementary Fig. S5. The data are presented as mean ± SD; *p ≤ 0.05 compared with the TBI group (*t*-test). TBI group—brain traumatized rats that received 30 µl PBS intranasally (n = 4), TBI + GPC-EV group—rats with brain injury, and intranasal administration of GPC-EV (n = 5).

The active Tau protein is indispensable for microtubule assembly and stabilization in axons, whereas its hyperphosphorylation disrupts these processes with disastrous consequences for brain architecture. The Tau levels in all brain regions were similar after GPC-EV administration compared with untreated animals (Fig. 13). The hyperphosphorylated Tau isoforms are inactive, as p-Tau (Thr205) has altered conformation, and p-Tau(Ser396) tends to aggregate^22,23^. The extent of Tau hyperphosphorylation in the studied brain regions was expressed as a content ratio between p-Tau (including both p-Ser396 and p-Thr205 isoforms) and non-phosphorylated Tau. Within the cortical traumatic lesion at the site of injury, the treatment caused a sixfold decrease for p-Tau(Ser396)/Tau, but not p-Tau (Thr205)/Tau. In the striatum, both ratios p-Tau(Ser396)/Tau and p-Tau (Thr205)/Tau were about 5 times lower compared with the TBI group. In the hippocampus, there are no statistically differences between compared groups (Fig. 13).

## Discussion

Neuroprotective effects exerted by EV of various cellular origins have been convincingly demonstrated in recent studies^23^. Advantages of prospective EV-based therapies include the receptor-mediated selective binding of the vesicles to target cells, and smooth delivery of the content preserved by a lipid membrane. The use of EV isolates instead of cell and tissue transplants alleviates certain associated risks, notably those of embolism and immune rejection^24^. Still, a decisive bench-to-bedside transition of EV-based techniques would require extensive clinical research, and mechanistic insights into their therapeutic potential.

The choice of cells for EV production appears pivotal. Glial cells are unique in many aspects of their natural connection to neurons, including their roles in metabolic support and mechanical scaffolding, as well as post-traumatic repair of neural structures^25,26^. Accordingly, GPC cultures are considered a likely candidate source for post-TBI therapies, especially given their capacity to produce certain neurotrophic factors expressed at early developmental stages of the brain.

EVs are known to contain miRNAs which inhibit gene expression at the post-transcriptional level. MiRNAs specifically bind their target transcripts to prevent translation, and thereby can influence various cellular processes^27^ and contribute to diverse some processes, including apoptosis, inflammation and neurodegeneration. The sequence of many miRNAs is found to be highly conserved, in their mature form, among different organisms, and they can influence on various processes bypassing species specificity^28–30^. Accordingly, the miRNA content of GPC-EV should not be overlooked from a therapeutic perspective. In this study, EVs from three independent human GPC cultures were characterized, and identified a total of 203 miRNAs, some of them attributed to neuroprotective properties. Simultaneous delivery of these molecules as a GPC-EV cargo may trigger complex neuroprotective responses further amplified by the multiplicity of target transcripts for each particular miRNA.

Intranasal administration of such EV, as shown by many data, can be established as an effective and reliable way to bypass the blood–brain barrier, and deliver drugs to the central nervous system. Exploitation of such direct route occurs due to the olfactory region. In addition, the intranasal administration increases the bioavailability of drugs in the brain, since it prevents their absorption through the gastrointestinal tract and hepatic metabolism^31^. For example, EV obtained from mesenchymal stromal cells (MSC-EV) were targeted and accumulated in damaged regions associated with stroke, brain injury, and Alzheimer’s disease for up to 96 h after intranasal administration in mouse models of the disease, while they showed diffuse migration and were cleared in 24 h in the healthy brain^32^. The accumulation of EV was closely correlated with the neuroinflammatory signal in the damaged brain, which suggests that the homing mechanism is due to inflammation. These data indicate that EV have the ability to target and accumulate in various types of cells and in damaged areas of the brain^31^.

In our study, intranasal administration of GPC-EV in rat TBI model were demonstrated significant functional recovery with improved sensorimotor output from the affected hemisphere, but without reducing the damage volume in the rat brain. Studies by other authors also demonstrate the enhanced post-TBI functional recovery in animals treated with EV obtained from different types of cell cultures^33–35^. Sun et al. observed significant improvement in locomotor performance 4 weeks post-TBI in response to EV derived from neural stem cells^34^, whereas Zhang et al. encountered no significant reduction in infarction volume in response to MSC-EV, although the therapy significantly improved sensorimotor deficit^35^.

GPC-EV infusions were shown to mitigate the inflammatory reaction and apoptosis. In response to the treatment, significant reduction in the amount of astroglia, witch capable of inhibiting neurorecovery, as well as suppression of monocytic macrophages (CD68^+^ cells) at the site of injury, and decline of microglia (iba1^+^ cells) in the hippocampus. In addition, GPC-EV treatment was led to decrease the level of active caspase-9 in damaged cortex. In molecular level, the reduction on pro-apoptotic and inflammatory marker genes were also shown. The evidence of the mitigated inflammatory reaction and apoptosis is supported by molecular studies including those featuring miRNAs as plausible neuroprotective agents with anti-inflammatory and anti-apoptotic properties^36^. In particular, the expression of certain miRNAs was shown to correlate with reduced expression of pro-inflammatory and pro-apoptotic genes in brain tissues. For instance, EV derived from MSCs of bone marrow origin inhibited the release of pro-inflammatory factors and early inflammatory reactions while stimulating the antiapoptotic BCL-2 expression, thus regulating the microglia/macrophage polarization with an overall neuroprotective impact in post-TBI brain recovery^37^. In another study, EV derived from human MSCs of umbilical cord origin were shown to inhibit microglia proliferation^38^. In addition, the EV polarized the microglia towards M2 and inhibited expression of pro-inflammatory IL-6 while supporting IL-4 and IL-10 production, thereby mitigating the TBI-induced neuroinflammation.

Another important aspect of the injured brain response to EV administration identified in this study involves NF-κB that has diverse functions in the nervous system, depending on the cellular context. Neuronal NF-κB involvement in synaptic processes, and their inhibition resulted in the loss of neuroprotection. Activation of NF-κB led it translocation to the nucleus and activated processes such as axon growth. In glia, NF-κB is regulates inflammatory processes, and its inhibition might ameliorate disease^39^. Also, this factor is characterized by two-phase activation. For example, in a model of ischemic brain injury in rat pups, the primary increase in NF-kB (0–3 h) showed a negative effect, while the second peak in a later period (after 24 h) had a neuroprotective effect^40^. So, in our study observed promotion of NF-kB was on 7 day post-TBI, while an increase in pro-inflammatory cytokines was not detected during this time period. It should be assumed that such increase of NF-kB is realized in neurons. It is possible that this type of cells is a target for GPC-EV. The key molecule involved in the implementation of the NF-kB activation was miR-181b-5p identifying in GPC-EV. This miRNA can activate NF-κB through CYLD inhibition^41^.

Yet another apparent mechanism of GPC-EV therapeutic impact identified in this study involves the structural maintenance of neurons by decreasing phosphorylated Tau forms, plausibly counteracting neurodegeneration. Probably, GPC-EV treatment may effect on brain plasticity. MiR-124-3p is known to exert neuroprotective effects by reducing the abnormal hyperphosphorylation of Tau through PI3K/AKT/GSK-3β signaling pathway regulation^42,43^. The increasing expression of miR-124-3p specifically under the action of GPC-EV was observed in all studied brain regions, reaching statistical significance in the damaged cortex and the hippocampus. This findings are consistent with those of Huang et al., who demonstrate that miR-124-3p-cargoes microglial vesicles prevent neurite shortening by reducing p-Tau levels^44^.

**Figure 14:**
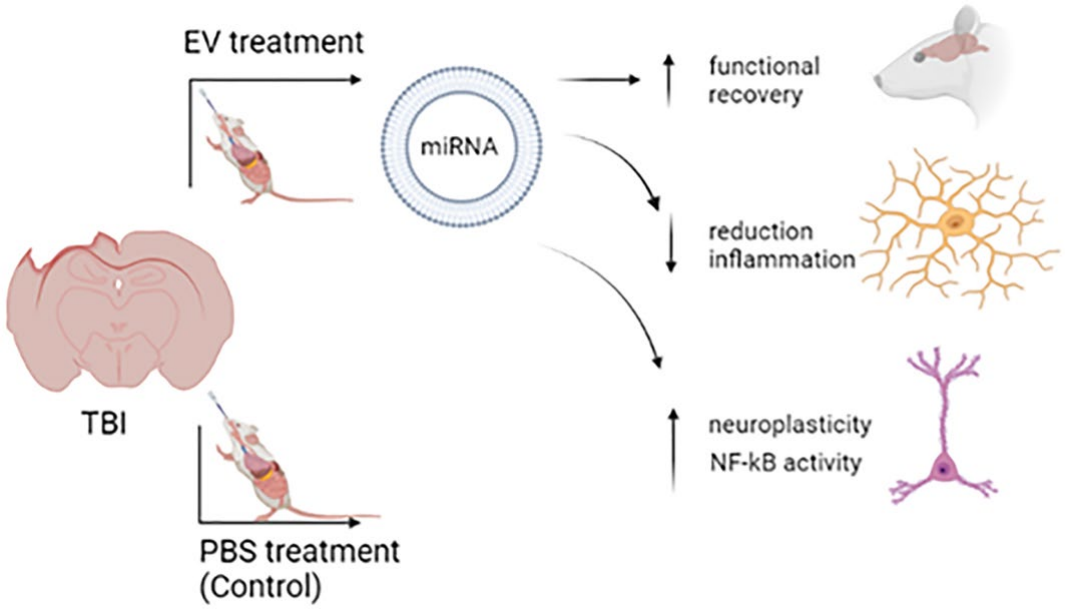
General mechanism of action GPC-EV. Created using https://biorender.com/.

The intranasal administration of GPC-EV has a wide therapeutic potential in TBI model. These therapy exhibit additional advantages due to EV small size, low immunogenicity, and migrating abilities through chemotaxis mediated by neuroinflammation. The therapeutic effect of GPC-EV can be attributed to their ability to transport different proteins, peptides, miRNAs, and other molecules to recipient cells and thereby mediate neurorecovery, reduce inflammation, enhancement neuroplasticity, and NF-kb activity (Fig. 14). These therapy leads to a change miRNAs expression profile in the rat brain, thereby indicating a certain role of miRNAs in GPC-EV cargo. Thus, the safety of GPC-EV and a wide spectrum of therapeutic effects make this therapy more promising, than the cell transplantation. Despite the promising prospects, the further studies are still needed to determine the optimal doses, standardization of EV, as well as determination of signal pathways involved in the mechanism of their action.

## Conclusion

The results suggest that extracellular vesicles secreted by GPCs are able to exert a therapeutic effect in TBI by stimulating neurorecovery and neuroplasticity. The mechanisms are likely to involve suppression of inflammatory response and apoptosis, as well as augmentation of neuronal plasticity by decreasing hyperphosphorylated Tau pools. The observed multimodal effect of extracellular vesicles apparently involves miRNA signatures of both the vesicles and the brain as a key regulator.

### Supplementary Information

Supplementary Information 1.

Supplementary Information 2.

## Data Availability

All data collected or analyzed during this study are included in this article and its additional files. The datasets generated and/or analysed during the current study are available in the GEO NCBI, and INSDC repository, https://www.ncbi.nlm.nih.gov/geo/query/acc.cgi?acc=GSE242996 for real-time quantitative PCR analysis of rat’s brain mRNAs, https://www.ncbi.nlm.nih.gov/geo/query/acc.cgi?acc=GSE242828 for real-time quantitative PCR analysis of rat’s brain miRNAs, and https://www.ncbi.nlm.nih.gov/bioproject/1014362 for miRNAs raw sequence reads.

## Abbreviations

TBI: Traumatic brain injury
GPCs: Glial progenitor cells
EV: Extracellular vesicles
PBS: Phosphate-buffered saline
MRI: Magnetic resonance imaging
PCR: Polymerase chain reaction
NF-κB: Nuclear factor kappa-light-chain-enhancer of activated B cells
iPSCs: Induced pluripotent stem cells
DMEM/F12: Dulbecco’s modified eagle medium/nutrient mixture F12
EGF: Epidermal growth factor
CNTF: Ciliary neurotrophic factor
DNA: Deoxyribonucleic acid
RNA: Ribonucleic acid
Akt: Protein kinase B
Bax: Bcl-2-associated X protein
Bcl2: Apoptosis regulator Bcl-2
IL: Interleukin
MMPs: Matrix metalloproteinases
GFAP: Glial fibrillary acidic protein
