# Supplementary material for: Extracellular vesicles of human glial cells exert neuroprotective effects via brain miRNA modulation in a rat model of traumatic brain injury
Source: Sci Rep. 2023 Nov 21;13:20388. doi: 10.1038/s41598-023-47627-2 (PMC10663567; doi:10.1038/s41598-023-47627-2)
Supplement: Supplementary file 1 — Supplementary Information 1. [file 41598_2023_47627_MOESM1_ESM.pdf]

GPC-EV

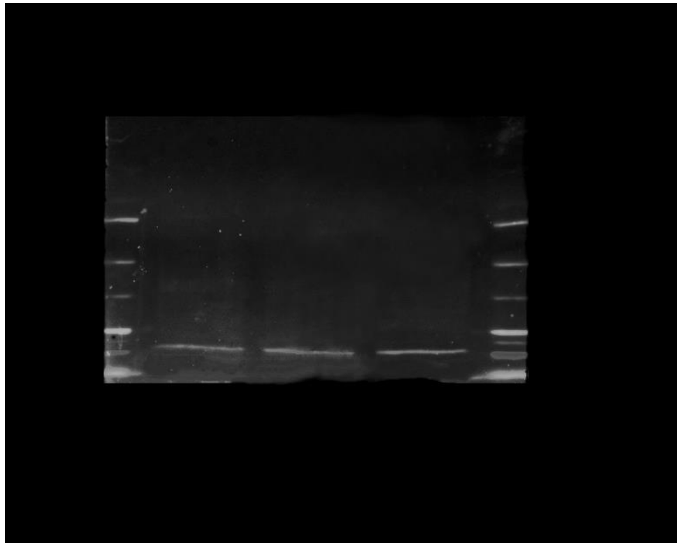

CD63

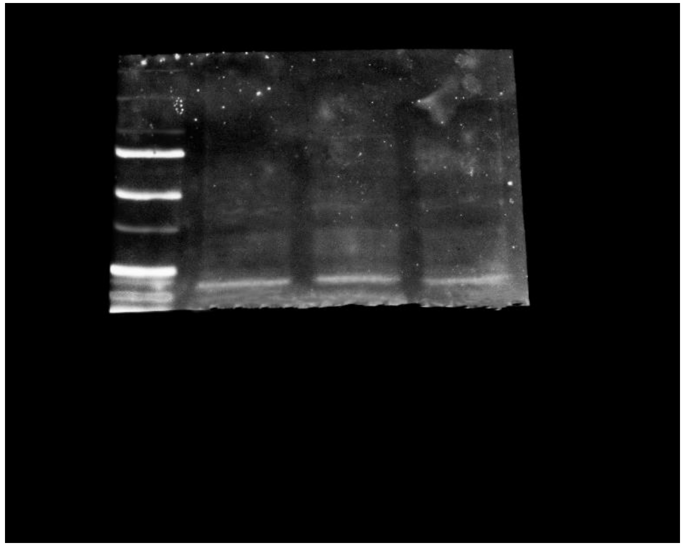

CD9

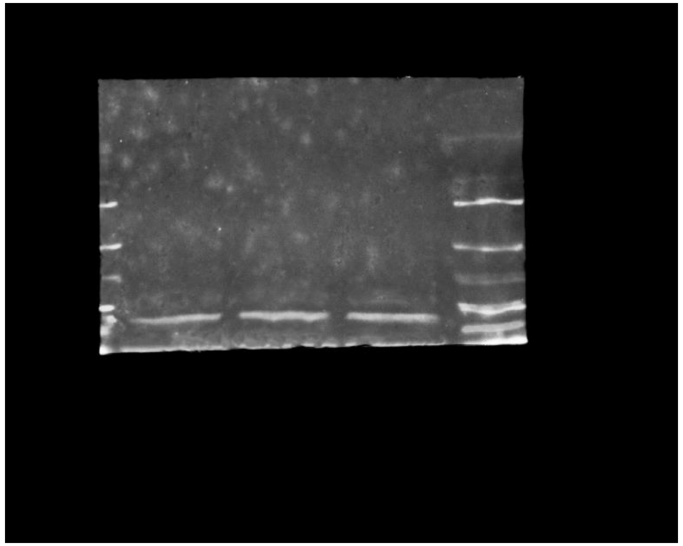

CD81

Caspase-9_cortex_7d

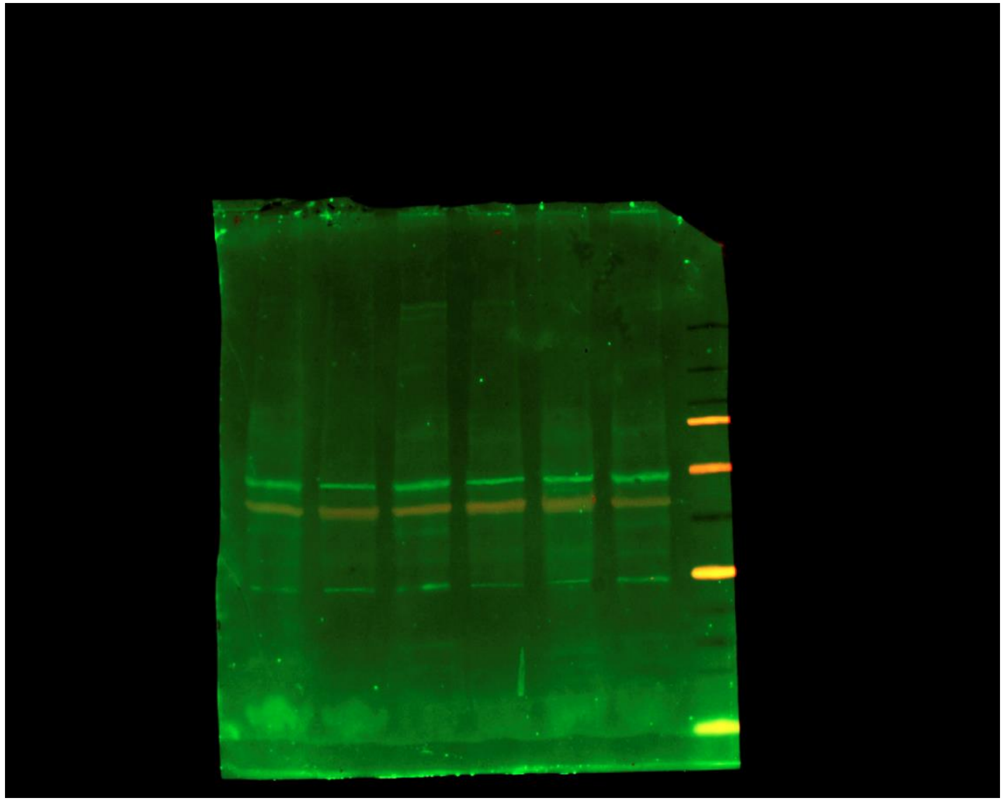

EV12 EV10 EV4 EV16 EV9 EV1_M

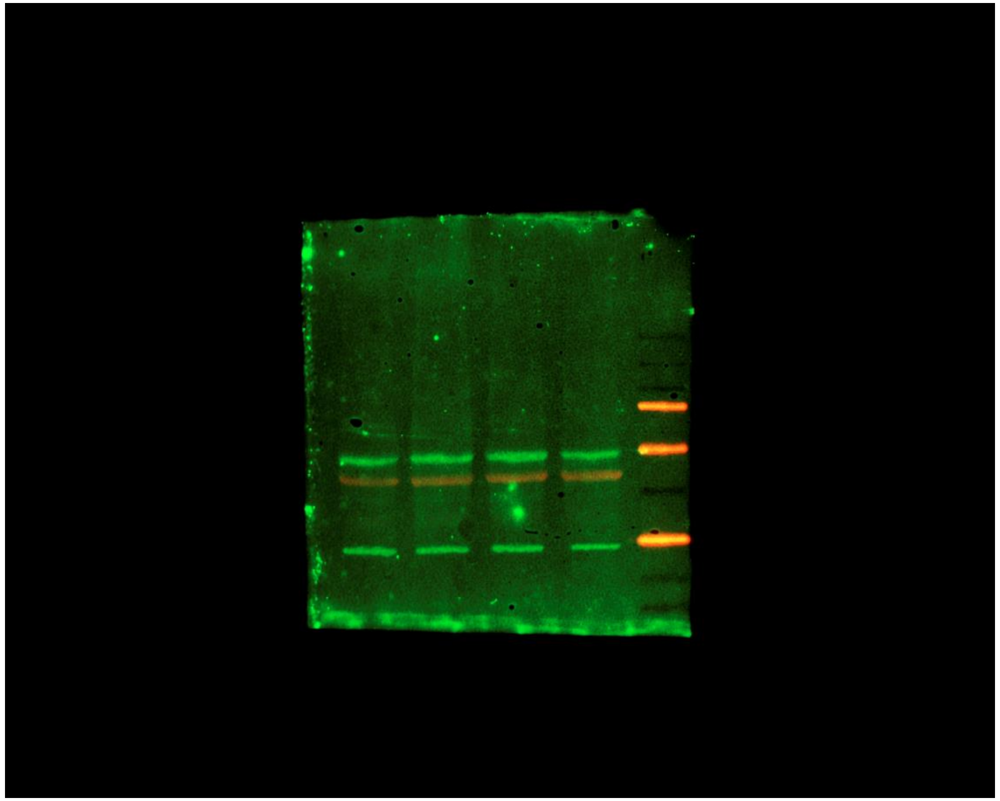

TBI7 TBI15 TBI13 TBI5_M

—procaspase-9
—β-actin
— active caspase-9

Nf-kb_cortex_14d

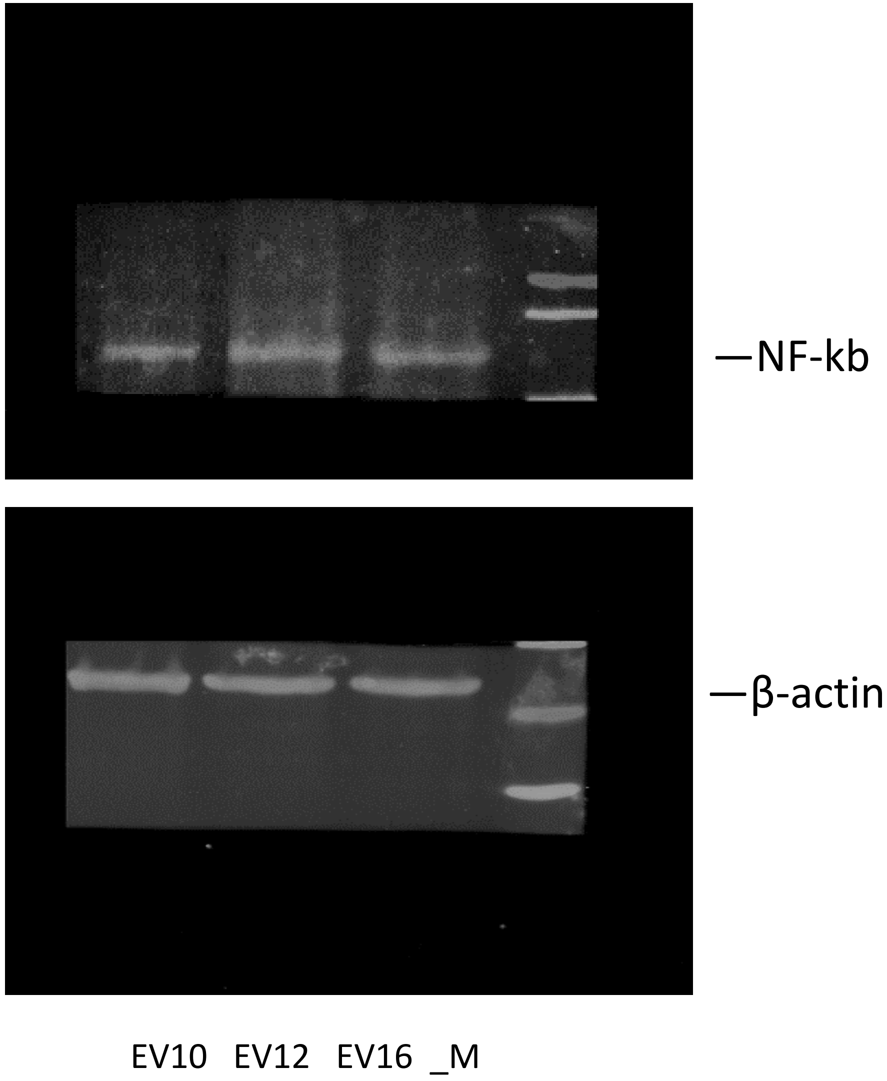

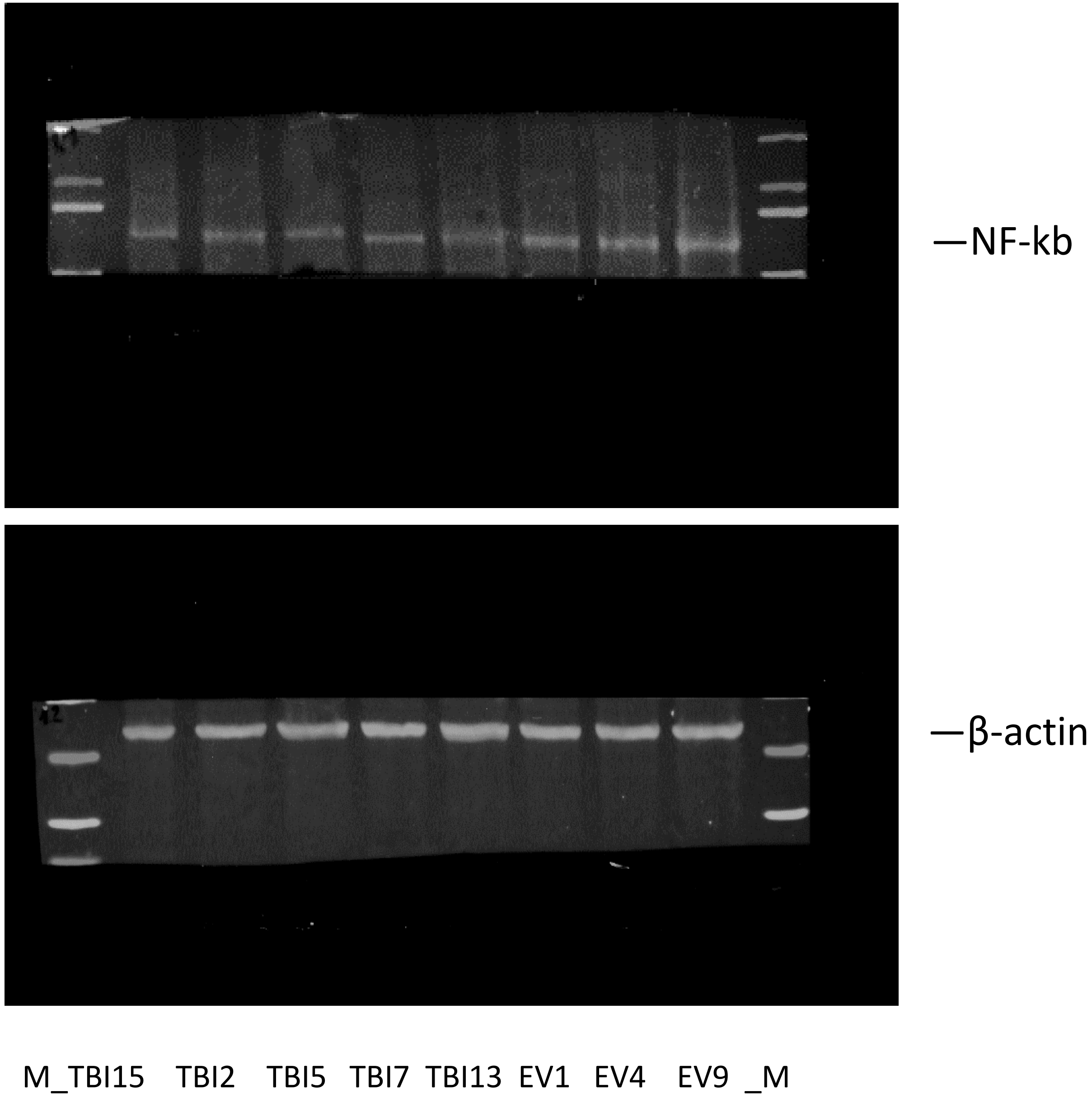

Nf-kb_hippocampus_14d

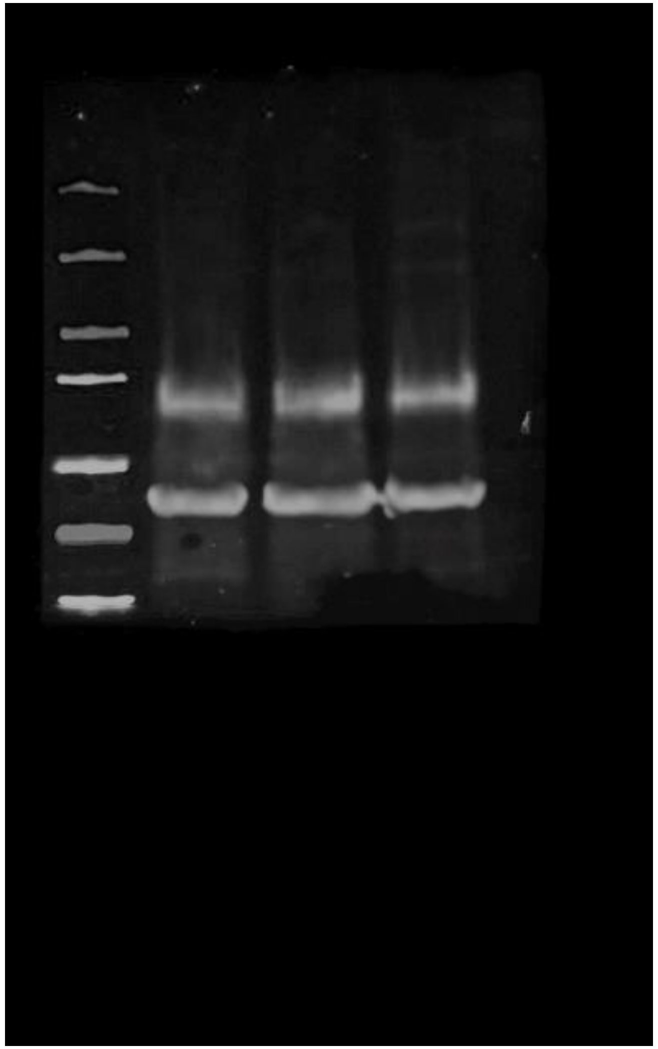

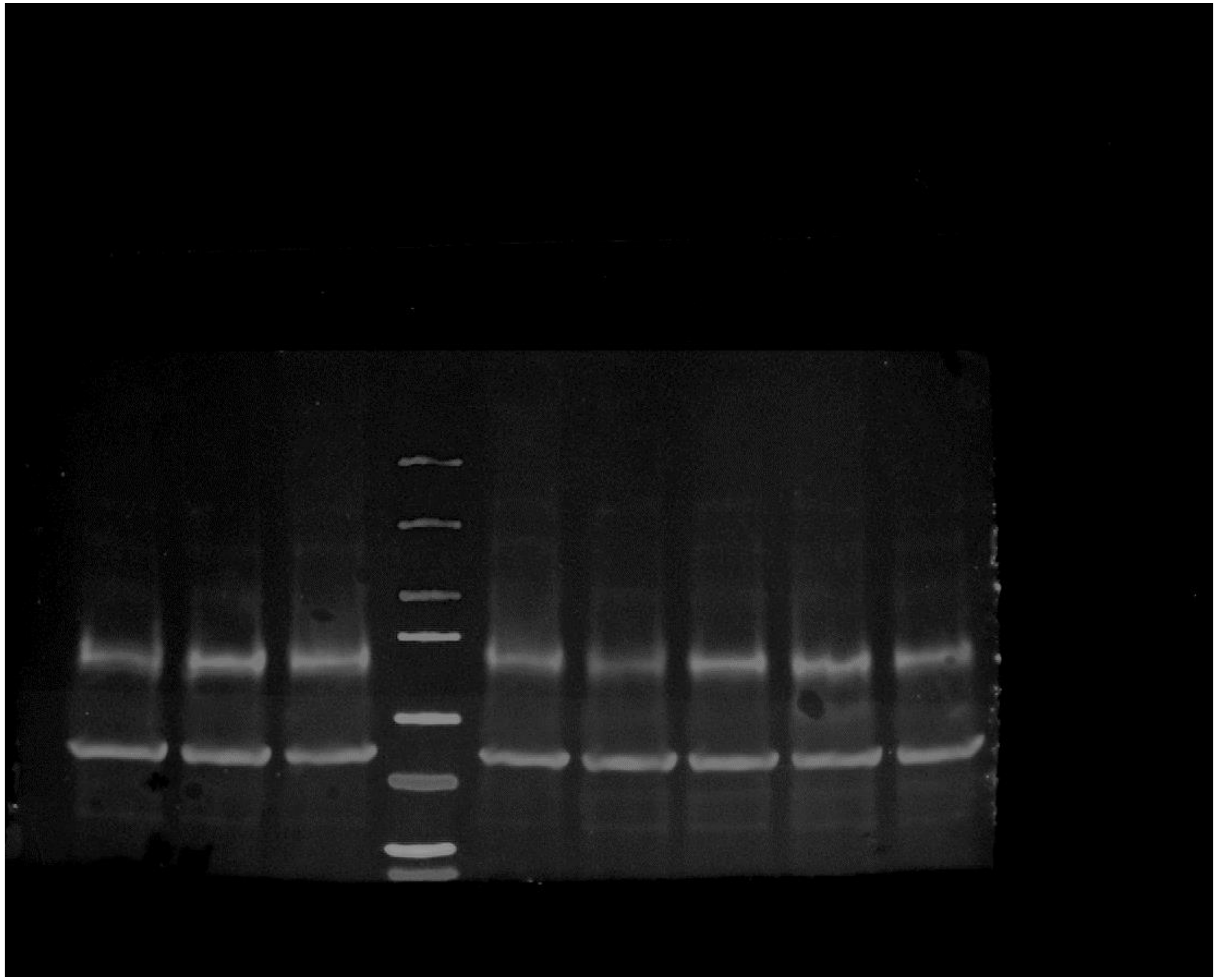

Nf-kb_striatum_14d

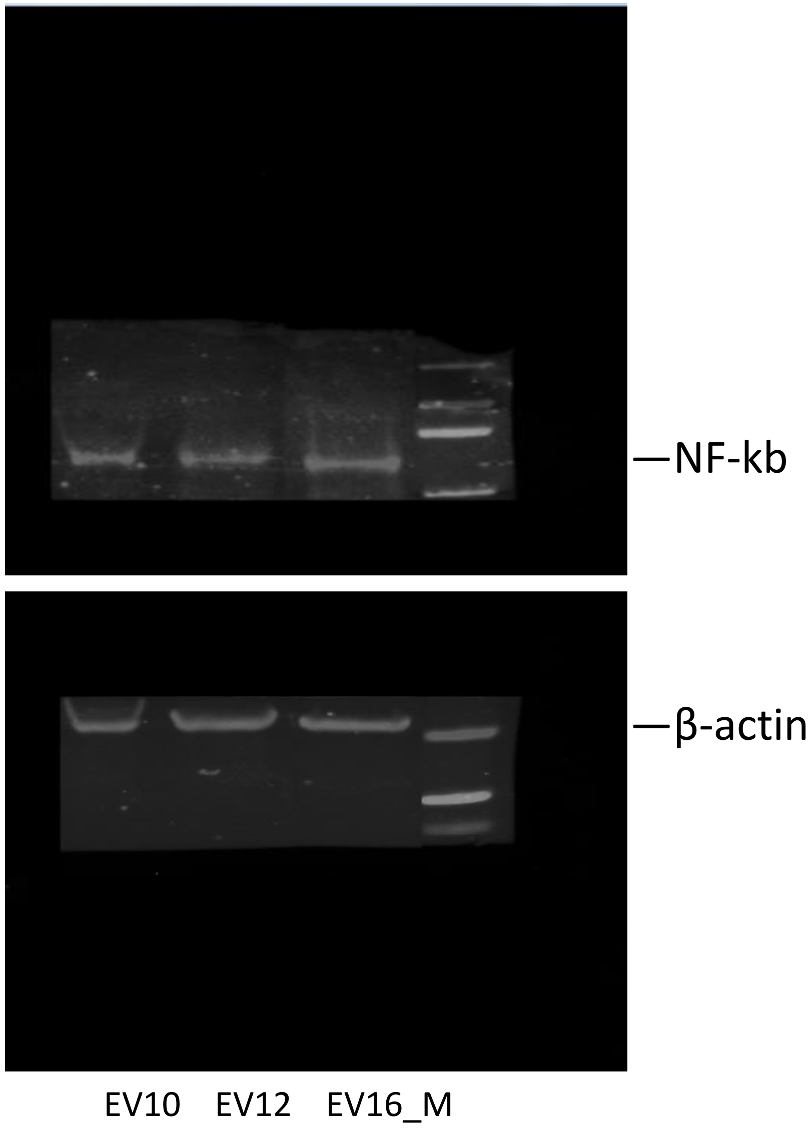

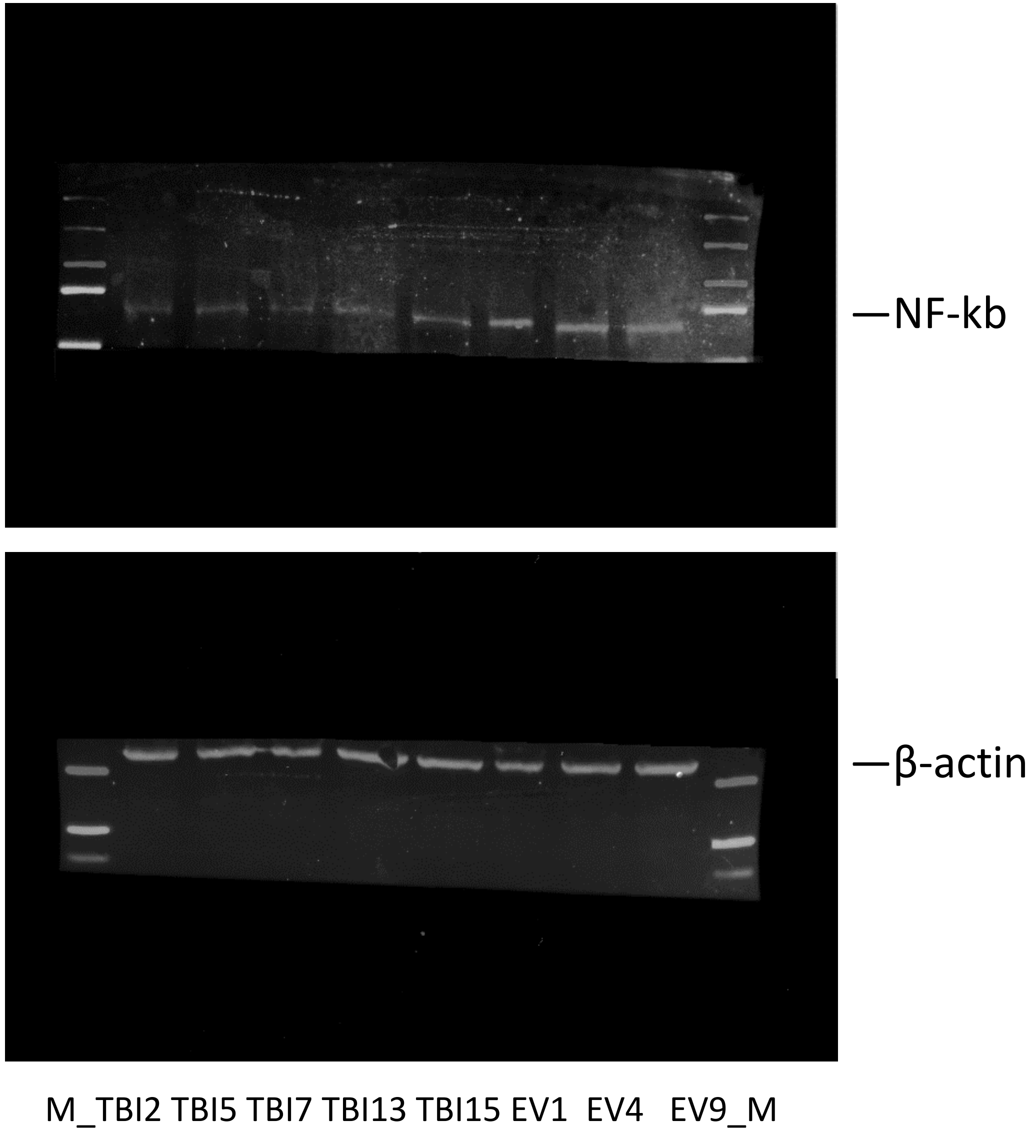

p-Tau (S396)_cortex_14d

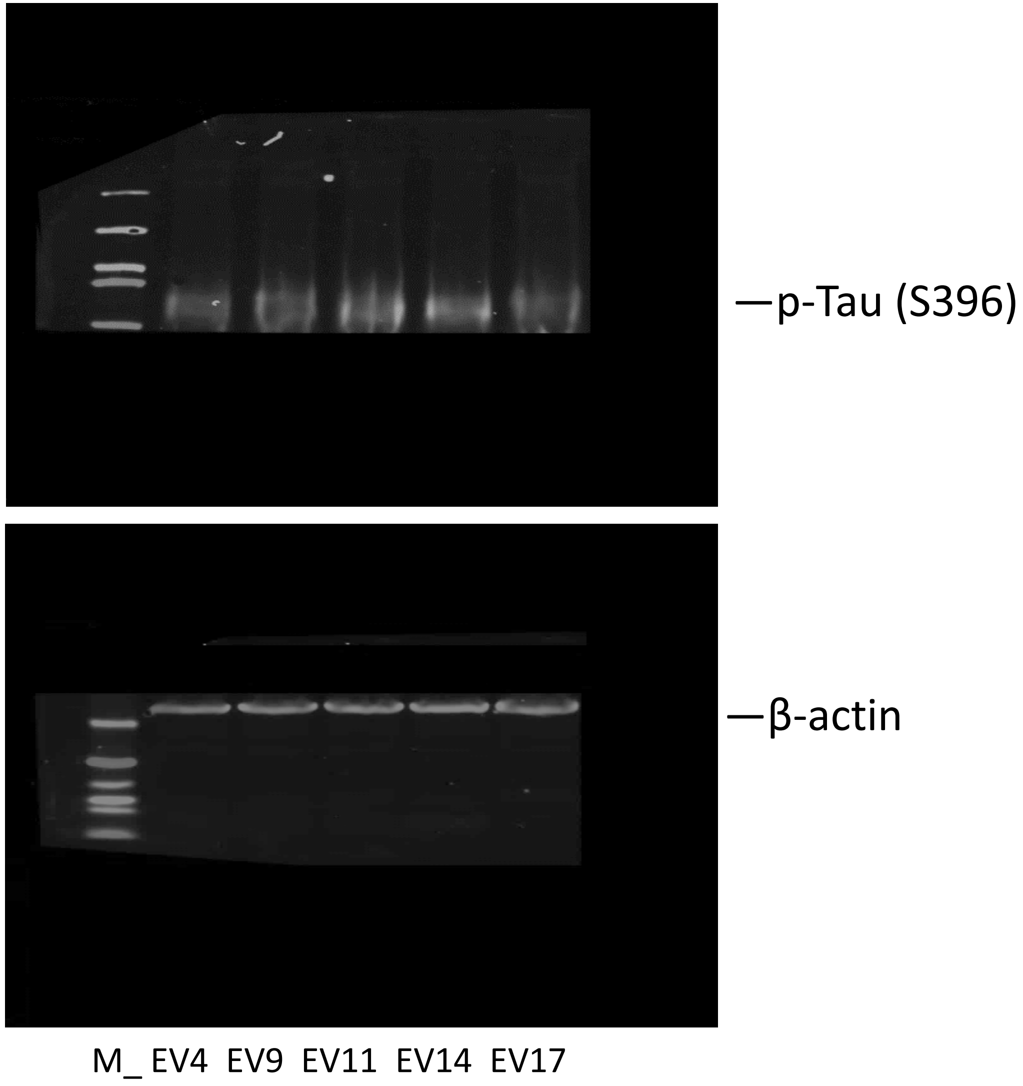

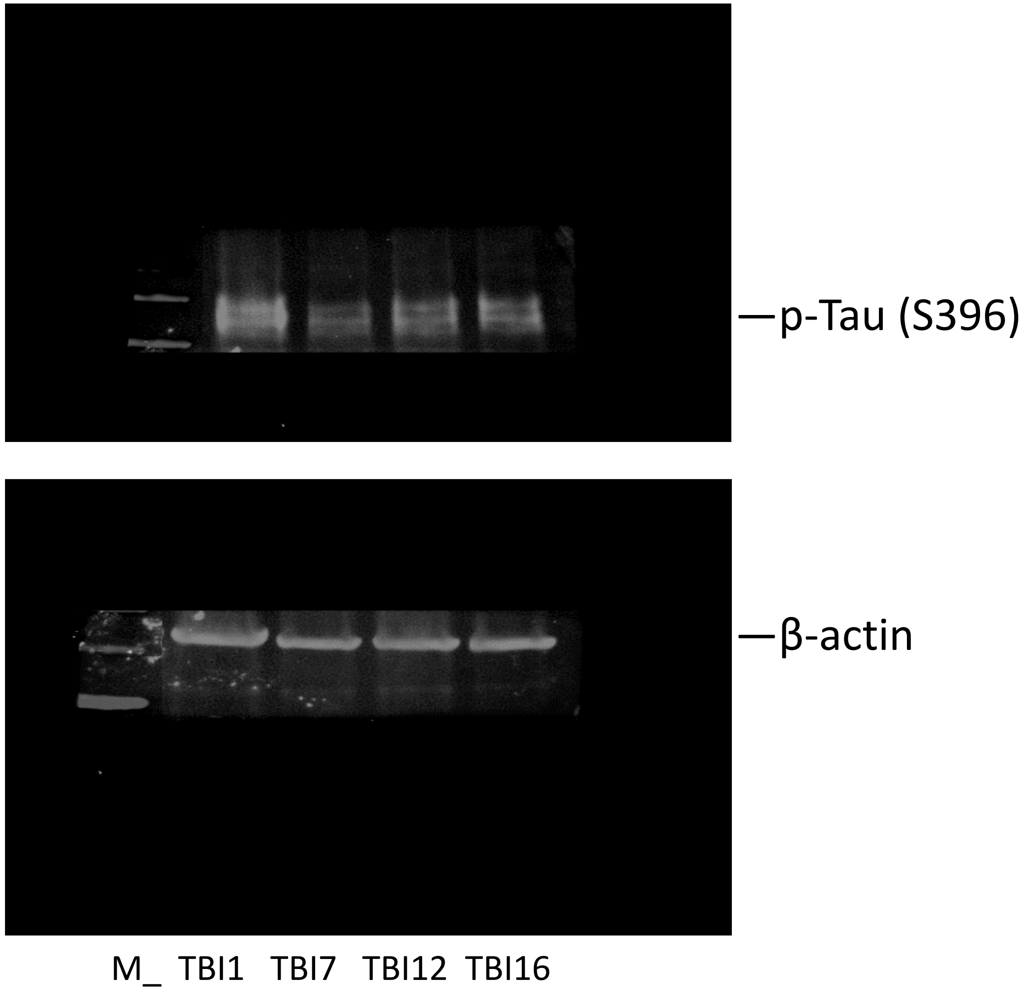

p-Tau (S396)_hippocampus_14d

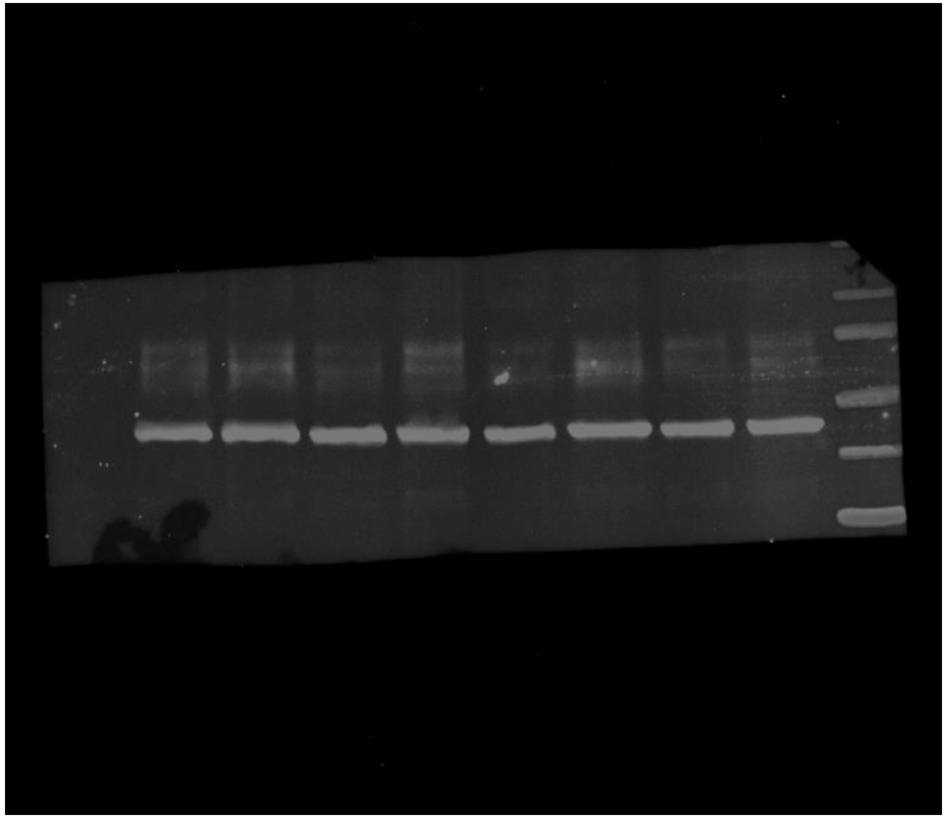

p-Tau (S396)_striatum_14d

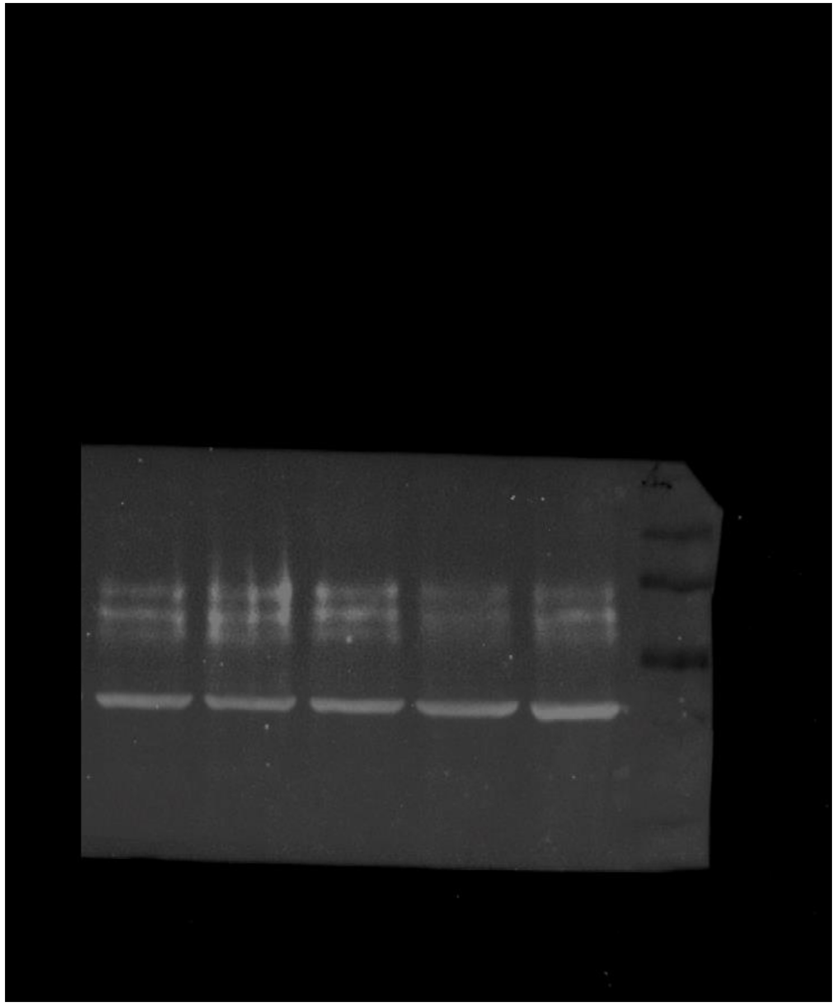

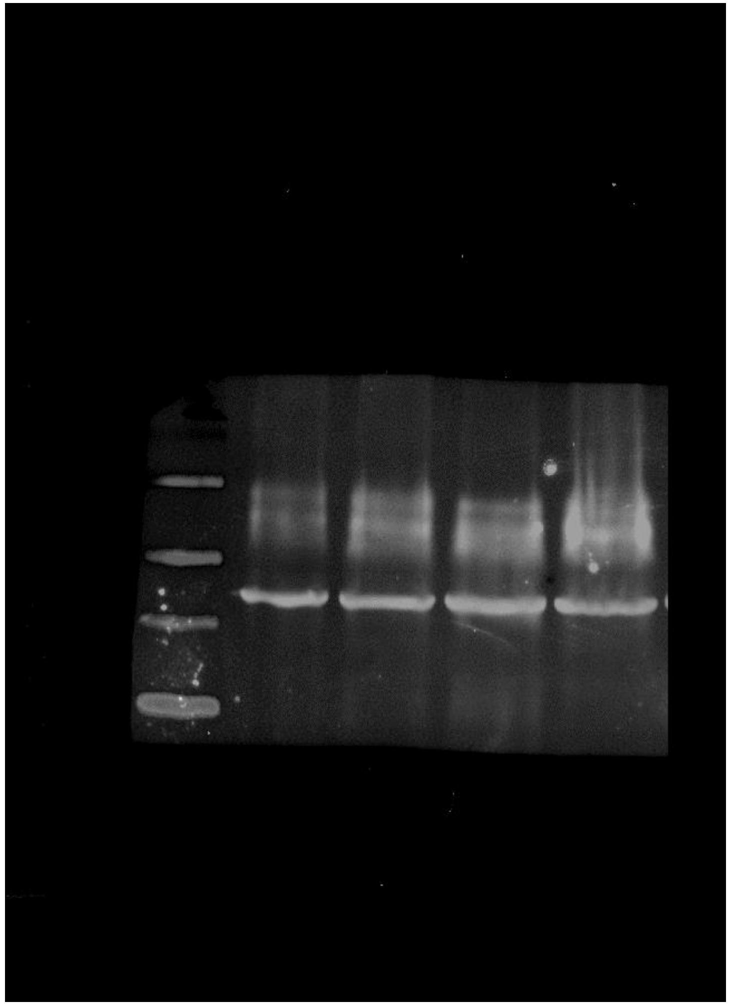

p-Tau (Thr205)_cortex_14d

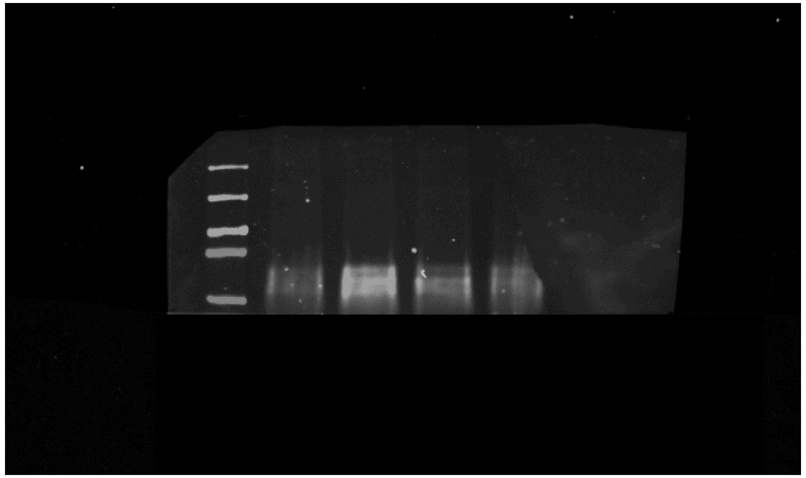

—p-Tau (Thr205)

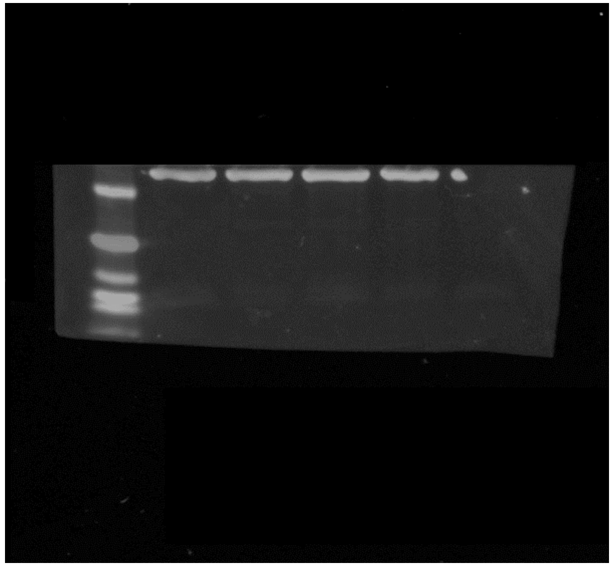

—β-actin

M_EV4 EV9 EV11 EV14

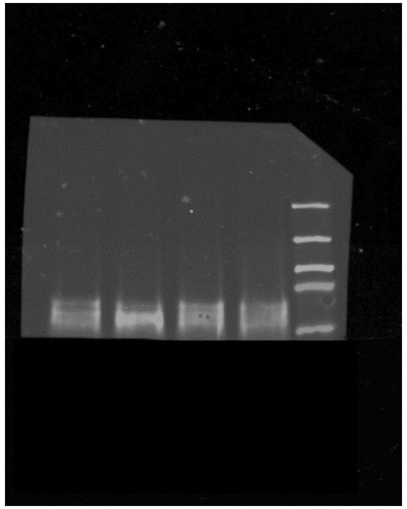

—p-Tau (Thr205)

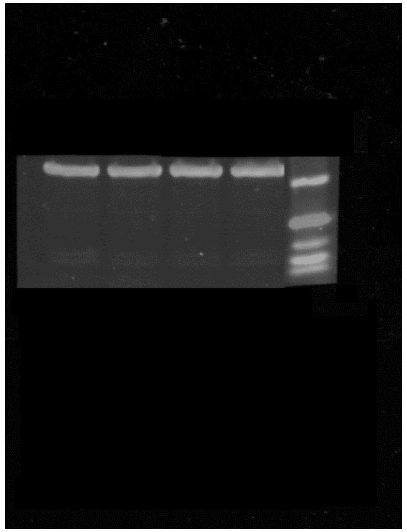

—β-actin

TBI16 TBI12 TBI7 TBI1_M

p-Tau (Thr205)_hippocampus_14d

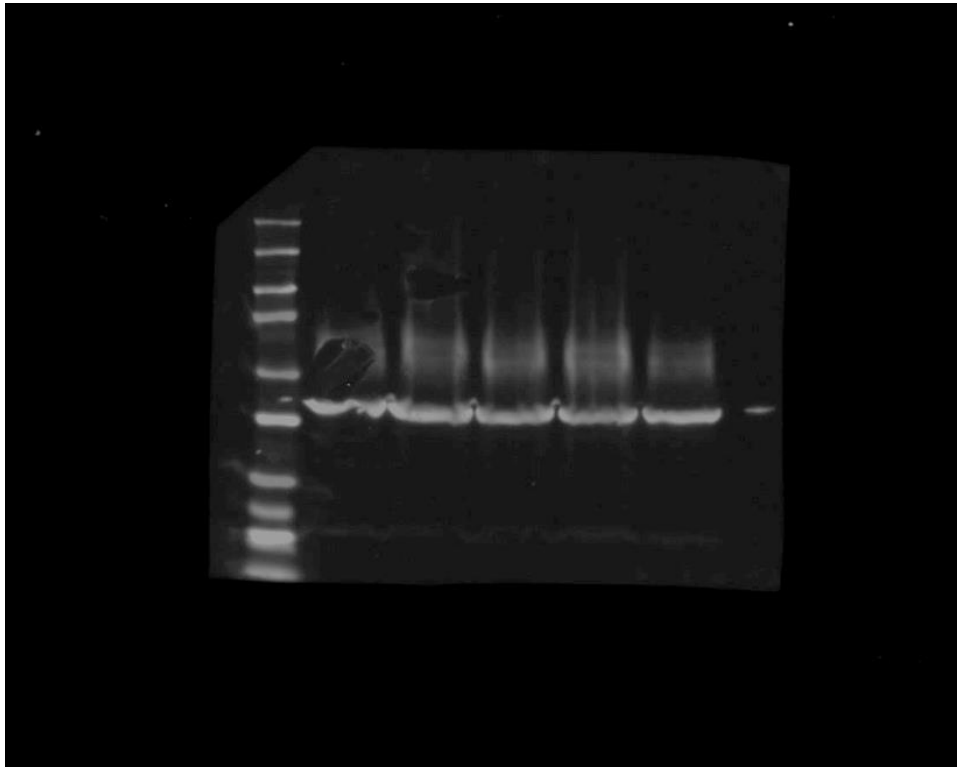

—p-Tau (Thr205)
—β-actin

M_EV4 EV9 EV11 EV14 EV17

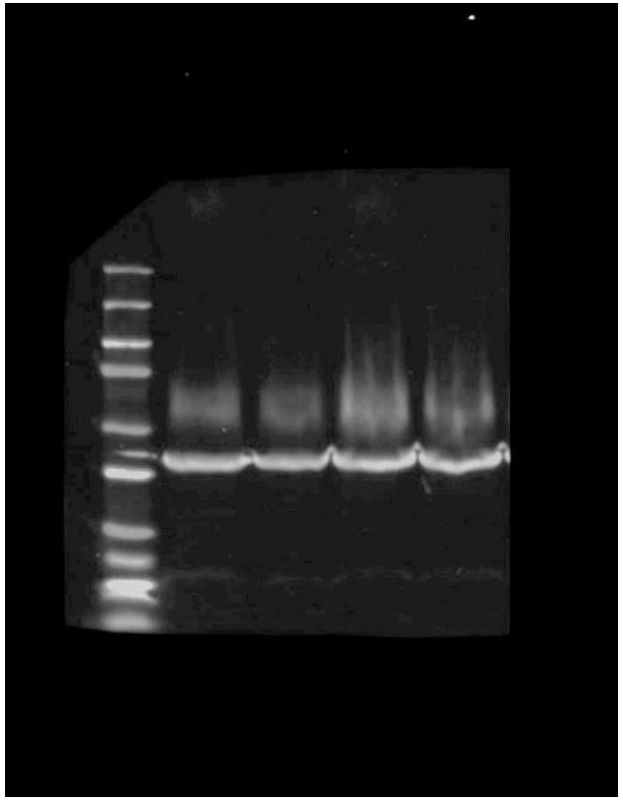

—p-Tau (Thr205)
—β-actin

M_TBI1 TBI7 TBI12 TBI16

p-Tau (Thr205)_striatum_14d

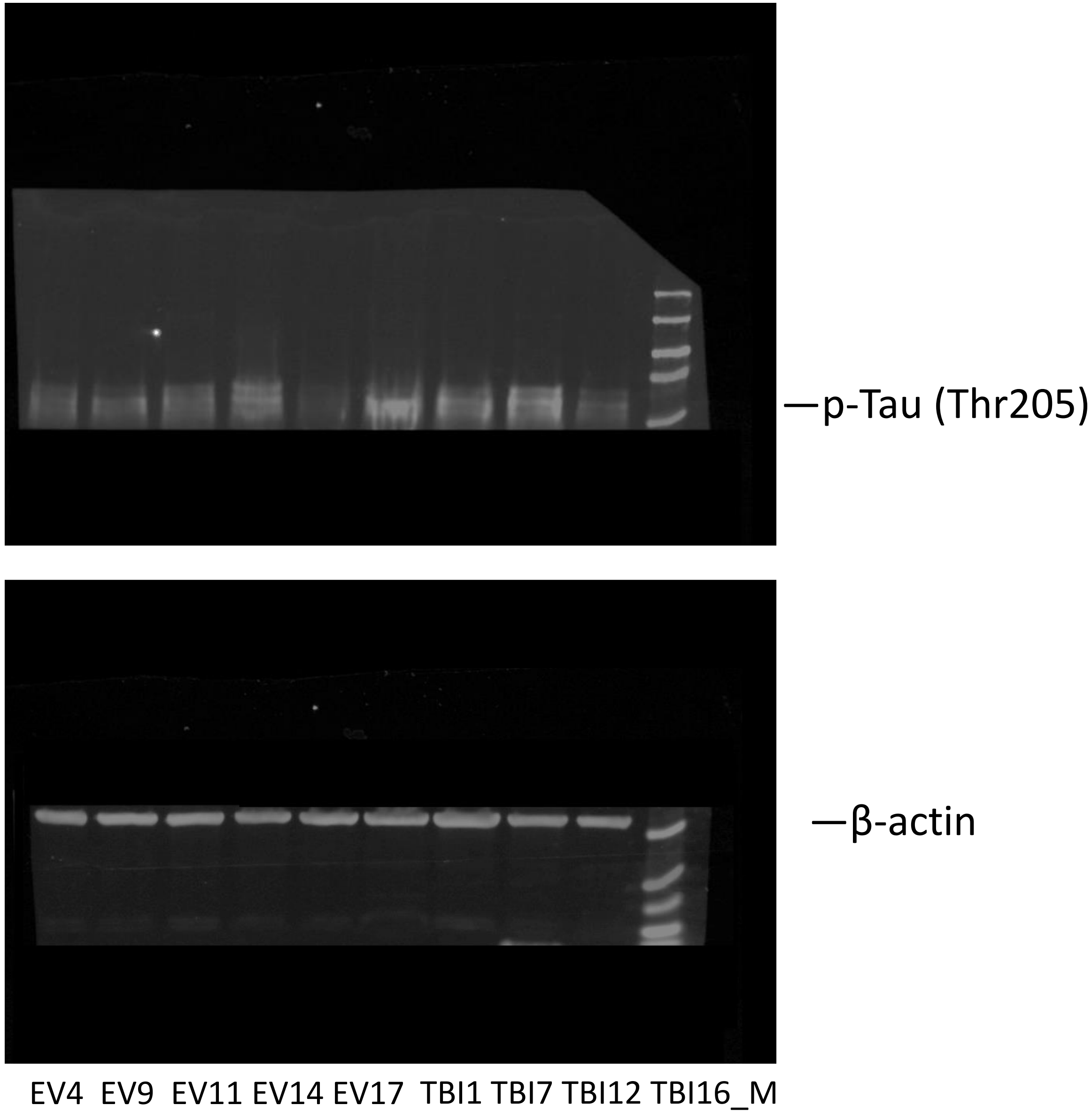

Tau_cortex_14d

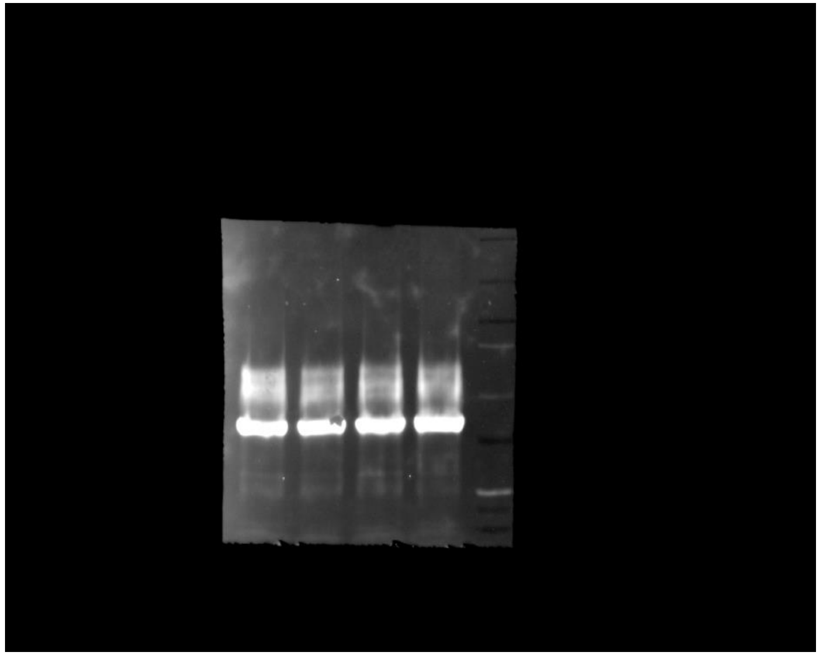

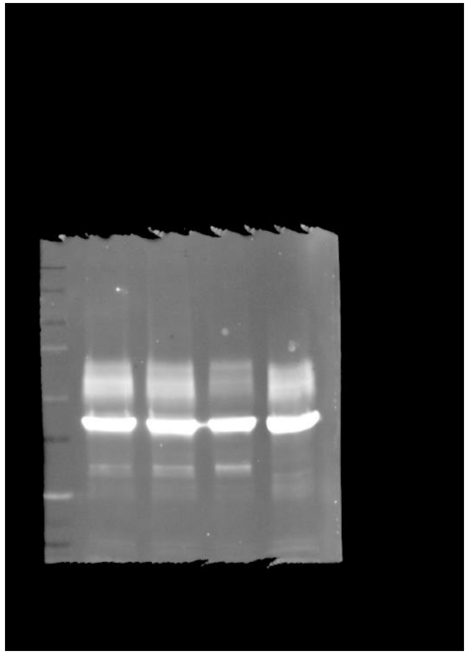

Tau_hippocampus_14d

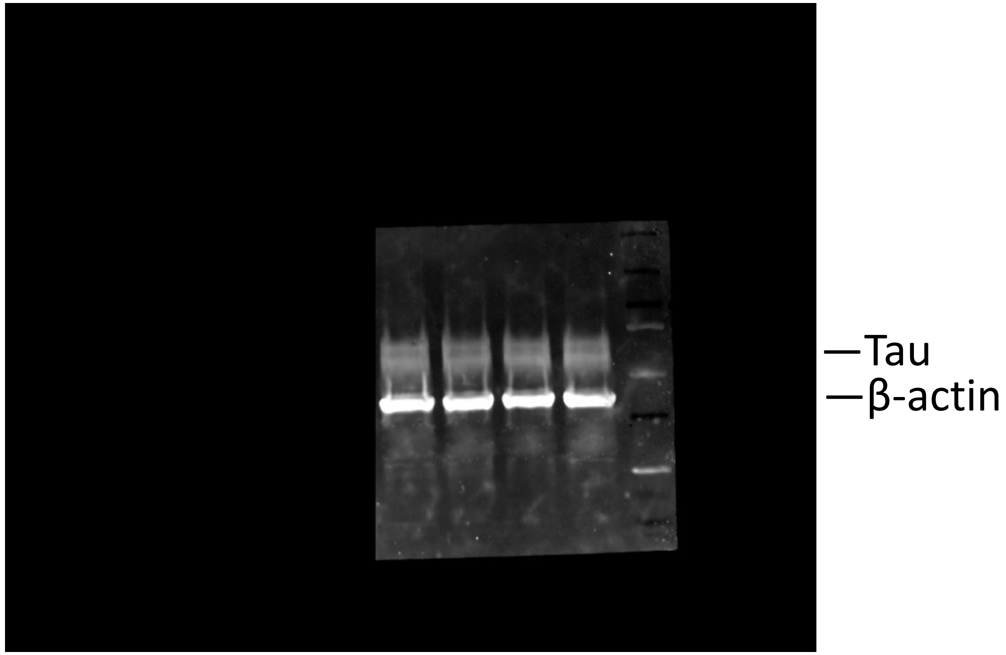

EV4 EV9 EV11 EV17_M

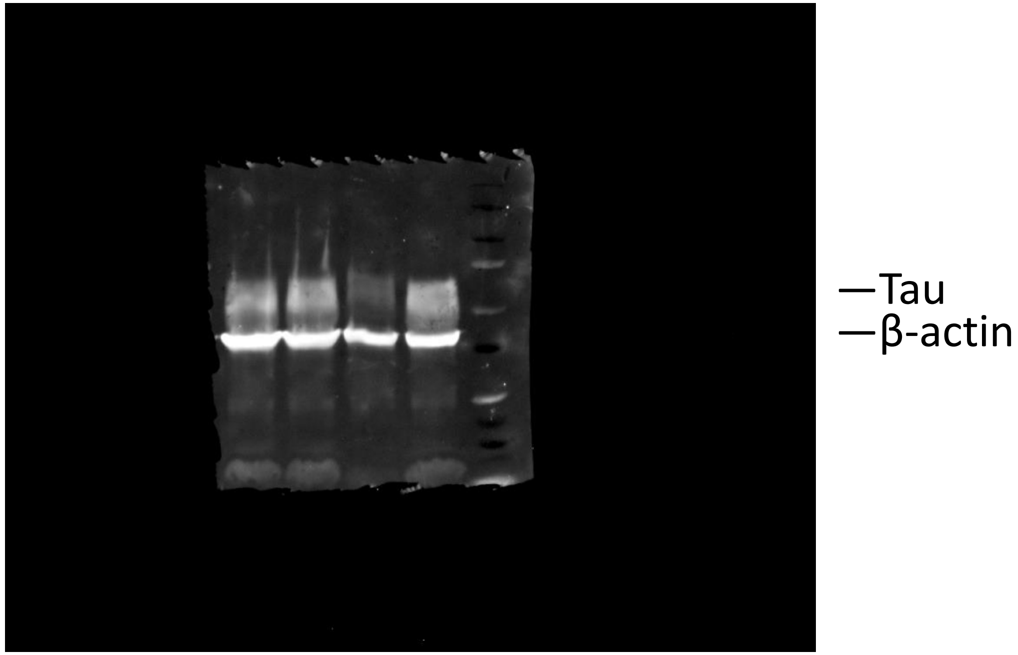

TBI16 TBI12 TBI7 TBI1_M

Tau_striatum_14d

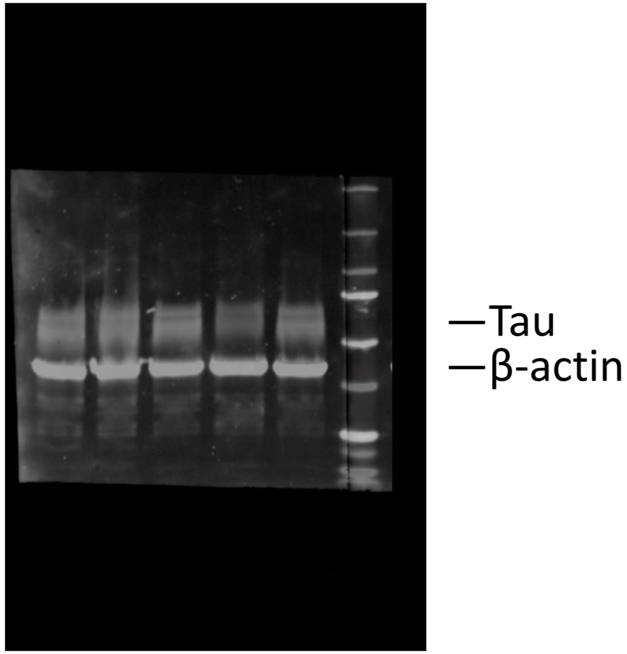

EV4 EV9 EV11 EV14 EV17_M

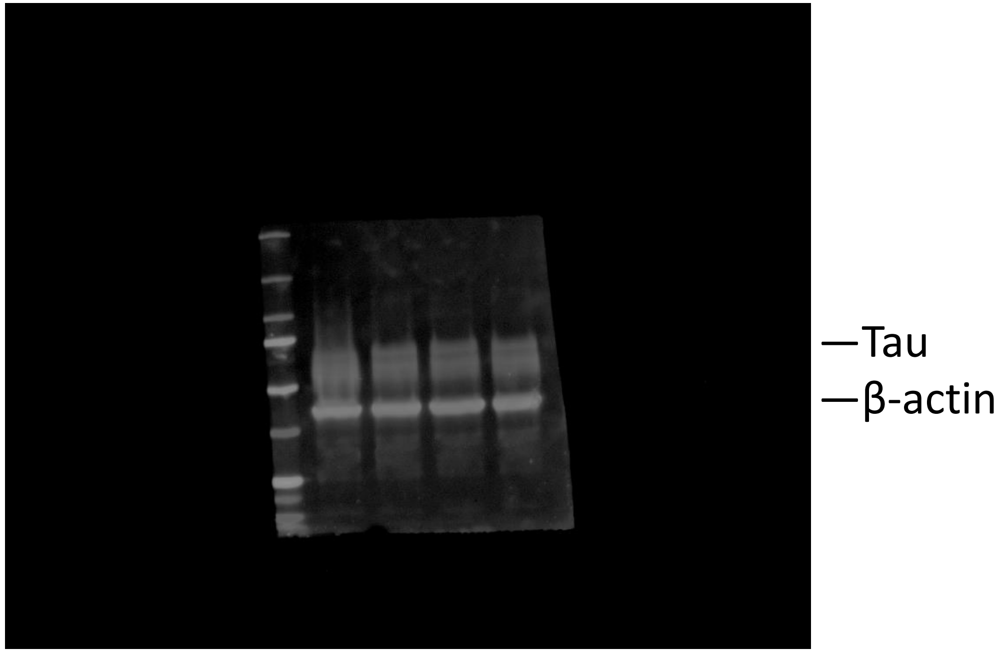

M_TBI16 TBI12 TBI7 TBI1
