# Supplementary material for: Extracellular vesicles of human glial cells exert neuroprotective effects via brain miRNA modulation in a rat model of traumatic brain injury
Source: Sci Rep. 2023 Nov 21;13:20388. doi: 10.1038/s41598-023-47627-2 (PMC10663567; doi:10.1038/s41598-023-47627-2)
Supplement: Supplementary file 2 — Supplementary Information 2. [file 41598_2023_47627_MOESM2_ESM.docx]

Supplementary Material

Table S1. Primer sequences used for RT-PCR for analysis gene expression in rat’s brain tissue.

| Gene | Primer sequences | Annealing temperature, °С |
| --- | --- | --- |
| Mmp2 | for CAGACAAAGAGTTGGCAGTG  rev TCAGGTGTGTAACCAATGATC | 56.5 |
| Mmp9 | for ATGGTTTCTGCCCCAGTGAG  rev CACCAGCGATAACCATCCGA | 63 |
| Ttnfa | for CCACCACGCTCTTCTGTCTA  rev GCTACGGGCTTGTCACTCG | 61.9 |
| Gapdh | for GAGATTACTGCCCTGGCTCC  rev GCTCAGTAACAGTCCGCCTA | 56.65 |
| Actb | for GCGAGATCCCGCTAACATCA  rev CCCTTCCACGATGCCAAAGT | 56 |
| Il12a | for CTGCCAAGTGTCTTAACCAGT  rev GCAGGCCTCCAGTGTGCT | 60.5 |
| Il12b | for CTGGTGTCTCCACTCATGG  rev CAGGTGTATTGGCCAGCATC | 60.5 |
| Il1b | for CTGTCTGACCCATGTGAGCT  rev ACTCCACTTTGGTCTTGACTT | 57.4 |
| Il6 | for TACATATGTTCTCAGGGAGAT  rev GGTAGAAACGGAACTCCAG | 56 |
| Il10 | for GCCCAGAAATCAAGGAGCAT  rev TGAGTGTCACGTAGGCTTCTA | 58.8 |
| Timp1 | for CAGACAGCTTTCTGCAACTC  rev CACAGCGTCGAATCCTTTGA | 58.8 |
| Timp2 | for ATGCAGACGTAGTGATCAGG  rev AGTCACAGAGGGTAATGTGC | 56 |
| Bax | for TTGTGGCTGGAGTCCTCACT  rev TTTCCCCGTTCCCCATTCATC | 63 |
| Bcl2 | for GGGGCTACGAGTGGGATACT  rev GACGGTAGCGACGAGAGAAG | 62.6 |

Table S2. Read numbers for micro RNA in GPC-EV isolates obtained from three independent donors (NGS data).

| miRNA | Culture 1 | Culture 2 | Culture 3 | average | stdev | scatter coefficient |
| --- | --- | --- | --- | --- | --- | --- |
| hsa-miR-425-3p | 80 | 77 | 80 | 79 | 2 | 0,02417743 |
| hsa-miR-135a-5p | 86 | 77 | 86 | 83 | 5 | 0,06467414 |
| hsa-miR-381-3p | 375 | 326 | 364 | 355 | 26 | 0,07256807 |
| hsa-miR-126-3p | 2543 | 2199 | 2403 | 2382 | 173 | 0,07267971 |
| hsa-miR-221-3p | 1592 | 1655 | 1840 | 1696 | 129 | 0,07590702 |
| hsa-miR-107 | 149 | 153 | 132 | 145 | 11 | 0,07870624 |
| hsa-miR-148a-3p | 38777 | 33728 | 39188 | 37231 | 3040 | 0,08166299 |
| hsa-miR-200a-5p | 177 | 192 | 210 | 193 | 16 | 0,08547223 |
| hsa-miR-221-5p | 65 | 77 | 66 | 69 | 7 | 0,09563451 |
| hsa-miR-503-5p | 108 | 134 | 124 | 122 | 13 | 0,10904798 |
| hsa-miR-140-5p | 116 | 128 | 145 | 130 | 14 | 0,11057372 |
| hsa-miR-30e-5p | 1094 | 1400 | 1393 | 1295 | 175 | 0,13493624 |
| hsa-miR-769-5p | 121 | 109 | 142 | 124 | 17 | 0,13501157 |
| hsa-miR-148a-5p | 99 | 89 | 71 | 87 | 14 | 0,16289009 |
| hsa-miR-455-5p | 1723 | 1413 | 1244 | 1460 | 243 | 0,16643076 |
| hsa-miR-495-3p | 99 | 70 | 83 | 84 | 14 | 0,17161605 |
| hsa-miR-95-3p | 112 | 134 | 159 | 135 | 24 | 0,17426025 |
| hsa-miR-200c-3p | 9622 | 13710 | 12009 | 11780 | 2054 | 0,17434287 |
| hsa-miR-339-3p | 86 | 83 | 113 | 94 | 16 | 0,17518563 |
| hsa-miR-199a-5p | 9602 | 7286 | 7065 | 7985 | 1405 | 0,17599713 |
| hsa-miR-425-5p | 302 | 320 | 418 | 347 | 63 | 0,18111016 |
| hsa-miR-194-5p | 170 | 128 | 186 | 161 | 30 | 0,18567717 |
| hsa-miR-24-3p | 5920 | 5804 | 8037 | 6587 | 1257 | 0,19084343 |
| hsa-miR-1296-5p | 65 | 45 | 51 | 53 | 10 | 0,19264986 |
| hsa-let-7e-5p | 878 | 1291 | 1219 | 1129 | 221 | 0,19548575 |
| hsa-miR-181a-5p | 3554 | 5343 | 4947 | 4615 | 939 | 0,20358038 |
| hsa-miR-340-5p | 106 | 121 | 157 | 128 | 26 | 0,20548975 |
| hsa-miR-186-5p | 766 | 1029 | 685 | 827 | 180 | 0,21750525 |
| hsa-miR-505-3p | 65 | 58 | 87 | 70 | 15 | 0,21871576 |
| hsa-miR-450b-5p | 114 | 141 | 90 | 115 | 25 | 0,21989079 |
| hsa-let-7a-5p | 151563 | 99370 | 108195 | 119709 | 27937 | 0,23337362 |
| hsa-miR-24-2-5p | 84 | 83 | 123 | 97 | 23 | 0,23521367 |
| hsa-miR-101-3p | 1736 | 2352 | 1472 | 1853 | 452 | 0,24375247 |
| hsa-miR-100-5p | 9399 | 5682 | 7569 | 7550 | 1859 | 0,24617943 |
| hsa-let-7b-3p | 69 | 96 | 60 | 75 | 19 | 0,2463471 |
| hsa-miR-106b-3p | 638 | 390 | 604 | 544 | 135 | 0,24749595 |
| hsa-miR-182-5p | 794 | 524 | 872 | 730 | 183 | 0,25002641 |
| hsa-miR-10a-5p | 26043 | 16618 | 27608 | 23423 | 5945 | 0,25380853 |
| hsa-miR-374a-5p | 136 | 96 | 164 | 132 | 34 | 0,25847684 |
| hsa-miR-134-5p | 99 | 58 | 89 | 82 | 22 | 0,26523837 |
| hsa-let-7d-3p | 179 | 115 | 202 | 165 | 45 | 0,27263362 |
| hsa-miR-379-5p | 349 | 262 | 201 | 271 | 75 | 0,27546191 |
| hsa-let-7f-5p | 60087 | 72838 | 40133 | 57686 | 16484 | 0,28575677 |
| hsa-miR-92a-3p | 5651 | 10048 | 7407 | 7702 | 2213 | 0,2873616 |
| hsa-miR-429 | 632 | 984 | 602 | 739 | 213 | 0,28762328 |
| hsa-miR-26a-5p | 167545 | 93713 | 124702 | 128653 | 37074 | 0,2881719 |
| hsa-miR-98-5p | 1122 | 818 | 1478 | 1139 | 330 | 0,29001798 |
| hsa-miR-29a-3p | 1014 | 1905 | 1635 | 1518 | 457 | 0,30104123 |
| hsa-miR-196b-5p | 2209 | 1297 | 2452 | 1986 | 609 | 0,30648678 |
| hsa-miR-145-3p | 239 | 128 | 225 | 197 | 61 | 0,30750559 |
| hsa-miR-127-3p | 1941 | 1182 | 2257 | 1793 | 552 | 0,30791035 |
| hsa-miR-493-3p | 283 | 153 | 200 | 212 | 65 | 0,30863037 |
| hsa-miR-501-3p | 132 | 173 | 91 | 132 | 41 | 0,30895634 |
| hsa-miR-34c-3p | 142 | 179 | 93 | 138 | 43 | 0,3107733 |
| hsa-miR-200b-3p | 10911 | 18497 | 11169 | 13526 | 4307 | 0,31844254 |
| hsa-miR-191-5p | 5746 | 3924 | 7716 | 5795 | 1896 | 0,32722349 |
| hsa-miR-10b-5p | 22392 | 11952 | 14427 | 16257 | 5455 | 0,33556441 |
| hsa-miR-152-3p | 1374 | 2039 | 1050 | 1488 | 504 | 0,33894115 |
| hsa-miR-132-3p | 175 | 211 | 331 | 239 | 82 | 0,34168834 |
| hsa-miR-195-3p | 80 | 38 | 71 | 63 | 22 | 0,34680758 |
| hsa-miR-187-3p | 123 | 211 | 116 | 150 | 53 | 0,35166353 |
| hsa-miR-25-3p | 1074 | 505 | 868 | 816 | 288 | 0,35329504 |
| hsa-miR-1271-5p | 69 | 32 | 58 | 53 | 19 | 0,35917972 |
| hsa-miR-27b-5p | 97 | 51 | 58 | 69 | 25 | 0,3593994 |
| hsa-miR-185-5p | 145 | 211 | 102 | 153 | 55 | 0,3595137 |
| hsa-miR-493-5p | 352 | 217 | 179 | 249 | 91 | 0,36346313 |
| hsa-miR-149-5p | 157 | 134 | 262 | 185 | 68 | 0,36996781 |
| hsa-let-7a-3p | 280 | 128 | 209 | 206 | 76 | 0,37116677 |
| hsa-miR-135b-5p | 86 | 58 | 124 | 89 | 33 | 0,37393213 |
| hsa-miR-183-5p | 546 | 256 | 567 | 456 | 174 | 0,38118444 |
| hsa-miR-22-3p | 1497 | 748 | 1778 | 1341 | 533 | 0,39714278 |
| hsa-miR-30c-5p | 4145 | 7580 | 3827 | 5184 | 2081 | 0,40141424 |
| hsa-miR-151a-3p | 5800 | 2640 | 6403 | 4947 | 2021 | 0,40852307 |
| hsa-miR-450a-5p | 992 | 422 | 679 | 698 | 286 | 0,40944027 |
| hsa-miR-484 | 155 | 109 | 246 | 170 | 70 | 0,41088633 |
| hsa-miR-27a-3p | 4361 | 4014 | 8185 | 5520 | 2315 | 0,41930958 |
| hsa-miR-193b-3p | 86 | 134 | 56 | 92 | 39 | 0,42789164 |
| hsa-miR-125a-5p | 2200 | 1649 | 3749 | 2533 | 1089 | 0,42997421 |
| hsa-miR-181b-5p | 804 | 1668 | 871 | 1115 | 481 | 0,43124652 |
| hsa-miR-210-3p | 78 | 160 | 204 | 147 | 64 | 0,43631255 |
| hsa-miR-125b-5p | 7158 | 4583 | 11328 | 7690 | 3404 | 0,44267101 |
| hsa-miR-99a-5p | 41603 | 15461 | 36550 | 31205 | 13866 | 0,44436564 |
| hsa-miR-320a | 3255 | 1406 | 3796 | 2819 | 1253 | 0,44448745 |
| hsa-miR-345-5p | 263 | 575 | 304 | 381 | 170 | 0,44526913 |
| hsa-miR-30d-5p | 15717 | 39347 | 24891 | 26651 | 11913 | 0,44699191 |
| hsa-miR-382-5p | 108 | 51 | 139 | 99 | 45 | 0,44993769 |
| hsa-miR-21-5p | 39304 | 103646 | 70915 | 71288 | 32173 | 0,45130612 |
| hsa-miR-141-3p | 123 | 249 | 115 | 163 | 75 | 0,46296336 |
| hsa-miR-26b-5p | 2424 | 5541 | 2829 | 3598 | 1695 | 0,47106013 |
| hsa-miR-339-5p | 97 | 153 | 57 | 103 | 48 | 0,47186915 |
| hsa-miR-30e-3p | 229 | 134 | 362 | 242 | 115 | 0,47430065 |
| hsa-miR-200a-3p | 2771 | 6935 | 3854 | 4520 | 2160 | 0,47791034 |
| hsa-miR-148b-3p | 507 | 1400 | 899 | 935 | 448 | 0,47846262 |
| hsa-miR-342-3p | 280 | 428 | 731 | 480 | 230 | 0,47902213 |
| hsa-miR-10b-3p | 142 | 64 | 68 | 91 | 44 | 0,48252247 |
| hsa-let-7c-5p | 15361 | 5139 | 14187 | 11562 | 5594 | 0,48379144 |
| hsa-miR-125b-1-3p | 214 | 70 | 214 | 166 | 83 | 0,49922668 |
| hsa-let-7d-5p | 3078 | 1227 | 3923 | 2743 | 1379 | 0,50271925 |
| hsa-miR-411-5p | 155 | 288 | 104 | 182 | 95 | 0,51863326 |
| hsa-miR-28-5p | 151 | 89 | 261 | 167 | 87 | 0,52069681 |
| hsa-miR-532-5p | 3037 | 984 | 1880 | 1967 | 1029 | 0,52310499 |
| hsa-miR-143-3p | 20431 | 34655 | 11410 | 22165 | 11719 | 0,52872086 |
| hsa-miR-409-3p | 324 | 96 | 320 | 246 | 130 | 0,52901921 |
| hsa-miR-378a-3p | 690 | 447 | 1303 | 813 | 441 | 0,54177688 |
| hsa-miR-192-5p | 509 | 211 | 741 | 487 | 266 | 0,54585544 |
| hsa-miR-28-3p | 468 | 230 | 764 | 488 | 268 | 0,54907223 |
| hsa-miR-494-3p | 116 | 179 | 52 | 116 | 64 | 0,55044205 |
| hsa-miR-29b-3p | 52 | 179 | 113 | 115 | 64 | 0,5550161 |
| hsa-miR-30a-3p | 436 | 243 | 783 | 487 | 274 | 0,56187531 |
| hsa-miR-30a-5p | 3848 | 10489 | 4770 | 6369 | 3598 | 0,56488984 |
| hsa-let-7b-5p | 79742 | 19967 | 71907 | 57205 | 32486 | 0,56788609 |
| hsa-miR-34c-5p | 1773 | 4391 | 6644 | 4269 | 2438 | 0,57104135 |
| hsa-miR-218-5p | 1057 | 256 | 905 | 739 | 426 | 0,57571983 |
| hsa-miR-320b | 526 | 153 | 650 | 443 | 259 | 0,5833211 |
| hsa-miR-330-3p | 99 | 38 | 152 | 96 | 57 | 0,58789681 |
| hsa-miR-99b-5p | 4434 | 1726 | 6897 | 4352 | 2587 | 0,59429872 |
| hsa-miR-16-5p | 1027 | 2691 | 1042 | 1587 | 956 | 0,60278384 |
| hsa-miR-424-3p | 815 | 173 | 697 | 562 | 342 | 0,60908166 |
| hsa-miR-370-3p | 380 | 83 | 379 | 281 | 171 | 0,60951595 |
| hsa-let-7i-5p | 60087 | 25138 | 102430 | 62552 | 38705 | 0,61876902 |
| hsa-miR-22-5p | 179 | 38 | 192 | 137 | 85 | 0,62461889 |
| hsa-miR-199b-5p | 3229 | 9549 | 3697 | 5491 | 3522 | 0,64130245 |
| hsa-miR-574-3p | 93 | 256 | 92 | 147 | 94 | 0,64133873 |
| hsa-miR-485-5p | 63 | 13 | 71 | 49 | 32 | 0,64601117 |
| hsa-miR-363-3p | 481 | 217 | 901 | 533 | 345 | 0,64655002 |
| hsa-miR-455-3p | 287 | 607 | 161 | 352 | 230 | 0,65335785 |
| hsa-miR-654-3p | 283 | 58 | 175 | 172 | 113 | 0,65597142 |
| hsa-miR-140-3p | 490 | 1496 | 562 | 849 | 561 | 0,66066868 |
| hsa-miR-432-5p | 192 | 32 | 178 | 134 | 89 | 0,66147775 |
| hsa-miR-99b-3p | 218 | 45 | 268 | 177 | 117 | 0,66227731 |
| hsa-miR-652-3p | 84 | 217 | 68 | 123 | 82 | 0,66508181 |
| hsa-miR-34b-3p | 145 | 83 | 318 | 182 | 122 | 0,67079349 |
| hsa-miR-375 | 147 | 741 | 920 | 603 | 405 | 0,67191288 |
| hsa-miR-146b-5p | 11399 | 3132 | 17483 | 10671 | 7203 | 0,67501036 |
| hsa-miR-181a-2-3p | 242 | 38 | 249 | 176 | 120 | 0,67811101 |
| hsa-miR-222-3p | 2875 | 626 | 3893 | 2465 | 1672 | 0,67817317 |
| hsa-let-7g-5p | 14239 | 49062 | 19426 | 27576 | 18787 | 0,68130458 |
| hsa-miR-125b-2-3p | 73 | 179 | 49 | 101 | 69 | 0,68541717 |
| hsa-miR-340-3p | 80 | 13 | 89 | 61 | 42 | 0,68726135 |
| hsa-miR-193a-5p | 93 | 45 | 194 | 111 | 76 | 0,69071573 |
| hsa-miR-654-5p | 86 | 13 | 91 | 63 | 44 | 0,69249164 |
| hsa-miR-361-5p | 127 | 422 | 152 | 234 | 164 | 0,70013963 |
| hsa-miR-130b-5p | 26 | 19 | 68 | 38 | 27 | 0,70311862 |
| hsa-miR-150-5p | 136 | 121 | 399 | 219 | 156 | 0,71368112 |
| hsa-miR-92b-3p | 865 | 371 | 1812 | 1016 | 732 | 0,72100532 |
| hsa-miR-106b-5p | 56 | 166 | 49 | 91 | 66 | 0,72410154 |
| hsa-miR-93-5p | 196 | 594 | 178 | 323 | 235 | 0,72892896 |
| hsa-miR-629-5p | 125 | 64 | 289 | 159 | 116 | 0,73010703 |
| hsa-miR-19b-3p | 106 | 345 | 111 | 187 | 137 | 0,73038892 |
| hsa-miR-320c | 145 | 26 | 205 | 125 | 91 | 0,73075473 |
| hsa-miR-224-5p | 423 | 45 | 360 | 276 | 203 | 0,73437738 |
| hsa-miR-941 | 1693 | 1023 | 4355 | 2357 | 1762 | 0,74776557 |
| hsa-miR-31-5p | 58 | 173 | 47 | 93 | 69 | 0,74894708 |
| hsa-miR-10a-3p | 84 | 294 | 93 | 157 | 119 | 0,7546968 |
| hsa-miR-1307-3p | 561 | 179 | 1108 | 616 | 467 | 0,7582293 |
| hsa-miR-7-5p | 2618 | 1777 | 7373 | 3923 | 3017 | 0,76919251 |
| hsa-miR-16-2-3p | 593 | 51 | 412 | 352 | 276 | 0,78373624 |
| hsa-miR-543 | 336 | 32 | 480 | 283 | 229 | 0,80895833 |
| hsa-miR-328-3p | 134 | 77 | 373 | 195 | 157 | 0,80903357 |
| hsa-miR-433-3p | 67 | 19 | 142 | 76 | 62 | 0,81351124 |
| hsa-miR-483-5p | 114 | 6 | 83 | 68 | 56 | 0,81675702 |
| hsa-miR-197-3p | 73 | 262 | 67 | 134 | 111 | 0,82634281 |
| hsa-miR-361-3p | 746 | 300 | 1869 | 972 | 808 | 0,83171552 |
| hsa-miR-143-5p | 71 | 217 | 44 | 111 | 93 | 0,84145862 |
| hsa-miR-449a | 235 | 192 | 824 | 417 | 353 | 0,84685158 |
| hsa-miR-200b-5p | 147 | 32 | 305 | 161 | 137 | 0,85093205 |
| hsa-miR-199a-3p | 20205 | 76488 | 18367 | 38353 | 33038 | 0,86141986 |
| hsa-miR-199b-3p | 10094 | 38241 | 9180 | 19172 | 16521 | 0,86172566 |
| hsa-miR-9-5p | 356 | 1649 | 461 | 822 | 718 | 0,87356069 |
| hsa-miR-223-5p | 88 | 13 | 174 | 92 | 80 | 0,8781128 |
| hsa-miR-17-5p | 123 | 524 | 131 | 259 | 229 | 0,88491458 |
| hsa-miR-128-3p | 1954 | 300 | 4016 | 2090 | 1862 | 0,89064883 |
| hsa-miR-27b-3p | 10698 | 43833 | 10213 | 21581 | 19272 | 0,89301803 |
| hsa-miR-205-5p | 65 | 32 | 200 | 99 | 89 | 0,90053825 |
| hsa-miR-505-5p | 69 | 13 | 156 | 79 | 72 | 0,91015494 |
| hsa-miR-502-3p | 71 | 236 | 40 | 116 | 106 | 0,91388175 |
| hsa-miR-223-3p | 127 | 6 | 62 | 65 | 61 | 0,93020116 |
| hsa-miR-142-5p | 824 | 96 | 1835 | 918 | 873 | 0,95118107 |
| hsa-miR-214-3p | 170 | 741 | 143 | 352 | 338 | 0,96135065 |
| hsa-miR-500a-3p | 101 | 352 | 49 | 167 | 162 | 0,96470159 |
| hsa-miR-744-5p | 761 | 268 | 2363 | 1131 | 1095 | 0,96840073 |
| hsa-miR-151a-5p | 47 | 339 | 97 | 161 | 156 | 0,96884277 |
| hsa-miR-660-5p | 39 | 211 | 46 | 99 | 97 | 0,9867994 |
| hsa-miR-449c-5p | 1037 | 224 | 2920 | 1394 | 1383 | 0,99235771 |
| hsa-miR-144-3p | 173 | 6 | 72 | 84 | 84 | 0,99816705 |
| hsa-miR-1180-3p | 434 | 70 | 1160 | 555 | 555 | 1,00033288 |
| hsa-miR-142-3p | 99 | 38 | 382 | 173 | 183 | 1,05908711 |
| hsa-miR-365a-3p | 26 | 192 | 40 | 86 | 92 | 1,07410166 |
| hsa-miR-365b-3p | 26 | 192 | 40 | 86 | 92 | 1,07410166 |
| hsa-miR-23b-3p | 2681 | 9728 | 659 | 4356 | 4761 | 1,09296455 |
| hsa-miR-146a-5p | 503 | 288 | 2437 | 1076 | 1184 | 1,10046692 |
| hsa-miR-451a | 14192 | 2991 | 1178 | 6120 | 7048 | 1,15162033 |
| hsa-miR-23a-3p | 1029 | 6053 | 601 | 2561 | 3032 | 1,18391531 |
| hsa-miR-195-5p | 93 | 767 | 97 | 319 | 388 | 1,21757702 |
| hsa-miR-424-5p | 41 | 371 | 51 | 154 | 188 | 1,21813118 |
| hsa-miR-1301-3p | 112 | 6 | 448 | 189 | 231 | 1,22103536 |
| hsa-miR-20a-5p | 91 | 972 | 134 | 399 | 497 | 1,2454389 |
| hsa-miR-92b-5p | 334 | 38 | 1547 | 640 | 800 | 1,24938896 |
| hsa-miR-342-5p | 235 | 6 | 1025 | 422 | 534 | 1,26587285 |
| hsa-miR-145-5p | 224 | 1540 | 100 | 622 | 798 | 1,28416041 |
| hsa-miR-2110 | 123 | 13 | 704 | 280 | 371 | 1,32679391 |
| hsa-miR-155-5p | 498 | 166 | 4426 | 1697 | 2369 | 1,39637568 |
| hsa-miR-30b-5p | 380 | 6641 | 269 | 2430 | 3647 | 1,50102923 |

Table S3. Reverse transcription PCR quantitation of microRNA in cerebral cortex of rats after intranasal administration of extracellular vesicles (EV) or sham infusions (control, C) on post-TBI day 7.

| miRNA | ∆Ct | | | | | | *t*-test |
| --- | --- | --- | --- | --- | --- | --- | --- |
|  | C5 TR | C7 TR | C13 TR | EV1 TR | EV12 TR | EV16 TR | p-value |
| miR-99a-3p | 8.588343 | 8.508963 | 7.460496 | 13.65556 | 13.97899 | 12.26709 | 0.000659 |
| miR-30a-5p | 1.018041 | 1.006621 | 1.034558 | 0.132421 | 0.438399 | 0.525792 | 0.002712 |
| miR-26a-5p | -0.65682 | -1.00019 | -0.98099 | -1.679 | -2.63815 | -2.1109 | 0.006701 |
| let-7i-3p | 8.524105 | 10.32283 | 8.470272 | 7.100896 | 6.562463 | 6.574438 | 0.010224 |
| miR-9a-5p | -0.23184 | -0.93591 | -1.99326 | -2.47464 | -3.25397 | -2.52197 | 0.020512 |
| miR-128-3p | 1.19738 | -0.62644 | -0.94006 | -1.94807 | -2.13398 | -1.9967 | 0.02329 |
| miR-212-3p | 4.881556 | 2.373144 | 3.059692 | 1.110964 | 1.123377 | 1.689601 | 0.025479 |
| miR-181b-5p | 0.662736 | -1.42201 | -1.15026 | -2.6913 | -2.18968 | -2.51411 | 0.026314 |
| miR-30a-3p | 3.064209 | 1.905923 | 1.326146 | 0.489945 | 0.648671 | 0.942029 | 0.028125 |
| miR-124-3p | -0.15033 | -3.51642 | -1.22085 | -3.71929 | -4.37567 | -5.22429 | 0.030276 |
| miR-125b-5p | -1.11123 | -2.7444 | -1.98335 | -3.75485 | -2.76117 | -3.82904 | 0.030934 |
| miR-27b-3p | 1.969828 | 2.250014 | 1.374188 | 0.880943 | 1.252877 | 1.286641 | 0.033166 |
| miR-29a-3p | 1.162883 | -0.74993 | 0.446983 | -0.76106 | -0.95486 | -1.42125 | 0.043676 |
| miR-221-3p | 1.222041 | 1.013423 | -0.12387 | -0.50245 | -0.46412 | 0.078103 | 0.047269 |
| miR-29c-3p | 0.174933 | -1.80416 | -0.90085 | -1.86798 | -2.19937 | -2.24207 | 0.048624 |
| miR-27a-5p | 6.931307 | 6.380024 | 4.505055 | 8.541685 | 12.02862 | 7.370023 | 0.049782 |
| miR-129-5p | 3.772012 | 1.693601 | 1.277868 | 0.370461 | 0.752407 | 0.72333 | 0.052435 |
| miR-23a-3p | -2.38265 | -2.3684 | -3.40699 | -3.46224 | -4.73942 | -3.36121 | 0.056597 |
| miR-22-5p | 6.312946 | 13.81513 | 8.881887 | 5.893895 | 4.62015 | 6.069189 | 0.069594 |
| let-7f-5p | 0.109873 | 1.025886 | -0.58666 | -0.47658 | -1.98714 | -0.43098 | 0.086344 |
| let-7c-5p | -2.64833 | -2.85282 | -3.50027 | -3.28739 | -4.31181 | -3.41578 | 0.08932 |
| miR-376b-5p | 7.616166 | 14.76237 | 5.512583 | 5.222338 | 3.719334 | 6.325343 | 0.110157 |
| miR-30c-5p | 1.686251 | 2.72381 | 1.856562 | 1.585886 | 0.978052 | 1.893217 | 0.111678 |
| miR-138-5p | 3.794895 | 2.421945 | 2.254713 | 2.473137 | 2.084993 | 1.997271 | 0.139067 |
| miR-29c-5p | 6.844469 | 5.709009 | 5.625171 | 6.011708 | 7.280041 | 6.822394 | 0.14928 |
| miR-22-3p | 4.23063 | 4.848526 | 4.81362 | 4.26527 | 3.15647 | 4.754339 | 0.163743 |
| miR-30b-3p | 12.77111 | 5.884822 | 6.705876 | 6.442921 | 4.268026 | 7.093791 | 0.170562 |
| miR-107-3p | 5.496224 | 13.04343 | 6.113382 | 8.049577 | 4.514483 | 4.058843 | 0.191058 |
| miR-223-3p | -1.69223 | 4.150282 | 0.46588 | 6.113038 | -0.80252 | 5.239879 | 0.20484 |
| miR-300-5p | 8.007431 | 14.76237 | 13.80779 | 13.65556 | 7.763703 | 7.129152 | 0.20864 |
| miR-200b-3p | 5.125211 | 12.95422 | 6.677212 | 13.65556 | 12.05425 | 7.221043 | 0.212955 |
| miR-186-5p | 6.118359 | 6.895609 | 5.398972 | 13.65556 | 6.114279 | 5.456531 | 0.221098 |
| let-7i-5p | 0.137405 | 1.501972 | 0.163484 | 0.47581 | -0.41436 | 0.571169 | 0.258338 |
| miR-30c-2-3p | 7.975718 | 5.563164 | 6.12274 | 13.65556 | 5.698694 | 5.666953 | 0.276068 |
| miR-92b-3p | 0.732026 | -0.97028 | -0.99813 | -1.47881 | 0.037827 | -1.14085 | 0.287086 |
| miR-99a-5p | 4.898629 | 5.339738 | 5.155735 | 4.078388 | 5.937844 | 4.419437 | 0.307202 |
| miR-21-5p | -0.9289 | 1.917334 | -0.143 | 1.966196 | -1.80898 | 2.697309 | 0.351453 |
| miR-30c-1-3p | 6.837058 | 14.76237 | 5.420626 | 13.65556 | 6.375892 | 10.16577 | 0.391253 |
| miR-330-5p | 14.57922 | 6.304322 | 13.80779 | 6.926781 | 13.97899 | 11.11833 | 0.401639 |
| miR-551b-3p | 8.28779 | 14.76237 | 6.862402 | 6.318221 | 6.843374 | 14.42033 | 0.419239 |
| miR-29b-3p | 7.178147 | 10.77499 | 5.45546 | 8.799688 | 4.262782 | 9.281893 | 0.440886 |
| miR-497-5p | 5.664344 | 7.77726 | 6.188023 | 5.778586 | 7.150102 | 6.425446 | 0.454146 |
| miR-29a-5p | 7.869595 | 9.964232 | 10.21402 | 7.49674 | 13.97899 | 6.462389 | 0.49444 |
| miR-27a-3p | 3.751132 | 3.923527 | 5.121201 | 5.290136 | 3.043151 | 4.450156 | 0.498021 |
| miR-24-3p | 0.172499 | 0.216515 | 0.157315 | 1.455006 | -0.78278 | -0.11826 | 0.498561 |

Table S4. Reverse transcription PCR quantitation of microRNA in hippocampus of rats after intranasal administration of extracellular vesicles (EV) or sham infusions (control, C) on post-TBI day 7.

| miRNA | ∆Ct | | | | | | *t*-test |
| --- | --- | --- | --- | --- | --- | --- | --- |
|  | C5 hipp | C7 hipp | C13 hipp | EV1 hipp | EV12 hipp | EV16 hipp | *p*-value |
| miR-124-3p | -1.44676 | -1.53531 | -1.18771 | -5.14492 | -3.72443 | -3.95475 | 0.001551 |
| miR-212-3p | 3.615617 | 4.062197 | 4.415586 | 2.33755 | 1.72715 | 2.602201 | 0.003244 |
| miR-221-3p | 0.753965 | 1.707339 | 0.938811 | -1.17085 | -0.76092 | -0.15477 | 0.005819 |
| miR-30a-3p | 2.918999 | 2.531223 | 1.890703 | 0.622296 | 1.330602 | 1.208848 | 0.009938 |
| miR-26a-5p | -1.12606 | 0.01556 | -0.24873 | -1.8499 | -1.42949 | -1.63691 | 0.01581 |
| miR-9a-5p | -0.52427 | -0.06411 | -1.05528 | -3.07108 | -2.05386 | -1.56342 | 0.016723 |
| miR-300-5p | 9.577188 | 12.88446 | 14.74694 | 7.447425 | 7.313924 | 7.916229 | 0.016751 |
| miR-107-3p | 6.617193 | 8.136854 | 6.492261 | 5.047551 | 5.367431 | 5.627734 | 0.017634 |
| miR-92b-3p | -0.36246 | -1.06514 | -0.08668 | -2.12707 | -1.47401 | -1.35497 | 0.019206 |
| miR-129-5p | 3.146249 | 2.505305 | 2.840155 | 0.7377 | 1.468584 | 2.168158 | 0.01934 |
| miR-181b-5p | -0.23393 | -1.21278 | -0.17366 | -1.83669 | -1.4009 | -1.64442 | 0.019518 |
| miR-128-3p | 0.212768 | 1.006757 | 0.904169 | -1.35315 | -0.14613 | -0.28951 | 0.022831 |
| miR-125b-5p | -2.91442 | -2.51853 | -2.95314 | -3.19561 | -3.24897 | -3.84151 | 0.031958 |
| let-7c-5p | -2.73055 | -2.64767 | -3.29733 | -4.27992 | -4.28836 | -3.21626 | 0.032489 |
| miR-29a-3p | -0.03799 | 0.160539 | 1.814341 | -0.60869 | -0.72401 | -0.68506 | 0.044245 |
| miR-22-3p | 4.10016 | 5.330758 | 4.716355 | 4.113632 | 3.765315 | 3.832953 | 0.046915 |
| miR-29c-3p | -1.08767 | -0.71454 | 1.011805 | -1.52122 | -2.0181 | -1.57169 | 0.048252 |
| let-7f-5p | 1.70052 | 1.885031 | 0.107042 | -1.15815 | -0.61677 | 0.642903 | 0.053578 |
| miR-24-3p | 1.484631 | 1.866675 | 0.952854 | 0.657553 | 1.086794 | 0.717679 | 0.053774 |
| miR-23a-3p | -1.37344 | -0.71305 | -1.84015 | -2.35052 | -2.72456 | -1.56733 | 0.063906 |
| miR-99a-5p | 5.378173 | 6.669793 | 4.961951 | 4.828951 | 5.166969 | 4.459988 | 0.099325 |
| miR-138-5p | 2.604695 | 3.33708 | 2.798988 | 1.746978 | 3.038175 | 1.843754 | 0.104058 |
| let-7i-5p | 0.950745 | 1.954 | 1.005977 | 0.112114 | 0.677996 | 1.149975 | 0.10603 |
| let-7i-3p | 9.945796 | 8.263776 | 6.954963 | 11.80207 | 14.24234 | 7.247896 | 0.145131 |
| miR-551b-3p | 11.76136 | 12.34895 | 10.20516 | 8.29074 | 12.51084 | 7.752198 | 0.152619 |
| miR-30a-5p | 1.127996 | 1.469264 | 2.147339 | 1.081952 | 1.113342 | 1.510753 | 0.176565 |
| miR-376b-5p | 8.063481 | 6.890639 | 6.043957 | 4.988894 | 5.013421 | 7.776691 | 0.191285 |
| miR-186-5p | 6.649059 | 7.885591 | 7.267268 | 6.540228 | 11.67548 | 7.948673 | 0.203785 |
| miR-200b-3p | 10.03495 | 8.156748 | 11.85015 | 7.217758 | 6.699481 | 11.22806 | 0.206069 |
| miR-223-3p | 3.174229 | 6.714502 | 4.298515 | 7.928019 | 3.870041 | 6.189817 | 0.232794 |
| miR-30c-2-3p | 7.04286 | 6.137851 | 7.340818 | 7.527672 | 5.916975 | 5.501888 | 0.252008 |
| miR-30b-3p | 9.385594 | 7.999095 | 5.812105 | 7.219555 | 5.324025 | 7.836956 | 0.252914 |
| miR-22-5p | 7.076607 | 7.025452 | 7.361814 | 5.721107 | 8.067849 | 6.095294 | 0.256792 |
| miR-330-5p | 9.281898 | 15.20334 | 10.06439 | 7.434544 | 14.24234 | 6.62192 | 0.26585 |
| miR-29a-5p | 9.214693 | 8.899092 | 13.09904 | 14.57901 | 6.749185 | 15.37054 | 0.29144 |
| miR-29c-5p | 6.61512 | 6.28395 | 6.913334 | 5.172869 | 14.24234 | 5.643311 | 0.29282 |
| miR-21-5p | 1.187451 | 4.017446 | 2.164003 | 4.573807 | 1.112089 | 4.102245 | 0.293235 |
| miR-30c-5p | 2.298383 | 3.335187 | 2.533066 | 2.362269 | 2.72441 | 2.559802 | 0.313983 |
| miR-497-5p | 6.438131 | 8.730541 | 6.393869 | 7.864038 | 7.090683 | 7.858693 | 0.317588 |
| miR-27b-3p | 2.826229 | 2.688444 | 2.108293 | 2.7482 | 2.377861 | 2.076741 | 0.329021 |
| miR-27a-5p | 7.287977 | 8.124839 | 10.75703 | 8.281589 | 11.56628 | 8.06509 | 0.362663 |
| miR-27a-3p | 4.172677 | 5.228263 | 5.526385 | 7.063927 | 4.165473 | 4.725079 | 0.371977 |
| miR-30c-1-3p | 8.030854 | 15.20334 | 7.608605 | 6.697233 | 14.24234 | 6.34701 | 0.37808 |
| miR-29b-3p | 6.730708 | 6.626819 | 6.273978 | 6.20558 | 5.497299 | 7.458917 | 0.401908 |
| miR-99a-3p | 11.3636 | 7.827534 | 6.246747 | 8.243853 | 6.820681 | 10.7754 | 0.473633 |

Table S5. Reverse transcription PCR quantitation of microRNA in striatum of rats after intranasal administration of extracellular vesicles (EV) or sham infusions (control, C) on post-TBI day 7.

| miRNA | ∆Ct | | | | *t*-test |
| --- | --- | --- | --- | --- | --- |
|  | C5 striatum | C7 striatum | EV1 striatum | EV12 striatum | *p*-value |
| miR-107-3p | 6.071651 | 6.985319 | 5.156954 | 4.189393 | 0.054077 |
| miR-212-3p | 3.448956 | 3.832851 | 1.471159 | 2.580587 | 0.055304 |
| miR-124-3p | -1.36517 | -1.73739 | -4.59343 | -2.40225 | 0.110968 |
| miR-129-5p | 1.200979 | 2.46036 | 0.600046 | 0.862764 | 0.114794 |
| let-7c-5p | -2.93673 | -1.95452 | -3.18294 | -3.28374 | 0.12582 |
| miR-376b-5p | 14.64711 | 14.04205 | 13.34802 | 9.896282 | 0.130235 |
| miR-200b-3p | 10.72299 | 14.04205 | 8.701282 | 10.39207 | 0.133643 |
| let-7f-5p | 0.647342 | 4.035011 | -0.6864 | 0.398197 | 0.14857 |
| miR-181b-5p | -1.35929 | -0.4005 | -1.54302 | -1.54364 | 0.150295 |
| miR-30b-3p | 7.405507 | 14.04205 | 7.731073 | 4.792773 | 0.171947 |
| miR-9a-5p | -0.58284 | 3.200417 | -1.46776 | -0.48648 | 0.181322 |
| miR-186-5p | 7.167678 | 13.70884 | 6.766075 | 6.457672 | 0.181498 |
| miR-30c-1-3p | 14.64711 | 6.670491 | 6.456753 | 5.606932 | 0.183953 |
| miR-221-3p | 0.292283 | 4.20044 | -0.69329 | 0.506633 | 0.18544 |
| miR-125b-5p | -2.09371 | -2.47277 | -2.3938 | -2.69802 | 0.196385 |
| miR-26a-5p | -0.72285 | 2.13928 | -1.10488 | -0.57408 | 0.199511 |
| miR-128-3p | 0.674959 | 5.129348 | -0.03338 | 1.034175 | 0.202182 |
| miR-27a-5p | 6.391223 | 14.04205 | 6.993859 | 6.186789 | 0.222668 |
| miR-23a-3p | -1.81059 | 1.54655 | -1.26896 | -1.68186 | 0.255142 |
| miR-138-5p | 1.664193 | 4.824613 | 1.773007 | 2.250415 | 0.260588 |
| miR-223-3p | 4.898791 | 14.04205 | 7.283151 | 4.231434 | 0.260803 |
| miR-29b-3p | 14.64711 | 7.289764 | 9.847235 | 6.592717 | 0.282485 |
| miR-29a-3p | 0.361033 | 2.254076 | 0.495626 | 0.888596 | 0.294751 |
| let-7i-5p | 0.618048 | 3.40133 | 0.750911 | 1.483635 | 0.299205 |
| miR-99a-3p | 6.395538 | 14.04205 | 11.17703 | 13.74197 | 0.317156 |
| miR-497-5p | 6.040472 | 11.18192 | 9.39194 | 10.59181 | 0.326564 |
| miR-22-3p | 4.745312 | 6.245725 | 4.392669 | 5.597325 | 0.327375 |
| miR-29a-5p | 9.89208 | 9.27402 | 8.275012 | 13.74197 | 0.327981 |
| miR-30c-2-3p | 9.687842 | 8.298645 | 7.515607 | 13.74197 | 0.329571 |
| miR-29c-3p | -0.75208 | 0.71714 | -0.52938 | -0.23468 | 0.337332 |
| miR-27b-3p | 2.319619 | 4.578598 | 2.728777 | 3.144156 | 0.349498 |
| miR-27a-3p | 4.563127 | 5.834861 | 7.81373 | 4.256679 | 0.350622 |
| miR-24-3p | 1.032945 | 3.579917 | 1.425656 | 2.050145 | 0.353436 |
| miR-300-5p | 10.63408 | 14.04205 | 8.065022 | 13.74197 | 0.353521 |
| miR-21-5p | 2.238239 | 7.82199 | 6.638849 | 1.257259 | 0.403203 |
| miR-30a-3p | 1.983031 | 3.088851 | 1.578011 | 3.018532 | 0.409004 |
| miR-30a-5p | 0.962602 | 2.411829 | 1.956004 | 1.780222 | 0.413694 |
| miR-29c-5p | 7.273658 | 6.78621 | 5.526517 | 9.310838 | 0.428694 |
| miR-30c-5p | 2.434092 | 5.473111 | 2.665012 | 4.607312 | 0.438241 |
| miR-551b-3p | 14.64711 | 14.04205 | 14.7653 | 13.74197 | 0.446222 |
| miR-92b-3p | -0.85107 | -0.73304 | -1.40031 | -0.34512 | 0.446588 |
| miR-330-5p | 6.796929 | 10.96154 | 10.49144 | 6.435259 | 0.449672 |
| miR-99a-5p | 6.267643 | 6.891987 | 6.227177 | 6.846875 | 0.465686 |
| let-7i-3p | 10.07924 | 6.869543 | 10.21553 | 7.084315 | 0.472363 |
| miR-22-5p | 5.885272 | 13.20852 | 5.877726 | 13.74197 | 0.482708 |

Table S6. miRNAs classification by cellular components.

| Cellular component | No. of genes in the dataset | No. of genes in the background dataset | Percentage of genes | Fold enrichment | P-value (Hypergeometric test) | Bonferroni method | BH method | Q-value (Storey-Tibshirani method) |
| --- | --- | --- | --- | --- | --- | --- | --- | --- |
| Plasma membrane | 3386 | 3479 | 24,24633 | 1,014248 | 6,39E-07 | 0,000501 | 0,000501 | 0,002297263 |
| Integral to  plasma membrane | 919 | 934 | 6,580738 | 1,025369 | 1,03E-05 | 0,008082 | 0,004041 | 0,01853878 |
| Extracellular | 1780 | 1825 | 12,74615 | 1,01641 | 7,17E-05 | 0,0562 | 0,018733 | 0,085939786 |
| Golgi aparatus | 878 | 897 | 6,287146 | 1,020032 | 0,000786 | 0,616454 | 0,154114 | 0,70700649 |
| Cytoplasm | 5490 | 5684 | 39,31257 | 1,006537 | 0,001101 | 0,863497 | 0,172699 | 0,792270187 |
| Membrane fraction | 341 | 346 | 2,441819 | 1,027046 | 0,004583 | 1 | 0,598833 | 1 |
| Extracellular region | 434 | 442 | 3,107769 | 1,023244 | 0,006191 | 1 | 0,69339 | 1 |
| Peroxisome | 111 | 111 | 0,794844 | 1,042105 | 0,010095 | 1 | 0,989302 | 1 |
| Endosome | 298 | 303 | 2,133906 | 1,024909 | 0,015095 | 1 | 1 | 1 |
| Endoplasmic reticulum | 1072 | 1104 | 7,676334 | 1,0119 | 0,023302 | 1 | 1 | 1 |
| Extracellular space | 395 | 404 | 2,8285 | 1,018891 | 0,032373 | 1 | 1 | 1 |
| Extracellular matrix | 119 | 120 | 0,85213 | 1,033422 | 0,042329 | 1 | 1 | 1 |
| Microsome | 154 | 156 | 1,102757 | 1,028746 | 0,045774 | 1 | 1 | 1 |
| Membrane | 342 | 350 | 2,44898 | 1,018286 | 0,052276 | 1 | 1 | 1 |
| Ribosome | 142 | 144 | 1,016828 | 1,027633 | 0,065777 | 1 | 1 | 1 |
| Protein complex | 65 | 65 | 0,465449 | 1,042105 | 0,068097 | 1 | 1 | 1 |
| Perinuclear region | 129 | 131 | 0,923738 | 1,026196 | 0,096312 | 1 | 1 | 1 |
| Golgi membrane | 55 | 55 | 0,393842 | 1,042105 | 0,103037 | 1 | 1 | 1 |
| Intracellular membrane-bounded organelle | 125 | 127 | 0,895095 | 1,025695 | 0,108014 | 1 | 1 | 1 |
| Others | 51 | 51 | 0,365199 | 1,042105 | 0,121591 | 1 | 1 | 1 |
| Microtubule cytoskeleton | 47 | 47 | 0,336556 | 1,042105 | 0,143481 | 1 | 1 | 1 |
| Spliceosomal complex | 46 | 46 | 0,329395 | 1,042105 | 0,149542 | 1 | 1 | 1 |
| Focal adhesion | 45 | 45 | 0,322234 | 1,042105 | 0,155859 | 1 | 1 | 1 |
| Nuclear pore | 42 | 42 | 0,300752 | 1,042105 | 0,176453 | 1 | 1 | 1 |
| Voltage-gated potassium channel complex | 42 | 42 | 0,300752 | 1,042105 | 0,176453 | 1 | 1 | 1 |
| Cell projection | 41 | 41 | 0,293591 | 1,042105 | 0,183905 | 1 | 1 | 1 |
| Basolateral plasma membrane | 40 | 40 | 0,28643 | 1,042105 | 0,19167 | 1 | 1 | 1 |
| PML body | 37 | 37 | 0,264948 | 1,042105 | 0,216987 | 1 | 1 | 1 |
| Nuclear matrix | 37 | 37 | 0,264948 | 1,042105 | 0,216987 | 1 | 1 | 1 |
| Proteinaceous extracellular matrix | 69 | 70 | 0,494092 | 1,02722 | 0,21931 | 1 | 1 | 1 |
| Cytosolic small ribosomal subunit | 36 | 36 | 0,257787 | 1,042105 | 0,226146 | 1 | 1 | 1 |
| Membrane raft | 36 | 36 | 0,257787 | 1,042105 | 0,226146 | 1 | 1 | 1 |
| Cytosolic large ribosomal subunit | 36 | 36 | 0,257787 | 1,042105 | 0,226146 | 1 | 1 | 1 |
| Intermediate filament | 36 | 36 | 0,257787 | 1,042105 | 0,226146 | 1 | 1 | 1 |
| External side of plasma membrane | 35 | 35 | 0,250627 | 1,042105 | 0,235692 | 1 | 1 | 1 |
| Ribonucleoprotein complex | 64 | 65 | 0,458289 | 1,026075 | 0,255324 | 1 | 1 | 1 |
| Synapse | 33 | 33 | 0,236305 | 1,042105 | 0,256008 | 1 | 1 | 1 |
| Chromatin | 32 | 32 | 0,229144 | 1,042105 | 0,266812 | 1 | 1 | 1 |
| Sarcoplasmic reticulum | 32 | 32 | 0,229144 | 1,042105 | 0,266812 | 1 | 1 | 1 |
| Cytosol | 1135 | 1178 | 8,127462 | 1,004066 | 0,267572 | 1 | 1 | 1 |
| Nuclear speck | 31 | 31 | 0,221984 | 1,042105 | 0,278071 | 1 | 1 | 1 |
| Kinetochore | 88 | 90 | 0,630147 | 1,01895 | 0,289614 | 1 | 1 | 1 |
| ER-Golgi intermediate compartment | 30 | 30 | 0,214823 | 1,042105 | 0,289804 | 1 | 1 | 1 |
| Mitochondrial outer membrane | 29 | 29 | 0,207662 | 1,042105 | 0,302032 | 1 | 1 | 1 |
| Dendrite | 27 | 27 | 0,19334 | 1,042105 | 0,328054 | 1 | 1 | 1 |
| Endosome membrane | 27 | 27 | 0,19334 | 1,042105 | 0,328054 | 1 | 1 | 1 |
| Axon | 27 | 27 | 0,19334 | 1,042105 | 0,328054 | 1 | 1 | 1 |
| MLL1 complex | 27 | 27 | 0,19334 | 1,042105 | 0,328054 | 1 | 1 | 1 |
| Cytoskeleton | 412 | 427 | 2,950233 | 1,005498 | 0,34187 | 1 | 1 | 1 |
| Cytoplasmic vesicle | 155 | 160 | 1,109918 | 1,009541 | 0,368588 | 1 | 1 | 1 |
| Ruffle | 24 | 24 | 0,171858 | 1,042105 | 0,371343 | 1 | 1 | 1 |
| Spindle microtubule | 24 | 24 | 0,171858 | 1,042105 | 0,371343 | 1 | 1 | 1 |
| Actin filament | 24 | 24 | 0,171858 | 1,042105 | 0,371343 | 1 | 1 | 1 |
| Microtubule associated complex | 24 | 24 | 0,171858 | 1,042105 | 0,371343 | 1 | 1 | 1 |
| Mediator complex | 23 | 23 | 0,164697 | 1,042105 | 0,387004 | 1 | 1 | 1 |
| Lamellipodium | 23 | 23 | 0,164697 | 1,042105 | 0,387004 | 1 | 1 | 1 |
| Secretory granule | 49 | 50 | 0,350877 | 1,021267 | 0,394468 | 1 | 1 | 1 |
| Integrin complex | 22 | 22 | 0,157537 | 1,042105 | 0,403324 | 1 | 1 | 1 |
| Centriole | 22 | 22 | 0,157537 | 1,042105 | 0,403324 | 1 | 1 | 1 |
| Endocytic vesicle membrane | 22 | 22 | 0,157537 | 1,042105 | 0,403324 | 1 | 1 | 1 |
| Cell surface | 198 | 205 | 1,41783 | 1,006523 | 0,409459 | 1 | 1 | 1 |
| Histone deacetylase complex | 21 | 21 | 0,150376 | 1,042105 | 0,420332 | 1 | 1 | 1 |
| Cajal body | 21 | 21 | 0,150376 | 1,042105 | 0,420332 | 1 | 1 | 1 |
| Intracellular | 146 | 151 | 1,045471 | 1,007601 | 0,425111 | 1 | 1 | 1 |
| Endoplasmic reticulum membrane | 145 | 150 | 1,03831 | 1,007371 | 0,431651 | 1 | 1 | 1 |
| Voltage-gated calcium channel complex | 20 | 20 | 0,143215 | 1,042105 | 0,438056 | 1 | 1 | 1 |
| Cell cortex | 20 | 20 | 0,143215 | 1,042105 | 0,438056 | 1 | 1 | 1 |
| Integral to membrane | 1635 | 1702 | 11,70784 | 1,001083 | 0,43998 | 1 | 1 | 1 |
| Nuclear envelope | 69 | 71 | 0,494092 | 1,012754 | 0,448571 | 1 | 1 | 1 |
| Lysosome | 1556 | 1620 | 11,14214 | 1,000936 | 0,455412 | 1 | 1 | 1 |
| Clathrin-coated vesicle | 19 | 19 | 0,136054 | 1,042105 | 0,456525 | 1 | 1 | 1 |
| Stored secretory granule | 19 | 19 | 0,136054 | 1,042105 | 0,456525 | 1 | 1 | 1 |
| Anaphase-promoting complex | 18 | 18 | 0,128894 | 1,042105 | 0,475772 | 1 | 1 | 1 |
| Ubiquitin ligase complex | 42 | 43 | 0,300752 | 1,017876 | 0,476755 | 1 | 1 | 1 |
| Apical plasma membrane | 41 | 42 | 0,293591 | 1,017299 | 0,489415 | 1 | 1 | 1 |
| Heterogeneous nuclear ribonucleoprotein complex | 17 | 17 | 0,121733 | 1,042105 | 0,495829 | 1 | 1 | 1 |
| Mitochondrial small ribosomal subunit | 17 | 17 | 0,121733 | 1,042105 | 0,495829 | 1 | 1 | 1 |
| Transcriptional repressor complex | 17 | 17 | 0,121733 | 1,042105 | 0,495829 | 1 | 1 | 1 |
| Transcription factor TFIID complex | 17 | 17 | 0,121733 | 1,042105 | 0,495829 | 1 | 1 | 1 |
| Nuclear chromosome | 17 | 17 | 0,121733 | 1,042105 | 0,495829 | 1 | 1 | 1 |
| Endocytic vesicle | 16 | 16 | 0,114572 | 1,042105 | 0,51673 | 1 | 1 | 1 |
| Small nuclear ribonucleoprotein complex | 16 | 16 | 0,114572 | 1,042105 | 0,51673 | 1 | 1 | 1 |
| Eukaryotic translation initiation factor 3 complex | 15 | 15 | 0,107411 | 1,042105 | 0,53851 | 1 | 1 | 1 |
| SCF ubiquitin ligase complex | 15 | 15 | 0,107411 | 1,042105 | 0,53851 | 1 | 1 | 1 |
| Muscle myosin complex | 15 | 15 | 0,107411 | 1,042105 | 0,53851 | 1 | 1 | 1 |
| Sarcomere | 15 | 15 | 0,107411 | 1,042105 | 0,53851 | 1 | 1 | 1 |
| Cell junction | 37 | 38 | 0,264948 | 1,014689 | 0,542197 | 1 | 1 | 1 |
| NuA4 histone acetyltransferase complex | 14 | 14 | 0,100251 | 1,042105 | 0,561207 | 1 | 1 | 1 |
| Lysosomal membrane | 14 | 14 | 0,100251 | 1,042105 | 0,561207 | 1 | 1 | 1 |
| Transcription factor TFTC complex | 14 | 14 | 0,100251 | 1,042105 | 0,561207 | 1 | 1 | 1 |
| Tight junction | 14 | 14 | 0,100251 | 1,042105 | 0,561207 | 1 | 1 | 1 |
| Platelet alpha granule lumen | 34 | 35 | 0,243466 | 1,012339 | 0,583876 | 1 | 1 | 1 |
| Peroxisomal membrane | 13 | 13 | 0,09309 | 1,042105 | 0,584859 | 1 | 1 | 1 |
| Apical part of cell | 13 | 13 | 0,09309 | 1,042105 | 0,584859 | 1 | 1 | 1 |
| Mitochondrial ribosome | 13 | 13 | 0,09309 | 1,042105 | 0,584859 | 1 | 1 | 1 |
| Coated pit | 13 | 13 | 0,09309 | 1,042105 | 0,584859 | 1 | 1 | 1 |
| Extrinsic to membrane | 13 | 13 | 0,09309 | 1,042105 | 0,584859 | 1 | 1 | 1 |
| SWI/SNF complex | 13 | 13 | 0,09309 | 1,042105 | 0,584859 | 1 | 1 | 1 |
| Small ribosomal subunit | 13 | 13 | 0,09309 | 1,042105 | 0,584859 | 1 | 1 | 1 |
| DNA-directed RNA polymerase II, core complex | 13 | 13 | 0,09309 | 1,042105 | 0,584859 | 1 | 1 | 1 |
| Mitochondrial intermembrane space | 12 | 12 | 0,085929 | 1,042105 | 0,609506 | 1 | 1 | 1 |
| HOPS complex | 12 | 12 | 0,085929 | 1,042105 | 0,609506 | 1 | 1 | 1 |
| STAGA complex | 12 | 12 | 0,085929 | 1,042105 | 0,609506 | 1 | 1 | 1 |
| T cell receptor complex | 12 | 12 | 0,085929 | 1,042105 | 0,609506 | 1 | 1 | 1 |
| Condensed nuclear chromosome | 12 | 12 | 0,085929 | 1,042105 | 0,609506 | 1 | 1 | 1 |
| Neuron projection | 12 | 12 | 0,085929 | 1,042105 | 0,609506 | 1 | 1 | 1 |
| NuRD complex | 12 | 12 | 0,085929 | 1,042105 | 0,609506 | 1 | 1 | 1 |
| Nuclear body | 12 | 12 | 0,085929 | 1,042105 | 0,609506 | 1 | 1 | 1 |
| Nuclear membrane | 75 | 78 | 0,537057 | 1,002029 | 0,612586 | 1 | 1 | 1 |
| Holo TFIIH complex | 11 | 11 | 0,078768 | 1,042105 | 0,63519 | 1 | 1 | 1 |
| Receptor complex | 11 | 11 | 0,078768 | 1,042105 | 0,63519 | 1 | 1 | 1 |
| Histone methyltransferase complex | 11 | 11 | 0,078768 | 1,042105 | 0,63519 | 1 | 1 | 1 |
| Ruffle membrane | 11 | 11 | 0,078768 | 1,042105 | 0,63519 | 1 | 1 | 1 |
| Nicotinic acetylcholine-gated receptor-channel complex | 11 | 11 | 0,078768 | 1,042105 | 0,63519 | 1 | 1 | 1 |
| High-density lipoprotein particle | 11 | 11 | 0,078768 | 1,042105 | 0,63519 | 1 | 1 | 1 |
| Heterotrimeric G-protein complex | 11 | 11 | 0,078768 | 1,042105 | 0,63519 | 1 | 1 | 1 |
| Golgi lumen | 11 | 11 | 0,078768 | 1,042105 | 0,63519 | 1 | 1 | 1 |
| Platelet alpha granule membrane | 11 | 11 | 0,078768 | 1,042105 | 0,63519 | 1 | 1 | 1 |
| Integral to peroxisomal membrane | 11 | 11 | 0,078768 | 1,042105 | 0,63519 | 1 | 1 | 1 |
| Peroxisomal matrix | 11 | 11 | 0,078768 | 1,042105 | 0,63519 | 1 | 1 | 1 |
| CUL4 RING ubiquitin ligase complex | 11 | 11 | 0,078768 | 1,042105 | 0,63519 | 1 | 1 | 1 |
| Intermediate filament cytoskeleton | 11 | 11 | 0,078768 | 1,042105 | 0,63519 | 1 | 1 | 1 |
| Acrosome | 11 | 11 | 0,078768 | 1,042105 | 0,63519 | 1 | 1 | 1 |
| Desmosome | 11 | 11 | 0,078768 | 1,042105 | 0,63519 | 1 | 1 | 1 |
| Trans-Golgi network | 30 | 31 | 0,214823 | 1,0085 | 0,641808 | 1 | 1 | 1 |
| Axonemal dynein complex | 10 | 10 | 0,071608 | 1,042105 | 0,661954 | 1 | 1 | 1 |
| Cilium | 10 | 10 | 0,071608 | 1,042105 | 0,661954 | 1 | 1 | 1 |
| Filopodium | 10 | 10 | 0,071608 | 1,042105 | 0,661954 | 1 | 1 | 1 |
| Myosin complex | 10 | 10 | 0,071608 | 1,042105 | 0,661954 | 1 | 1 | 1 |
| Condensed chromosome | 10 | 10 | 0,071608 | 1,042105 | 0,661954 | 1 | 1 | 1 |
| Recycling endosome | 10 | 10 | 0,071608 | 1,042105 | 0,661954 | 1 | 1 | 1 |
| Hemoglobin complex | 10 | 10 | 0,071608 | 1,042105 | 0,661954 | 1 | 1 | 1 |
| Nuclear inner membrane | 10 | 10 | 0,071608 | 1,042105 | 0,661954 | 1 | 1 | 1 |
| Platelet alpha granule | 10 | 10 | 0,071608 | 1,042105 | 0,661954 | 1 | 1 | 1 |
| Dystrophin-associated glycoprotein complex | 10 | 10 | 0,071608 | 1,042105 | 0,661954 | 1 | 1 | 1 |
| Nup107-160 complex | 10 | 10 | 0,071608 | 1,042105 | 0,661954 | 1 | 1 | 1 |
| Microtubule organizing center | 10 | 10 | 0,071608 | 1,042105 | 0,661954 | 1 | 1 | 1 |
| Nuclear chromatin | 10 | 10 | 0,071608 | 1,042105 | 0,661954 | 1 | 1 | 1 |
| Mitochondrial inner membrane presequence translocase complex | 9 | 9 | 0,064447 | 1,042105 | 0,689843 | 1 | 1 | 1 |
| Oligosaccharyltransferase complex | 9 | 9 | 0,064447 | 1,042105 | 0,689843 | 1 | 1 | 1 |
| Golgi transport complex | 9 | 9 | 0,064447 | 1,042105 | 0,689843 | 1 | 1 | 1 |
| Cul3-RING ubiquitin ligase complex | 9 | 9 | 0,064447 | 1,042105 | 0,689843 | 1 | 1 | 1 |
| Cis-Golgi network | 9 | 9 | 0,064447 | 1,042105 | 0,689843 | 1 | 1 | 1 |
| Eukaryotic translation initiation factor 4F complex | 9 | 9 | 0,064447 | 1,042105 | 0,689843 | 1 | 1 | 1 |
| SNARE complex | 9 | 9 | 0,064447 | 1,042105 | 0,689843 | 1 | 1 | 1 |
| ESC/E(Z) complex | 9 | 9 | 0,064447 | 1,042105 | 0,689843 | 1 | 1 | 1 |
| Spectrin | 9 | 9 | 0,064447 | 1,042105 | 0,689843 | 1 | 1 | 1 |
| U12-type spliceosomal complex | 9 | 9 | 0,064447 | 1,042105 | 0,689843 | 1 | 1 | 1 |
| Kinesin complex | 9 | 9 | 0,064447 | 1,042105 | 0,689843 | 1 | 1 | 1 |
| Filamentous actin | 9 | 9 | 0,064447 | 1,042105 | 0,689843 | 1 | 1 | 1 |
| Internal side of plasma membrane | 8 | 8 | 0,057286 | 1,042105 | 0,718906 | 1 | 1 | 1 |
| Cytoplasmic microtubule | 8 | 8 | 0,057286 | 1,042105 | 0,718906 | 1 | 1 | 1 |
| Centriolar satellite | 8 | 8 | 0,057286 | 1,042105 | 0,718906 | 1 | 1 | 1 |
| Spherical high-density lipoprotein particle | 8 | 8 | 0,057286 | 1,042105 | 0,718906 | 1 | 1 | 1 |
| HAUS complex | 8 | 8 | 0,057286 | 1,042105 | 0,718906 | 1 | 1 | 1 |
| Chylomicron | 8 | 8 | 0,057286 | 1,042105 | 0,718906 | 1 | 1 | 1 |
| Lateral plasma membrane | 8 | 8 | 0,057286 | 1,042105 | 0,718906 | 1 | 1 | 1 |
| Z disc | 8 | 8 | 0,057286 | 1,042105 | 0,718906 | 1 | 1 | 1 |
| NADPH oxidase complex | 8 | 8 | 0,057286 | 1,042105 | 0,718906 | 1 | 1 | 1 |
| IkappaB kinase complex | 8 | 8 | 0,057286 | 1,042105 | 0,718906 | 1 | 1 | 1 |
| Specific granule | 8 | 8 | 0,057286 | 1,042105 | 0,718906 | 1 | 1 | 1 |
| Integral to membrane of membrane fraction | 8 | 8 | 0,057286 | 1,042105 | 0,718906 | 1 | 1 | 1 |
| Troponin complex | 8 | 8 | 0,057286 | 1,042105 | 0,718906 | 1 | 1 | 1 |
| Neuronal cell body | 8 | 8 | 0,057286 | 1,042105 | 0,718906 | 1 | 1 | 1 |
| Chromatin remodeling complex | 8 | 8 | 0,057286 | 1,042105 | 0,718906 | 1 | 1 | 1 |
| Outer kinetochore of condensed chromosome | 8 | 8 | 0,057286 | 1,042105 | 0,718906 | 1 | 1 | 1 |
| Growth cone | 8 | 8 | 0,057286 | 1,042105 | 0,718906 | 1 | 1 | 1 |
| Sarcolemma | 8 | 8 | 0,057286 | 1,042105 | 0,718906 | 1 | 1 | 1 |
| Microtubule basal body | 8 | 8 | 0,057286 | 1,042105 | 0,718906 | 1 | 1 | 1 |
| MOZ/MORF histone acetyltransferase complex | 8 | 8 | 0,057286 | 1,042105 | 0,718906 | 1 | 1 | 1 |
| Intracellular vesicle (None) | 8 | 8 | 0,057286 | 1,042105 | 0,718906 | 1 | 1 | 1 |
| Proteasome regulatory particle | 8 | 8 | 0,057286 | 1,042105 | 0,718906 | 1 | 1 | 1 |
| Anchored to plasma membrane | 8 | 8 | 0,057286 | 1,042105 | 0,718906 | 1 | 1 | 1 |
| Nuclear telomere cap complex | 8 | 8 | 0,057286 | 1,042105 | 0,718906 | 1 | 1 | 1 |
| Mitochondrial respiratory chain | 8 | 8 | 0,057286 | 1,042105 | 0,718906 | 1 | 1 | 1 |
| MLL5-L complex | 8 | 8 | 0,057286 | 1,042105 | 0,718906 | 1 | 1 | 1 |
| Transcription export complex | 8 | 8 | 0,057286 | 1,042105 | 0,718906 | 1 | 1 | 1 |
| Small nucleolar ribonucleoprotein complex | 8 | 8 | 0,057286 | 1,042105 | 0,718906 | 1 | 1 | 1 |
| Cytoplasmic part | 8 | 8 | 0,057286 | 1,042105 | 0,718906 | 1 | 1 | 1 |
| Stress granule | 8 | 8 | 0,057286 | 1,042105 | 0,718906 | 1 | 1 | 1 |
| Microtubule | 123 | 129 | 0,880773 | 0,993639 | 0,73364 | 1 | 1 | 1 |
| Caveola | 23 | 24 | 0,164697 | 0,998702 | 0,747213 | 1 | 1 | 1 |
| Spindle pole | 23 | 24 | 0,164697 | 0,998702 | 0,747213 | 1 | 1 | 1 |
| Synaptic vesicle | 23 | 24 | 0,164697 | 0,998702 | 0,747213 | 1 | 1 | 1 |
| Endoplasmic reticulum lumen | 23 | 24 | 0,164697 | 0,998702 | 0,747213 | 1 | 1 | 1 |
| Transcription factor complex | 41 | 43 | 0,293591 | 0,993646 | 0,748945 | 1 | 1 | 1 |
| Brush border membrane | 7 | 7 | 0,050125 | 1,042105 | 0,749191 | 1 | 1 | 1 |
| Gap junction | 7 | 7 | 0,050125 | 1,042105 | 0,749191 | 1 | 1 | 1 |
| Cortical cytoskeleton | 7 | 7 | 0,050125 | 1,042105 | 0,749191 | 1 | 1 | 1 |
| Nuclear outer membrane | 7 | 7 | 0,050125 | 1,042105 | 0,749191 | 1 | 1 | 1 |
| Early endosome membrane | 7 | 7 | 0,050125 | 1,042105 | 0,749191 | 1 | 1 | 1 |
| Cul4A-RING ubiquitin ligase complex | 7 | 7 | 0,050125 | 1,042105 | 0,749191 | 1 | 1 | 1 |
| Polysome | 7 | 7 | 0,050125 | 1,042105 | 0,749191 | 1 | 1 | 1 |
| U7 snRNP | 7 | 7 | 0,050125 | 1,042105 | 0,749191 | 1 | 1 | 1 |
| Unconventional myosin complex | 7 | 7 | 0,050125 | 1,042105 | 0,749191 | 1 | 1 | 1 |
| Sin3 complex | 7 | 7 | 0,050125 | 1,042105 | 0,749191 | 1 | 1 | 1 |
| Cyclin-dependent protein kinase holoenzyme complex | 7 | 7 | 0,050125 | 1,042105 | 0,749191 | 1 | 1 | 1 |
| Arp2/3 protein complex | 7 | 7 | 0,050125 | 1,042105 | 0,749191 | 1 | 1 | 1 |
| Melanosome | 7 | 7 | 0,050125 | 1,042105 | 0,749191 | 1 | 1 | 1 |
| BBSome | 7 | 7 | 0,050125 | 1,042105 | 0,749191 | 1 | 1 | 1 |
| ATP-binding cassette (ABC) transporter complex | 7 | 7 | 0,050125 | 1,042105 | 0,749191 | 1 | 1 | 1 |
| Fibrinogen complex | 7 | 7 | 0,050125 | 1,042105 | 0,749191 | 1 | 1 | 1 |
| Cleavage furrow | 7 | 7 | 0,050125 | 1,042105 | 0,749191 | 1 | 1 | 1 |
| Catenin complex | 7 | 7 | 0,050125 | 1,042105 | 0,749191 | 1 | 1 | 1 |
| Recycling endosome membrane | 7 | 7 | 0,050125 | 1,042105 | 0,749191 | 1 | 1 | 1 |
| Golgi stack | 7 | 7 | 0,050125 | 1,042105 | 0,749191 | 1 | 1 | 1 |
| Condensed chromosome kinetochore | 7 | 7 | 0,050125 | 1,042105 | 0,749191 | 1 | 1 | 1 |
| Basal lamina | 7 | 7 | 0,050125 | 1,042105 | 0,749191 | 1 | 1 | 1 |
| Postsynaptic membrane | 7 | 7 | 0,050125 | 1,042105 | 0,749191 | 1 | 1 | 1 |
| Immunological synapse | 7 | 7 | 0,050125 | 1,042105 | 0,749191 | 1 | 1 | 1 |
| Eukaryotic translation initiation factor 2B complex | 6 | 6 | 0,042965 | 1,042105 | 0,780749 | 1 | 1 | 1 |
| F-actin capping protein complex | 6 | 6 | 0,042965 | 1,042105 | 0,780749 | 1 | 1 | 1 |
| Vacuolar proton-transporting V-type ATPase complex | 6 | 6 | 0,042965 | 1,042105 | 0,780749 | 1 | 1 | 1 |
| Sarcoplasmic reticulum membrane | 6 | 6 | 0,042965 | 1,042105 | 0,780749 | 1 | 1 | 1 |
| U5 snRNP | 6 | 6 | 0,042965 | 1,042105 | 0,780749 | 1 | 1 | 1 |
| Beta-catenin destruction complex | 6 | 6 | 0,042965 | 1,042105 | 0,780749 | 1 | 1 | 1 |
| Autophagic vacuole | 6 | 6 | 0,042965 | 1,042105 | 0,780749 | 1 | 1 | 1 |
| COPI vesicle coat | 6 | 6 | 0,042965 | 1,042105 | 0,780749 | 1 | 1 | 1 |
| Signal recognition particle, endoplasmic reticulum targeting | 6 | 6 | 0,042965 | 1,042105 | 0,780749 | 1 | 1 | 1 |
| Gamma-tubulin ring complex | 6 | 6 | 0,042965 | 1,042105 | 0,780749 | 1 | 1 | 1 |
| DNA-directed RNA polymerase III complex | 6 | 6 | 0,042965 | 1,042105 | 0,780749 | 1 | 1 | 1 |
| Neurofilament | 6 | 6 | 0,042965 | 1,042105 | 0,780749 | 1 | 1 | 1 |
| Inclusion body | 6 | 6 | 0,042965 | 1,042105 | 0,780749 | 1 | 1 | 1 |
| Telomerase holoenzyme complex | 6 | 6 | 0,042965 | 1,042105 | 0,780749 | 1 | 1 | 1 |
| Golgi-associated vesicle | 6 | 6 | 0,042965 | 1,042105 | 0,780749 | 1 | 1 | 1 |
| SWI/SNF-type complex | 6 | 6 | 0,042965 | 1,042105 | 0,780749 | 1 | 1 | 1 |
| AP-2 adaptor complex | 6 | 6 | 0,042965 | 1,042105 | 0,780749 | 1 | 1 | 1 |
| Gamma-tubulin complex | 6 | 6 | 0,042965 | 1,042105 | 0,780749 | 1 | 1 | 1 |
| Costamere | 6 | 6 | 0,042965 | 1,042105 | 0,780749 | 1 | 1 | 1 |
| Nonhomologous end joining complex | 6 | 6 | 0,042965 | 1,042105 | 0,780749 | 1 | 1 | 1 |
| Axin-APC-beta-catenin-GSK3B complex | 6 | 6 | 0,042965 | 1,042105 | 0,780749 | 1 | 1 | 1 |
| DNA replication factor C complex | 6 | 6 | 0,042965 | 1,042105 | 0,780749 | 1 | 1 | 1 |
| Cell leading edge | 6 | 6 | 0,042965 | 1,042105 | 0,780749 | 1 | 1 | 1 |
| Cytoplasmic dynein complex | 6 | 6 | 0,042965 | 1,042105 | 0,780749 | 1 | 1 | 1 |
| Smooth endoplasmic reticulum | 6 | 6 | 0,042965 | 1,042105 | 0,780749 | 1 | 1 | 1 |
| Sodium:potassium-exchanging ATPase complex | 6 | 6 | 0,042965 | 1,042105 | 0,780749 | 1 | 1 | 1 |
| PCAF complex | 6 | 6 | 0,042965 | 1,042105 | 0,780749 | 1 | 1 | 1 |
| I band | 6 | 6 | 0,042965 | 1,042105 | 0,780749 | 1 | 1 | 1 |
| Trans-Golgi network transport vesicle | 6 | 6 | 0,042965 | 1,042105 | 0,780749 | 1 | 1 | 1 |
| Transcription factor TFIIIC complex | 6 | 6 | 0,042965 | 1,042105 | 0,780749 | 1 | 1 | 1 |
| Pre-snoRNP complex | 6 | 6 | 0,042965 | 1,042105 | 0,780749 | 1 | 1 | 1 |
| Origin recognition complex | 6 | 6 | 0,042965 | 1,042105 | 0,780749 | 1 | 1 | 1 |
| Golgi vesicle | 6 | 6 | 0,042965 | 1,042105 | 0,780749 | 1 | 1 | 1 |
| Cyclin-dependent protein kinase activating kinase holoenzyme complex | 6 | 6 | 0,042965 | 1,042105 | 0,780749 | 1 | 1 | 1 |
| Azurophil granule | 6 | 6 | 0,042965 | 1,042105 | 0,780749 | 1 | 1 | 1 |
| Chromatin assembly complex | 6 | 6 | 0,042965 | 1,042105 | 0,780749 | 1 | 1 | 1 |
| Axoneme | 6 | 6 | 0,042965 | 1,042105 | 0,780749 | 1 | 1 | 1 |
| Secretory vesicle (None) | 6 | 6 | 0,042965 | 1,042105 | 0,780749 | 1 | 1 | 1 |
| Late endosome | 38 | 40 | 0,272109 | 0,990013 | 0,781448 | 1 | 1 | 1 |
| Transport vesicle | 20 | 21 | 0,143215 | 0,992505 | 0,792527 | 1 | 1 | 1 |
| Basement membrane | 20 | 21 | 0,143215 | 0,992505 | 0,792527 | 1 | 1 | 1 |
| Perinuclear region of cytoplasm | 113 | 119 | 0,809166 | 0,989567 | 0,793887 | 1 | 1 | 1 |
| Cell-cell junction | 36 | 38 | 0,257787 | 0,987272 | 0,802595 | 1 | 1 | 1 |
| Spectrin-associated cytoskeleton | 5 | 5 | 0,035804 | 1,042105 | 0,813635 | 1 | 1 | 1 |
| mRNA cap binding complex | 5 | 5 | 0,035804 | 1,042105 | 0,813635 | 1 | 1 | 1 |
| N-methyl-D-aspartate selective glutamate receptor complex | 5 | 5 | 0,035804 | 1,042105 | 0,813635 | 1 | 1 | 1 |
| Connexon complex | 5 | 5 | 0,035804 | 1,042105 | 0,813635 | 1 | 1 | 1 |
| Cytoplasmic vesicle membrane | 5 | 5 | 0,035804 | 1,042105 | 0,813635 | 1 | 1 | 1 |
| Dendritic spine | 5 | 5 | 0,035804 | 1,042105 | 0,813635 | 1 | 1 | 1 |
| Serine C-palmitoyltransferase complex | 5 | 5 | 0,035804 | 1,042105 | 0,813635 | 1 | 1 | 1 |
| AP-type membrane coat adaptor complex | 5 | 5 | 0,035804 | 1,042105 | 0,813635 | 1 | 1 | 1 |
| Cilium axoneme | 5 | 5 | 0,035804 | 1,042105 | 0,813635 | 1 | 1 | 1 |
| Rough endoplasmic reticulum | 5 | 5 | 0,035804 | 1,042105 | 0,813635 | 1 | 1 | 1 |
| THO complex part of transcription export complex | 5 | 5 | 0,035804 | 1,042105 | 0,813635 | 1 | 1 | 1 |
| Pseudopodium | 5 | 5 | 0,035804 | 1,042105 | 0,813635 | 1 | 1 | 1 |
| Kinetochore microtubule | 5 | 5 | 0,035804 | 1,042105 | 0,813635 | 1 | 1 | 1 |
| Endomembrane system | 5 | 5 | 0,035804 | 1,042105 | 0,813635 | 1 | 1 | 1 |
| Photoreceptor outer segment | 5 | 5 | 0,035804 | 1,042105 | 0,813635 | 1 | 1 | 1 |
| SUN-KASH complex | 5 | 5 | 0,035804 | 1,042105 | 0,813635 | 1 | 1 | 1 |
| Histone acetyltransferase complex | 5 | 5 | 0,035804 | 1,042105 | 0,813635 | 1 | 1 | 1 |
| COPII vesicle coat | 5 | 5 | 0,035804 | 1,042105 | 0,813635 | 1 | 1 | 1 |
| Chromatin silencing complex | 5 | 5 | 0,035804 | 1,042105 | 0,813635 | 1 | 1 | 1 |
| Paraspeckles | 5 | 5 | 0,035804 | 1,042105 | 0,813635 | 1 | 1 | 1 |
| Lipopolysaccharide receptor complex | 5 | 5 | 0,035804 | 1,042105 | 0,813635 | 1 | 1 | 1 |
| Collagen type IV | 5 | 5 | 0,035804 | 1,042105 | 0,813635 | 1 | 1 | 1 |
| Protein-DNA complex | 5 | 5 | 0,035804 | 1,042105 | 0,813635 | 1 | 1 | 1 |
| CRD-mediated mRNA stability complex | 5 | 5 | 0,035804 | 1,042105 | 0,813635 | 1 | 1 | 1 |
| Junctional sarcoplasmic reticulum membrane | 5 | 5 | 0,035804 | 1,042105 | 0,813635 | 1 | 1 | 1 |
| Golgi-associated vesicle membrane | 5 | 5 | 0,035804 | 1,042105 | 0,813635 | 1 | 1 | 1 |
| THO complex | 5 | 5 | 0,035804 | 1,042105 | 0,813635 | 1 | 1 | 1 |
| Pericentriolar material | 5 | 5 | 0,035804 | 1,042105 | 0,813635 | 1 | 1 | 1 |
| Cortical actin cytoskeleton | 5 | 5 | 0,035804 | 1,042105 | 0,813635 | 1 | 1 | 1 |
| AP-1 adaptor complex | 5 | 5 | 0,035804 | 1,042105 | 0,813635 | 1 | 1 | 1 |
| Condensin complex | 5 | 5 | 0,035804 | 1,042105 | 0,813635 | 1 | 1 | 1 |
| Nuclear heterochromatin | 5 | 5 | 0,035804 | 1,042105 | 0,813635 | 1 | 1 | 1 |
| Nucleolar riboNuclease P complex | 5 | 5 | 0,035804 | 1,042105 | 0,813635 | 1 | 1 | 1 |
| Presynaptic membrane | 5 | 5 | 0,035804 | 1,042105 | 0,813635 | 1 | 1 | 1 |
| Proton-transporting two-sector ATPase complex | 5 | 5 | 0,035804 | 1,042105 | 0,813635 | 1 | 1 | 1 |
| GPI-anchor transamidase complex | 5 | 5 | 0,035804 | 1,042105 | 0,813635 | 1 | 1 | 1 |
| Platelet dense granule membrane | 5 | 5 | 0,035804 | 1,042105 | 0,813635 | 1 | 1 | 1 |
| Keratin filament | 5 | 5 | 0,035804 | 1,042105 | 0,813635 | 1 | 1 | 1 |
| Myofibril | 5 | 5 | 0,035804 | 1,042105 | 0,813635 | 1 | 1 | 1 |
| Mitochondrial envelope | 5 | 5 | 0,035804 | 1,042105 | 0,813635 | 1 | 1 | 1 |
| Perinuclear vesicle (None) | 5 | 5 | 0,035804 | 1,042105 | 0,813635 | 1 | 1 | 1 |
| Midbody | 18 | 19 | 0,128894 | 0,987286 | 0,822217 | 1 | 1 | 1 |
| Integral to Golgi membrane | 18 | 19 | 0,128894 | 0,987286 | 0,822217 | 1 | 1 | 1 |
| Cytoplasmic membrane-bounded vesicle | 18 | 19 | 0,128894 | 0,987286 | 0,822217 | 1 | 1 | 1 |
| Cornified envelope | 18 | 19 | 0,128894 | 0,987286 | 0,822217 | 1 | 1 | 1 |
| Actin cytoskeleton | 125 | 132 | 0,895095 | 0,986846 | 0,834584 | 1 | 1 | 1 |
| Proteasome complex | 32 | 34 | 0,229144 | 0,980823 | 0,843158 | 1 | 1 | 1 |
| Zymogen granule | 32 | 34 | 0,229144 | 0,980823 | 0,843158 | 1 | 1 | 1 |
| Calcium- and calmodulin-dependent protein kinase complex | 4 | 4 | 0,028643 | 1,042105 | 0,847903 | 1 | 1 | 1 |
| cAMP-dependent protein kinase complex | 4 | 4 | 0,028643 | 1,042105 | 0,847903 | 1 | 1 | 1 |
| Cul4B-RING ubiquitin ligase complex | 4 | 4 | 0,028643 | 1,042105 | 0,847903 | 1 | 1 | 1 |
| Striated muscle thin filament | 4 | 4 | 0,028643 | 1,042105 | 0,847903 | 1 | 1 | 1 |
| DNA replication factor A complex | 4 | 4 | 0,028643 | 1,042105 | 0,847903 | 1 | 1 | 1 |
| Mitochondrial alpha-ketoglutarate dehydrogenase complex | 4 | 4 | 0,028643 | 1,042105 | 0,847903 | 1 | 1 | 1 |
| Plasma membrane enriched fraction | 4 | 4 | 0,028643 | 1,042105 | 0,847903 | 1 | 1 | 1 |
| Muscle thin filament tropomyosin | 4 | 4 | 0,028643 | 1,042105 | 0,847903 | 1 | 1 | 1 |
| Sarcoglycan complex | 4 | 4 | 0,028643 | 1,042105 | 0,847903 | 1 | 1 | 1 |
| Vesicle membrane | 4 | 4 | 0,028643 | 1,042105 | 0,847903 | 1 | 1 | 1 |
| Intermediate-density lipoprotein particle | 4 | 4 | 0,028643 | 1,042105 | 0,847903 | 1 | 1 | 1 |
| Nuclear RNA export factor complex | 4 | 4 | 0,028643 | 1,042105 | 0,847903 | 1 | 1 | 1 |
| Photoreceptor inner segment | 4 | 4 | 0,028643 | 1,042105 | 0,847903 | 1 | 1 | 1 |
| Apolipoprotein B mRNA editing enzyme complex | 4 | 4 | 0,028643 | 1,042105 | 0,847903 | 1 | 1 | 1 |
| Extrinsic to internal side of plasma membrane | 4 | 4 | 0,028643 | 1,042105 | 0,847903 | 1 | 1 | 1 |
| Zonula adherens | 4 | 4 | 0,028643 | 1,042105 | 0,847903 | 1 | 1 | 1 |
| Postsynaptic density | 4 | 4 | 0,028643 | 1,042105 | 0,847903 | 1 | 1 | 1 |
| Cdc73/Paf1 complex | 4 | 4 | 0,028643 | 1,042105 | 0,847903 | 1 | 1 | 1 |
| Dynactin complex | 4 | 4 | 0,028643 | 1,042105 | 0,847903 | 1 | 1 | 1 |
| Motile secondary cilium | 4 | 4 | 0,028643 | 1,042105 | 0,847903 | 1 | 1 | 1 |
| CCAAT-binding factor complex | 4 | 4 | 0,028643 | 1,042105 | 0,847903 | 1 | 1 | 1 |
| Phagocytic vesicle | 4 | 4 | 0,028643 | 1,042105 | 0,847903 | 1 | 1 | 1 |
| A band | 4 | 4 | 0,028643 | 1,042105 | 0,847903 | 1 | 1 | 1 |
| MIS12/MIND type complex | 4 | 4 | 0,028643 | 1,042105 | 0,847903 | 1 | 1 | 1 |
| Box C/D snoRNP complex | 4 | 4 | 0,028643 | 1,042105 | 0,847903 | 1 | 1 | 1 |
| Multivesicular body | 4 | 4 | 0,028643 | 1,042105 | 0,847903 | 1 | 1 | 1 |
| Vesicle | 4 | 4 | 0,028643 | 1,042105 | 0,847903 | 1 | 1 | 1 |
| SOSS complex | 4 | 4 | 0,028643 | 1,042105 | 0,847903 | 1 | 1 | 1 |
| Spindle midzone | 4 | 4 | 0,028643 | 1,042105 | 0,847903 | 1 | 1 | 1 |
| Vacuole | 4 | 4 | 0,028643 | 1,042105 | 0,847903 | 1 | 1 | 1 |
| Synaptonemal complex | 4 | 4 | 0,028643 | 1,042105 | 0,847903 | 1 | 1 | 1 |
| Death-inducing signaling complex | 4 | 4 | 0,028643 | 1,042105 | 0,847903 | 1 | 1 | 1 |
| Cytoplasmic mRNA processing body | 4 | 4 | 0,028643 | 1,042105 | 0,847903 | 1 | 1 | 1 |
| Proteasome core complex | 4 | 4 | 0,028643 | 1,042105 | 0,847903 | 1 | 1 | 1 |
| Secretory granule membrane | 4 | 4 | 0,028643 | 1,042105 | 0,847903 | 1 | 1 | 1 |
| Basal part of cell | 4 | 4 | 0,028643 | 1,042105 | 0,847903 | 1 | 1 | 1 |
| Collagen | 4 | 4 | 0,028643 | 1,042105 | 0,847903 | 1 | 1 | 1 |
| Ndc80 complex | 4 | 4 | 0,028643 | 1,042105 | 0,847903 | 1 | 1 | 1 |
| Polar microtubule | 4 | 4 | 0,028643 | 1,042105 | 0,847903 | 1 | 1 | 1 |
| Lipid particle | 4 | 4 | 0,028643 | 1,042105 | 0,847903 | 1 | 1 | 1 |
| U4/U6 x U5 tri-snRNP complex | 4 | 4 | 0,028643 | 1,042105 | 0,847903 | 1 | 1 | 1 |
| Nuclear replication fork | 4 | 4 | 0,028643 | 1,042105 | 0,847903 | 1 | 1 | 1 |
| Polysomal ribosome | 4 | 4 | 0,028643 | 1,042105 | 0,847903 | 1 | 1 | 1 |
| Nuclear lamina | 4 | 4 | 0,028643 | 1,042105 | 0,847903 | 1 | 1 | 1 |
| Voltage-gated sodium channel complex | 4 | 4 | 0,028643 | 1,042105 | 0,847903 | 1 | 1 | 1 |
| Early endosome | 65 | 69 | 0,465449 | 0,981702 | 0,853949 | 1 | 1 | 1 |
| Nucleoplasm | 427 | 449 | 3,057644 | 0,991046 | 0,855197 | 1 | 1 | 1 |
| Apical membrane | 15 | 16 | 0,107411 | 0,977014 | 0,865217 | 1 | 1 | 1 |
| Mitochondrial nucleoid | 29 | 31 | 0,207662 | 0,974894 | 0,871623 | 1 | 1 | 1 |
| Integral to endoplasmic reticulum membrane | 29 | 31 | 0,207662 | 0,974894 | 0,871623 | 1 | 1 | 1 |
| Spindle | 28 | 30 | 0,200501 | 0,972655 | 0,880663 | 1 | 1 | 1 |
| Oncostatin-M receptor complex | 3 | 3 | 0,021482 | 1,042105 | 0,883612 | 1 | 1 | 1 |
| Condensed nuclear chromosome, centromeric region | 3 | 3 | 0,021482 | 1,042105 | 0,883612 | 1 | 1 | 1 |
| Synaptosome | 3 | 3 | 0,021482 | 1,042105 | 0,883612 | 1 | 1 | 1 |
| Collagen type V | 3 | 3 | 0,021482 | 1,042105 | 0,883612 | 1 | 1 | 1 |
| Integral to nuclear inner membrane | 3 | 3 | 0,021482 | 1,042105 | 0,883612 | 1 | 1 | 1 |
| SAGA complex | 3 | 3 | 0,021482 | 1,042105 | 0,883612 | 1 | 1 | 1 |
| 6-phosphofructokinase complex | 3 | 3 | 0,021482 | 1,042105 | 0,883612 | 1 | 1 | 1 |
| TSC1-TSC2 complex | 3 | 3 | 0,021482 | 1,042105 | 0,883612 | 1 | 1 | 1 |
| Phagocytic vesicle membrane | 3 | 3 | 0,021482 | 1,042105 | 0,883612 | 1 | 1 | 1 |
| Microfibril | 3 | 3 | 0,021482 | 1,042105 | 0,883612 | 1 | 1 | 1 |
| Pore complex | 3 | 3 | 0,021482 | 1,042105 | 0,883612 | 1 | 1 | 1 |
| Interleukin-1 receptor complex | 3 | 3 | 0,021482 | 1,042105 | 0,883612 | 1 | 1 | 1 |
| Activin responsive factor complex | 3 | 3 | 0,021482 | 1,042105 | 0,883612 | 1 | 1 | 1 |
| U4/U6 snRNP | 3 | 3 | 0,021482 | 1,042105 | 0,883612 | 1 | 1 | 1 |
| Extrinsic to external side of plasma membrane | 3 | 3 | 0,021482 | 1,042105 | 0,883612 | 1 | 1 | 1 |
| Intrinsic to internal side of plasma membrane | 3 | 3 | 0,021482 | 1,042105 | 0,883612 | 1 | 1 | 1 |
| Anchored to external side of plasma membrane | 3 | 3 | 0,021482 | 1,042105 | 0,883612 | 1 | 1 | 1 |
| Platelet dense tubular network membrane | 3 | 3 | 0,021482 | 1,042105 | 0,883612 | 1 | 1 | 1 |
| Apical junction complex | 3 | 3 | 0,021482 | 1,042105 | 0,883612 | 1 | 1 | 1 |
| Exosome (RNase complex) | 3 | 3 | 0,021482 | 1,042105 | 0,883612 | 1 | 1 | 1 |
| RNA-induced silencing complex | 3 | 3 | 0,021482 | 1,042105 | 0,883612 | 1 | 1 | 1 |
| Alpha DNA polymerase:primase complex | 3 | 3 | 0,021482 | 1,042105 | 0,883612 | 1 | 1 | 1 |
| Clathrin coat of trans-Golgi network vesicle | 3 | 3 | 0,021482 | 1,042105 | 0,883612 | 1 | 1 | 1 |
| Nonmotile primary cilium | 3 | 3 | 0,021482 | 1,042105 | 0,883612 | 1 | 1 | 1 |
| Laminin-10 complex | 3 | 3 | 0,021482 | 1,042105 | 0,883612 | 1 | 1 | 1 |
| Alphav-beta3 integrin-vitronectin complex | 3 | 3 | 0,021482 | 1,042105 | 0,883612 | 1 | 1 | 1 |
| U2 snRNP | 3 | 3 | 0,021482 | 1,042105 | 0,883612 | 1 | 1 | 1 |
| Interleukin-6 receptor complex | 3 | 3 | 0,021482 | 1,042105 | 0,883612 | 1 | 1 | 1 |
| Lamin fiLament | 3 | 3 | 0,021482 | 1,042105 | 0,883612 | 1 | 1 | 1 |
| Golgi trans cisterna | 3 | 3 | 0,021482 | 1,042105 | 0,883612 | 1 | 1 | 1 |
| Inhibin-betaglycan-ActRII complex | 3 | 3 | 0,021482 | 1,042105 | 0,883612 | 1 | 1 | 1 |
| Chromosome passenger complex | 3 | 3 | 0,021482 | 1,042105 | 0,883612 | 1 | 1 | 1 |
| Exocytic vesicle | 3 | 3 | 0,021482 | 1,042105 | 0,883612 | 1 | 1 | 1 |
| BLOC-1 complex | 3 | 3 | 0,021482 | 1,042105 | 0,883612 | 1 | 1 | 1 |
| APC-Axin-1-beta-catenin complex | 3 | 3 | 0,021482 | 1,042105 | 0,883612 | 1 | 1 | 1 |
| DNA heterochromatin | 3 | 3 | 0,021482 | 1,042105 | 0,883612 | 1 | 1 | 1 |
| Nuclear inclusion body | 3 | 3 | 0,021482 | 1,042105 | 0,883612 | 1 | 1 | 1 |
| Interleukin-28 receptor complex | 3 | 3 | 0,021482 | 1,042105 | 0,883612 | 1 | 1 | 1 |
| Integral to mitochondrial membrane | 3 | 3 | 0,021482 | 1,042105 | 0,883612 | 1 | 1 | 1 |
| Transcription factor TFIIA complex | 3 | 3 | 0,021482 | 1,042105 | 0,883612 | 1 | 1 | 1 |
| I-kappaB/NF-kappaB complex | 3 | 3 | 0,021482 | 1,042105 | 0,883612 | 1 | 1 | 1 |
| Intrinsic to Golgi membrane | 3 | 3 | 0,021482 | 1,042105 | 0,883612 | 1 | 1 | 1 |
| Clathrin coat | 3 | 3 | 0,021482 | 1,042105 | 0,883612 | 1 | 1 | 1 |
| Laminin-1 complex | 3 | 3 | 0,021482 | 1,042105 | 0,883612 | 1 | 1 | 1 |
| Eukaryotic translation elongation factor 1 complex | 3 | 3 | 0,021482 | 1,042105 | 0,883612 | 1 | 1 | 1 |
| Integral to mitochondrial inner membrane | 3 | 3 | 0,021482 | 1,042105 | 0,883612 | 1 | 1 | 1 |
| Cohesin complex | 3 | 3 | 0,021482 | 1,042105 | 0,883612 | 1 | 1 | 1 |
| Fibril | 3 | 3 | 0,021482 | 1,042105 | 0,883612 | 1 | 1 | 1 |
| Outer kinetochore of condensed nuclear chromosome | 3 | 3 | 0,021482 | 1,042105 | 0,883612 | 1 | 1 | 1 |
| Nuclear part | 3 | 3 | 0,021482 | 1,042105 | 0,883612 | 1 | 1 | 1 |
| Glycosylphosphatidylinositol-N-acetylglucosaminyltransferase (GPI-GnT) complex | 3 | 3 | 0,021482 | 1,042105 | 0,883612 | 1 | 1 | 1 |
| Autophagic vacuole membrane | 3 | 3 | 0,021482 | 1,042105 | 0,883612 | 1 | 1 | 1 |
| CBM complex | 3 | 3 | 0,021482 | 1,042105 | 0,883612 | 1 | 1 | 1 |
| Replication fork | 3 | 3 | 0,021482 | 1,042105 | 0,883612 | 1 | 1 | 1 |
| Sarcoplasm | 3 | 3 | 0,021482 | 1,042105 | 0,883612 | 1 | 1 | 1 |
| Trans-Golgi network membrane | 3 | 3 | 0,021482 | 1,042105 | 0,883612 | 1 | 1 | 1 |
| Spindle pole centrosome | 3 | 3 | 0,021482 | 1,042105 | 0,883612 | 1 | 1 | 1 |
| Actomyosin contractile ring | 3 | 3 | 0,021482 | 1,042105 | 0,883612 | 1 | 1 | 1 |
| TAP complex | 3 | 3 | 0,021482 | 1,042105 | 0,883612 | 1 | 1 | 1 |
| Slx1-Slx4 complex | 3 | 3 | 0,021482 | 1,042105 | 0,883612 | 1 | 1 | 1 |
| Extrinsic to plasma membrane | 3 | 3 | 0,021482 | 1,042105 | 0,883612 | 1 | 1 | 1 |
| Coated vesicle | 3 | 3 | 0,021482 | 1,042105 | 0,883612 | 1 | 1 | 1 |
| Clathrin sculpted acetylcholine transport vesicle membrane | 3 | 3 | 0,021482 | 1,042105 | 0,883612 | 1 | 1 | 1 |
| Collagen type IX | 3 | 3 | 0,021482 | 1,042105 | 0,883612 | 1 | 1 | 1 |
| Chromaffin granule | 3 | 3 | 0,021482 | 1,042105 | 0,883612 | 1 | 1 | 1 |
| Vesicular fraction | 3 | 3 | 0,021482 | 1,042105 | 0,883612 | 1 | 1 | 1 |
| PeBoW complex | 3 | 3 | 0,021482 | 1,042105 | 0,883612 | 1 | 1 | 1 |
| Intrinsic to membrane | 3 | 3 | 0,021482 | 1,042105 | 0,883612 | 1 | 1 | 1 |
| Dolichol-phosphate-mannose synthase complex | 3 | 3 | 0,021482 | 1,042105 | 0,883612 | 1 | 1 | 1 |
| ULK1-ATG13-FIP200 complex | 3 | 3 | 0,021482 | 1,042105 | 0,883612 | 1 | 1 | 1 |
| Clathrin-coated endocytic vesicle | 3 | 3 | 0,021482 | 1,042105 | 0,883612 | 1 | 1 | 1 |
| TORC1 complex | 3 | 3 | 0,021482 | 1,042105 | 0,883612 | 1 | 1 | 1 |
| Laminin-11 complex | 3 | 3 | 0,021482 | 1,042105 | 0,883612 | 1 | 1 | 1 |
| Signalosome | 3 | 3 | 0,021482 | 1,042105 | 0,883612 | 1 | 1 | 1 |
| Heterochromatin | 3 | 3 | 0,021482 | 1,042105 | 0,883612 | 1 | 1 | 1 |
| Nucleotide-excision repair complex | 3 | 3 | 0,021482 | 1,042105 | 0,883612 | 1 | 1 | 1 |
| Piccolo NuA4 histone acetyltransferase complex | 3 | 3 | 0,021482 | 1,042105 | 0,883612 | 1 | 1 | 1 |
| Nuclear periphery | 3 | 3 | 0,021482 | 1,042105 | 0,883612 | 1 | 1 | 1 |
| Very-low-density lipoprotein particle | 13 | 14 | 0,09309 | 0,967722 | 0,892333 | 1 | 1 | 1 |
| Protein phosphatase type 2A complex | 12 | 13 | 0,085929 | 0,962005 | 0,905268 | 1 | 1 | 1 |
| Insoluble fraction | 12 | 13 | 0,085929 | 0,962005 | 0,905268 | 1 | 1 | 1 |
| Cell-cell adherens junction | 12 | 13 | 0,085929 | 0,962005 | 0,905268 | 1 | 1 | 1 |
| Vesicle (None) | 12 | 13 | 0,085929 | 0,962005 | 0,905268 | 1 | 1 | 1 |
| Brush border | 11 | 12 | 0,078768 | 0,955335 | 0,91771 | 1 | 1 | 1 |
| Nuclear chromosome, telomeric region | 11 | 12 | 0,078768 | 0,955335 | 0,91771 | 1 | 1 | 1 |
| PcG protein complex | 2 | 2 | 0,014322 | 1,042105 | 0,920822 | 1 | 1 | 1 |
| Plus-end kinesin complex | 2 | 2 | 0,014322 | 1,042105 | 0,920822 | 1 | 1 | 1 |
| Pre-autophagosomal structure membrane | 2 | 2 | 0,014322 | 1,042105 | 0,920822 | 1 | 1 | 1 |
| Nuclear lumen | 2 | 2 | 0,014322 | 1,042105 | 0,920822 | 1 | 1 | 1 |
| Intercalated disc | 2 | 2 | 0,014322 | 1,042105 | 0,920822 | 1 | 1 | 1 |
| Pre-autophagosomal structure | 2 | 2 | 0,014322 | 1,042105 | 0,920822 | 1 | 1 | 1 |
| Calcineurin complex | 2 | 2 | 0,014322 | 1,042105 | 0,920822 | 1 | 1 | 1 |
| BRCA1-BARD1 complex | 2 | 2 | 0,014322 | 1,042105 | 0,920822 | 1 | 1 | 1 |
| Cohesin loading complex | 2 | 2 | 0,014322 | 1,042105 | 0,920822 | 1 | 1 | 1 |
| Outer membrane | 2 | 2 | 0,014322 | 1,042105 | 0,920822 | 1 | 1 | 1 |
| Cortical microtubule cytoskeleton | 2 | 2 | 0,014322 | 1,042105 | 0,920822 | 1 | 1 | 1 |
| Filopodium membrane | 2 | 2 | 0,014322 | 1,042105 | 0,920822 | 1 | 1 | 1 |
| Annulate lamellae | 2 | 2 | 0,014322 | 1,042105 | 0,920822 | 1 | 1 | 1 |
| Clathrin-coated endocytic vesicle membrane | 2 | 2 | 0,014322 | 1,042105 | 0,920822 | 1 | 1 | 1 |
| Intracellular ferritin complex | 2 | 2 | 0,014322 | 1,042105 | 0,920822 | 1 | 1 | 1 |
| mRNA cleavage factor complex | 2 | 2 | 0,014322 | 1,042105 | 0,920822 | 1 | 1 | 1 |
| Cytoplasmic cyclin-dependent protein kinase holoenzyme complex | 2 | 2 | 0,014322 | 1,042105 | 0,920822 | 1 | 1 | 1 |
| Prefoldin complex | 2 | 2 | 0,014322 | 1,042105 | 0,920822 | 1 | 1 | 1 |
| Late endosome membrane | 2 | 2 | 0,014322 | 1,042105 | 0,920822 | 1 | 1 | 1 |
| U4 snRNP | 2 | 2 | 0,014322 | 1,042105 | 0,920822 | 1 | 1 | 1 |
| Striated myosin muscle thick filament | 2 | 2 | 0,014322 | 1,042105 | 0,920822 | 1 | 1 | 1 |
| Microtubule-based flagellum | 2 | 2 | 0,014322 | 1,042105 | 0,920822 | 1 | 1 | 1 |
| RSF complex | 2 | 2 | 0,014322 | 1,042105 | 0,920822 | 1 | 1 | 1 |
| Syntrophin complex | 2 | 2 | 0,014322 | 1,042105 | 0,920822 | 1 | 1 | 1 |
| Ku70:Ku80 complex | 2 | 2 | 0,014322 | 1,042105 | 0,920822 | 1 | 1 | 1 |
| SPOTS complex | 2 | 2 | 0,014322 | 1,042105 | 0,920822 | 1 | 1 | 1 |
| Eukaryotic translation initiation factor 2 complex | 2 | 2 | 0,014322 | 1,042105 | 0,920822 | 1 | 1 | 1 |
| PR-DUB complex | 2 | 2 | 0,014322 | 1,042105 | 0,920822 | 1 | 1 | 1 |
| Mitochondrial part | 2 | 2 | 0,014322 | 1,042105 | 0,920822 | 1 | 1 | 1 |
| DNA ligase IV complex | 2 | 2 | 0,014322 | 1,042105 | 0,920822 | 1 | 1 | 1 |
| Weibel-Palade body | 2 | 2 | 0,014322 | 1,042105 | 0,920822 | 1 | 1 | 1 |
| Phosphorylase kinase complex | 2 | 2 | 0,014322 | 1,042105 | 0,920822 | 1 | 1 | 1 |
| Integral to synaptic vesicle membrane | 2 | 2 | 0,014322 | 1,042105 | 0,920822 | 1 | 1 | 1 |
| Fibrillar collagen | 2 | 2 | 0,014322 | 1,042105 | 0,920822 | 1 | 1 | 1 |
| U6 snRNP | 2 | 2 | 0,014322 | 1,042105 | 0,920822 | 1 | 1 | 1 |
| Striated muscle thick filament | 2 | 2 | 0,014322 | 1,042105 | 0,920822 | 1 | 1 | 1 |
| Nuclear exosome (RNase complex) | 2 | 2 | 0,014322 | 1,042105 | 0,920822 | 1 | 1 | 1 |
| Complement component C1 complex | 2 | 2 | 0,014322 | 1,042105 | 0,920822 | 1 | 1 | 1 |
| Tubulin complex | 2 | 2 | 0,014322 | 1,042105 | 0,920822 | 1 | 1 | 1 |
| Toll-like receptor 1-Toll-like receptor 2 protein complex | 2 | 2 | 0,014322 | 1,042105 | 0,920822 | 1 | 1 | 1 |
| Nuclear telomeric heterochromatin | 2 | 2 | 0,014322 | 1,042105 | 0,920822 | 1 | 1 | 1 |
| PCNA-p21 complex | 2 | 2 | 0,014322 | 1,042105 | 0,920822 | 1 | 1 | 1 |
| Activin receptor complex | 2 | 2 | 0,014322 | 1,042105 | 0,920822 | 1 | 1 | 1 |
| Protein phosphatase type 1 complex | 2 | 2 | 0,014322 | 1,042105 | 0,920822 | 1 | 1 | 1 |
| Nuclear origin of replication recognition complex | 2 | 2 | 0,014322 | 1,042105 | 0,920822 | 1 | 1 | 1 |
| Laminin-3 complex | 2 | 2 | 0,014322 | 1,042105 | 0,920822 | 1 | 1 | 1 |
| Cell body fiber | 2 | 2 | 0,014322 | 1,042105 | 0,920822 | 1 | 1 | 1 |
| ESCRT III complex | 2 | 2 | 0,014322 | 1,042105 | 0,920822 | 1 | 1 | 1 |
| Collagen type VI | 2 | 2 | 0,014322 | 1,042105 | 0,920822 | 1 | 1 | 1 |
| Bleb | 2 | 2 | 0,014322 | 1,042105 | 0,920822 | 1 | 1 | 1 |
| Cilium membrane | 2 | 2 | 0,014322 | 1,042105 | 0,920822 | 1 | 1 | 1 |
| DNA-directed RNA polymerase I complex | 2 | 2 | 0,014322 | 1,042105 | 0,920822 | 1 | 1 | 1 |
| Ino80 complex | 2 | 2 | 0,014322 | 1,042105 | 0,920822 | 1 | 1 | 1 |
| Membrane attack complex | 2 | 2 | 0,014322 | 1,042105 | 0,920822 | 1 | 1 | 1 |
| Synaptic vesicle membrane | 2 | 2 | 0,014322 | 1,042105 | 0,920822 | 1 | 1 | 1 |
| Interleukin-12 complex | 2 | 2 | 0,014322 | 1,042105 | 0,920822 | 1 | 1 | 1 |
| MutSbeta complex | 2 | 2 | 0,014322 | 1,042105 | 0,920822 | 1 | 1 | 1 |
| Intracellular cyclic nucleotide activated cation channel complex | 2 | 2 | 0,014322 | 1,042105 | 0,920822 | 1 | 1 | 1 |
| Collagen type I | 2 | 2 | 0,014322 | 1,042105 | 0,920822 | 1 | 1 | 1 |
| SMN complex | 2 | 2 | 0,014322 | 1,042105 | 0,920822 | 1 | 1 | 1 |
| Collagen type XI | 2 | 2 | 0,014322 | 1,042105 | 0,920822 | 1 | 1 | 1 |
| MutSalpha complex | 2 | 2 | 0,014322 | 1,042105 | 0,920822 | 1 | 1 | 1 |
| Acrosomal matrix | 2 | 2 | 0,014322 | 1,042105 | 0,920822 | 1 | 1 | 1 |
| Tertiary granule | 2 | 2 | 0,014322 | 1,042105 | 0,920822 | 1 | 1 | 1 |
| Golgi cisterna membrane | 2 | 2 | 0,014322 | 1,042105 | 0,920822 | 1 | 1 | 1 |
| Ribonuclease MRP complex | 2 | 2 | 0,014322 | 1,042105 | 0,920822 | 1 | 1 | 1 |
| Cytosolic ribosome | 2 | 2 | 0,014322 | 1,042105 | 0,920822 | 1 | 1 | 1 |
| Lamellar body | 2 | 2 | 0,014322 | 1,042105 | 0,920822 | 1 | 1 | 1 |
| Platelet dense tubular network | 2 | 2 | 0,014322 | 1,042105 | 0,920822 | 1 | 1 | 1 |
| Beta-catenin-TCF7L2 complex | 2 | 2 | 0,014322 | 1,042105 | 0,920822 | 1 | 1 | 1 |
| Neuromuscular junction | 2 | 2 | 0,014322 | 1,042105 | 0,920822 | 1 | 1 | 1 |
| Phosphoinositide 3-kinase complex | 2 | 2 | 0,014322 | 1,042105 | 0,920822 | 1 | 1 | 1 |
| Intracellular organelle | 2 | 2 | 0,014322 | 1,042105 | 0,920822 | 1 | 1 | 1 |
| Micro-ribonucleoprotein complex | 2 | 2 | 0,014322 | 1,042105 | 0,920822 | 1 | 1 | 1 |
| Inhibin A complex | 2 | 2 | 0,014322 | 1,042105 | 0,920822 | 1 | 1 | 1 |
| Insulin receptor complex | 2 | 2 | 0,014322 | 1,042105 | 0,920822 | 1 | 1 | 1 |
| Bcl3/NF-kappaB2 complex | 2 | 2 | 0,014322 | 1,042105 | 0,920822 | 1 | 1 | 1 |
| Preribosome, large subunit Precursor | 2 | 2 | 0,014322 | 1,042105 | 0,920822 | 1 | 1 | 1 |
| SREBP-SCAP-Insig complex | 2 | 2 | 0,014322 | 1,042105 | 0,920822 | 1 | 1 | 1 |
| Mitochondrial crista | 2 | 2 | 0,014322 | 1,042105 | 0,920822 | 1 | 1 | 1 |
| Peripheral to membrane of membrane fraction | 2 | 2 | 0,014322 | 1,042105 | 0,920822 | 1 | 1 | 1 |
| TORC2 complex | 2 | 2 | 0,014322 | 1,042105 | 0,920822 | 1 | 1 | 1 |
| M band | 2 | 2 | 0,014322 | 1,042105 | 0,920822 | 1 | 1 | 1 |
| Dynein complex | 2 | 2 | 0,014322 | 1,042105 | 0,920822 | 1 | 1 | 1 |
| Lamellipodium membrane | 2 | 2 | 0,014322 | 1,042105 | 0,920822 | 1 | 1 | 1 |
| UBC13-MMS2 complex | 2 | 2 | 0,014322 | 1,042105 | 0,920822 | 1 | 1 | 1 |
| Myosin | 2 | 2 | 0,014322 | 1,042105 | 0,920822 | 1 | 1 | 1 |
| Golgi cisterna | 2 | 2 | 0,014322 | 1,042105 | 0,920822 | 1 | 1 | 1 |
| Angiogenin-PRI complex | 2 | 2 | 0,014322 | 1,042105 | 0,920822 | 1 | 1 | 1 |
| Plasma membrane part | 2 | 2 | 0,014322 | 1,042105 | 0,920822 | 1 | 1 | 1 |
| Tubulin | 2 | 2 | 0,014322 | 1,042105 | 0,920822 | 1 | 1 | 1 |
| Secreted (None) | 2 | 2 | 0,014322 | 1,042105 | 0,920822 | 1 | 1 | 1 |
| Cell | 2 | 2 | 0,014322 | 1,042105 | 0,920822 | 1 | 1 | 1 |
| Microvillus | 10 | 11 | 0,071608 | 0,947454 | 0,929593 | 1 | 1 | 1 |
| Transcription elongation factor complex | 10 | 11 | 0,071608 | 0,947454 | 0,929593 | 1 | 1 | 1 |
| Basolateral membrane | 8 | 9 | 0,057286 | 0,926444 | 0,951407 | 1 | 1 | 1 |
| Methionine adenosyltransferase complex | 1 | 1 | 0,007161 | 1,042105 | 0,959596 | 1 | 1 | 1 |
| Nucleolus organizer region | 1 | 1 | 0,007161 | 1,042105 | 0,959596 | 1 | 1 | 1 |
| Nuclear envelope lumen | 1 | 1 | 0,007161 | 1,042105 | 0,959596 | 1 | 1 | 1 |
| Centromeric heterochromatin | 1 | 1 | 0,007161 | 1,042105 | 0,959596 | 1 | 1 | 1 |
| Molybdopterin synthase complex | 1 | 1 | 0,007161 | 1,042105 | 0,959596 | 1 | 1 | 1 |
| Apical cortex | 1 | 1 | 0,007161 | 1,042105 | 0,959596 | 1 | 1 | 1 |
| Mitochondrial permeability transition pore complex | 1 | 1 | 0,007161 | 1,042105 | 0,959596 | 1 | 1 | 1 |
| Collagen type III | 1 | 1 | 0,007161 | 1,042105 | 0,959596 | 1 | 1 | 1 |
| BRCA2-MAGE-D1 complex | 1 | 1 | 0,007161 | 1,042105 | 0,959596 | 1 | 1 | 1 |
| Uropod | 1 | 1 | 0,007161 | 1,042105 | 0,959596 | 1 | 1 | 1 |
| Granular component | 1 | 1 | 0,007161 | 1,042105 | 0,959596 | 1 | 1 | 1 |
| U1 snRNP | 1 | 1 | 0,007161 | 1,042105 | 0,959596 | 1 | 1 | 1 |
| Cul2-RING ubiquitin ligase complex | 1 | 1 | 0,007161 | 1,042105 | 0,959596 | 1 | 1 | 1 |
| Paraferritin complex | 1 | 1 | 0,007161 | 1,042105 | 0,959596 | 1 | 1 | 1 |
| Dendritic shaft | 1 | 1 | 0,007161 | 1,042105 | 0,959596 | 1 | 1 | 1 |
| Lysosomal lumen | 1 | 1 | 0,007161 | 1,042105 | 0,959596 | 1 | 1 | 1 |
| Photoreceptor connecting cilium | 1 | 1 | 0,007161 | 1,042105 | 0,959596 | 1 | 1 | 1 |
| Granulocyte macrophage colony-stimulating factor receptor complex | 1 | 1 | 0,007161 | 1,042105 | 0,959596 | 1 | 1 | 1 |
| Mitochondrial pyruvate dehydrogenase complex | 1 | 1 | 0,007161 | 1,042105 | 0,959596 | 1 | 1 | 1 |
| Collagen type II | 1 | 1 | 0,007161 | 1,042105 | 0,959596 | 1 | 1 | 1 |
| AMP-activated protein kinase complex | 1 | 1 | 0,007161 | 1,042105 | 0,959596 | 1 | 1 | 1 |
| Vacuolar proton-transporting V-type ATPase, V1 domain | 1 | 1 | 0,007161 | 1,042105 | 0,959596 | 1 | 1 | 1 |
| Mitochondrial sorting and assembly machinery complex | 1 | 1 | 0,007161 | 1,042105 | 0,959596 | 1 | 1 | 1 |
| Activin A complex | 1 | 1 | 0,007161 | 1,042105 | 0,959596 | 1 | 1 | 1 |
| Gamma-secretase complex | 1 | 1 | 0,007161 | 1,042105 | 0,959596 | 1 | 1 | 1 |
| Nucleocytoplasmic shuttling complex | 1 | 1 | 0,007161 | 1,042105 | 0,959596 | 1 | 1 | 1 |
| Intrinsic to endosome membrane | 1 | 1 | 0,007161 | 1,042105 | 0,959596 | 1 | 1 | 1 |
| Microtubule plus end | 1 | 1 | 0,007161 | 1,042105 | 0,959596 | 1 | 1 | 1 |
| Preribosome | 1 | 1 | 0,007161 | 1,042105 | 0,959596 | 1 | 1 | 1 |
| Laminin-2 complex | 1 | 1 | 0,007161 | 1,042105 | 0,959596 | 1 | 1 | 1 |
| Dendrite cytoplasm | 1 | 1 | 0,007161 | 1,042105 | 0,959596 | 1 | 1 | 1 |
| Astral microtubule | 1 | 1 | 0,007161 | 1,042105 | 0,959596 | 1 | 1 | 1 |
| Juxtaparanode region of axon | 1 | 1 | 0,007161 | 1,042105 | 0,959596 | 1 | 1 | 1 |
| Collagen type XIV | 1 | 1 | 0,007161 | 1,042105 | 0,959596 | 1 | 1 | 1 |
| Neurofilament cytoskeleton | 1 | 1 | 0,007161 | 1,042105 | 0,959596 | 1 | 1 | 1 |
| Mitochondrial endopeptidase Clp complex | 1 | 1 | 0,007161 | 1,042105 | 0,959596 | 1 | 1 | 1 |
| Isoamylase complex | 1 | 1 | 0,007161 | 1,042105 | 0,959596 | 1 | 1 | 1 |
| Photoreceptor outer segment membrane | 1 | 1 | 0,007161 | 1,042105 | 0,959596 | 1 | 1 | 1 |
| Actomyosin, actin part | 1 | 1 | 0,007161 | 1,042105 | 0,959596 | 1 | 1 | 1 |
| External side of mitochondrial outer membrane | 1 | 1 | 0,007161 | 1,042105 | 0,959596 | 1 | 1 | 1 |
| MHC class I peptide loading complex | 1 | 1 | 0,007161 | 1,042105 | 0,959596 | 1 | 1 | 1 |
| Ribonucleoside-diphosphate reductase complex | 1 | 1 | 0,007161 | 1,042105 | 0,959596 | 1 | 1 | 1 |
| H zone | 1 | 1 | 0,007161 | 1,042105 | 0,959596 | 1 | 1 | 1 |
| Golgi medial cisterna | 1 | 1 | 0,007161 | 1,042105 | 0,959596 | 1 | 1 | 1 |
| Chylomicron remnant | 1 | 1 | 0,007161 | 1,042105 | 0,959596 | 1 | 1 | 1 |
| Catenin-TCF7L2 complex | 1 | 1 | 0,007161 | 1,042105 | 0,959596 | 1 | 1 | 1 |
| Perichromatin fibrils | 1 | 1 | 0,007161 | 1,042105 | 0,959596 | 1 | 1 | 1 |
| Ornithine carbamoyltransferase complex | 1 | 1 | 0,007161 | 1,042105 | 0,959596 | 1 | 1 | 1 |
| Condensed nuclear chromosome kinetochore | 1 | 1 | 0,007161 | 1,042105 | 0,959596 | 1 | 1 | 1 |
| Melanosome membrane | 1 | 1 | 0,007161 | 1,042105 | 0,959596 | 1 | 1 | 1 |
| Extracellular vesicular exosome | 1 | 1 | 0,007161 | 1,042105 | 0,959596 | 1 | 1 | 1 |
| Interleukin-13 receptor complex | 1 | 1 | 0,007161 | 1,042105 | 0,959596 | 1 | 1 | 1 |
| Leading edge membrane | 1 | 1 | 0,007161 | 1,042105 | 0,959596 | 1 | 1 | 1 |
| CAAX-protein geranylgeranyltransferase complex | 1 | 1 | 0,007161 | 1,042105 | 0,959596 | 1 | 1 | 1 |
| T-tubule | 1 | 1 | 0,007161 | 1,042105 | 0,959596 | 1 | 1 | 1 |
| Glycocalyx | 1 | 1 | 0,007161 | 1,042105 | 0,959596 | 1 | 1 | 1 |
| Hydrogen:potassium-exchanging ATPase complex | 1 | 1 | 0,007161 | 1,042105 | 0,959596 | 1 | 1 | 1 |
| Transcription factor TFIIF complex | 1 | 1 | 0,007161 | 1,042105 | 0,959596 | 1 | 1 | 1 |
| Membrane part | 1 | 1 | 0,007161 | 1,042105 | 0,959596 | 1 | 1 | 1 |
| U4atac snRNP | 1 | 1 | 0,007161 | 1,042105 | 0,959596 | 1 | 1 | 1 |
| Multivesicular body membrane | 1 | 1 | 0,007161 | 1,042105 | 0,959596 | 1 | 1 | 1 |
| Rab-protein geranylgeranyltransferase complex | 1 | 1 | 0,007161 | 1,042105 | 0,959596 | 1 | 1 | 1 |
| Type III intermediate filament | 1 | 1 | 0,007161 | 1,042105 | 0,959596 | 1 | 1 | 1 |
| Aminoacyl-tRNA synthetase multienzyme complex | 1 | 1 | 0,007161 | 1,042105 | 0,959596 | 1 | 1 | 1 |
| Extracellular matrix part | 1 | 1 | 0,007161 | 1,042105 | 0,959596 | 1 | 1 | 1 |
| C zone | 1 | 1 | 0,007161 | 1,042105 | 0,959596 | 1 | 1 | 1 |
| Cytoplasmic ubiquitin ligase complex | 1 | 1 | 0,007161 | 1,042105 | 0,959596 | 1 | 1 | 1 |
| Subapical complex | 1 | 1 | 0,007161 | 1,042105 | 0,959596 | 1 | 1 | 1 |
| Protein serine/threonine phosphatase complex | 1 | 1 | 0,007161 | 1,042105 | 0,959596 | 1 | 1 | 1 |
| Laminin-8 complex | 1 | 1 | 0,007161 | 1,042105 | 0,959596 | 1 | 1 | 1 |
| Outer dense fiber | 1 | 1 | 0,007161 | 1,042105 | 0,959596 | 1 | 1 | 1 |
| Gamma-tubulin small complex | 1 | 1 | 0,007161 | 1,042105 | 0,959596 | 1 | 1 | 1 |
| Intrinsic to endoplasmic reticulum membrane | 1 | 1 | 0,007161 | 1,042105 | 0,959596 | 1 | 1 | 1 |
| Nuclear euchromatin | 1 | 1 | 0,007161 | 1,042105 | 0,959596 | 1 | 1 | 1 |
| Actomyosin | 1 | 1 | 0,007161 | 1,042105 | 0,959596 | 1 | 1 | 1 |
| Fascia adherens | 1 | 1 | 0,007161 | 1,042105 | 0,959596 | 1 | 1 | 1 |
| U2-type spliceosomal complex | 1 | 1 | 0,007161 | 1,042105 | 0,959596 | 1 | 1 | 1 |
| Collagen type VII | 1 | 1 | 0,007161 | 1,042105 | 0,959596 | 1 | 1 | 1 |
| Platelet dense granule | 1 | 1 | 0,007161 | 1,042105 | 0,959596 | 1 | 1 | 1 |
| DNA polymerase III complex | 1 | 1 | 0,007161 | 1,042105 | 0,959596 | 1 | 1 | 1 |
| tRNA-intron endonuclease complex | 1 | 1 | 0,007161 | 1,042105 | 0,959596 | 1 | 1 | 1 |
| Bcl3-Bcl10 complex | 1 | 1 | 0,007161 | 1,042105 | 0,959596 | 1 | 1 | 1 |
| ACF complex | 1 | 1 | 0,007161 | 1,042105 | 0,959596 | 1 | 1 | 1 |
| Chromatin accessibility complex | 1 | 1 | 0,007161 | 1,042105 | 0,959596 | 1 | 1 | 1 |
| Centrosomal corona | 1 | 1 | 0,007161 | 1,042105 | 0,959596 | 1 | 1 | 1 |
| Mitochondrial chromosome | 1 | 1 | 0,007161 | 1,042105 | 0,959596 | 1 | 1 | 1 |
| Euchromatin | 1 | 1 | 0,007161 | 1,042105 | 0,959596 | 1 | 1 | 1 |
| Integral to organelle membrane | 1 | 1 | 0,007161 | 1,042105 | 0,959596 | 1 | 1 | 1 |
| Trans-Golgi network transport vesicle membrane | 1 | 1 | 0,007161 | 1,042105 | 0,959596 | 1 | 1 | 1 |
| Signal peptidase complex | 1 | 1 | 0,007161 | 1,042105 | 0,959596 | 1 | 1 | 1 |
| Photoreceptor inner segment membrane | 1 | 1 | 0,007161 | 1,042105 | 0,959596 | 1 | 1 | 1 |
| Condensin core heterodimer | 1 | 1 | 0,007161 | 1,042105 | 0,959596 | 1 | 1 | 1 |
| Axolemma | 1 | 1 | 0,007161 | 1,042105 | 0,959596 | 1 | 1 | 1 |
| Collagen type XII | 1 | 1 | 0,007161 | 1,042105 | 0,959596 | 1 | 1 | 1 |
| Proteasome regulatory particle, base subcomplex | 1 | 1 | 0,007161 | 1,042105 | 0,959596 | 1 | 1 | 1 |
| Cell projection membrane | 1 | 1 | 0,007161 | 1,042105 | 0,959596 | 1 | 1 | 1 |
| Spindle pole body | 1 | 1 | 0,007161 | 1,042105 | 0,959596 | 1 | 1 | 1 |
| Cytosolic part | 1 | 1 | 0,007161 | 1,042105 | 0,959596 | 1 | 1 | 1 |
| Mature chylomicron | 1 | 1 | 0,007161 | 1,042105 | 0,959596 | 1 | 1 | 1 |
| Collagen type VIII | 1 | 1 | 0,007161 | 1,042105 | 0,959596 | 1 | 1 | 1 |
| Transcription factor TFIIIB complex | 1 | 1 | 0,007161 | 1,042105 | 0,959596 | 1 | 1 | 1 |
| Terminal button | 1 | 1 | 0,007161 | 1,042105 | 0,959596 | 1 | 1 | 1 |
| 4-aminobutyrate transaminase complex | 1 | 1 | 0,007161 | 1,042105 | 0,959596 | 1 | 1 | 1 |
| Mast cell granule | 1 | 1 | 0,007161 | 1,042105 | 0,959596 | 1 | 1 | 1 |
| Citrate lyase complex | 1 | 1 | 0,007161 | 1,042105 | 0,959596 | 1 | 1 | 1 |
| Cytoskeletal part | 1 | 1 | 0,007161 | 1,042105 | 0,959596 | 1 | 1 | 1 |
| Cytoplasmic chromatin | 1 | 1 | 0,007161 | 1,042105 | 0,959596 | 1 | 1 | 1 |
| Organelle inner membrane | 1 | 1 | 0,007161 | 1,042105 | 0,959596 | 1 | 1 | 1 |
| Invadopodium membrane | 1 | 1 | 0,007161 | 1,042105 | 0,959596 | 1 | 1 | 1 |
| Spot adherens junction | 1 | 1 | 0,007161 | 1,042105 | 0,959596 | 1 | 1 | 1 |
| Node of Ranvier | 1 | 1 | 0,007161 | 1,042105 | 0,959596 | 1 | 1 | 1 |
| Cell fraction | 1 | 1 | 0,007161 | 1,042105 | 0,959596 | 1 | 1 | 1 |
| Growth hormone receptor complex | 1 | 1 | 0,007161 | 1,042105 | 0,959596 | 1 | 1 | 1 |
| MCM complex | 1 | 1 | 0,007161 | 1,042105 | 0,959596 | 1 | 1 | 1 |
| COPI-coated vesicle | 1 | 1 | 0,007161 | 1,042105 | 0,959596 | 1 | 1 | 1 |
| Phagocytic cup | 1 | 1 | 0,007161 | 1,042105 | 0,959596 | 1 | 1 | 1 |
| Organelle membrane | 1 | 1 | 0,007161 | 1,042105 | 0,959596 | 1 | 1 | 1 |
| Stereocilium | 1 | 1 | 0,007161 | 1,042105 | 0,959596 | 1 | 1 | 1 |
| Protein kinase CK2 complex | 1 | 1 | 0,007161 | 1,042105 | 0,959596 | 1 | 1 | 1 |
| snRNA-activating protein complex | 1 | 1 | 0,007161 | 1,042105 | 0,959596 | 1 | 1 | 1 |
| CD95 death-inducing signaling complex | 1 | 1 | 0,007161 | 1,042105 | 0,959596 | 1 | 1 | 1 |
| Extrinsic to mitochondrial inner membrane | 1 | 1 | 0,007161 | 1,042105 | 0,959596 | 1 | 1 | 1 |
| Cytoplasmic exosome (RNase complex) | 1 | 1 | 0,007161 | 1,042105 | 0,959596 | 1 | 1 | 1 |
| Elongator holoenzyme complex | 1 | 1 | 0,007161 | 1,042105 | 0,959596 | 1 | 1 | 1 |
| Ciliary neurotrophic factor receptor complex | 1 | 1 | 0,007161 | 1,042105 | 0,959596 | 1 | 1 | 1 |
| Glycine cleavage complex | 1 | 1 | 0,007161 | 1,042105 | 0,959596 | 1 | 1 | 1 |
| Nuclear cyclin-dependent protein kinase holoenzyme complex | 1 | 1 | 0,007161 | 1,042105 | 0,959596 | 1 | 1 | 1 |
| Collagen type XVI | 1 | 1 | 0,007161 | 1,042105 | 0,959596 | 1 | 1 | 1 |
| 6-phosphofructo-2-kinase/fructose-2,6-biphosphatase 1 complex | 1 | 1 | 0,007161 | 1,042105 | 0,959596 | 1 | 1 | 1 |
| Caspase complex | 1 | 1 | 0,007161 | 1,042105 | 0,959596 | 1 | 1 | 1 |
| Collagen type XIII | 1 | 1 | 0,007161 | 1,042105 | 0,959596 | 1 | 1 | 1 |
| Junctional membrane complex | 1 | 1 | 0,007161 | 1,042105 | 0,959596 | 1 | 1 | 1 |
| Hrd1p ubiquitin ligase complex | 1 | 1 | 0,007161 | 1,042105 | 0,959596 | 1 | 1 | 1 |
| Katanin complex | 1 | 1 | 0,007161 | 1,042105 | 0,959596 | 1 | 1 | 1 |
| Interleukin-18 receptor complex | 1 | 1 | 0,007161 | 1,042105 | 0,959596 | 1 | 1 | 1 |
| Extacellular | 1 | 1 | 0,007161 | 1,042105 | 0,959596 | 1 | 1 | 1 |
| Respiratory chain complex II | 1 | 1 | 0,007161 | 1,042105 | 0,959596 | 1 | 1 | 1 |
| Polarisome | 1 | 1 | 0,007161 | 1,042105 | 0,959596 | 1 | 1 | 1 |
| Collagen type XV | 1 | 1 | 0,007161 | 1,042105 | 0,959596 | 1 | 1 | 1 |
| VCB complex | 1 | 1 | 0,007161 | 1,042105 | 0,959596 | 1 | 1 | 1 |
| Apicolateral plasma membrane | 1 | 1 | 0,007161 | 1,042105 | 0,959596 | 1 | 1 | 1 |
| Holliday junction resolvase complex | 1 | 1 | 0,007161 | 1,042105 | 0,959596 | 1 | 1 | 1 |
| Lateral element | 1 | 1 | 0,007161 | 1,042105 | 0,959596 | 1 | 1 | 1 |
| Presynaptic active zone | 1 | 1 | 0,007161 | 1,042105 | 0,959596 | 1 | 1 | 1 |
| Clathrin sculpted glutamate transport vesicle membrane | 1 | 1 | 0,007161 | 1,042105 | 0,959596 | 1 | 1 | 1 |
| Vacuolar membrane | 1 | 1 | 0,007161 | 1,042105 | 0,959596 | 1 | 1 | 1 |
| DNA topoisomerase complex (ATP-hydrolyzing) | 1 | 1 | 0,007161 | 1,042105 | 0,959596 | 1 | 1 | 1 |
| Endosome lumen | 1 | 1 | 0,007161 | 1,042105 | 0,959596 | 1 | 1 | 1 |
| Tertiary granule membrane | 1 | 1 | 0,007161 | 1,042105 | 0,959596 | 1 | 1 | 1 |
| Rb-E2F complex | 1 | 1 | 0,007161 | 1,042105 | 0,959596 | 1 | 1 | 1 |
| Guanyl-nucleotide exchange factor complex | 1 | 1 | 0,007161 | 1,042105 | 0,959596 | 1 | 1 | 1 |
| Cohesin core heterodimer | 1 | 1 | 0,007161 | 1,042105 | 0,959596 | 1 | 1 | 1 |
| Mitochondrial respiratory chain complex II | 1 | 1 | 0,007161 | 1,042105 | 0,959596 | 1 | 1 | 1 |
| Contractile ring | 1 | 1 | 0,007161 | 1,042105 | 0,959596 | 1 | 1 | 1 |
| MutLalpha complex | 1 | 1 | 0,007161 | 1,042105 | 0,959596 | 1 | 1 | 1 |
| Viral integration complex | 1 | 1 | 0,007161 | 1,042105 | 0,959596 | 1 | 1 | 1 |
| Intercellular bridge | 1 | 1 | 0,007161 | 1,042105 | 0,959596 | 1 | 1 | 1 |
| Discoidal high-density lipoprotein particle | 1 | 1 | 0,007161 | 1,042105 | 0,959596 | 1 | 1 | 1 |
| Golgi complex | 1 | 1 | 0,007161 | 1,042105 | 0,959596 | 1 | 1 | 1 |
| Exocyst | 1 | 1 | 0,007161 | 1,042105 | 0,959596 | 1 | 1 | 1 |
| Lsd1/2 complex | 1 | 1 | 0,007161 | 1,042105 | 0,959596 | 1 | 1 | 1 |
| Chromocenter | 1 | 1 | 0,007161 | 1,042105 | 0,959596 | 1 | 1 | 1 |
| mRNA cleavage and polyadenylation specificity factor complex | 1 | 1 | 0,007161 | 1,042105 | 0,959596 | 1 | 1 | 1 |
| Nuclear condensin complex | 1 | 1 | 0,007161 | 1,042105 | 0,959596 | 1 | 1 | 1 |
| Perinucleolar chromocenter | 1 | 1 | 0,007161 | 1,042105 | 0,959596 | 1 | 1 | 1 |
| MHC class II protein complex | 1 | 1 | 0,007161 | 1,042105 | 0,959596 | 1 | 1 | 1 |
| Nascent polypeptide-associated complex | 1 | 1 | 0,007161 | 1,042105 | 0,959596 | 1 | 1 | 1 |
| ERCC4-ERCC1 complex | 1 | 1 | 0,007161 | 1,042105 | 0,959596 | 1 | 1 | 1 |
| Cell-substrate adherens junction | 1 | 1 | 0,007161 | 1,042105 | 0,959596 | 1 | 1 | 1 |
| Gamma DNA polymerase complex | 1 | 1 | 0,007161 | 1,042105 | 0,959596 | 1 | 1 | 1 |
| Nuclear centromeric heterochromatin | 1 | 1 | 0,007161 | 1,042105 | 0,959596 | 1 | 1 | 1 |
| Symbiont-containing vacuole membrane | 1 | 1 | 0,007161 | 1,042105 | 0,959596 | 1 | 1 | 1 |
| Delta DNA polymerase complex | 1 | 1 | 0,007161 | 1,042105 | 0,959596 | 1 | 1 | 1 |
| Epsilon DNA polymerase complex | 1 | 1 | 0,007161 | 1,042105 | 0,959596 | 1 | 1 | 1 |
| Glycine-gated chloride channel complex | 1 | 1 | 0,007161 | 1,042105 | 0,959596 | 1 | 1 | 1 |
| Eosinophil | 1 | 1 | 0,007161 | 1,042105 | 0,959596 | 1 | 1 | 1 |
| Sec61 translocon complex | 1 | 1 | 0,007161 | 1,042105 | 0,959596 | 1 | 1 | 1 |
| Endoplasmic reticulum Sec complex | 1 | 1 | 0,007161 | 1,042105 | 0,959596 | 1 | 1 | 1 |
| CCR4-NOT complex | 1 | 1 | 0,007161 | 1,042105 | 0,959596 | 1 | 1 | 1 |
| Extracelluler | 1 | 1 | 0,007161 | 1,042105 | 0,959596 | 1 | 1 | 1 |
| Flagellum | 1 | 1 | 0,007161 | 1,042105 | 0,959596 | 1 | 1 | 1 |
| Positive transcription elongation factor complex b | 1 | 1 | 0,007161 | 1,042105 | 0,959596 | 1 | 1 | 1 |
| Smooth microsome | 1 | 1 | 0,007161 | 1,042105 | 0,959596 | 1 | 1 | 1 |
| Centromere | 1 | 1 | 0,007161 | 1,042105 | 0,959596 | 1 | 1 | 1 |
| Large ribosomal subunit | 1 | 1 | 0,007161 | 1,042105 | 0,959596 | 1 | 1 | 1 |
| Gamma-catenin-TCF7L2 complex | 1 | 1 | 0,007161 | 1,042105 | 0,959596 | 1 | 1 | 1 |
| Clathrin coat of coated pit | 1 | 1 | 0,007161 | 1,042105 | 0,959596 | 1 | 1 | 1 |
| Interphase microtubule organizing center | 1 | 1 | 0,007161 | 1,042105 | 0,959596 | 1 | 1 | 1 |
| Scrib-APC-beta-catenin complex | 1 | 1 | 0,007161 | 1,042105 | 0,959596 | 1 | 1 | 1 |
| Cell-substrate junction | 1 | 1 | 0,007161 | 1,042105 | 0,959596 | 1 | 1 | 1 |
| Nucleus(periNuclear) | 1 | 1 | 0,007161 | 1,042105 | 0,959596 | 1 | 1 | 1 |
| Intracellular organelle part | 1 | 1 | 0,007161 | 1,042105 | 0,959596 | 1 | 1 | 1 |
| Dense-core vesicle (None) | 1 | 1 | 0,007161 | 1,042105 | 0,959596 | 1 | 1 | 1 |
| Interleukin-5 receptor complex | 1 | 1 | 0,007161 | 1,042105 | 0,959596 | 1 | 1 | 1 |
| Rough microsome | 1 | 1 | 0,007161 | 1,042105 | 0,959596 | 1 | 1 | 1 |
| Nuclei | 1 | 1 | 0,007161 | 1,042105 | 0,959596 | 1 | 1 | 1 |
| Cilium part | 1 | 1 | 0,007161 | 1,042105 | 0,959596 | 1 | 1 | 1 |
| Others(granules of neutrophils) | 1 | 1 | 0,007161 | 1,042105 | 0,959596 | 1 | 1 | 1 |
| Mannosyltransferase complex | 1 | 1 | 0,007161 | 1,042105 | 0,959596 | 1 | 1 | 1 |
| Chromosome, centromeric region | 17 | 19 | 0,121733 | 0,932468 | 0,960727 | 1 | 1 | 1 |
| Basal plasma membrane | 7 | 8 | 0,050125 | 0,912005 | 0,961185 | 1 | 1 | 1 |
| Mitochondrial large ribosomal subunit | 7 | 8 | 0,050125 | 0,912005 | 0,961185 | 1 | 1 | 1 |
| Chromosome | 54 | 59 | 0,386681 | 0,953806 | 0,968748 | 1 | 1 | 1 |
| Calcium channel complex | 6 | 7 | 0,042965 | 0,893445 | 0,9701 | 1 | 1 | 1 |
| Chromosome, telomeric region | 15 | 17 | 0,107411 | 0,919577 | 0,970756 | 1 | 1 | 1 |
| Low-density lipoprotein particle | 5 | 6 | 0,035804 | 0,86871 | 0,978062 | 1 | 1 | 1 |
| Aggresome | 5 | 6 | 0,035804 | 0,86871 | 0,978062 | 1 | 1 | 1 |
| Integral to mitochondrial outer membrane | 5 | 6 | 0,035804 | 0,86871 | 0,978062 | 1 | 1 | 1 |
| Mitochondrial outer membrane translocase complex | 5 | 6 | 0,035804 | 0,86871 | 0,978062 | 1 | 1 | 1 |
| FHF complex | 4 | 5 | 0,028643 | 0,8341 | 0,984975 | 1 | 1 | 1 |
| DNA-directed RNA polymerase II, holoenzyme | 4 | 5 | 0,028643 | 0,8341 | 0,984975 | 1 | 1 | 1 |
| Mre11 complex | 4 | 5 | 0,028643 | 0,8341 | 0,984975 | 1 | 1 | 1 |
| MMXD complex | 4 | 5 | 0,028643 | 0,8341 | 0,984975 | 1 | 1 | 1 |
| Integrator complex | 11 | 13 | 0,078768 | 0,881905 | 0,986139 | 1 | 1 | 1 |
| Nucleus | 5585 | 5847 | 39,99284 | 0,995409 | 0,987633 | 1 | 1 | 1 |
| Exosomes | 1942 | 2043 | 13,90619 | 0,990587 | 0,98764 | 1 | 1 | 1 |
| DNA-dependent protein kinase-DNA ligase 4 complex | 3 | 4 | 0,021482 | 0,782229 | 0,990738 | 1 | 1 | 1 |
| Exon-exon junction complex | 3 | 4 | 0,021482 | 0,782229 | 0,990738 | 1 | 1 | 1 |
| Unknown membrane (None) | 3 | 4 | 0,021482 | 0,782229 | 0,990738 | 1 | 1 | 1 |
| Mitochondrial respiratory chain complex III | 2 | 3 | 0,014322 | 0,695891 | 0,995242 | 1 | 1 | 1 |
| Ubiquitin conjugating enzyme complex | 2 | 3 | 0,014322 | 0,695891 | 0,995242 | 1 | 1 | 1 |
| Condensed chromosome, centromeric region | 2 | 3 | 0,014322 | 0,695891 | 0,995242 | 1 | 1 | 1 |
| Stress fiber | 13 | 16 | 0,09309 | 0,846833 | 0,996744 | 1 | 1 | 1 |
| BRCA1-A complex | 5 | 7 | 0,035804 | 0,744786 | 0,997967 | 1 | 1 | 1 |
| Hemidesmosome | 1 | 2 | 0,007161 | 0,523645 | 0,99837 | 1 | 1 | 1 |
| Nuclear ubiquitin ligase complex | 1 | 2 | 0,007161 | 0,523645 | 0,99837 | 1 | 1 | 1 |
| Signal recognition particle receptor complex | 1 | 2 | 0,007161 | 0,523645 | 0,99837 | 1 | 1 | 1 |
| ER to Golgi transport vesicle | 1 | 2 | 0,007161 | 0,523645 | 0,99837 | 1 | 1 | 1 |
| Mitochondrial matrix | 70 | 79 | 0,501253 | 0,923399 | 0,998744 | 1 | 1 | 1 |
| Adherens junction | 4 | 6 | 0,028643 | 0,695315 | 0,998802 | 1 | 1 | 1 |
| Mitochondrial respiratory chain complex I | 39 | 46 | 0,27927 | 0,883558 | 0,999546 | 1 | 1 | 1 |
| BRISC complex | 2 | 4 | 0,014322 | 0,522352 | 0,999745 | 1 | 1 | 1 |
| Fanconi anaemia nuclear complex | 2 | 4 | 0,014322 | 0,522352 | 0,999745 | 1 | 1 | 1 |
| Soluble fraction | 162 | 180 | 1,160043 | 0,9379 | 0,999874 | 1 | 1 | 1 |
| Mitochondrial membrane | 47 | 56 | 0,336556 | 0,874654 | 0,999929 | 1 | 1 | 1 |
| Mitochondrion | 1181 | 1259 | 8,456856 | 0,977543 | 0,99995 | 1 | 1 | 1 |
| Centrosome | 608 | 656 | 4,353741 | 0,965855 | 0,999976 | 1 | 1 | 1 |
| Mitochondrial inner membrane | 47 | 58 | 0,336556 | 0,844499 | 0,999997 | 1 | 1 | 1 |
| Nucleolus | 1170 | 1257 | 8,378088 | 0,969979 | 1 | 1 | 1 | 1 |
| WASH complex | 2 | 7 | 0,014322 | 0,298806 | 1 | 1 | 1 | 1 |
| Nucleosome | 2 | 44 | 0,014322 | 0,047594 | 1 | 1 | 1 | 1 |

Table S7. miRNAs classification by molecular function.

| Molecular function | No. of genes in the dataset | No. of genes in the background dataset | Percentage of genes | Fold enrichment | P-value (Hypergeometric test) | Bonferroni method | BH method | Q-value (Storey-Tibshirani method) |
| --- | --- | --- | --- | --- | --- | --- | --- | --- |
| Transcription factor activity | 832 | 842 | 4,849898 | 1,043995 | 7,56E-11 | 1,69E-08 | 1,69E-08 | 5,31432E-08 |
| G-protein coupled receptor activity | 727 | 736 | 4,237832 | 1,043624 | 1,94E-09 | 4,35E-07 | 2,17E-07 | 6,8226E-07 |
| Catalytic activity | 524 | 532 | 3,054503 | 1,040656 | 3,29E-06 | 0,000736 | 0,000245 | 0,000770336 |
| Cytoskeletal protein binding | 218 | 218 | 1,270767 | 1,056543 | 5,76E-06 | 0,00129 | 0,000322 | 0,001012057 |
| Structural molecule activity | 267 | 269 | 1,556398 | 1,048688 | 4,53E-05 | 0,010148 | 0,00203 | 0,006370361 |
| Transcription regulator activity | 810 | 832 | 4,721655 | 1,028606 | 7,74E-05 | 0,017334 | 0,002889 | 0,009067684 |
| Structural constituent of ribosome | 152 | 152 | 0,886039 | 1,056543 | 0,000226 | 0,050552 | 0,0066 | 0,020715532 |
| RNA binding | 360 | 366 | 2,098514 | 1,039223 | 0,000236 | 0,052799 | 0,0066 | 0,020715532 |
| GTPase activator activity | 144 | 144 | 0,839405 | 1,056543 | 0,000352 | 0,078787 | 0,008754 | 0,027476964 |
| Receptor binding | 129 | 129 | 0,751967 | 1,056543 | 0,000808 | 0,180945 | 0,016713 | 0,052457105 |
| Auxiliary transport protein activity | 327 | 333 | 1,90615 | 1,037507 | 0,000869 | 0,194674 | 0,016713 | 0,052457105 |
| Receptor activity | 354 | 361 | 2,063538 | 1,036057 | 0,000895 | 0,200552 | 0,016713 | 0,052457105 |
| Cytokine activity | 107 | 107 | 0,623725 | 1,056543 | 0,002731 | 0,611777 | 0,043656 | 0,137027194 |
| Cell adhesion molecule activity | 348 | 356 | 2,028563 | 1,032801 | 0,002896 | 0,648754 | 0,043656 | 0,137027194 |
| Protein serine/threonine kinase activity | 295 | 301 | 1,719615 | 1,035483 | 0,002923 | 0,654846 | 0,043656 | 0,137027194 |
| Transporter activity | 559 | 576 | 3,258525 | 1,025361 | 0,003644 | 0,816245 | 0,051015 | 0,160125101 |
| Structural constituent of cytoskeleton | 136 | 137 | 0,792772 | 1,048832 | 0,004567 | 1 | 0,060179 | 0,188888463 |
| Extracellular matrix structural constituent | 164 | 166 | 0,95599 | 1,043815 | 0,005714 | 1 | 0,071107 | 0,22318901 |
| Voltage-gated ion channel activity | 129 | 130 | 0,751967 | 1,048417 | 0,006425 | 1 | 0,075742 | 0,237736018 |
| Transferase activity | 154 | 156 | 0,897697 | 1,042999 | 0,008897 | 1 | 0,099647 | 0,312767997 |
| Calcium ion binding | 182 | 185 | 1,060915 | 1,039411 | 0,009401 | 1 | 0,100276 | 0,314743237 |
| Motor activity | 79 | 79 | 0,460507 | 1,056543 | 0,012844 | 1 | 0,13078 | 0,410488987 |
| Guanyl-nucleotide exchange factor activity | 111 | 112 | 0,647042 | 1,047111 | 0,015271 | 1 | 0,148729 | 0,466824341 |
| Metallopeptidase activity | 100 | 101 | 0,58292 | 1,046083 | 0,025672 | 1 | 0,239603 | 0,752058571 |
| Receptor signaling complex scaffold activity | 312 | 322 | 1,818712 | 1,023732 | 0,038729 | 1 | 0,347016 | 1 |
| Peptidase activity | 58 | 58 | 0,338094 | 1,056543 | 0,040954 | 1 | 0,350239 | 1 |
| DNA repair protein | 57 | 57 | 0,332265 | 1,056543 | 0,043277 | 1 | 0,350239 | 1 |
| GTPase activity | 216 | 222 | 1,259108 | 1,027989 | 0,04378 | 1 | 0,350239 | 1 |
| Transmembrane receptor protein tyrosine kinase activity | 56 | 56 | 0,326435 | 1,056543 | 0,045732 | 1 | 0,353241 | 1 |
| Sulfotransferase activity | 53 | 53 | 0,308948 | 1,056543 | 0,053964 | 1 | 0,40293 | 1 |
| Transmembrane receptor activity | 52 | 52 | 0,303119 | 1,056543 | 0,057024 | 1 | 0,412047 | 1 |
| Ribonuclease activity | 48 | 48 | 0,279802 | 1,056543 | 0,071102 | 1 | 0,497711 | 1 |
| Cysteine-type peptidase activity | 46 | 46 | 0,268143 | 1,056543 | 0,079393 | 1 | 0,522004 | 1 |
| Isomerase activity | 45 | 45 | 0,262314 | 1,056543 | 0,083893 | 1 | 0,522004 | 1 |
| Peptide hormone | 45 | 45 | 0,262314 | 1,056543 | 0,083893 | 1 | 0,522004 | 1 |
| Phospholipase activity | 45 | 45 | 0,262314 | 1,056543 | 0,083893 | 1 | 0,522004 | 1 |
| Antigen binding | 42 | 42 | 0,244827 | 1,056543 | 0,098984 | 1 | 0,599255 | 1 |
| Extracellular ligand-gated ion channel activity | 41 | 41 | 0,238997 | 1,056543 | 0,104594 | 1 | 0,600747 | 1 |
| Ribonucleoprotein | 41 | 41 | 0,238997 | 1,056543 | 0,104594 | 1 | 0,600747 | 1 |
| Protease inhibitor activity | 92 | 94 | 0,536287 | 1,034066 | 0,114677 | 1 | 0,642189 | 1 |
| Protein-tyrosine kinase activity | 38 | 38 | 0,22151 | 1,056543 | 0,123404 | 1 | 0,666586 | 1 |
| Ion channel activity | 65 | 66 | 0,378898 | 1,040537 | 0,124985 | 1 | 0,666586 | 1 |
| Ligand-dependent nuclear receptor activity | 35 | 35 | 0,204022 | 1,056543 | 0,145592 | 1 | 0,741196 | 1 |
| Lipid kinase activity | 35 | 35 | 0,204022 | 1,056543 | 0,145592 | 1 | 0,741196 | 1 |
| Chaperone activity | 122 | 126 | 0,711163 | 1,023005 | 0,189158 | 1 | 0,933859 | 1 |
| Intracellular ligand-gated ion channel activity | 30 | 30 | 0,174876 | 1,056543 | 0,191775 | 1 | 0,933859 | 1 |
| DNA-directed RNA polymerase activity | 29 | 29 | 0,169047 | 1,056543 | 0,202637 | 1 | 0,945637 | 1 |
| Phosphoric diester hydrolase activity | 29 | 29 | 0,169047 | 1,056543 | 0,202637 | 1 | 0,945637 | 1 |
| Complement activity | 28 | 28 | 0,163218 | 1,056543 | 0,214113 | 1 | 0,959226 | 1 |
| Protein threonine/tyrosine kinase activity | 28 | 28 | 0,163218 | 1,056543 | 0,214113 | 1 | 0,959226 | 1 |
| Serine-type peptidase activity | 95 | 98 | 0,553774 | 1,024203 | 0,22378 | 1 | 0,974567 | 1 |
| Heat shock protein activity | 27 | 27 | 0,157389 | 1,056543 | 0,226239 | 1 | 0,974567 | 1 |
| Protein tyrosine phosphatase activity | 25 | 25 | 0,14573 | 1,056543 | 0,252587 | 1 | 1 | 1 |
| Ubiquitin-specific protease activity | 360 | 377 | 2,098514 | 1,008902 | 0,274884 | 1 | 1 | 1 |
| Protein serine/threonine phosphatase activity | 43 | 44 | 0,250656 | 1,032536 | 0,309755 | 1 | 1 | 1 |
| Carboxypeptidase activity | 21 | 21 | 0,122413 | 1,056543 | 0,314834 | 1 | 1 | 1 |
| Carboxy-lyase activity | 20 | 20 | 0,116584 | 1,056543 | 0,332657 | 1 | 1 | 1 |
| Phosphorylase activity | 20 | 20 | 0,116584 | 1,056543 | 0,332657 | 1 | 1 | 1 |
| Hydrolase activity | 194 | 203 | 1,130866 | 1,009704 | 0,348565 | 1 | 1 | 1 |
| Sialyltransferase activity | 19 | 19 | 0,110755 | 1,056543 | 0,351487 | 1 | 1 | 1 |
| Protein binding | 174 | 182 | 1,014282 | 1,010104 | 0,356109 | 1 | 1 | 1 |
| Oxidoreductase activity | 154 | 161 | 0,897697 | 1,010609 | 0,364144 | 1 | 1 | 1 |
| Translation regulator activity | 97 | 101 | 0,565433 | 1,014704 | 0,365661 | 1 | 1 | 1 |
| Cytoskeletal anchoring activity | 38 | 39 | 0,22151 | 1,029459 | 0,374892 | 1 | 1 | 1 |
| Methyltransferase activity | 57 | 59 | 0,332265 | 1,020734 | 0,381691 | 1 | 1 | 1 |
| Galactosyltransferase activity | 37 | 38 | 0,215681 | 1,028747 | 0,389128 | 1 | 1 | 1 |
| Deoxyribonuclease activity | 17 | 17 | 0,099096 | 1,056543 | 0,392402 | 1 | 1 | 1 |
| Protein tyrosine/serine/threonine phosphatase activity | 36 | 37 | 0,209851 | 1,027996 | 0,403774 | 1 | 1 | 1 |
| Kinase binding | 16 | 16 | 0,093267 | 1,056543 | 0,414611 | 1 | 1 | 1 |
| Receptor signaling protein tyrosine phosphatase activity | 16 | 16 | 0,093267 | 1,056543 | 0,414611 | 1 | 1 | 1 |
| Growth factor activity | 105 | 110 | 0,612066 | 1,008523 | 0,459588 | 1 | 1 | 1 |
| Deacetylase activity | 14 | 14 | 0,081609 | 1,056543 | 0,462865 | 1 | 1 | 1 |
| Water channel activity | 14 | 14 | 0,081609 | 1,056543 | 0,462865 | 1 | 1 | 1 |
| Inward rectifier channel | 14 | 14 | 0,081609 | 1,056543 | 0,462865 | 1 | 1 | 1 |
| Enzyme regulator activity | 13 | 13 | 0,07578 | 1,056543 | 0,489057 | 1 | 1 | 1 |
| Signal transducer activity | 30 | 31 | 0,174876 | 1,022472 | 0,500183 | 1 | 1 | 1 |
| Aminopeptidase activity | 28 | 29 | 0,163218 | 1,020123 | 0,535454 | 1 | 1 | 1 |
| Nucleotidyltransferase activity | 11 | 11 | 0,064121 | 1,056543 | 0,545966 | 1 | 1 | 1 |
| Fucosyltransferase activity | 11 | 11 | 0,064121 | 1,056543 | 0,545966 | 1 | 1 | 1 |
| Lipid binding | 11 | 11 | 0,064121 | 1,056543 | 0,545966 | 1 | 1 | 1 |
| Adenylate cyclase activity | 10 | 10 | 0,058292 | 1,056543 | 0,576854 | 1 | 1 | 1 |
| Transcription factor binding | 10 | 10 | 0,058292 | 1,056543 | 0,576854 | 1 | 1 | 1 |
| Mannosyltransferase activity | 10 | 10 | 0,058292 | 1,056543 | 0,576854 | 1 | 1 | 1 |
| Defense/immunity protein activity | 58 | 61 | 0,338094 | 1,004591 | 0,586518 | 1 | 1 | 1 |
| Lipid transporter activity | 9 | 9 | 0,052463 | 1,056543 | 0,609489 | 1 | 1 | 1 |
| Nucleic acid binding | 9 | 9 | 0,052463 | 1,056543 | 0,609489 | 1 | 1 | 1 |
| Ubiquitin binding | 9 | 9 | 0,052463 | 1,056543 | 0,609489 | 1 | 1 | 1 |
| Hormone activity | 24 | 25 | 0,139901 | 1,014298 | 0,610139 | 1 | 1 | 1 |
| Helicase activity | 24 | 25 | 0,139901 | 1,014298 | 0,610139 | 1 | 1 | 1 |
| Growth factor binding | 8 | 8 | 0,046634 | 1,056543 | 0,643967 | 1 | 1 | 1 |
| Transcription cofactor activity | 8 | 8 | 0,046634 | 1,056543 | 0,643967 | 1 | 1 | 1 |
| Transaminase activity | 22 | 23 | 0,128242 | 1,010627 | 0,649211 | 1 | 1 | 1 |
| Ion transporter activity | 22 | 23 | 0,128242 | 1,010627 | 0,649211 | 1 | 1 | 1 |
| Kinase regulator activity | 22 | 23 | 0,128242 | 1,010627 | 0,649211 | 1 | 1 | 1 |
| Binding | 21 | 22 | 0,122413 | 1,00854 | 0,669075 | 1 | 1 | 1 |
| Hydro-lyase activity | 7 | 7 | 0,040804 | 1,056543 | 0,680394 | 1 | 1 | 1 |
| Palmitoyltransferase activity | 7 | 7 | 0,040804 | 1,056543 | 0,680394 | 1 | 1 | 1 |
| Aspartic-type signal peptidase activity | 7 | 7 | 0,040804 | 1,056543 | 0,680394 | 1 | 1 | 1 |
| Carbohydrate binding | 7 | 7 | 0,040804 | 1,056543 | 0,680394 | 1 | 1 | 1 |
| Lyase activity | 20 | 21 | 0,116584 | 1,006256 | 0,689108 | 1 | 1 | 1 |
| DNA-directed DNA polymerase activity | 19 | 20 | 0,110755 | 1,003742 | 0,709264 | 1 | 1 | 1 |
| ATP binding | 6 | 6 | 0,034975 | 1,056543 | 0,718879 | 1 | 1 | 1 |
| Peptide binding | 6 | 6 | 0,034975 | 1,056543 | 0,718879 | 1 | 1 | 1 |
| Channel regulator activity | 6 | 6 | 0,034975 | 1,056543 | 0,718879 | 1 | 1 | 1 |
| Phosphoprotein phosphatase activity | 6 | 6 | 0,034975 | 1,056543 | 0,718879 | 1 | 1 | 1 |
| Receptor regulator activity | 6 | 6 | 0,034975 | 1,056543 | 0,718879 | 1 | 1 | 1 |
| DNA topoisomerase activity | 6 | 6 | 0,034975 | 1,056543 | 0,718879 | 1 | 1 | 1 |
| Deaminase activity | 18 | 19 | 0,104926 | 1,000965 | 0,729495 | 1 | 1 | 1 |
| Lipase activity | 18 | 19 | 0,104926 | 1,000965 | 0,729495 | 1 | 1 | 1 |
| Acyltransferase activity | 75 | 80 | 0,43719 | 0,990518 | 0,743408 | 1 | 1 | 1 |
| Amylase activity | 5 | 5 | 0,029146 | 1,056543 | 0,759539 | 1 | 1 | 1 |
| Complement receptor activity | 5 | 5 | 0,029146 | 1,056543 | 0,759539 | 1 | 1 | 1 |
| Protein kinase binding | 5 | 5 | 0,029146 | 1,056543 | 0,759539 | 1 | 1 | 1 |
| Cation channel activity | 5 | 5 | 0,029146 | 1,056543 | 0,759539 | 1 | 1 | 1 |
| B cell receptor activity | 5 | 5 | 0,029146 | 1,056543 | 0,759539 | 1 | 1 | 1 |
| Neurotransmitter transporter activity | 5 | 5 | 0,029146 | 1,056543 | 0,759539 | 1 | 1 | 1 |
| Apoptotic protease activator activity | 5 | 5 | 0,029146 | 1,056543 | 0,759539 | 1 | 1 | 1 |
| Phosphatase regulator activity | 15 | 16 | 0,087438 | 0,990551 | 0,790034 | 1 | 1 | 1 |
| Enzyme activator activity | 4 | 4 | 0,023317 | 1,056543 | 0,802496 | 1 | 1 | 1 |
| DNA-methyltransferase activity | 4 | 4 | 0,023317 | 1,056543 | 0,802496 | 1 | 1 | 1 |
| MRNA binding | 4 | 4 | 0,023317 | 1,056543 | 0,802496 | 1 | 1 | 1 |
| Regulator of G-protein signaling activity | 4 | 4 | 0,023317 | 1,056543 | 0,802496 | 1 | 1 | 1 |
| Sugar-phosphatase activity | 4 | 4 | 0,023317 | 1,056543 | 0,802496 | 1 | 1 | 1 |
| Receptor signaling protein activity | 4 | 4 | 0,023317 | 1,056543 | 0,802496 | 1 | 1 | 1 |
| Intracellular transporter activity | 4 | 4 | 0,023317 | 1,056543 | 0,802496 | 1 | 1 | 1 |
| Protein transporter activity | 4 | 4 | 0,023317 | 1,056543 | 0,802496 | 1 | 1 | 1 |
| Pre-mRNA splicing factor activity | 4 | 4 | 0,023317 | 1,056543 | 0,802496 | 1 | 1 | 1 |
| Receptor signaling protein serine/threonine kinase activity | 13 | 14 | 0,07578 | 0,98113 | 0,82955 | 1 | 1 | 1 |
| Kinase activity | 13 | 14 | 0,07578 | 0,98113 | 0,82955 | 1 | 1 | 1 |
| Chemokine activity | 49 | 53 | 0,285631 | 0,976819 | 0,847406 | 1 | 1 | 1 |
| Storage protein | 3 | 3 | 0,017488 | 1,056543 | 0,847879 | 1 | 1 | 1 |
| Amidinotransferase activity | 3 | 3 | 0,017488 | 1,056543 | 0,847879 | 1 | 1 | 1 |
| Complement binding | 3 | 3 | 0,017488 | 1,056543 | 0,847879 | 1 | 1 | 1 |
| Immunoglobulin receptor activity | 3 | 3 | 0,017488 | 1,056543 | 0,847879 | 1 | 1 | 1 |
| Histone binding | 3 | 3 | 0,017488 | 1,056543 | 0,847879 | 1 | 1 | 1 |
| Ligand-gated ion channel activity | 3 | 3 | 0,017488 | 1,056543 | 0,847879 | 1 | 1 | 1 |
| Protein kinase activity | 3 | 3 | 0,017488 | 1,056543 | 0,847879 | 1 | 1 | 1 |
| Superoxide dismutase activity | 3 | 3 | 0,017488 | 1,056543 | 0,847879 | 1 | 1 | 1 |
| Transferase activity, transferring aldehyde or ketonic groups | 3 | 3 | 0,017488 | 1,056543 | 0,847879 | 1 | 1 | 1 |
| Spliceosomal catalysis | 3 | 3 | 0,017488 | 1,056543 | 0,847879 | 1 | 1 | 1 |
| Carrier activity | 3 | 3 | 0,017488 | 1,056543 | 0,847879 | 1 | 1 | 1 |
| Nucleotide binding | 3 | 3 | 0,017488 | 1,056543 | 0,847879 | 1 | 1 | 1 |
| Protein translocase activity | 3 | 3 | 0,017488 | 1,056543 | 0,847879 | 1 | 1 | 1 |
| Chromatin binding | 12 | 13 | 0,06995 | 0,975333 | 0,848796 | 1 | 1 | 1 |
| ATPase activity | 103 | 111 | 0,600408 | 0,980403 | 0,859781 | 1 | 1 | 1 |
| MHC class I receptor activity | 34 | 37 | 0,198193 | 0,970901 | 0,866008 | 1 | 1 | 1 |
| Racemase and epimerase activity | 10 | 11 | 0,058292 | 0,960581 | 0,885741 | 1 | 1 | 1 |
| Carboxyl- and carbamoyltransferase activity | 2 | 2 | 0,011658 | 1,056543 | 0,895827 | 1 | 1 | 1 |
| Electron transporter activity | 2 | 2 | 0,011658 | 1,056543 | 0,895827 | 1 | 1 | 1 |
| Translation factor activity, nucleic acid binding | 2 | 2 | 0,011658 | 1,056543 | 0,895827 | 1 | 1 | 1 |
| Extracellular matrix binding | 2 | 2 | 0,011658 | 1,056543 | 0,895827 | 1 | 1 | 1 |
| CoA-ligase activity | 2 | 2 | 0,011658 | 1,056543 | 0,895827 | 1 | 1 | 1 |
| Nucleocytoplasmic transporter activity | 2 | 2 | 0,011658 | 1,056543 | 0,895827 | 1 | 1 | 1 |
| Immunoglobulin binding | 2 | 2 | 0,011658 | 1,056543 | 0,895827 | 1 | 1 | 1 |
| Polysaccharide binding | 2 | 2 | 0,011658 | 1,056543 | 0,895827 | 1 | 1 | 1 |
| DNA ligase activity | 2 | 2 | 0,011658 | 1,056543 | 0,895827 | 1 | 1 | 1 |
| Glucosyltransferase activity | 2 | 2 | 0,011658 | 1,056543 | 0,895827 | 1 | 1 | 1 |
| Antioxidant activity | 2 | 2 | 0,011658 | 1,056543 | 0,895827 | 1 | 1 | 1 |
| Receptor signaling protein tyrosine kinase activity | 2 | 2 | 0,011658 | 1,056543 | 0,895827 | 1 | 1 | 1 |
| Amino acid transporter activity | 2 | 2 | 0,011658 | 1,056543 | 0,895827 | 1 | 1 | 1 |
| Lipoprotein receptor activity | 2 | 2 | 0,011658 | 1,056543 | 0,895827 | 1 | 1 | 1 |
| Neurotransmitter receptor activity | 2 | 2 | 0,011658 | 1,056543 | 0,895827 | 1 | 1 | 1 |
| Structural constituent of nuclear pore | 2 | 2 | 0,011658 | 1,056543 | 0,895827 | 1 | 1 | 1 |
| Steroid hormone receptor activity | 2 | 2 | 0,011658 | 1,056543 | 0,895827 | 1 | 1 | 1 |
| Structural constituent of chromatin | 2 | 2 | 0,011658 | 1,056543 | 0,895827 | 1 | 1 | 1 |
| Cytokine binding | 2 | 2 | 0,011658 | 1,056543 | 0,895827 | 1 | 1 | 1 |
| TRNA ligase activity | 2 | 2 | 0,011658 | 1,056543 | 0,895827 | 1 | 1 | 1 |
| Endopeptidase activity | 2 | 2 | 0,011658 | 1,056543 | 0,895827 | 1 | 1 | 1 |
| Protein-hormone receptor activity | 2 | 2 | 0,011658 | 1,056543 | 0,895827 | 1 | 1 | 1 |
| Protease activator activity | 2 | 2 | 0,011658 | 1,056543 | 0,895827 | 1 | 1 | 1 |
| Antiporter activity | 2 | 2 | 0,011658 | 1,056543 | 0,895827 | 1 | 1 | 1 |
| Ubiquitin-like-protein-specific protease activity | 2 | 2 | 0,011658 | 1,056543 | 0,895827 | 1 | 1 | 1 |
| Glutathione transferase activity | 19 | 21 | 0,110755 | 0,955968 | 0,900751 | 1 | 1 | 1 |
| Peroxidase activity | 18 | 20 | 0,104926 | 0,950942 | 0,911567 | 1 | 1 | 1 |
| Glucosidase activity | 8 | 9 | 0,046634 | 0,93928 | 0,919796 | 1 | 1 | 1 |
| T cell receptor activity | 8 | 9 | 0,046634 | 0,93928 | 0,919796 | 1 | 1 | 1 |
| Enzyme inhibitor activity | 7 | 8 | 0,040804 | 0,92464 | 0,935382 | 1 | 1 | 1 |
| Aldehyde dehydrogenase activity | 1 | 1 | 0,005829 | 1,056543 | 0,946483 | 1 | 1 | 1 |
| Hormone binding | 1 | 1 | 0,005829 | 1,056543 | 0,946483 | 1 | 1 | 1 |
| Cofactor binding | 1 | 1 | 0,005829 | 1,056543 | 0,946483 | 1 | 1 | 1 |
| Galactosidase activity | 1 | 1 | 0,005829 | 1,056543 | 0,946483 | 1 | 1 | 1 |
| GTPase regulator activity | 1 | 1 | 0,005829 | 1,056543 | 0,946483 | 1 | 1 | 1 |
| Structural constituent of myelin sheath | 1 | 1 | 0,005829 | 1,056543 | 0,946483 | 1 | 1 | 1 |
| CoA-transferase activity | 1 | 1 | 0,005829 | 1,056543 | 0,946483 | 1 | 1 | 1 |
| Sterol transporter activity | 1 | 1 | 0,005829 | 1,056543 | 0,946483 | 1 | 1 | 1 |
| RNA-directed DNA polymerase activity | 1 | 1 | 0,005829 | 1,056543 | 0,946483 | 1 | 1 | 1 |
| Anion channel activity | 1 | 1 | 0,005829 | 1,056543 | 0,946483 | 1 | 1 | 1 |
| Clathrin binding | 1 | 1 | 0,005829 | 1,056543 | 0,946483 | 1 | 1 | 1 |
| Oxidative phosphorylation uncoupler activity | 1 | 1 | 0,005829 | 1,056543 | 0,946483 | 1 | 1 | 1 |
| Dipeptidase activity | 1 | 1 | 0,005829 | 1,056543 | 0,946483 | 1 | 1 | 1 |
| Coenzyme binding | 1 | 1 | 0,005829 | 1,056543 | 0,946483 | 1 | 1 | 1 |
| Catalase activity | 1 | 1 | 0,005829 | 1,056543 | 0,946483 | 1 | 1 | 1 |
| Steroid binding | 1 | 1 | 0,005829 | 1,056543 | 0,946483 | 1 | 1 | 1 |
| Peptide transporter activity | 1 | 1 | 0,005829 | 1,056543 | 0,946483 | 1 | 1 | 1 |
| Signal peptidase activity | 1 | 1 | 0,005829 | 1,056543 | 0,946483 | 1 | 1 | 1 |
| FAD binding | 1 | 1 | 0,005829 | 1,056543 | 0,946483 | 1 | 1 | 1 |
| G-protein-coupled receptor binding | 1 | 1 | 0,005829 | 1,056543 | 0,946483 | 1 | 1 | 1 |
| Pattern recognition receptor activity | 1 | 1 | 0,005829 | 1,056543 | 0,946483 | 1 | 1 | 1 |
| NADP binding | 1 | 1 | 0,005829 | 1,056543 | 0,946483 | 1 | 1 | 1 |
| Vitamin or cofactor transporter activity | 1 | 1 | 0,005829 | 1,056543 | 0,946483 | 1 | 1 | 1 |
| Glycosaminoglycan binding | 1 | 1 | 0,005829 | 1,056543 | 0,946483 | 1 | 1 | 1 |
| Protein channel activity | 1 | 1 | 0,005829 | 1,056543 | 0,946483 | 1 | 1 | 1 |
| Amine transporter activity | 1 | 1 | 0,005829 | 1,056543 | 0,946483 | 1 | 1 | 1 |
| Nuclease activity | 1 | 1 | 0,005829 | 1,056543 | 0,946483 | 1 | 1 | 1 |
| MHC class II receptor activity | 34 | 38 | 0,198193 | 0,945357 | 0,949492 | 1 | 1 | 1 |
| Metal ion binding | 6 | 7 | 0,034975 | 0,905824 | 0,94979 | 1 | 1 | 1 |
| Caspase activator activity | 6 | 7 | 0,034975 | 0,905824 | 0,94979 | 1 | 1 | 1 |
| Endonuclease activity | 6 | 7 | 0,034975 | 0,905824 | 0,94979 | 1 | 1 | 1 |
| Protein domain specific binding | 5 | 6 | 0,029146 | 0,880746 | 0,962837 | 1 | 1 | 1 |
| RNA methyltransferase activity | 10 | 12 | 0,058292 | 0,880599 | 0,976622 | 1 | 1 | 1 |
| Exonuclease activity | 8 | 10 | 0,046634 | 0,845446 | 0,986187 | 1 | 1 | 1 |
| Channel or pore class transporter activity | 2 | 3 | 0,011658 | 0,705532 | 0,991722 | 1 | 1 | 1 |
| Guanylate cyclase activity | 6 | 8 | 0,034975 | 0,792737 | 0,993014 | 1 | 1 | 1 |
| Alkaline phosphatase activity | 1 | 2 | 0,005829 | 0,5309 | 0,997139 | 1 | 1 | 1 |
| Aminomethyltransferase activity | 1 | 2 | 0,005829 | 0,5309 | 0,997139 | 1 | 1 | 1 |
| GTP binding | 5 | 8 | 0,029146 | 0,660834 | 0,99952 | 1 | 1 | 1 |
| Acid phosphatase activity | 8 | 12 | 0,046634 | 0,704655 | 0,999749 | 1 | 1 | 1 |
| Lipid phosphatase activity | 21 | 28 | 0,122413 | 0,792502 | 0,999922 | 1 | 1 | 1 |
| Ligase activity | 91 | 112 | 0,530458 | 0,858459 | 1 | 1 | 1 | 1 |
| DNA binding | 563 | 654 | 3,281842 | 0,909534 | 1 | 1 | 1 | 1 |
| Molecular function unknown | 5085 | 5685 | 29,6415 | 0,945035 | 1 | 1 | 1 | 1 |

Table S8. miRNAs classification by biological process.

| Biological process | No. of genes in the dataset | No. of genes in the background dataset | Percentage of genes | Fold enrichment | P-value (Hypergeometric test) | Bonferroni method | BH method | Q-value (Storey-Tibshirani method) |
| --- | --- | --- | --- | --- | --- | --- | --- | --- |
| Signal transduction | 3842 | 3934 | 22,39711 | 1,031838 | 3,74E-25 | 6,66E-23 | 6,66E-23 | 3,07734E-22 |
| Cell communication | 3628 | 3713 | 21,14959 | 1,03236 | 2,21E-24 | 3,93E-22 | 1,96E-22 | 9,07129E-22 |
| Transport | 1201 | 1215 | 7,001282 | 1,044372 | 1,47E-15 | 2,62E-13 | 8,72E-14 | 4,02829E-13 |
| Cell growth and/or maintenance | 1103 | 1125 | 6,429987 | 1,035885 | 3,01E-09 | 5,36E-07 | 1,34E-07 | 6,19121E-07 |
| Protein metabolism | 1284 | 1323 | 7,485135 | 1,025401 | 9,61E-06 | 0,00171 | 0,000342 | 0,001580304 |
| Metabolism | 1614 | 1683 | 9,408884 | 1,01323 | 0,007997 | 1 | 0,237248 | 1 |
| Energy pathways | 1565 | 1633 | 9,123237 | 1,012551 | 0,012631 | 1 | 0,3038 | 1 |
| Regulation of nucleobase, nucleoside, nucleotide and nucleic acid metabolism | 2701 | 2828 | 15,7456 | 1,009099 | 0,013654 | 1 | 0,3038 | 1 |
| Immune response | 556 | 576 | 3,241227 | 1,019862 | 0,021122 | 1 | 0,41774 | 1 |
| Cell differentiation | 29 | 29 | 0,169057 | 1,056547 | 0,202618 | 1 | 1 | 1 |
| Cell adhesion | 53 | 54 | 0,308966 | 1,036984 | 0,207451 | 1 | 1 | 1 |
| Apoptosis | 211 | 220 | 1,230034 | 1,013326 | 0,254046 | 1 | 1 | 1 |
| Transcription | 45 | 46 | 0,262329 | 1,033583 | 0,286406 | 1 | 1 | 1 |
| Regulation of gene expression, epigenetic | 64 | 66 | 0,373091 | 1,024535 | 0,306725 | 1 | 1 | 1 |
| Regulation of cell growth | 21 | 21 | 0,12242 | 1,056547 | 0,314814 | 1 | 1 | 1 |
| Cytoskeleton organization and biogenesis | 21 | 21 | 0,12242 | 1,056547 | 0,314814 | 1 | 1 | 1 |
| Protein modification | 20 | 20 | 0,116591 | 1,056547 | 0,332636 | 1 | 1 | 1 |
| DNA repair | 56 | 58 | 0,326454 | 1,02012 | 0,393314 | 1 | 1 | 1 |
| Proteolysis and peptidolysis | 16 | 16 | 0,093273 | 1,056547 | 0,41459 | 1 | 1 | 1 |
| Cell migration | 15 | 15 | 0,087443 | 1,056547 | 0,438054 | 1 | 1 | 1 |
| Inflammatory response | 14 | 14 | 0,081614 | 1,056547 | 0,462845 | 1 | 1 | 1 |
| Anti-apoptosis | 31 | 32 | 0,180716 | 1,02354 | 0,483086 | 1 | 1 | 1 |
| DNA replication | 13 | 13 | 0,075784 | 1,056547 | 0,489037 | 1 | 1 | 1 |
| Cell development | 13 | 13 | 0,075784 | 1,056547 | 0,489037 | 1 | 1 | 1 |
| Regulation of translation | 13 | 13 | 0,075784 | 1,056547 | 0,489037 | 1 | 1 | 1 |
| Fatty acid metabolism | 12 | 12 | 0,069955 | 1,056547 | 0,51671 | 1 | 1 | 1 |
| Cell-cell adhesion | 12 | 12 | 0,069955 | 1,056547 | 0,51671 | 1 | 1 | 1 |
| Protein transport | 11 | 11 | 0,064125 | 1,056547 | 0,545947 | 1 | 1 | 1 |
| G-protein coupled receptor protein signaling pathway | 10 | 10 | 0,058295 | 1,056547 | 0,576836 | 1 | 1 | 1 |
| RNA metabolism | 25 | 26 | 0,145739 | 1,015926 | 0,59097 | 1 | 1 | 1 |
| Calcium-mediated signaling | 9 | 9 | 0,052466 | 1,056547 | 0,609471 | 1 | 1 | 1 |
| Reproduction | 9 | 9 | 0,052466 | 1,056547 | 0,609471 | 1 | 1 | 1 |
| Carbohydrate metabolism | 8 | 8 | 0,046636 | 1,056547 | 0,643951 | 1 | 1 | 1 |
| Regulation of immune response | 8 | 8 | 0,046636 | 1,056547 | 0,643951 | 1 | 1 | 1 |
| Cell surface receptor linked signal transduction | 8 | 8 | 0,046636 | 1,056547 | 0,643951 | 1 | 1 | 1 |
| Regulation of cell proliferation | 22 | 23 | 0,12825 | 1,01063 | 0,649186 | 1 | 1 | 1 |
| Cell proliferation | 37 | 39 | 0,215693 | 1,002379 | 0,652463 | 1 | 1 | 1 |
| Lipid transport | 7 | 7 | 0,040807 | 1,056547 | 0,680379 | 1 | 1 | 1 |
| Neurogenesis | 7 | 7 | 0,040807 | 1,056547 | 0,680379 | 1 | 1 | 1 |
| Nucleobase, nucleoside, nucleotide and nucleic acid transport | 7 | 7 | 0,040807 | 1,056547 | 0,680379 | 1 | 1 | 1 |
| Cell organization and biogenesis | 7 | 7 | 0,040807 | 1,056547 | 0,680379 | 1 | 1 | 1 |
| Cell motility | 19 | 20 | 0,110761 | 1,003746 | 0,709242 | 1 | 1 | 1 |
| Xenobiotic metabolism | 6 | 6 | 0,034977 | 1,056547 | 0,718866 | 1 | 1 | 1 |
| Development | 6 | 6 | 0,034977 | 1,056547 | 0,718866 | 1 | 1 | 1 |
| Cell death | 6 | 6 | 0,034977 | 1,056547 | 0,718866 | 1 | 1 | 1 |
| Regulation of physiological process | 5 | 5 | 0,029148 | 1,056547 | 0,759527 | 1 | 1 | 1 |
| Synapse organization and biogenesis | 5 | 5 | 0,029148 | 1,056547 | 0,759527 | 1 | 1 | 1 |
| Mitochondrial transport | 5 | 5 | 0,029148 | 1,056547 | 0,759527 | 1 | 1 | 1 |
| Microtubule-based process | 5 | 5 | 0,029148 | 1,056547 | 0,759527 | 1 | 1 | 1 |
| Regulation of enzyme activity | 5 | 5 | 0,029148 | 1,056547 | 0,759527 | 1 | 1 | 1 |
| Muscle contraction | 5 | 5 | 0,029148 | 1,056547 | 0,759527 | 1 | 1 | 1 |
| Protein targeting | 5 | 5 | 0,029148 | 1,056547 | 0,759527 | 1 | 1 | 1 |
| Mitosis | 5 | 5 | 0,029148 | 1,056547 | 0,759527 | 1 | 1 | 1 |
| Muscle development | 5 | 5 | 0,029148 | 1,056547 | 0,759527 | 1 | 1 | 1 |
| Regulation of metabolism | 5 | 5 | 0,029148 | 1,056547 | 0,759527 | 1 | 1 | 1 |
| Protein folding | 15 | 16 | 0,087443 | 0,990554 | 0,790017 | 1 | 1 | 1 |
| Cell-cell signaling | 15 | 16 | 0,087443 | 0,990554 | 0,790017 | 1 | 1 | 1 |
| Amino acid and derivative metabolism | 4 | 4 | 0,023318 | 1,056547 | 0,802486 | 1 | 1 | 1 |
| Physiological process | 4 | 4 | 0,023318 | 1,056547 | 0,802486 | 1 | 1 | 1 |
| Regulation of biological process | 4 | 4 | 0,023318 | 1,056547 | 0,802486 | 1 | 1 | 1 |
| Peptide metabolism | 4 | 4 | 0,023318 | 1,056547 | 0,802486 | 1 | 1 | 1 |
| Synaptic transmission | 4 | 4 | 0,023318 | 1,056547 | 0,802486 | 1 | 1 | 1 |
| Cytokine and chemokine mediated signaling pathway | 4 | 4 | 0,023318 | 1,056547 | 0,802486 | 1 | 1 | 1 |
| Complement activation | 4 | 4 | 0,023318 | 1,056547 | 0,802486 | 1 | 1 | 1 |
| Hemopoiesis | 4 | 4 | 0,023318 | 1,056547 | 0,802486 | 1 | 1 | 1 |
| Cellular defense response | 4 | 4 | 0,023318 | 1,056547 | 0,802486 | 1 | 1 | 1 |
| Cytoskeletal anchoring | 4 | 4 | 0,023318 | 1,056547 | 0,802486 | 1 | 1 | 1 |
| Cell maturation | 4 | 4 | 0,023318 | 1,056547 | 0,802486 | 1 | 1 | 1 |
| Cell-matrix adhesion | 4 | 4 | 0,023318 | 1,056547 | 0,802486 | 1 | 1 | 1 |
| Cell recognition | 4 | 4 | 0,023318 | 1,056547 | 0,802486 | 1 | 1 | 1 |
| Ribosome biogenesis and assembly | 4 | 4 | 0,023318 | 1,056547 | 0,802486 | 1 | 1 | 1 |
| Neurotransmitter transport | 4 | 4 | 0,023318 | 1,056547 | 0,802486 | 1 | 1 | 1 |
| Regulation of endocytosis | 4 | 4 | 0,023318 | 1,056547 | 0,802486 | 1 | 1 | 1 |
| Vesicle docking | 4 | 4 | 0,023318 | 1,056547 | 0,802486 | 1 | 1 | 1 |
| Cell fate commitment | 4 | 4 | 0,023318 | 1,056547 | 0,802486 | 1 | 1 | 1 |
| Regulation of development | 4 | 4 | 0,023318 | 1,056547 | 0,802486 | 1 | 1 | 1 |
| Regulation of cell cycle | 53 | 57 | 0,308966 | 0,982416 | 0,81152 | 1 | 1 | 1 |
| Regulation of blood pressure | 3 | 3 | 0,017489 | 1,056547 | 0,847871 | 1 | 1 | 1 |
| Homeostasis | 3 | 3 | 0,017489 | 1,056547 | 0,847871 | 1 | 1 | 1 |
| Endosome transport | 3 | 3 | 0,017489 | 1,056547 | 0,847871 | 1 | 1 | 1 |
| Negative regulation of enzyme activity | 3 | 3 | 0,017489 | 1,056547 | 0,847871 | 1 | 1 | 1 |
| Bone remodeling | 3 | 3 | 0,017489 | 1,056547 | 0,847871 | 1 | 1 | 1 |
| Hormone metabolism | 3 | 3 | 0,017489 | 1,056547 | 0,847871 | 1 | 1 | 1 |
| Cellular morphogenesis during differentiation | 3 | 3 | 0,017489 | 1,056547 | 0,847871 | 1 | 1 | 1 |
| Regulation of cell shape | 3 | 3 | 0,017489 | 1,056547 | 0,847871 | 1 | 1 | 1 |
| Wound healing | 3 | 3 | 0,017489 | 1,056547 | 0,847871 | 1 | 1 | 1 |
| Regulation of transport | 3 | 3 | 0,017489 | 1,056547 | 0,847871 | 1 | 1 | 1 |
| Meiosis | 3 | 3 | 0,017489 | 1,056547 | 0,847871 | 1 | 1 | 1 |
| Intracellular signaling cascade | 3 | 3 | 0,017489 | 1,056547 | 0,847871 | 1 | 1 | 1 |
| Protein localization | 3 | 3 | 0,017489 | 1,056547 | 0,847871 | 1 | 1 | 1 |
| Amino acid transport | 3 | 3 | 0,017489 | 1,056547 | 0,847871 | 1 | 1 | 1 |
| Morphogenesis | 3 | 3 | 0,017489 | 1,056547 | 0,847871 | 1 | 1 | 1 |
| Steroid metabolism | 3 | 3 | 0,017489 | 1,056547 | 0,847871 | 1 | 1 | 1 |
| Synaptic vesicle transport | 3 | 3 | 0,017489 | 1,056547 | 0,847871 | 1 | 1 | 1 |
| Signal complex formation | 3 | 3 | 0,017489 | 1,056547 | 0,847871 | 1 | 1 | 1 |
| Regulation of protein kinase activity | 3 | 3 | 0,017489 | 1,056547 | 0,847871 | 1 | 1 | 1 |
| Gene silencing | 3 | 3 | 0,017489 | 1,056547 | 0,847871 | 1 | 1 | 1 |
| Biological_process | 3 | 3 | 0,017489 | 1,056547 | 0,847871 | 1 | 1 | 1 |
| Lipoprotein metabolism | 3 | 3 | 0,017489 | 1,056547 | 0,847871 | 1 | 1 | 1 |
| Humoral immune response | 3 | 3 | 0,017489 | 1,056547 | 0,847871 | 1 | 1 | 1 |
| Regulation of signal transduction | 22 | 24 | 0,12825 | 0,968538 | 0,865419 | 1 | 1 | 1 |
| Embryonic development | 10 | 11 | 0,058295 | 0,960584 | 0,885731 | 1 | 1 | 1 |
| Ion transport | 44 | 48 | 0,2565 | 0,968519 | 0,887601 | 1 | 1 | 1 |
| Cell cycle | 20 | 22 | 0,116591 | 0,96054 | 0,889421 | 1 | 1 | 1 |
| Neurotransmitter metabolism | 2 | 2 | 0,011659 | 1,056547 | 0,895821 | 1 | 1 | 1 |
| Lipid storage | 2 | 2 | 0,011659 | 1,056547 | 0,895821 | 1 | 1 | 1 |
| Osmoregulation | 2 | 2 | 0,011659 | 1,056547 | 0,895821 | 1 | 1 | 1 |
| Cellular morphogenesis | 2 | 2 | 0,011659 | 1,056547 | 0,895821 | 1 | 1 | 1 |
| CGMP-mediated signaling | 2 | 2 | 0,011659 | 1,056547 | 0,895821 | 1 | 1 | 1 |
| Transmembrane receptor protein tyrosine kinase signaling pathway | 2 | 2 | 0,011659 | 1,056547 | 0,895821 | 1 | 1 | 1 |
| CAMP-mediated signaling | 2 | 2 | 0,011659 | 1,056547 | 0,895821 | 1 | 1 | 1 |
| Intercellular junction assembly and/or maintenance | 2 | 2 | 0,011659 | 1,056547 | 0,895821 | 1 | 1 | 1 |
| Vasodilation | 2 | 2 | 0,011659 | 1,056547 | 0,895821 | 1 | 1 | 1 |
| Steroid hormone receptor signaling pathway | 2 | 2 | 0,011659 | 1,056547 | 0,895821 | 1 | 1 | 1 |
| Vitamin/cofactor transport | 2 | 2 | 0,011659 | 1,056547 | 0,895821 | 1 | 1 | 1 |
| Mitochondrion organization and biogenesis | 2 | 2 | 0,011659 | 1,056547 | 0,895821 | 1 | 1 | 1 |
| Reproductive physiological process | 2 | 2 | 0,011659 | 1,056547 | 0,895821 | 1 | 1 | 1 |
| Spindle assembly | 2 | 2 | 0,011659 | 1,056547 | 0,895821 | 1 | 1 | 1 |
| DNA metabolism | 2 | 2 | 0,011659 | 1,056547 | 0,895821 | 1 | 1 | 1 |
| Carbohydrate mediated signaling | 2 | 2 | 0,011659 | 1,056547 | 0,895821 | 1 | 1 | 1 |
| Reproductive behavior | 2 | 2 | 0,011659 | 1,056547 | 0,895821 | 1 | 1 | 1 |
| Vitamin metabolism | 2 | 2 | 0,011659 | 1,056547 | 0,895821 | 1 | 1 | 1 |
| Hormone secretion | 2 | 2 | 0,011659 | 1,056547 | 0,895821 | 1 | 1 | 1 |
| Organogenesis | 8 | 9 | 0,046636 | 0,939283 | 0,919788 | 1 | 1 | 1 |
| Vesicle-mediated transport | 15 | 17 | 0,087443 | 0,93232 | 0,940691 | 1 | 1 | 1 |
| Aldehyde metabolism | 1 | 1 | 0,00583 | 1,056547 | 0,94648 | 1 | 1 | 1 |
| Purine salvage | 1 | 1 | 0,00583 | 1,056547 | 0,94648 | 1 | 1 | 1 |
| Regulation of circadian rhythm | 1 | 1 | 0,00583 | 1,056547 | 0,94648 | 1 | 1 | 1 |
| Regulation of coagulation | 1 | 1 | 0,00583 | 1,056547 | 0,94648 | 1 | 1 | 1 |
| Glycosaminoglycan metabolism | 1 | 1 | 0,00583 | 1,056547 | 0,94648 | 1 | 1 | 1 |
| Enzyme linked receptor protein signaling pathway | 1 | 1 | 0,00583 | 1,056547 | 0,94648 | 1 | 1 | 1 |
| Lysosome organization and biogenesis | 1 | 1 | 0,00583 | 1,056547 | 0,94648 | 1 | 1 | 1 |
| Immune cell migration | 1 | 1 | 0,00583 | 1,056547 | 0,94648 | 1 | 1 | 1 |
| Hormone mediated signaling | 1 | 1 | 0,00583 | 1,056547 | 0,94648 | 1 | 1 | 1 |
| Chemosensory behavior | 1 | 1 | 0,00583 | 1,056547 | 0,94648 | 1 | 1 | 1 |
| Lymphocyte activation | 1 | 1 | 0,00583 | 1,056547 | 0,94648 | 1 | 1 | 1 |
| Lymphocyte proliferation | 1 | 1 | 0,00583 | 1,056547 | 0,94648 | 1 | 1 | 1 |
| Nuclear organization and biogenesis | 1 | 1 | 0,00583 | 1,056547 | 0,94648 | 1 | 1 | 1 |
| Pyrimidine salvage | 1 | 1 | 0,00583 | 1,056547 | 0,94648 | 1 | 1 | 1 |
| Peroxisome organization and biogenesis | 1 | 1 | 0,00583 | 1,056547 | 0,94648 | 1 | 1 | 1 |
| ER organization and biogenesis | 1 | 1 | 0,00583 | 1,056547 | 0,94648 | 1 | 1 | 1 |
| Golgi organization and biogenesis | 1 | 1 | 0,00583 | 1,056547 | 0,94648 | 1 | 1 | 1 |
| Glycoprotein metabolism | 1 | 1 | 0,00583 | 1,056547 | 0,94648 | 1 | 1 | 1 |
| Regulation of hormone secretion | 1 | 1 | 0,00583 | 1,056547 | 0,94648 | 1 | 1 | 1 |
| Antigen presentation | 1 | 1 | 0,00583 | 1,056547 | 0,94648 | 1 | 1 | 1 |
| Secretory pathway | 1 | 1 | 0,00583 | 1,056547 | 0,94648 | 1 | 1 | 1 |
| Antigen receptor-mediated signaling pathway | 1 | 1 | 0,00583 | 1,056547 | 0,94648 | 1 | 1 | 1 |
| Cell homeostasis | 1 | 1 | 0,00583 | 1,056547 | 0,94648 | 1 | 1 | 1 |
| Cellular process | 1 | 1 | 0,00583 | 1,056547 | 0,94648 | 1 | 1 | 1 |
| Coenzyme and prosthetic group metabolism | 1 | 1 | 0,00583 | 1,056547 | 0,94648 | 1 | 1 | 1 |
| Phosphoinositide-mediated signaling | 1 | 1 | 0,00583 | 1,056547 | 0,94648 | 1 | 1 | 1 |
| Extracellular structure organization and biogenesis | 1 | 1 | 0,00583 | 1,056547 | 0,94648 | 1 | 1 | 1 |
| Cell activation | 1 | 1 | 0,00583 | 1,056547 | 0,94648 | 1 | 1 | 1 |
| Learning and/or memory | 1 | 1 | 0,00583 | 1,056547 | 0,94648 | 1 | 1 | 1 |
| Blood vessel development | 1 | 1 | 0,00583 | 1,056547 | 0,94648 | 1 | 1 | 1 |
| Vesicle organization and biogenesis | 1 | 1 | 0,00583 | 1,056547 | 0,94648 | 1 | 1 | 1 |
| Gonad development | 1 | 1 | 0,00583 | 1,056547 | 0,94648 | 1 | 1 | 1 |
| Regulation of viral life cycle | 1 | 1 | 0,00583 | 1,056547 | 0,94648 | 1 | 1 | 1 |
| Translation | 1 | 1 | 0,00583 | 1,056547 | 0,94648 | 1 | 1 | 1 |
| Regulation of membrane potential | 1 | 1 | 0,00583 | 1,056547 | 0,94648 | 1 | 1 | 1 |
| Plasma membrane organization and biogenesis | 1 | 1 | 0,00583 | 1,056547 | 0,94648 | 1 | 1 | 1 |
| G-protein signaling, coupled to cyclic nucleotide second messenger | 1 | 1 | 0,00583 | 1,056547 | 0,94648 | 1 | 1 | 1 |
| Locomotory behavior | 1 | 1 | 0,00583 | 1,056547 | 0,94648 | 1 | 1 | 1 |
| Regulation of behavior | 1 | 1 | 0,00583 | 1,056547 | 0,94648 | 1 | 1 | 1 |
| Chromosome segregation | 6 | 7 | 0,034977 | 0,905827 | 0,949785 | 1 | 1 | 1 |
| Electron transport | 5 | 6 | 0,029148 | 0,880748 | 0,962833 | 1 | 1 | 1 |
| Regulation of exocytosis | 4 | 5 | 0,023318 | 0,845659 | 0,97432 | 1 | 1 | 1 |
| Drug metabolism | 3 | 4 | 0,017489 | 0,793069 | 0,984029 | 1 | 1 | 1 |
| Chromosome organization and biogenesis | 3 | 4 | 0,017489 | 0,793069 | 0,984029 | 1 | 1 | 1 |
| Skeletal development | 3 | 4 | 0,017489 | 0,793069 | 0,984029 | 1 | 1 | 1 |
| Innate immune response | 3 | 4 | 0,017489 | 0,793069 | 0,984029 | 1 | 1 | 1 |
| Regulation of cellular process | 2 | 3 | 0,011659 | 0,705534 | 0,991721 | 1 | 1 | 1 |
| Lipid metabolism | 28 | 33 | 0,163227 | 0,896512 | 0,9926 | 1 | 1 | 1 |
| RNA localization | 1 | 2 | 0,00583 | 0,530901 | 0,997138 | 1 | 1 | 1 |
| Behavior | 1 | 2 | 0,00583 | 0,530901 | 0,997138 | 1 | 1 | 1 |
| Biological_process unknown | 4179 | 4731 | 24,36166 | 0,933272 | 1 | 1 | 1 | 1 |

Table S9. miRNAs classification by biological pathway.

| Biological pathway | No. of genes in the dataset | No. of genes in the background dataset | Percentage of genes | Fold enrichment | P-value (Hypergeometric test) | Bonferroni method | BH method | Q-value (Storey-Tibshirani method) |
| --- | --- | --- | --- | --- | --- | --- | --- | --- |
| Neuronal System | 188 | 188 | 3,078435 | 1,029966 | 0 | 0 | 0 | 0 |
| Metabolism of proteins | 259 | 259 | 4,241035 | 1,029966 | 0 | 0 | 0 | 0 |
| Signal Transduction | 1198 | 1208 | 19,61683 | 1,021439 | 5,48E-08 | 9,14E-05 | 3,05E-05 | 9,28192E-05 |
| Developmental Biology | 431 | 432 | 7,057475 | 1,027581 | 2,53E-05 | 0,042133 | 0,00353 | 0,010750134 |
| Glypican pathway | 1317 | 1335 | 21,56542 | 1,016078 | 2,82E-05 | 0,046987 | 0,00353 | 0,010750134 |
| Signaling by GPCR | 793 | 800 | 12,9851 | 1,020953 | 2,9E-05 | 0,048331 | 0,00353 | 0,010750134 |
| TRAIL signaling pathway | 1307 | 1325 | 21,40167 | 1,015974 | 3,47E-05 | 0,057837 | 0,00353 | 0,010750134 |
| ErbB receptor signaling network | 1290 | 1308 | 21,1233 | 1,015792 | 4,92E-05 | 0,082028 | 0,00353 | 0,010750134 |
| Sphingosine 1-phosphate (S1P) pathway | 1290 | 1308 | 21,1233 | 1,015792 | 4,92E-05 | 0,082028 | 0,00353 | 0,010750134 |
| LKB1 signaling events | 1287 | 1305 | 21,07418 | 1,015759 | 5,23E-05 | 0,087203 | 0,00353 | 0,010750134 |
| Endothelins | 1286 | 1304 | 21,0578 | 1,015748 | 5,34E-05 | 0,088997 | 0,00353 | 0,010750134 |
| Proteoglycan syndecan-mediated signaling events | 1323 | 1342 | 21,66366 | 1,015383 | 6,07E-05 | 0,101311 | 0,00353 | 0,010750134 |
| Syndecan-1-mediated signaling events | 1279 | 1297 | 20,94318 | 1,015672 | 6,15E-05 | 0,102582 | 0,00353 | 0,010750134 |
| Glypican 1 network | 1278 | 1296 | 20,92681 | 1,015661 | 6,28E-05 | 0,104678 | 0,00353 | 0,010750134 |
| Signaling events mediated by VEGFR1 and VEGFR2 | 1275 | 1293 | 20,87768 | 1,015627 | 6,67E-05 | 0,111216 | 0,00353 | 0,010750134 |
| IFN-gamma pathway | 1275 | 1293 | 20,87768 | 1,015627 | 6,67E-05 | 0,111216 | 0,00353 | 0,010750134 |
| IL3-mediated signaling events | 1274 | 1292 | 20,86131 | 1,015616 | 6,8E-05 | 0,113481 | 0,00353 | 0,010750134 |
| Signaling events mediated by Hepatocyte Growth Factor Receptor (c-Met) | 1272 | 1290 | 20,82856 | 1,015594 | 7,08E-05 | 0,118145 | 0,00353 | 0,010750134 |
| PDGF receptor signaling network | 1272 | 1290 | 20,82856 | 1,015594 | 7,08E-05 | 0,118145 | 0,00353 | 0,010750134 |
| GMCSF-mediated signaling events | 1271 | 1289 | 20,81218 | 1,015583 | 7,23E-05 | 0,120545 | 0,00353 | 0,010750134 |
| IL5-mediated signaling events | 1271 | 1289 | 20,81218 | 1,015583 | 7,23E-05 | 0,120545 | 0,00353 | 0,010750134 |
| Integrin family cell surface interactions | 1355 | 1375 | 22,18765 | 1,014984 | 7,55E-05 | 0,125876 | 0,00353 | 0,010750134 |
| EGFR-dependent Endothelin signaling events | 1268 | 1286 | 20,76306 | 1,015549 | 7,68E-05 | 0,128029 | 0,00353 | 0,010750134 |
| Insulin Pathway | 1267 | 1285 | 20,74668 | 1,015538 | 7,83E-05 | 0,130622 | 0,00353 | 0,010750134 |
| Internalization of ErbB1 | 1267 | 1285 | 20,74668 | 1,015538 | 7,83E-05 | 0,130622 | 0,00353 | 0,010750134 |
| Urokinase-type plasminogen activator (uPA) and uPAR-mediated signaling | 1267 | 1285 | 20,74668 | 1,015538 | 7,83E-05 | 0,130622 | 0,00353 | 0,010750134 |
| PDGFR-beta signaling pathway | 1267 | 1285 | 20,74668 | 1,015538 | 7,83E-05 | 0,130622 | 0,00353 | 0,010750134 |
| EGF receptor (ErbB1) signaling pathway | 1267 | 1285 | 20,74668 | 1,015538 | 7,83E-05 | 0,130622 | 0,00353 | 0,010750134 |
| Class I PI3K signaling events | 1267 | 1285 | 20,74668 | 1,015538 | 7,83E-05 | 0,130622 | 0,00353 | 0,010750134 |
| Arf6 signaling events | 1267 | 1285 | 20,74668 | 1,015538 | 7,83E-05 | 0,130622 | 0,00353 | 0,010750134 |
| mTOR signaling pathway | 1267 | 1285 | 20,74668 | 1,015538 | 7,83E-05 | 0,130622 | 0,00353 | 0,010750134 |
| Signaling events mediated by focal adhesion kinase | 1267 | 1285 | 20,74668 | 1,015538 | 7,83E-05 | 0,130622 | 0,00353 | 0,010750134 |
| Arf6 downstream pathway | 1267 | 1285 | 20,74668 | 1,015538 | 7,83E-05 | 0,130622 | 0,00353 | 0,010750134 |
| S1P1 pathway | 1267 | 1285 | 20,74668 | 1,015538 | 7,83E-05 | 0,130622 | 0,00353 | 0,010750134 |
| ErbB1 downstream signaling | 1267 | 1285 | 20,74668 | 1,015538 | 7,83E-05 | 0,130622 | 0,00353 | 0,010750134 |
| Arf6 trafficking events | 1267 | 1285 | 20,74668 | 1,015538 | 7,83E-05 | 0,130622 | 0,00353 | 0,010750134 |
| Class I PI3K signaling events mediated by Akt | 1267 | 1285 | 20,74668 | 1,015538 | 7,83E-05 | 0,130622 | 0,00353 | 0,010750134 |
| Beta1 integrin cell surface interactions | 1328 | 1348 | 21,74554 | 1,014684 | 0,000128 | 0,212975 | 0,005605 | 0,017066557 |
| Alpha9 beta1 integrin signaling events | 1283 | 1302 | 21,00868 | 1,014935 | 0,000134 | 0,223017 | 0,005685 | 0,017309951 |
| VEGF and VEGFR signaling network | 1282 | 1301 | 20,9923 | 1,014924 | 0,000136 | 0,227382 | 0,005685 | 0,017309951 |
| Plasma membrane estrogen receptor signaling | 1279 | 1298 | 20,94318 | 1,014889 | 0,000144 | 0,240972 | 0,005825 | 0,017736539 |
| Thrombin/protease-activated receptor (PAR) pathway | 1278 | 1297 | 20,92681 | 1,014878 | 0,000147 | 0,245672 | 0,005825 | 0,017736539 |
| PAR1-mediated thrombin signaling events | 1277 | 1296 | 20,91043 | 1,014866 | 0,00015 | 0,250459 | 0,005825 | 0,017736539 |
| IGF1 pathway | 1269 | 1288 | 20,77943 | 1,014772 | 0,000175 | 0,292096 | 0,006639 | 0,020214968 |
| Nectin adhesion pathway | 1272 | 1292 | 20,82856 | 1,014022 | 0,000365 | 0,609544 | 0,013545 | 0,041247036 |
| GPCR ligand binding | 335 | 336 | 5,485508 | 1,0269 | 0,000396 | 0,661304 | 0,014376 | 0,04377673 |
| Class A/1 (Rhodopsin-like receptors) | 273 | 274 | 4,47028 | 1,026207 | 0,002193 | 1 | 0,077834 | 0,237011772 |
| ALK1 pathway | 322 | 324 | 5,272638 | 1,023608 | 0,003373 | 1 | 0,117204 | 0,356898217 |
| ALK1 signaling events | 319 | 321 | 5,223514 | 1,023549 | 0,003637 | 1 | 0,123353 | 0,375621746 |
| Transmembrane transport of small molecules | 375 | 378 | 6,140495 | 1,021791 | 0,003698 | 1 | 0,123353 | 0,375621746 |
| Regulation of CDC42 activity | 757 | 768 | 12,39561 | 1,015214 | 0,003776 | 1 | 0,123499 | 0,376065839 |
| GPCR downstream signaling | 524 | 530 | 8,580318 | 1,018306 | 0,004032 | 1 | 0,129323 | 0,393799328 |
| CDC42 signaling events | 744 | 755 | 12,18274 | 1,01496 | 0,004776 | 1 | 0,15031 | 0,457707942 |
| Regulation of cytoplasmic and nuclear SMAD2/3 signaling | 303 | 305 | 4,96152 | 1,023212 | 0,005419 | 1 | 0,161398 | 0,49147007 |
| TGF-beta receptor signaling | 303 | 305 | 4,96152 | 1,023212 | 0,005419 | 1 | 0,161398 | 0,49147007 |
| Regulation of nuclear SMAD2/3 signaling | 303 | 305 | 4,96152 | 1,023212 | 0,005419 | 1 | 0,161398 | 0,49147007 |
| Integrin-linked kinase signaling | 645 | 654 | 10,56165 | 1,015792 | 0,005774 | 1 | 0,16896 | 0,514498703 |
| TNF receptor signaling pathway | 297 | 299 | 4,863272 | 1,023076 | 0,006283 | 1 | 0,180177 | 0,548654379 |
| Metabolism of mRNA | 233 | 234 | 3,815294 | 1,025564 | 0,006373 | 1 | 0,180177 | 0,548654379 |
| Regulation of p38-alpha and p38-beta | 164 | 164 | 2,685443 | 1,029966 | 0,007395 | 1 | 0,205574 | 0,625990487 |
| Axon guidance | 218 | 219 | 3,569674 | 1,025263 | 0,009421 | 1 | 0,257609 | 0,784444052 |
| Diabetes pathways | 215 | 216 | 3,52055 | 1,025197 | 0,01018 | 1 | 0,273875 | 0,83397404 |
| AP-1 transcription factor network | 612 | 621 | 10,02129 | 1,015039 | 0,010518 | 1 | 0,27848 | 0,847997722 |
| Mitotic G1-G1/S phases | 140 | 140 | 2,292451 | 1,029966 | 0,015289 | 1 | 0,39847 | 1 |
| Platelet activation, signaling and aggregation | 138 | 138 | 2,259702 | 1,029966 | 0,016241 | 1 | 0,416768 | 1 |
| Validated transcriptional targets of AP1 family members Fra1 and Fra2 | 134 | 134 | 2,194203 | 1,029966 | 0,018325 | 1 | 0,463128 | 1 |
| p38 MAPK signaling pathway | 188 | 189 | 3,078435 | 1,024516 | 0,020214 | 1 | 0,503234 | 1 |
| FAS (CD95) signaling pathway | 130 | 130 | 2,128705 | 1,029966 | 0,020675 | 1 | 0,507153 | 1 |
| TGFBR | 125 | 125 | 2,046832 | 1,029966 | 0,024038 | 1 | 0,581104 | 1 |
| Immune System | 514 | 522 | 8,416571 | 1,014181 | 0,027055 | 1 | 0,628196 | 1 |
| Transmission across Chemical Synapses | 121 | 121 | 1,981333 | 1,029966 | 0,027116 | 1 | 0,628196 | 1 |
| S Phase | 121 | 121 | 1,981333 | 1,029966 | 0,027116 | 1 | 0,628196 | 1 |
| Innate Immune System | 182 | 183 | 2,980187 | 1,024338 | 0,027622 | 1 | 0,631146 | 1 |
| IL1-mediated signaling events | 232 | 234 | 3,798919 | 1,021163 | 0,029374 | 1 | 0,662106 | 1 |
| G1/S Transition | 117 | 117 | 1,915834 | 1,029966 | 0,030586 | 1 | 0,671282 | 1 |
| Translation | 117 | 117 | 1,915834 | 1,029966 | 0,030586 | 1 | 0,671282 | 1 |
| IL2-mediated signaling events | 115 | 115 | 1,883085 | 1,029966 | 0,032483 | 1 | 0,698076 | 1 |
| Metabolism of RNA | 278 | 281 | 4,552153 | 1,01897 | 0,032644 | 1 | 0,698076 | 1 |
| Post-translational protein modification | 114 | 114 | 1,86671 | 1,029966 | 0,033475 | 1 | 0,706784 | 1 |
| BMP receptor signaling | 224 | 226 | 3,667922 | 1,020851 | 0,035196 | 1 | 0,733842 | 1 |
| Eukaryotic Translation Initiation | 110 | 110 | 1,801212 | 1,029966 | 0,037753 | 1 | 0,767946 | 1 |
| Cap-dependent Translation Initiation | 110 | 110 | 1,801212 | 1,029966 | 0,037753 | 1 | 0,767946 | 1 |
| Synthesis of DNA | 109 | 109 | 1,784837 | 1,029966 | 0,038905 | 1 | 0,781842 | 1 |
| Regulation of beta-cell development | 108 | 108 | 1,768462 | 1,029966 | 0,040091 | 1 | 0,79531 | 1 |
| mRNA Splicing - Major Pathway | 107 | 107 | 1,752088 | 1,029966 | 0,041314 | 1 | 0,79531 | 1 |
| mRNA Splicing | 107 | 107 | 1,752088 | 1,029966 | 0,041314 | 1 | 0,79531 | 1 |
| Peptide ligand-binding receptors | 166 | 167 | 2,718192 | 1,023798 | 0,041482 | 1 | 0,79531 | 1 |
| Nonsense-Mediated Decay | 104 | 104 | 1,702964 | 1,029966 | 0,04521 | 1 | 0,837884 | 1 |
| Regulation of mRNA Stability by Proteins that Bind AU-rich Elements | 104 | 104 | 1,702964 | 1,029966 | 0,04521 | 1 | 0,837884 | 1 |
| Nonsense Mediated Decay Enhanced by the Exon Junction Complex | 104 | 104 | 1,702964 | 1,029966 | 0,04521 | 1 | 0,837884 | 1 |
| GTP hydrolysis and joining of the 60S ribosomal subunit | 103 | 103 | 1,686589 | 1,029966 | 0,046588 | 1 | 0,853934 | 1 |
| L13a-mediated translational silencing of Ceruloplasmin expression | 102 | 102 | 1,670215 | 1,029966 | 0,048007 | 1 | 0,861033 | 1 |
| 3' -UTR-mediated translational regulation | 102 | 102 | 1,670215 | 1,029966 | 0,048007 | 1 | 0,861033 | 1 |
| Neurotrophic factor-mediated Trk receptor signaling | 101 | 101 | 1,65384 | 1,029966 | 0,04947 | 1 | 0,87783 | 1 |
| LPA receptor mediated events | 100 | 100 | 1,637465 | 1,029966 | 0,050977 | 1 | 0,885178 | 1 |
| Apoptosis | 157 | 158 | 2,57082 | 1,023447 | 0,051971 | 1 | 0,885178 | 1 |
| Olfactory Signaling Pathway | 344 | 349 | 5,63288 | 1,01521 | 0,053936 | 1 | 0,885178 | 1 |
| Regulation of gene expression in beta cells | 98 | 98 | 1,604716 | 1,029966 | 0,05413 | 1 | 0,885178 | 1 |
| DNA Replication Pre-Initiation | 98 | 98 | 1,604716 | 1,029966 | 0,05413 | 1 | 0,885178 | 1 |
| M/G1 Transition | 98 | 98 | 1,604716 | 1,029966 | 0,05413 | 1 | 0,885178 | 1 |
| Influenza Viral RNA Transcription and Replication | 98 | 98 | 1,604716 | 1,029966 | 0,05413 | 1 | 0,885178 | 1 |
| Biological oxidations | 98 | 98 | 1,604716 | 1,029966 | 0,05413 | 1 | 0,885178 | 1 |
| Role of Calcineurin-dependent NFAT signaling in lymphocytes | 95 | 95 | 1,555592 | 1,029966 | 0,059225 | 1 | 0,95135 | 1 |
| Transport of inorganic cations/anions and amino acids/oligopeptides | 94 | 94 | 1,539217 | 1,029966 | 0,061028 | 1 | 0,95135 | 1 |
| Transport of glucose and other sugars, bile salts and organic acids, metal ions and amine compounds | 94 | 94 | 1,539217 | 1,029966 | 0,061028 | 1 | 0,95135 | 1 |
| Notch-mediated HES/HEY network | 94 | 94 | 1,539217 | 1,029966 | 0,061028 | 1 | 0,95135 | 1 |
| Notch signaling pathway | 94 | 94 | 1,539217 | 1,029966 | 0,061028 | 1 | 0,95135 | 1 |
| Formation of a pool of free 40S subunits | 92 | 92 | 1,506468 | 1,029966 | 0,064798 | 1 | 0,990745 | 1 |
| Signaling events mediated by TCPTP | 92 | 92 | 1,506468 | 1,029966 | 0,064798 | 1 | 0,990745 | 1 |
| SLC-mediated transmembrane transport | 244 | 247 | 3,995415 | 1,017456 | 0,065337 | 1 | 0,990745 | 1 |
| Cyclin A:Cdk2-associated events at S phase entry | 90 | 90 | 1,473719 | 1,029966 | 0,0688 | 1 | 1 | 1 |
| Neurotransmitter Receptor Binding And Downstream Transmission In The Postsynaptic Cell | 89 | 89 | 1,457344 | 1,029966 | 0,070892 | 1 | 1 | 1 |
| Cytokine Signaling in Immune system | 191 | 193 | 3,127559 | 1,019293 | 0,072291 | 1 | 1 | 1 |
| Regulation of DNA replication | 88 | 88 | 1,440969 | 1,029966 | 0,073047 | 1 | 1 | 1 |
| Nonsense Mediated Decay Independent of the Exon Junction Complex | 87 | 87 | 1,424595 | 1,029966 | 0,075268 | 1 | 1 | 1 |
| Removal of licensing factors from origins | 85 | 85 | 1,391845 | 1,029966 | 0,079912 | 1 | 1 | 1 |
| Eukaryotic Translation Elongation | 85 | 85 | 1,391845 | 1,029966 | 0,079912 | 1 | 1 | 1 |
| Membrane Trafficking | 84 | 84 | 1,375471 | 1,029966 | 0,08234 | 1 | 1 | 1 |
| Assembly of the pre-replicative complex | 83 | 83 | 1,359096 | 1,029966 | 0,084842 | 1 | 1 | 1 |
| Switching of origins to a post-replicative state | 83 | 83 | 1,359096 | 1,029966 | 0,084842 | 1 | 1 | 1 |
| Orc1 removal from chromatin | 83 | 83 | 1,359096 | 1,029966 | 0,084842 | 1 | 1 | 1 |
| Integration of energy metabolism | 83 | 83 | 1,359096 | 1,029966 | 0,084842 | 1 | 1 | 1 |
| Direct p53 effectors | 137 | 138 | 2,243327 | 1,022503 | 0,084943 | 1 | 1 | 1 |
| EGFR1 | 137 | 138 | 2,243327 | 1,022503 | 0,084943 | 1 | 1 | 1 |
| Eukaryotic Translation Termination | 82 | 82 | 1,342721 | 1,029966 | 0,087419 | 1 | 1 | 1 |
| Peptide chain elongation | 82 | 82 | 1,342721 | 1,029966 | 0,087419 | 1 | 1 | 1 |
| Viral mRNA Translation | 82 | 82 | 1,342721 | 1,029966 | 0,087419 | 1 | 1 | 1 |
| Wnt | 82 | 82 | 1,342721 | 1,029966 | 0,087419 | 1 | 1 | 1 |
| Regulation of mitotic cell cycle | 81 | 81 | 1,326347 | 1,029966 | 0,090074 | 1 | 1 | 1 |
| APC/C-mediated degradation of cell cycle proteins | 81 | 81 | 1,326347 | 1,029966 | 0,090074 | 1 | 1 | 1 |
| ER-Phagosome pathway | 80 | 80 | 1,309972 | 1,029966 | 0,092808 | 1 | 1 | 1 |
| L1CAM interactions | 79 | 79 | 1,293598 | 1,029966 | 0,095626 | 1 | 1 | 1 |
| Signaling by Insulin receptor | 79 | 79 | 1,293598 | 1,029966 | 0,095626 | 1 | 1 | 1 |
| Insulin Synthesis and Processing | 131 | 132 | 2,145079 | 1,022163 | 0,098146 | 1 | 1 | 1 |
| Regulation of APC/C activators between G1/S and early anaphase | 78 | 78 | 1,277223 | 1,029966 | 0,098529 | 1 | 1 | 1 |
| Cyclin E associated events during G1/S transition | 78 | 78 | 1,277223 | 1,029966 | 0,098529 | 1 | 1 | 1 |
| Asparagine N-linked glycosylation | 77 | 77 | 1,260848 | 1,029966 | 0,101519 | 1 | 1 | 1 |
| Interferon alpha/beta signaling | 77 | 77 | 1,260848 | 1,029966 | 0,101519 | 1 | 1 | 1 |
| Androgen-mediated signaling | 129 | 130 | 2,11233 | 1,022043 | 0,102955 | 1 | 1 | 1 |
| p75(NTR)-mediated signaling | 176 | 178 | 2,881939 | 1,018394 | 0,103505 | 1 | 1 | 1 |
| Mesenchymal-to-epithelial transition | 220 | 223 | 3,602423 | 1,01611 | 0,103667 | 1 | 1 | 1 |
| Potassium Channels | 76 | 76 | 1,244474 | 1,029966 | 0,104599 | 1 | 1 | 1 |
| Signaling by Wnt | 75 | 75 | 1,228099 | 1,029966 | 0,107772 | 1 | 1 | 1 |
| Degradation of beta-catenin by the destruction complex | 75 | 75 | 1,228099 | 1,029966 | 0,107772 | 1 | 1 | 1 |
| Regulation of Apoptosis | 75 | 75 | 1,228099 | 1,029966 | 0,107772 | 1 | 1 | 1 |
| TCR signaling in na&#xef;ve CD8+ T cells | 126 | 127 | 2,063206 | 1,021856 | 0,110578 | 1 | 1 | 1 |
| CDT1 association with the CDC6:ORC:origin complex | 74 | 74 | 1,211724 | 1,029966 | 0,111042 | 1 | 1 | 1 |
| E2F transcription factor network | 73 | 73 | 1,19535 | 1,029966 | 0,114409 | 1 | 1 | 1 |
| Activation of APC/C and APC/C:Cdc20 mediated degradation of mitotic proteins | 72 | 72 | 1,178975 | 1,029966 | 0,117878 | 1 | 1 | 1 |
| APC/C:Cdc20 mediated degradation of mitotic proteins | 71 | 71 | 1,1626 | 1,029966 | 0,121452 | 1 | 1 | 1 |
| Destabilization of mRNA by AUF1 (hnRNP D0) | 71 | 71 | 1,1626 | 1,029966 | 0,121452 | 1 | 1 | 1 |
| SCF-beta-TrCP mediated degradation of Emi1 | 70 | 70 | 1,146226 | 1,029966 | 0,125134 | 1 | 1 | 1 |
| p53-Independent G1/S DNA damage checkpoint | 70 | 70 | 1,146226 | 1,029966 | 0,125134 | 1 | 1 | 1 |
| p53-Independent DNA Damage Response | 70 | 70 | 1,146226 | 1,029966 | 0,125134 | 1 | 1 | 1 |
| Regulation of ornithine decarboxylase (ODC) | 70 | 70 | 1,146226 | 1,029966 | 0,125134 | 1 | 1 | 1 |
| Transcriptional Regulation of White Adipocyte Differentiation | 69 | 69 | 1,129851 | 1,029966 | 0,128926 | 1 | 1 | 1 |
| APC/C:Cdh1 mediated degradation of Cdc20 and other APC/C:Cdh1 targeted proteins in late mitosis/early G1 | 69 | 69 | 1,129851 | 1,029966 | 0,128926 | 1 | 1 | 1 |
| Ubiquitin Mediated Degradation of Phosphorylated Cdc25A | 69 | 69 | 1,129851 | 1,029966 | 0,128926 | 1 | 1 | 1 |
| Antigen processing: Ubiquitination & Proteasome degradation | 69 | 69 | 1,129851 | 1,029966 | 0,128926 | 1 | 1 | 1 |
| Cdc20:Phospho-APC/C mediated degradation of Cyclin A | 69 | 69 | 1,129851 | 1,029966 | 0,128926 | 1 | 1 | 1 |
| Signaling events regulated by Ret tyrosine kinase | 69 | 69 | 1,129851 | 1,029966 | 0,128926 | 1 | 1 | 1 |
| G alpha (q) signalling events | 119 | 120 | 1,948584 | 1,021383 | 0,13043 | 1 | 1 | 1 |
| Cell Cycle Checkpoints | 117 | 118 | 1,915834 | 1,021238 | 0,136673 | 1 | 1 | 1 |
| Vpu mediated degradation of CD4 | 67 | 67 | 1,097102 | 1,029966 | 0,136858 | 1 | 1 | 1 |
| SCF(Skp2)-mediated degradation of p27/p21 | 67 | 67 | 1,097102 | 1,029966 | 0,136858 | 1 | 1 | 1 |
| RIG-I/MDA5 mediated induction of IFN-alpha/beta pathways | 67 | 67 | 1,097102 | 1,029966 | 0,136858 | 1 | 1 | 1 |
| BCR signaling pathway | 67 | 67 | 1,097102 | 1,029966 | 0,136858 | 1 | 1 | 1 |
| IL2 signaling events mediated by PI3K | 67 | 67 | 1,097102 | 1,029966 | 0,136858 | 1 | 1 | 1 |
| N-cadherin signaling events | 247 | 251 | 4,044539 | 1,013552 | 0,137472 | 1 | 1 | 1 |
| APC/C:Cdc20 mediated degradation of Securin | 66 | 66 | 1,080727 | 1,029966 | 0,141004 | 1 | 1 | 1 |
| Ubiquitin-dependent degradation of Cyclin D | 66 | 66 | 1,080727 | 1,029966 | 0,141004 | 1 | 1 | 1 |
| Ubiquitin-dependent degradation of Cyclin D1 | 66 | 66 | 1,080727 | 1,029966 | 0,141004 | 1 | 1 | 1 |
| CDK-mediated phosphorylation and removal of Cdc6 | 66 | 66 | 1,080727 | 1,029966 | 0,141004 | 1 | 1 | 1 |
| Regulation of activated PAK-2p34 by proteasome mediated degradation | 66 | 66 | 1,080727 | 1,029966 | 0,141004 | 1 | 1 | 1 |
| Regulation of retinoblastoma protein | 66 | 66 | 1,080727 | 1,029966 | 0,141004 | 1 | 1 | 1 |
| IL23-mediated signaling events | 66 | 66 | 1,080727 | 1,029966 | 0,141004 | 1 | 1 | 1 |
| Regulation of RAC1 activity | 202 | 205 | 3,30768 | 1,014894 | 0,143958 | 1 | 1 | 1 |
| RAC1 signaling pathway | 202 | 205 | 3,30768 | 1,014894 | 0,143958 | 1 | 1 | 1 |
| RhoA signaling pathway | 202 | 205 | 3,30768 | 1,014894 | 0,143958 | 1 | 1 | 1 |
| Regulation of RhoA activity | 202 | 205 | 3,30768 | 1,014894 | 0,143958 | 1 | 1 | 1 |
| Downstream signaling in na&#xef;ve CD8+ T cells | 65 | 65 | 1,064352 | 1,029966 | 0,145274 | 1 | 1 | 1 |
| Semaphorin interactions | 64 | 64 | 1,047978 | 1,029966 | 0,149674 | 1 | 1 | 1 |
| Integrins in angiogenesis | 64 | 64 | 1,047978 | 1,029966 | 0,149674 | 1 | 1 | 1 |
| Aurora A signaling | 64 | 64 | 1,047978 | 1,029966 | 0,149674 | 1 | 1 | 1 |
| Trk receptor signaling mediated by PI3K and PLC-gamma | 63 | 63 | 1,031603 | 1,029966 | 0,154206 | 1 | 1 | 1 |
| mRNA Processing | 155 | 157 | 2,538071 | 1,016846 | 0,158556 | 1 | 1 | 1 |
| IL4-mediated signaling events | 62 | 62 | 1,015228 | 1,029966 | 0,158874 | 1 | 1 | 1 |
| IL12-mediated signaling events | 110 | 111 | 1,801212 | 1,020687 | 0,160724 | 1 | 1 | 1 |
| Coregulation of Androgen receptor activity | 61 | 61 | 0,998854 | 1,029966 | 0,163683 | 1 | 1 | 1 |
| Polo-like kinase signaling events in the cell cycle | 108 | 109 | 1,768462 | 1,020517 | 0,168264 | 1 | 1 | 1 |
| Autodegradation of Cdh1 by Cdh1:APC/C | 60 | 60 | 0,982479 | 1,029966 | 0,168636 | 1 | 1 | 1 |
| Phase 1 - Functionalization of compounds | 60 | 60 | 0,982479 | 1,029966 | 0,168636 | 1 | 1 | 1 |
| EphrinB-EPHB pathway | 60 | 60 | 0,982479 | 1,029966 | 0,168636 | 1 | 1 | 1 |
| Regulation of Androgen receptor activity | 107 | 108 | 1,752088 | 1,02043 | 0,172152 | 1 | 1 | 1 |
| NGF signalling via TRKA from the plasma membrane | 106 | 107 | 1,735713 | 1,020341 | 0,17612 | 1 | 1 | 1 |
| Cell Cycle, Mitotic | 311 | 317 | 5,092517 | 1,010472 | 0,176209 | 1 | 1 | 1 |
| Metabolism of nucleotides | 58 | 58 | 0,94973 | 1,029966 | 0,178995 | 1 | 1 | 1 |
| MyD88-independent cascade initiated on plasma membrane | 58 | 58 | 0,94973 | 1,029966 | 0,178995 | 1 | 1 | 1 |
| CD40/CD40L signaling | 58 | 58 | 0,94973 | 1,029966 | 0,178995 | 1 | 1 | 1 |
| NCAM signaling for neurite out-growth | 57 | 57 | 0,933355 | 1,029966 | 0,184409 | 1 | 1 | 1 |
| Endogenous TLR signaling | 57 | 57 | 0,933355 | 1,029966 | 0,184409 | 1 | 1 | 1 |
| C-MYC pathway | 147 | 149 | 2,407074 | 1,016141 | 0,185378 | 1 | 1 | 1 |
| PLK1 signaling events | 103 | 104 | 1,686589 | 1,020063 | 0,188519 | 1 | 1 | 1 |
| Posttranslational regulation of adherens junction stability and dissassembly | 227 | 231 | 3,717046 | 1,012131 | 0,189804 | 1 | 1 | 1 |
| p53 pathway | 186 | 189 | 3,045685 | 1,013618 | 0,189938 | 1 | 1 | 1 |
| Insulin receptor signalling cascade | 56 | 56 | 0,916981 | 1,029966 | 0,189986 | 1 | 1 | 1 |
| TRIF mediated TLR3 signaling | 56 | 56 | 0,916981 | 1,029966 | 0,189986 | 1 | 1 | 1 |
| Toll Like Receptor 3 (TLR3) Cascade | 56 | 56 | 0,916981 | 1,029966 | 0,189986 | 1 | 1 | 1 |
| Metabolism of amino acids and derivatives | 185 | 188 | 3,029311 | 1,013531 | 0,193161 | 1 | 1 | 1 |
| Activation of the mRNA upon binding of the cap-binding complex and eIFs, and subsequent binding to 43S | 55 | 55 | 0,900606 | 1,029966 | 0,195731 | 1 | 1 | 1 |
| Ribosomal scanning and start codon recognition | 54 | 54 | 0,884231 | 1,029966 | 0,201648 | 1 | 1 | 1 |
| Translation initiation complex formation | 54 | 54 | 0,884231 | 1,029966 | 0,201648 | 1 | 1 | 1 |
| NFkB and MAP kinases activation mediated by TLR4 signaling repertoire | 54 | 54 | 0,884231 | 1,029966 | 0,201648 | 1 | 1 | 1 |
| Validated transcriptional targets of TAp63 isoforms | 54 | 54 | 0,884231 | 1,029966 | 0,201648 | 1 | 1 | 1 |
| KitReceptor | 54 | 54 | 0,884231 | 1,029966 | 0,201648 | 1 | 1 | 1 |
| Class I MHC mediated antigen processing & presentation | 100 | 101 | 1,637465 | 1,019769 | 0,201685 | 1 | 1 | 1 |
| IRS-related events | 53 | 53 | 0,867857 | 1,029966 | 0,207744 | 1 | 1 | 1 |
| Signalling by NGF | 141 | 143 | 2,308826 | 1,015561 | 0,207917 | 1 | 1 | 1 |
| Mitotic G2-G2/M phases | 98 | 99 | 1,604716 | 1,019563 | 0,210906 | 1 | 1 | 1 |
| Costimulation by the CD28 family | 52 | 52 | 0,851482 | 1,029966 | 0,214023 | 1 | 1 | 1 |
| Caspase cascade in apoptosis | 52 | 52 | 0,851482 | 1,029966 | 0,214023 | 1 | 1 | 1 |
| Signaling events mediated by PTP1B | 52 | 52 | 0,851482 | 1,029966 | 0,214023 | 1 | 1 | 1 |
| Signaling by EGFR | 97 | 98 | 1,588341 | 1,019457 | 0,215654 | 1 | 1 | 1 |
| Interferon Signaling | 97 | 98 | 1,588341 | 1,019457 | 0,215654 | 1 | 1 | 1 |
| Influenza Infection | 138 | 140 | 2,259702 | 1,015253 | 0,22001 | 1 | 1 | 1 |
| IRS-mediated signalling | 51 | 51 | 0,835107 | 1,029966 | 0,22049 | 1 | 1 | 1 |
| G2/M Transition | 95 | 96 | 1,555592 | 1,019238 | 0,225429 | 1 | 1 | 1 |
| Muscle contraction | 50 | 50 | 0,818733 | 1,029966 | 0,227152 | 1 | 1 | 1 |
| TRAF6 Mediated Induction of proinflammatory cytokines | 50 | 50 | 0,818733 | 1,029966 | 0,227152 | 1 | 1 | 1 |
| Signaling mediated by p38-alpha and p38-beta | 50 | 50 | 0,818733 | 1,029966 | 0,227152 | 1 | 1 | 1 |
| Angiopoietin receptor Tie2-mediated signaling | 50 | 50 | 0,818733 | 1,029966 | 0,227152 | 1 | 1 | 1 |
| Processing of Capped Intron-Containing Pre-mRNA | 136 | 138 | 2,226953 | 1,01504 | 0,228387 | 1 | 1 | 1 |
| Signaling by FGFR | 94 | 95 | 1,539217 | 1,019125 | 0,23046 | 1 | 1 | 1 |
| Nucleotide Excision Repair | 49 | 49 | 0,802358 | 1,029966 | 0,234013 | 1 | 1 | 1 |
| Calcineurin-regulated NFAT-dependent transcription in lymphocytes | 49 | 49 | 0,802358 | 1,029966 | 0,234013 | 1 | 1 | 1 |
| Ceramide signaling pathway | 49 | 49 | 0,802358 | 1,029966 | 0,234013 | 1 | 1 | 1 |
| Amino acid and oligopeptide SLC transporters | 48 | 48 | 0,785983 | 1,029966 | 0,241081 | 1 | 1 | 1 |
| Regulation of Insulin Secretion | 48 | 48 | 0,785983 | 1,029966 | 0,241081 | 1 | 1 | 1 |
| Cytochrome P450 - arranged by substrate type | 48 | 48 | 0,785983 | 1,029966 | 0,241081 | 1 | 1 | 1 |
| FGF signaling pathway | 48 | 48 | 0,785983 | 1,029966 | 0,241081 | 1 | 1 | 1 |
| Hedgehog signaling events mediated by Gli proteins | 48 | 48 | 0,785983 | 1,029966 | 0,241081 | 1 | 1 | 1 |
| Alpha6Beta4Integrin | 48 | 48 | 0,785983 | 1,029966 | 0,241081 | 1 | 1 | 1 |
| Influenza Life Cycle | 133 | 135 | 2,177829 | 1,014708 | 0,241435 | 1 | 1 | 1 |
| Signaling by Interleukins | 91 | 92 | 1,490093 | 1,018772 | 0,246138 | 1 | 1 | 1 |
| Class B/2 (Secretin family receptors) | 47 | 47 | 0,769609 | 1,029966 | 0,248361 | 1 | 1 | 1 |
| Formation of the ternary complex, and subsequently, the 43S complex | 47 | 47 | 0,769609 | 1,029966 | 0,248361 | 1 | 1 | 1 |
| Metabolism of vitamins and cofactors | 47 | 47 | 0,769609 | 1,029966 | 0,248361 | 1 | 1 | 1 |
| Metabolism of water-soluble vitamins and cofactors | 47 | 47 | 0,769609 | 1,029966 | 0,248361 | 1 | 1 | 1 |
| IL6-mediated signaling events | 47 | 47 | 0,769609 | 1,029966 | 0,248361 | 1 | 1 | 1 |
| Retinoic acid receptors-mediated signaling | 47 | 47 | 0,769609 | 1,029966 | 0,248361 | 1 | 1 | 1 |
| TCR signaling in na&#xef;ve CD4+ T cells | 131 | 133 | 2,145079 | 1,014479 | 0,250461 | 1 | 1 | 1 |
| Ion channel transport | 46 | 46 | 0,753234 | 1,029966 | 0,25586 | 1 | 1 | 1 |
| Toll Receptor Cascades | 89 | 90 | 1,457344 | 1,018523 | 0,257091 | 1 | 1 | 1 |
| Syndecan-4-mediated signaling events | 205 | 209 | 3,356804 | 1,010254 | 0,263978 | 1 | 1 | 1 |
| Transcription-coupled NER (TC-NER) | 44 | 44 | 0,720485 | 1,029966 | 0,27154 | 1 | 1 | 1 |
| Glypican 3 network | 202 | 206 | 3,30768 | 1,009967 | 0,275513 | 1 | 1 | 1 |
| Post-Elongation Processing of the Transcript | 43 | 43 | 0,70411 | 1,029966 | 0,279734 | 1 | 1 | 1 |
| Cleavage of Growing Transcript in the Termination Region | 43 | 43 | 0,70411 | 1,029966 | 0,279734 | 1 | 1 | 1 |
| RNA Polymerase II Transcription Termination | 43 | 43 | 0,70411 | 1,029966 | 0,279734 | 1 | 1 | 1 |
| G2/M Checkpoints | 43 | 43 | 0,70411 | 1,029966 | 0,279734 | 1 | 1 | 1 |
| EphrinA-EPHA pathway | 43 | 43 | 0,70411 | 1,029966 | 0,279734 | 1 | 1 | 1 |
| Antigen processing-Cross presentation | 85 | 86 | 1,391845 | 1,017991 | 0,280241 | 1 | 1 | 1 |
| E-cadherin signaling events | 274 | 280 | 4,486655 | 1,007896 | 0,286159 | 1 | 1 | 1 |
| Glucocorticoid receptor signaling | 84 | 85 | 1,375471 | 1,01785 | 0,286294 | 1 | 1 | 1 |
| mRNA Splicing - Minor Pathway | 42 | 42 | 0,687735 | 1,029966 | 0,288175 | 1 | 1 | 1 |
| Ras signaling in the CD4+ TCR pathway | 42 | 42 | 0,687735 | 1,029966 | 0,288175 | 1 | 1 | 1 |
| JNK signaling in the CD4+ TCR pathway | 42 | 42 | 0,687735 | 1,029966 | 0,288175 | 1 | 1 | 1 |
| C-MYB transcription factor network | 83 | 84 | 1,359096 | 1,017706 | 0,292456 | 1 | 1 | 1 |
| Wnt signaling network | 196 | 200 | 3,209432 | 1,009367 | 0,29961 | 1 | 1 | 1 |
| Stabilization and expansion of the E-cadherin adherens junction | 269 | 275 | 4,404781 | 1,007494 | 0,303967 | 1 | 1 | 1 |
| E-cadherin signaling in the nascent adherens junction | 269 | 275 | 4,404781 | 1,007494 | 0,303967 | 1 | 1 | 1 |
| EPHB forward signaling | 40 | 40 | 0,654986 | 1,029966 | 0,305823 | 1 | 1 | 1 |
| Validated targets of C-MYC transcriptional activation | 80 | 81 | 1,309972 | 1,017252 | 0,311603 | 1 | 1 | 1 |
| Response to elevated platelet cytosolic Ca2+ | 39 | 39 | 0,638611 | 1,029966 | 0,315046 | 1 | 1 | 1 |
| Voltage gated Potassium channels | 39 | 39 | 0,638611 | 1,029966 | 0,315046 | 1 | 1 | 1 |
| HIV-1 Transcription Initiation | 39 | 39 | 0,638611 | 1,029966 | 0,315046 | 1 | 1 | 1 |
| RNA Polymerase II HIV-1 Promoter Escape | 39 | 39 | 0,638611 | 1,029966 | 0,315046 | 1 | 1 | 1 |
| RNA Polymerase II Transcription Initiation And Promoter Clearance | 39 | 39 | 0,638611 | 1,029966 | 0,315046 | 1 | 1 | 1 |
| RNA Polymerase II Transcription Initiation | 39 | 39 | 0,638611 | 1,029966 | 0,315046 | 1 | 1 | 1 |
| RNA Polymerase II Transcription Pre-Initiation And Promoter Opening | 39 | 39 | 0,638611 | 1,029966 | 0,315046 | 1 | 1 | 1 |
| RNA Polymerase II Promoter Escape | 39 | 39 | 0,638611 | 1,029966 | 0,315046 | 1 | 1 | 1 |
| Phase II conjugation | 39 | 39 | 0,638611 | 1,029966 | 0,315046 | 1 | 1 | 1 |
| Apoptotic cleavage of cellular proteins | 39 | 39 | 0,638611 | 1,029966 | 0,315046 | 1 | 1 | 1 |
| Glucocorticoid receptor regulatory network | 79 | 80 | 1,293598 | 1,017093 | 0,318209 | 1 | 1 | 1 |
| PI3K Cascade | 38 | 38 | 0,622237 | 1,029966 | 0,324546 | 1 | 1 | 1 |
| Regulation of IFNA signaling | 38 | 38 | 0,622237 | 1,029966 | 0,324546 | 1 | 1 | 1 |
| ErbB2/ErbB3 signaling events | 38 | 38 | 0,622237 | 1,029966 | 0,324546 | 1 | 1 | 1 |
| Signaling by PDGF | 77 | 78 | 1,260848 | 1,016763 | 0,331762 | 1 | 1 | 1 |
| AndrogenReceptor | 77 | 78 | 1,260848 | 1,016763 | 0,331762 | 1 | 1 | 1 |
| Activation of ATR in response to replication stress | 37 | 37 | 0,605862 | 1,029966 | 0,33433 | 1 | 1 | 1 |
| Iron uptake and transport | 37 | 37 | 0,605862 | 1,029966 | 0,33433 | 1 | 1 | 1 |
| CXCR3-mediated signaling events | 37 | 37 | 0,605862 | 1,029966 | 0,33433 | 1 | 1 | 1 |
| Syndecan-2-mediated signaling events | 76 | 77 | 1,244474 | 1,016591 | 0,33871 | 1 | 1 | 1 |
| CXCR4-mediated signaling events | 186 | 190 | 3,045685 | 1,008283 | 0,342748 | 1 | 1 | 1 |
| GABA receptor activation | 36 | 36 | 0,589487 | 1,029966 | 0,344408 | 1 | 1 | 1 |
| Amine ligand-binding receptors | 36 | 36 | 0,589487 | 1,029966 | 0,344408 | 1 | 1 | 1 |
| Glucose metabolism | 36 | 36 | 0,589487 | 1,029966 | 0,344408 | 1 | 1 | 1 |
| CD28 co-stimulation | 36 | 36 | 0,589487 | 1,029966 | 0,344408 | 1 | 1 | 1 |
| G1/S DNA Damage Checkpoints | 75 | 76 | 1,228099 | 1,016415 | 0,345774 | 1 | 1 | 1 |
| Toll Like Receptor 4 (TLR4) Cascade | 75 | 76 | 1,228099 | 1,016415 | 0,345774 | 1 | 1 | 1 |
| Downstream signal transduction | 74 | 75 | 1,211724 | 1,016235 | 0,352953 | 1 | 1 | 1 |
| Signal transduction by L1 | 35 | 35 | 0,573113 | 1,029966 | 0,354788 | 1 | 1 | 1 |
| Generic Transcription Pathway | 35 | 35 | 0,573113 | 1,029966 | 0,354788 | 1 | 1 | 1 |
| Canonical NF-kappaB pathway | 35 | 35 | 0,573113 | 1,029966 | 0,354788 | 1 | 1 | 1 |
| HIV-1 Nef: Negative effector of Fas and TNF-alpha | 35 | 35 | 0,573113 | 1,029966 | 0,354788 | 1 | 1 | 1 |
| a6b1 and a6b4 Integrin signaling | 35 | 35 | 0,573113 | 1,029966 | 0,354788 | 1 | 1 | 1 |
| DNA Replication | 255 | 261 | 4,175536 | 1,006289 | 0,357435 | 1 | 1 | 1 |
| Post-Elongation Processing of Intron-Containing pre-mRNA | 34 | 34 | 0,556738 | 1,029966 | 0,365479 | 1 | 1 | 1 |
| mRNA 3'-end processing | 34 | 34 | 0,556738 | 1,029966 | 0,365479 | 1 | 1 | 1 |
| Activation of NMDA receptor upon glutamate binding and postsynaptic events | 34 | 34 | 0,556738 | 1,029966 | 0,365479 | 1 | 1 | 1 |
| Post NMDA receptor activation events | 34 | 34 | 0,556738 | 1,029966 | 0,365479 | 1 | 1 | 1 |
| RNA Polymerase III Transcription | 34 | 34 | 0,556738 | 1,029966 | 0,365479 | 1 | 1 | 1 |
| RNA Polymerase III Abortive And Retractive Initiation | 34 | 34 | 0,556738 | 1,029966 | 0,365479 | 1 | 1 | 1 |
| EPO signaling pathway | 34 | 34 | 0,556738 | 1,029966 | 0,365479 | 1 | 1 | 1 |
| IL8- and CXCR2-mediated signaling events | 34 | 34 | 0,556738 | 1,029966 | 0,365479 | 1 | 1 | 1 |
| Trk receptor signaling mediated by the MAPK pathway | 34 | 34 | 0,556738 | 1,029966 | 0,365479 | 1 | 1 | 1 |
| Cell surface interactions at the vascular wall | 71 | 72 | 1,1626 | 1,015662 | 0,375196 | 1 | 1 | 1 |
| Integrin cell surface interactions | 71 | 72 | 1,1626 | 1,015662 | 0,375196 | 1 | 1 | 1 |
| p53-Dependent G1 DNA Damage Response | 71 | 72 | 1,1626 | 1,015662 | 0,375196 | 1 | 1 | 1 |
| p53-Dependent G1/S DNA damage checkpoint | 71 | 72 | 1,1626 | 1,015662 | 0,375196 | 1 | 1 | 1 |
| Activated TLR4 signalling | 71 | 72 | 1,1626 | 1,015662 | 0,375196 | 1 | 1 | 1 |
| Formation of Fibrin Clot (Clotting Cascade) | 33 | 33 | 0,540364 | 1,029966 | 0,37649 | 1 | 1 | 1 |
| Global Genomic NER (GG-NER) | 33 | 33 | 0,540364 | 1,029966 | 0,37649 | 1 | 1 | 1 |
| trans-Golgi Network Vesicle Budding | 33 | 33 | 0,540364 | 1,029966 | 0,37649 | 1 | 1 | 1 |
| Clathrin derived vesicle budding | 33 | 33 | 0,540364 | 1,029966 | 0,37649 | 1 | 1 | 1 |
| Glucagon signaling in metabolic regulation | 33 | 33 | 0,540364 | 1,029966 | 0,37649 | 1 | 1 | 1 |
| Noncanonical Wnt signaling pathway | 178 | 182 | 2,914688 | 1,00733 | 0,38408 | 1 | 1 | 1 |
| Recycling pathway of L1 | 32 | 32 | 0,523989 | 1,029966 | 0,387831 | 1 | 1 | 1 |
| Formation of the HIV-1 Early Elongation Complex | 32 | 32 | 0,523989 | 1,029966 | 0,387831 | 1 | 1 | 1 |
| GAB1 signalosome | 32 | 32 | 0,523989 | 1,029966 | 0,387831 | 1 | 1 | 1 |
| Formation of the Early Elongation Complex | 32 | 32 | 0,523989 | 1,029966 | 0,387831 | 1 | 1 | 1 |
| Metabolism of steroid hormones and vitamins A and D | 32 | 32 | 0,523989 | 1,029966 | 0,387831 | 1 | 1 | 1 |
| Regulation of Lipid Metabolism by Peroxisome proliferator-activated receptor alpha (PPARalpha) | 32 | 32 | 0,523989 | 1,029966 | 0,387831 | 1 | 1 | 1 |
| Circadian Clock | 32 | 32 | 0,523989 | 1,029966 | 0,387831 | 1 | 1 | 1 |
| ErbB4 signaling events | 32 | 32 | 0,523989 | 1,029966 | 0,387831 | 1 | 1 | 1 |
| Netrin-mediated signaling events | 32 | 32 | 0,523989 | 1,029966 | 0,387831 | 1 | 1 | 1 |
| Recruitment of mitotic centrosome proteins and complexes | 69 | 70 | 1,129851 | 1,015254 | 0,390613 | 1 | 1 | 1 |
| Centrosome maturation | 69 | 70 | 1,129851 | 1,015254 | 0,390613 | 1 | 1 | 1 |
| Stabilization of p53 | 68 | 69 | 1,113476 | 1,015041 | 0,3985 | 1 | 1 | 1 |
| DNA Repair | 104 | 106 | 1,702964 | 1,010534 | 0,399182 | 1 | 1 | 1 |
| G1 Phase | 31 | 31 | 0,507614 | 1,029966 | 0,399512 | 1 | 1 | 1 |
| Cyclin D associated events in G1 | 31 | 31 | 0,507614 | 1,029966 | 0,399512 | 1 | 1 | 1 |
| DNA strand elongation | 31 | 31 | 0,507614 | 1,029966 | 0,399512 | 1 | 1 | 1 |
| G alpha (i) signalling events | 31 | 31 | 0,507614 | 1,029966 | 0,399512 | 1 | 1 | 1 |
| G alpha (s) signalling events | 31 | 31 | 0,507614 | 1,029966 | 0,399512 | 1 | 1 | 1 |
| Pyruvate metabolism and Citric Acid (TCA) cycle | 31 | 31 | 0,507614 | 1,029966 | 0,399512 | 1 | 1 | 1 |
| Amino acid transport across the plasma membrane | 31 | 31 | 0,507614 | 1,029966 | 0,399512 | 1 | 1 | 1 |
| Intrinsic Pathway for Apoptosis | 31 | 31 | 0,507614 | 1,029966 | 0,399512 | 1 | 1 | 1 |
| EPHA forward signaling | 31 | 31 | 0,507614 | 1,029966 | 0,399512 | 1 | 1 | 1 |
| Lissencephaly gene (LIS1) in neuronal migration and development | 31 | 31 | 0,507614 | 1,029966 | 0,399512 | 1 | 1 | 1 |
| Autodegradation of the E3 ubiquitin ligase COP1 | 67 | 68 | 1,097102 | 1,014821 | 0,406505 | 1 | 1 | 1 |
| Regulation of Telomerase | 67 | 68 | 1,097102 | 1,014821 | 0,406505 | 1 | 1 | 1 |
| Striated Muscle Contraction | 30 | 30 | 0,49124 | 1,029966 | 0,411543 | 1 | 1 | 1 |
| PI3K/AKT activation | 30 | 30 | 0,49124 | 1,029966 | 0,411543 | 1 | 1 | 1 |
| Signalling to ERKs | 30 | 30 | 0,49124 | 1,029966 | 0,411543 | 1 | 1 | 1 |
| Activation of the pre-replicative complex | 30 | 30 | 0,49124 | 1,029966 | 0,411543 | 1 | 1 | 1 |
| Biosynthesis of the N-glycan precursor (dolichol lipid-linked oligosaccharide, LLO) and transfer to a nascent protein | 30 | 30 | 0,49124 | 1,029966 | 0,411543 | 1 | 1 | 1 |
| Transport of vitamins, nucleosides, and related molecules | 30 | 30 | 0,49124 | 1,029966 | 0,411543 | 1 | 1 | 1 |
| MAP kinase activation in TLR cascade | 30 | 30 | 0,49124 | 1,029966 | 0,411543 | 1 | 1 | 1 |
| Ephrin B reverse signaling | 30 | 30 | 0,49124 | 1,029966 | 0,411543 | 1 | 1 | 1 |
| Cross-presentation of soluble exogenous antigens (endosomes) | 66 | 67 | 1,080727 | 1,014595 | 0,414629 | 1 | 1 | 1 |
| Downstream signaling of activated FGFR | 65 | 66 | 1,064352 | 1,014362 | 0,422871 | 1 | 1 | 1 |
| Signaling by SCF-KIT | 65 | 66 | 1,064352 | 1,014362 | 0,422871 | 1 | 1 | 1 |
| HIF-1-alpha transcription factor network | 65 | 66 | 1,064352 | 1,014362 | 0,422871 | 1 | 1 | 1 |
| Platelet Aggregation (Plug Formation) | 29 | 29 | 0,474865 | 1,029966 | 0,423934 | 1 | 1 | 1 |
| Amine compound SLC transporters | 29 | 29 | 0,474865 | 1,029966 | 0,423934 | 1 | 1 | 1 |
| G2/M DNA damage checkpoint | 29 | 29 | 0,474865 | 1,029966 | 0,423934 | 1 | 1 | 1 |
| Ion transport by P-type ATPases | 29 | 29 | 0,474865 | 1,029966 | 0,423934 | 1 | 1 | 1 |
| RNA Polymerase III Transcription Initiation | 29 | 29 | 0,474865 | 1,029966 | 0,423934 | 1 | 1 | 1 |
| Interleukin-3, 5 and GM-CSF signaling | 29 | 29 | 0,474865 | 1,029966 | 0,423934 | 1 | 1 | 1 |
| Osteopontin-mediated events | 29 | 29 | 0,474865 | 1,029966 | 0,423934 | 1 | 1 | 1 |
| Reelin signaling pathway | 29 | 29 | 0,474865 | 1,029966 | 0,423934 | 1 | 1 | 1 |
| S1P3 pathway | 29 | 29 | 0,474865 | 1,029966 | 0,423934 | 1 | 1 | 1 |
| IL12 signaling mediated by STAT4 | 29 | 29 | 0,474865 | 1,029966 | 0,423934 | 1 | 1 | 1 |
| VEGFR1 specific signals | 29 | 29 | 0,474865 | 1,029966 | 0,423934 | 1 | 1 | 1 |
| Insulin-mediated glucose transport | 29 | 29 | 0,474865 | 1,029966 | 0,423934 | 1 | 1 | 1 |
| Calcium signaling in the CD4+ TCR pathway | 29 | 29 | 0,474865 | 1,029966 | 0,423934 | 1 | 1 | 1 |
| Toll Like Receptor 9 (TLR9) Cascade | 64 | 65 | 1,047978 | 1,014122 | 0,431232 | 1 | 1 | 1 |
| Toll Like Receptor 2 (TLR2) Cascade | 64 | 65 | 1,047978 | 1,014122 | 0,431232 | 1 | 1 | 1 |
| Signaling events mediated by the Hedgehog family | 64 | 65 | 1,047978 | 1,014122 | 0,431232 | 1 | 1 | 1 |
| Platelet degranulation | 28 | 28 | 0,45849 | 1,029966 | 0,436696 | 1 | 1 | 1 |
| CREB phosphorylation through the activation of Ras | 28 | 28 | 0,45849 | 1,029966 | 0,436696 | 1 | 1 | 1 |
| Activation of GABAB receptors | 28 | 28 | 0,45849 | 1,029966 | 0,436696 | 1 | 1 | 1 |
| GABA B receptor activation | 28 | 28 | 0,45849 | 1,029966 | 0,436696 | 1 | 1 | 1 |
| Insulin receptor recycling | 28 | 28 | 0,45849 | 1,029966 | 0,436696 | 1 | 1 | 1 |
| PKB-mediated events | 28 | 28 | 0,45849 | 1,029966 | 0,436696 | 1 | 1 | 1 |
| E2F mediated regulation of DNA replication | 28 | 28 | 0,45849 | 1,029966 | 0,436696 | 1 | 1 | 1 |
| mRNA Capping | 28 | 28 | 0,45849 | 1,029966 | 0,436696 | 1 | 1 | 1 |
| Extension of Telomeres | 28 | 28 | 0,45849 | 1,029966 | 0,436696 | 1 | 1 | 1 |
| Dual incision reaction in TC-NER | 28 | 28 | 0,45849 | 1,029966 | 0,436696 | 1 | 1 | 1 |
| Formation of transcription-coupled NER (TC-NER) repair complex | 28 | 28 | 0,45849 | 1,029966 | 0,436696 | 1 | 1 | 1 |
| Transport to the Golgi and subsequent modification | 28 | 28 | 0,45849 | 1,029966 | 0,436696 | 1 | 1 | 1 |
| Protein folding | 28 | 28 | 0,45849 | 1,029966 | 0,436696 | 1 | 1 | 1 |
| Endosomal Sorting Complex Required For Transport (ESCRT) | 28 | 28 | 0,45849 | 1,029966 | 0,436696 | 1 | 1 | 1 |
| Negative regulators of RIG-I/MDA5 signaling | 28 | 28 | 0,45849 | 1,029966 | 0,436696 | 1 | 1 | 1 |
| Interleukin-2 signaling | 28 | 28 | 0,45849 | 1,029966 | 0,436696 | 1 | 1 | 1 |
| IL2 signaling events mediated by STAT5 | 28 | 28 | 0,45849 | 1,029966 | 0,436696 | 1 | 1 | 1 |
| Mitotic M-M/G1 phases | 236 | 242 | 3,864418 | 1,00443 | 0,43771 | 1 | 1 | 1 |
| Toll Like Receptor TLR1:TLR2 Cascade | 63 | 64 | 1,031603 | 1,013875 | 0,43971 | 1 | 1 | 1 |
| Toll Like Receptor TLR6:TLR2 Cascade | 63 | 64 | 1,031603 | 1,013875 | 0,43971 | 1 | 1 | 1 |
| Validated nuclear estrogen receptor alpha network | 63 | 64 | 1,031603 | 1,013875 | 0,43971 | 1 | 1 | 1 |
| TNF alpha/NF-kB | 167 | 171 | 2,734567 | 1,005874 | 0,440005 | 1 | 1 | 1 |
| Unfolded Protein Response | 62 | 63 | 1,015228 | 1,013619 | 0,448306 | 1 | 1 | 1 |
| Validated targets of C-MYC transcriptional repression | 62 | 63 | 1,015228 | 1,013619 | 0,448306 | 1 | 1 | 1 |
| The role of Nef in HIV-1 replication and disease pathogenesis | 27 | 27 | 0,442116 | 1,029966 | 0,44984 | 1 | 1 | 1 |
| Sema4D in semaphorin signaling | 27 | 27 | 0,442116 | 1,029966 | 0,44984 | 1 | 1 | 1 |
| GPVI-mediated activation cascade | 27 | 27 | 0,442116 | 1,029966 | 0,44984 | 1 | 1 | 1 |
| mTOR signalling | 27 | 27 | 0,442116 | 1,029966 | 0,44984 | 1 | 1 | 1 |
| EGFR downregulation | 27 | 27 | 0,442116 | 1,029966 | 0,44984 | 1 | 1 | 1 |
| PI-3K cascade | 27 | 27 | 0,442116 | 1,029966 | 0,44984 | 1 | 1 | 1 |
| Bile acid and bile salt metabolism | 27 | 27 | 0,442116 | 1,029966 | 0,44984 | 1 | 1 | 1 |
| Golgi Associated Vesicle Biogenesis | 27 | 27 | 0,442116 | 1,029966 | 0,44984 | 1 | 1 | 1 |
| Lipoprotein metabolism | 27 | 27 | 0,442116 | 1,029966 | 0,44984 | 1 | 1 | 1 |
| Transferrin endocytosis and recycling | 27 | 27 | 0,442116 | 1,029966 | 0,44984 | 1 | 1 | 1 |
| Aquaporin-mediated transport | 27 | 27 | 0,442116 | 1,029966 | 0,44984 | 1 | 1 | 1 |
| HIV Infection | 198 | 203 | 3,242181 | 1,004598 | 0,454061 | 1 | 1 | 1 |
| Mitotic Prophase | 61 | 62 | 0,998854 | 1,013356 | 0,457019 | 1 | 1 | 1 |
| Golgi Cisternae Pericentriolar Stack Reorganization | 61 | 62 | 0,998854 | 1,013356 | 0,457019 | 1 | 1 | 1 |
| Toll Like Receptor 7/8 (TLR7/8) Cascade | 61 | 62 | 0,998854 | 1,013356 | 0,457019 | 1 | 1 | 1 |
| MyD88:Mal cascade initiated on plasma membrane | 61 | 62 | 0,998854 | 1,013356 | 0,457019 | 1 | 1 | 1 |
| ATM pathway | 299 | 307 | 4,896021 | 1,003127 | 0,459699 | 1 | 1 | 1 |
| Neurotransmitter Release Cycle | 26 | 26 | 0,425741 | 1,029966 | 0,463377 | 1 | 1 | 1 |
| RNA Pol II CTD phosphorylation and interaction with CE | 26 | 26 | 0,425741 | 1,029966 | 0,463377 | 1 | 1 | 1 |
| RNA Pol II CTD phosphorylation and interaction with CE - 11 | 26 | 26 | 0,425741 | 1,029966 | 0,463377 | 1 | 1 | 1 |
| Post-translational modification: synthesis of GPI-anchored proteins | 26 | 26 | 0,425741 | 1,029966 | 0,463377 | 1 | 1 | 1 |
| 3-phosphoinositide biosynthesis | 26 | 26 | 0,425741 | 1,029966 | 0,463377 | 1 | 1 | 1 |
| IL27-mediated signaling events | 26 | 26 | 0,425741 | 1,029966 | 0,463377 | 1 | 1 | 1 |
| S1P2 pathway | 26 | 26 | 0,425741 | 1,029966 | 0,463377 | 1 | 1 | 1 |
| Nongenotropic Androgen signaling | 26 | 26 | 0,425741 | 1,029966 | 0,463377 | 1 | 1 | 1 |
| RXR and RAR heterodimerization with other nuclear receptor | 26 | 26 | 0,425741 | 1,029966 | 0,463377 | 1 | 1 | 1 |
| Loss of Nlp from mitotic centrosomes | 60 | 61 | 0,982479 | 1,013084 | 0,465847 | 1 | 1 | 1 |
| Loss of proteins required for interphase microtubule organizationÃ‚Â from the centrosome | 60 | 61 | 0,982479 | 1,013084 | 0,465847 | 1 | 1 | 1 |
| Fc-epsilon receptor I signaling in mast cells | 60 | 61 | 0,982479 | 1,013084 | 0,465847 | 1 | 1 | 1 |
| Toll Like Receptor 5 (TLR5) Cascade | 59 | 60 | 0,966104 | 1,012802 | 0,474791 | 1 | 1 | 1 |
| MyD88 dependent cascade initiated on endosome | 59 | 60 | 0,966104 | 1,012802 | 0,474791 | 1 | 1 | 1 |
| Toll Like Receptor 10 (TLR10) Cascade | 59 | 60 | 0,966104 | 1,012802 | 0,474791 | 1 | 1 | 1 |
| ABC-family proteins mediated transport | 25 | 25 | 0,409366 | 1,029966 | 0,47732 | 1 | 1 | 1 |
| Metal ion SLC transporters | 25 | 25 | 0,409366 | 1,029966 | 0,47732 | 1 | 1 | 1 |
| Steroid hormones | 25 | 25 | 0,409366 | 1,029966 | 0,47732 | 1 | 1 | 1 |
| TRAF6 mediated IRF7 activation | 25 | 25 | 0,409366 | 1,029966 | 0,47732 | 1 | 1 | 1 |
| superpathway of cholesterol biosynthesis | 25 | 25 | 0,409366 | 1,029966 | 0,47732 | 1 | 1 | 1 |
| TRAF6 mediated induction of NFkB and MAP kinases upon TLR7/8 or 9 activation | 58 | 59 | 0,94973 | 1,012511 | 0,483849 | 1 | 1 | 1 |
| MyD88 cascade initiated on plasma membrane | 58 | 59 | 0,94973 | 1,012511 | 0,483849 | 1 | 1 | 1 |
| ATF-2 transcription factor network | 58 | 59 | 0,94973 | 1,012511 | 0,483849 | 1 | 1 | 1 |
| CDO in myogenesis | 24 | 24 | 0,392992 | 1,029966 | 0,49168 | 1 | 1 | 1 |
| Myogenesis | 24 | 24 | 0,392992 | 1,029966 | 0,49168 | 1 | 1 | 1 |
| Smooth Muscle Contraction | 24 | 24 | 0,392992 | 1,029966 | 0,49168 | 1 | 1 | 1 |
| Purine metabolism | 24 | 24 | 0,392992 | 1,029966 | 0,49168 | 1 | 1 | 1 |
| Regulation of Water Balance by Renal Aquaporins | 24 | 24 | 0,392992 | 1,029966 | 0,49168 | 1 | 1 | 1 |
| RNA Polymerase I Transcription Initiation | 24 | 24 | 0,392992 | 1,029966 | 0,49168 | 1 | 1 | 1 |
| Visual signal transduction: Rods | 24 | 24 | 0,392992 | 1,029966 | 0,49168 | 1 | 1 | 1 |
| NOTCH | 56 | 57 | 0,916981 | 1,011899 | 0,502302 | 1 | 1 | 1 |
| Signaling by Robo receptor | 23 | 23 | 0,376617 | 1,029966 | 0,506469 | 1 | 1 | 1 |
| APC-Cdc20 mediated degradation of Nek2A | 23 | 23 | 0,376617 | 1,029966 | 0,506469 | 1 | 1 | 1 |
| Processing of Capped Intronless Pre-mRNA | 23 | 23 | 0,376617 | 1,029966 | 0,506469 | 1 | 1 | 1 |
| Post-Elongation Processing of Intronless pre-mRNA | 23 | 23 | 0,376617 | 1,029966 | 0,506469 | 1 | 1 | 1 |
| NCAM1 interactions | 23 | 23 | 0,376617 | 1,029966 | 0,506469 | 1 | 1 | 1 |
| Inwardly rectifying K+ channels | 23 | 23 | 0,376617 | 1,029966 | 0,506469 | 1 | 1 | 1 |
| Abortive elongation of HIV-1 transcript in the absence of Tat | 23 | 23 | 0,376617 | 1,029966 | 0,506469 | 1 | 1 | 1 |
| BMAL1:CLOCK/NPAS2 Activates Gene Expression | 23 | 23 | 0,376617 | 1,029966 | 0,506469 | 1 | 1 | 1 |
| Antigen Presentation: Folding, assembly and peptide loading of class I MHC | 23 | 23 | 0,376617 | 1,029966 | 0,506469 | 1 | 1 | 1 |
| heparan sulfate biosynthesis | 23 | 23 | 0,376617 | 1,029966 | 0,506469 | 1 | 1 | 1 |
| Visual signal transduction: Cones | 23 | 23 | 0,376617 | 1,029966 | 0,506469 | 1 | 1 | 1 |
| Signaling events mediated by PRL | 23 | 23 | 0,376617 | 1,029966 | 0,506469 | 1 | 1 | 1 |
| MicroRNA (miRNA) Biogenesis | 22 | 22 | 0,360242 | 1,029966 | 0,5217 | 1 | 1 | 1 |
| Regulatory RNA pathways | 22 | 22 | 0,360242 | 1,029966 | 0,5217 | 1 | 1 | 1 |
| Sema4D induced cell migration and growth-cone collapse | 22 | 22 | 0,360242 | 1,029966 | 0,5217 | 1 | 1 | 1 |
| Mitotic Metaphase/Anaphase Transition | 22 | 22 | 0,360242 | 1,029966 | 0,5217 | 1 | 1 | 1 |
| APC/C:Cdc20 mediated degradation of Cyclin B | 22 | 22 | 0,360242 | 1,029966 | 0,5217 | 1 | 1 | 1 |
| Telomere C-strand (Lagging Strand) Synthesis | 22 | 22 | 0,360242 | 1,029966 | 0,5217 | 1 | 1 | 1 |
| G alpha (z) signalling events | 22 | 22 | 0,360242 | 1,029966 | 0,5217 | 1 | 1 | 1 |
| Pyrimidine metabolism | 22 | 22 | 0,360242 | 1,029966 | 0,5217 | 1 | 1 | 1 |
| Fanconi Anemia pathway | 22 | 22 | 0,360242 | 1,029966 | 0,5217 | 1 | 1 | 1 |
| RNA Polymerase III Transcription Initiation From Type 3 Promoter | 22 | 22 | 0,360242 | 1,029966 | 0,5217 | 1 | 1 | 1 |
| Complement cascade | 22 | 22 | 0,360242 | 1,029966 | 0,5217 | 1 | 1 | 1 |
| Cellular roles of Anthrax toxin | 22 | 22 | 0,360242 | 1,029966 | 0,5217 | 1 | 1 | 1 |
| Metabolism of lipids and lipoproteins | 250 | 257 | 4,093663 | 1,001913 | 0,525146 | 1 | 1 | 1 |
| Canonical Wnt signaling pathway | 151 | 155 | 2,472572 | 1,003388 | 0,527718 | 1 | 1 | 1 |
| Kinesins | 21 | 21 | 0,343868 | 1,029966 | 0,537387 | 1 | 1 | 1 |
| Glutamate Binding, Activation of AMPA Receptors and Synaptic Plasticity | 21 | 21 | 0,343868 | 1,029966 | 0,537387 | 1 | 1 | 1 |
| Trafficking of AMPA receptors | 21 | 21 | 0,343868 | 1,029966 | 0,537387 | 1 | 1 | 1 |
| Signalling to RAS | 21 | 21 | 0,343868 | 1,029966 | 0,537387 | 1 | 1 | 1 |
| PIP3 activates AKT signaling | 21 | 21 | 0,343868 | 1,029966 | 0,537387 | 1 | 1 | 1 |
| G0 and Early G1 | 21 | 21 | 0,343868 | 1,029966 | 0,537387 | 1 | 1 | 1 |
| Signaling by Notch | 21 | 21 | 0,343868 | 1,029966 | 0,537387 | 1 | 1 | 1 |
| G alpha (12/13) signalling events | 21 | 21 | 0,343868 | 1,029966 | 0,537387 | 1 | 1 | 1 |
| Incretin Synthesis, Secretion, and Inactivation | 21 | 21 | 0,343868 | 1,029966 | 0,537387 | 1 | 1 | 1 |
| RNA Polymerase III Transcription Initiation From Type 1 Promoter | 21 | 21 | 0,343868 | 1,029966 | 0,537387 | 1 | 1 | 1 |
| MAPK targets/ Nuclear events mediated by MAP kinases | 21 | 21 | 0,343868 | 1,029966 | 0,537387 | 1 | 1 | 1 |
| p38 signaling mediated by MAPKAP kinases | 21 | 21 | 0,343868 | 1,029966 | 0,537387 | 1 | 1 | 1 |
| PDGFR-alpha signaling pathway | 21 | 21 | 0,343868 | 1,029966 | 0,537387 | 1 | 1 | 1 |
| E-cadherin signaling in keratinocytes | 21 | 21 | 0,343868 | 1,029966 | 0,537387 | 1 | 1 | 1 |
| Opioid Signalling | 52 | 53 | 0,851482 | 1,010536 | 0,540512 | 1 | 1 | 1 |
| Formation and Maturation of mRNA Transcript | 180 | 185 | 2,947437 | 1,00213 | 0,543383 | 1 | 1 | 1 |
| Signaling events mediated by Stem cell factor receptor (c-Kit) | 51 | 52 | 0,835107 | 1,010162 | 0,550323 | 1 | 1 | 1 |
| Nef-mediates down modulation of cell surface receptors by recruiting them to clathrin adapters | 20 | 20 | 0,327493 | 1,029966 | 0,553543 | 1 | 1 | 1 |
| Effects of PIP2 hydrolysis | 20 | 20 | 0,327493 | 1,029966 | 0,553543 | 1 | 1 | 1 |
| Integrin alphaIIb beta3 signaling | 20 | 20 | 0,327493 | 1,029966 | 0,553543 | 1 | 1 | 1 |
| Prolonged ERK activation events | 20 | 20 | 0,327493 | 1,029966 | 0,553543 | 1 | 1 | 1 |
| PKA-mediated phosphorylation of CREB | 20 | 20 | 0,327493 | 1,029966 | 0,553543 | 1 | 1 | 1 |
| Phosphorylation of Emi1 | 20 | 20 | 0,327493 | 1,029966 | 0,553543 | 1 | 1 | 1 |
| Lagging Strand Synthesis | 20 | 20 | 0,327493 | 1,029966 | 0,553543 | 1 | 1 | 1 |
| Cholesterol biosynthesis | 20 | 20 | 0,327493 | 1,029966 | 0,553543 | 1 | 1 | 1 |
| Peroxisomal lipid metabolism | 20 | 20 | 0,327493 | 1,029966 | 0,553543 | 1 | 1 | 1 |
| Formation of incision complex in GG-NER | 20 | 20 | 0,327493 | 1,029966 | 0,553543 | 1 | 1 | 1 |
| Dual incision reaction in GG-NER | 20 | 20 | 0,327493 | 1,029966 | 0,553543 | 1 | 1 | 1 |
| Destabilization of mRNA by Butyrate Response Factor 1 (BRF1) | 20 | 20 | 0,327493 | 1,029966 | 0,553543 | 1 | 1 | 1 |
| Gluconeogenesis | 20 | 20 | 0,327493 | 1,029966 | 0,553543 | 1 | 1 | 1 |
| RNA Polymerase I Promoter Escape | 20 | 20 | 0,327493 | 1,029966 | 0,553543 | 1 | 1 | 1 |
| RNA Polymerase III Transcription Initiation From Type 2 Promoter | 20 | 20 | 0,327493 | 1,029966 | 0,553543 | 1 | 1 | 1 |
| chondroitin sulfate biosynthesis | 20 | 20 | 0,327493 | 1,029966 | 0,553543 | 1 | 1 | 1 |
| Arf1 pathway | 20 | 20 | 0,327493 | 1,029966 | 0,553543 | 1 | 1 | 1 |
| ATR signaling pathway | 243 | 250 | 3,97904 | 1,001128 | 0,556179 | 1 | 1 | 1 |
| Fatty acid, triacylglycerol, and ketone body metabolism | 81 | 83 | 1,326347 | 1,00515 | 0,563583 | 1 | 1 | 1 |
| Frs2-mediated activation | 19 | 19 | 0,311118 | 1,029966 | 0,570182 | 1 | 1 | 1 |
| PKA activation | 19 | 19 | 0,311118 | 1,029966 | 0,570182 | 1 | 1 | 1 |
| Signaling by BMP | 19 | 19 | 0,311118 | 1,029966 | 0,570182 | 1 | 1 | 1 |
| Base Excision Repair | 19 | 19 | 0,311118 | 1,029966 | 0,570182 | 1 | 1 | 1 |
| Synthesis of bile acids and bile salts | 19 | 19 | 0,311118 | 1,029966 | 0,570182 | 1 | 1 | 1 |
| Resolution of Abasic Sites (AP sites) | 19 | 19 | 0,311118 | 1,029966 | 0,570182 | 1 | 1 | 1 |
| Mitotic Spindle Checkpoint | 19 | 19 | 0,311118 | 1,029966 | 0,570182 | 1 | 1 | 1 |
| Destabilization of mRNA by KSRP | 19 | 19 | 0,311118 | 1,029966 | 0,570182 | 1 | 1 | 1 |
| Citric acid cycle (TCA cycle) | 19 | 19 | 0,311118 | 1,029966 | 0,570182 | 1 | 1 | 1 |
| fatty acid beta-oxidation I | 19 | 19 | 0,311118 | 1,029966 | 0,570182 | 1 | 1 | 1 |
| EPHA2 forward signaling | 19 | 19 | 0,311118 | 1,029966 | 0,570182 | 1 | 1 | 1 |
| FOXA transcription factor networks | 79 | 81 | 1,293598 | 1,004537 | 0,579144 | 1 | 1 | 1 |
| FoxO family signaling | 48 | 49 | 0,785983 | 1,00895 | 0,580341 | 1 | 1 | 1 |
| Tie2 Signaling | 18 | 18 | 0,294744 | 1,029966 | 0,587319 | 1 | 1 | 1 |
| Intrinsic Pathway | 18 | 18 | 0,294744 | 1,029966 | 0,587319 | 1 | 1 | 1 |
| Mitotic Telophase /Cytokinesis | 18 | 18 | 0,294744 | 1,029966 | 0,587319 | 1 | 1 | 1 |
| Inactivation of APC/C via direct inhibition of the APC/C complex | 18 | 18 | 0,294744 | 1,029966 | 0,587319 | 1 | 1 | 1 |
| Inhibition of the proteolytic activity of APC/C required for the onset of anaphase by mitotic spindle checkpoint components | 18 | 18 | 0,294744 | 1,029966 | 0,587319 | 1 | 1 | 1 |
| ARMS-mediated activation | 18 | 18 | 0,294744 | 1,029966 | 0,587319 | 1 | 1 | 1 |
| Phosphorylation of the APC/C | 18 | 18 | 0,294744 | 1,029966 | 0,587319 | 1 | 1 | 1 |
| Polo-like kinase mediated events | 18 | 18 | 0,294744 | 1,029966 | 0,587319 | 1 | 1 | 1 |
| DARPP-32 events | 18 | 18 | 0,294744 | 1,029966 | 0,587319 | 1 | 1 | 1 |
| eNOS activation and regulation | 18 | 18 | 0,294744 | 1,029966 | 0,587319 | 1 | 1 | 1 |
| Metabolism of nitric oxide | 18 | 18 | 0,294744 | 1,029966 | 0,587319 | 1 | 1 | 1 |
| Activated AMPK stimulates fatty-acid oxidation in muscle | 18 | 18 | 0,294744 | 1,029966 | 0,587319 | 1 | 1 | 1 |
| Synthesis, Secretion, and Inactivation of Glucagon-like Peptide-1 (GLP-1) | 18 | 18 | 0,294744 | 1,029966 | 0,587319 | 1 | 1 | 1 |
| Fatty Acyl-CoA Biosynthesis | 18 | 18 | 0,294744 | 1,029966 | 0,587319 | 1 | 1 | 1 |
| Na+/Cl- dependent neurotransmitter transporters | 18 | 18 | 0,294744 | 1,029966 | 0,587319 | 1 | 1 | 1 |
| CD28 dependent Vav1 pathway | 18 | 18 | 0,294744 | 1,029966 | 0,587319 | 1 | 1 | 1 |
| Growth hormone receptor signaling | 18 | 18 | 0,294744 | 1,029966 | 0,587319 | 1 | 1 | 1 |
| Degradation of beta catenin | 18 | 18 | 0,294744 | 1,029966 | 0,587319 | 1 | 1 | 1 |
| Apoptotic execution phase | 47 | 48 | 0,769609 | 1,008512 | 0,59053 | 1 | 1 | 1 |
| Interferon gamma signaling | 46 | 47 | 0,753234 | 1,008056 | 0,600804 | 1 | 1 | 1 |
| Thrombin signalling through proteinase activated receptors (PARs) | 17 | 17 | 0,278369 | 1,029966 | 0,604967 | 1 | 1 | 1 |
| GABA synthesis, release, reuptake and degradation | 17 | 17 | 0,278369 | 1,029966 | 0,604967 | 1 | 1 | 1 |
| Ras activation uopn Ca2+ infux through NMDA receptor | 17 | 17 | 0,278369 | 1,029966 | 0,604967 | 1 | 1 | 1 |
| Energy dependent regulation of mTOR by LKB1-AMPK | 17 | 17 | 0,278369 | 1,029966 | 0,604967 | 1 | 1 | 1 |
| Negative regulation of FGFR signaling | 17 | 17 | 0,278369 | 1,029966 | 0,604967 | 1 | 1 | 1 |
| Removal of DNA patch containing abasic residue | 17 | 17 | 0,278369 | 1,029966 | 0,604967 | 1 | 1 | 1 |
| Resolution of AP sites via the multiple-nucleotide patch replacement pathway | 17 | 17 | 0,278369 | 1,029966 | 0,604967 | 1 | 1 | 1 |
| Synthesis of glycosylphosphatidylinositol (GPI) | 17 | 17 | 0,278369 | 1,029966 | 0,604967 | 1 | 1 | 1 |
| PKA activation in glucagon signalling | 17 | 17 | 0,278369 | 1,029966 | 0,604967 | 1 | 1 | 1 |
| Destabilization of mRNA by Tristetraprolin (TTP) | 17 | 17 | 0,278369 | 1,029966 | 0,604967 | 1 | 1 | 1 |
| Branched-chain amino acid catabolism | 17 | 17 | 0,278369 | 1,029966 | 0,604967 | 1 | 1 | 1 |
| Zinc transporters | 17 | 17 | 0,278369 | 1,029966 | 0,604967 | 1 | 1 | 1 |
| Ligand-gated ion channel transport | 17 | 17 | 0,278369 | 1,029966 | 0,604967 | 1 | 1 | 1 |
| CD28 dependent PI3K/Akt signaling | 17 | 17 | 0,278369 | 1,029966 | 0,604967 | 1 | 1 | 1 |
| RNA Polymerase III Transcription Termination | 17 | 17 | 0,278369 | 1,029966 | 0,604967 | 1 | 1 | 1 |
| Activation of BH3-only proteins | 17 | 17 | 0,278369 | 1,029966 | 0,604967 | 1 | 1 | 1 |
| Inflammasomes | 17 | 17 | 0,278369 | 1,029966 | 0,604967 | 1 | 1 | 1 |
| dermatan sulfate biosynthesis | 17 | 17 | 0,278369 | 1,029966 | 0,604967 | 1 | 1 | 1 |
| Syndecan-3-mediated signaling events | 17 | 17 | 0,278369 | 1,029966 | 0,604967 | 1 | 1 | 1 |
| Host Interactions of HIV factors | 137 | 141 | 2,243327 | 1,000749 | 0,608434 | 1 | 1 | 1 |
| Deadenylation-dependent mRNA decay | 45 | 46 | 0,736859 | 1,00758 | 0,611161 | 1 | 1 | 1 |
| Lipid digestion, mobilization, and transport | 45 | 46 | 0,736859 | 1,00758 | 0,611161 | 1 | 1 | 1 |
| Presenilin action in Notch and Wnt signaling | 45 | 46 | 0,736859 | 1,00758 | 0,611161 | 1 | 1 | 1 |
| Netrin-1 signaling | 44 | 45 | 0,720485 | 1,007083 | 0,621594 | 1 | 1 | 1 |
| Activation of Chaperones by IRE1alpha | 44 | 45 | 0,720485 | 1,007083 | 0,621594 | 1 | 1 | 1 |
| Other semaphorin interactions | 16 | 16 | 0,261994 | 1,029966 | 0,623143 | 1 | 1 | 1 |
| CRMPs in Sema3A signaling | 16 | 16 | 0,261994 | 1,029966 | 0,623143 | 1 | 1 | 1 |
| Signal amplification | 16 | 16 | 0,261994 | 1,029966 | 0,623143 | 1 | 1 | 1 |
| Signaling by NODAL | 16 | 16 | 0,261994 | 1,029966 | 0,623143 | 1 | 1 | 1 |
| Common Pathway | 16 | 16 | 0,261994 | 1,029966 | 0,623143 | 1 | 1 | 1 |
| Regulation of AMPK activity via LKB1 | 16 | 16 | 0,261994 | 1,029966 | 0,623143 | 1 | 1 | 1 |
| Signalling to p38 via RIT and RIN | 16 | 16 | 0,261994 | 1,029966 | 0,623143 | 1 | 1 | 1 |
| p75NTR signals via NF-kB | 16 | 16 | 0,261994 | 1,029966 | 0,623143 | 1 | 1 | 1 |
| SHC1 events in EGFR signaling | 16 | 16 | 0,261994 | 1,029966 | 0,623143 | 1 | 1 | 1 |
| SHC-related events | 16 | 16 | 0,261994 | 1,029966 | 0,623143 | 1 | 1 | 1 |
| Spry regulation of FGF signaling | 16 | 16 | 0,261994 | 1,029966 | 0,623143 | 1 | 1 | 1 |
| Conversion from APC/C:Cdc20 to APC/C:Cdh1 in late anaphase | 16 | 16 | 0,261994 | 1,029966 | 0,623143 | 1 | 1 | 1 |
| Synthesis, Secretion, and Deacylation of Ghrelin | 16 | 16 | 0,261994 | 1,029966 | 0,623143 | 1 | 1 | 1 |
| Glucagon-type ligand receptors | 16 | 16 | 0,261994 | 1,029966 | 0,623143 | 1 | 1 | 1 |
| Synthesis and interconversion of nucleotide di- and triphosphates | 16 | 16 | 0,261994 | 1,029966 | 0,623143 | 1 | 1 | 1 |
| Gap-filling DNA repair synthesis and ligation in TC-NER | 16 | 16 | 0,261994 | 1,029966 | 0,623143 | 1 | 1 | 1 |
| Gap-filling DNA repair synthesis and ligation in GG-NER | 16 | 16 | 0,261994 | 1,029966 | 0,623143 | 1 | 1 | 1 |
| Synthesis of substrates in N-glycan biosythesis | 16 | 16 | 0,261994 | 1,029966 | 0,623143 | 1 | 1 | 1 |
| Chaperonin-mediated protein folding | 16 | 16 | 0,261994 | 1,029966 | 0,623143 | 1 | 1 | 1 |
| Regulation of Insulin Secretion by Glucagon-like Peptide-1 | 16 | 16 | 0,261994 | 1,029966 | 0,623143 | 1 | 1 | 1 |
| Chylomicron-mediated lipid transport | 16 | 16 | 0,261994 | 1,029966 | 0,623143 | 1 | 1 | 1 |
| Cell-extracellular matrix interactions | 16 | 16 | 0,261994 | 1,029966 | 0,623143 | 1 | 1 | 1 |
| heparan sulfate biosynthesis (late stages) | 16 | 16 | 0,261994 | 1,029966 | 0,623143 | 1 | 1 | 1 |
| LPA4-mediated signaling events | 16 | 16 | 0,261994 | 1,029966 | 0,623143 | 1 | 1 | 1 |
| Hedgehog | 16 | 16 | 0,261994 | 1,029966 | 0,623143 | 1 | 1 | 1 |
| FOXA1 transcription factor network | 43 | 44 | 0,70411 | 1,006563 | 0,6321 | 1 | 1 | 1 |
| p63 transcription factor network | 102 | 105 | 1,670215 | 1,000541 | 0,634734 | 1 | 1 | 1 |
| Sema3A PAK dependent Axon repulsion | 15 | 15 | 0,24562 | 1,029966 | 0,641862 | 1 | 1 | 1 |
| Netrin mediated repulsion signals | 15 | 15 | 0,24562 | 1,029966 | 0,641862 | 1 | 1 | 1 |
| Activation of G protein gated Potassium channels | 15 | 15 | 0,24562 | 1,029966 | 0,641862 | 1 | 1 | 1 |
| G protein gated Potassium channels | 15 | 15 | 0,24562 | 1,029966 | 0,641862 | 1 | 1 | 1 |
| Inhibition of voltage gated Ca2+ channels via Gbeta/gamma subunits | 15 | 15 | 0,24562 | 1,029966 | 0,641862 | 1 | 1 | 1 |
| CREB phosphorylation through the activation of CaMKII | 15 | 15 | 0,24562 | 1,029966 | 0,641862 | 1 | 1 | 1 |
| Nuclear Events (kinase and transcription factor activation) | 15 | 15 | 0,24562 | 1,029966 | 0,641862 | 1 | 1 | 1 |
| SOS-mediated signalling | 15 | 15 | 0,24562 | 1,029966 | 0,641862 | 1 | 1 | 1 |
| GRB2 events in EGFR signaling | 15 | 15 | 0,24562 | 1,029966 | 0,641862 | 1 | 1 | 1 |
| FRS2-mediated cascade | 15 | 15 | 0,24562 | 1,029966 | 0,641862 | 1 | 1 | 1 |
| G1/S-Specific Transcription | 15 | 15 | 0,24562 | 1,029966 | 0,641862 | 1 | 1 | 1 |
| Processive synthesis on the lagging strand | 15 | 15 | 0,24562 | 1,029966 | 0,641862 | 1 | 1 | 1 |
| Association of licensing factors with the pre-replicative complex | 15 | 15 | 0,24562 | 1,029966 | 0,641862 | 1 | 1 | 1 |
| Class C/3 (Metabotropic glutamate/pheromone receptors) | 15 | 15 | 0,24562 | 1,029966 | 0,641862 | 1 | 1 | 1 |
| Eicosanoid ligand-binding receptors | 15 | 15 | 0,24562 | 1,029966 | 0,641862 | 1 | 1 | 1 |
| Nucleotide-like (purinergic) receptors | 15 | 15 | 0,24562 | 1,029966 | 0,641862 | 1 | 1 | 1 |
| Synthesis of bile acids and bile salts via 7alpha-hydroxycholesterol | 15 | 15 | 0,24562 | 1,029966 | 0,641862 | 1 | 1 | 1 |
| Repair synthesis of patch ~27-30 bases long by DNA polymerase | 15 | 15 | 0,24562 | 1,029966 | 0,641862 | 1 | 1 | 1 |
| Repair synthesis for gap-filling by DNA polymerase in TC-NER | 15 | 15 | 0,24562 | 1,029966 | 0,641862 | 1 | 1 | 1 |
| Cooperation of Prefoldin and TriC/CCT in actin and tubulin folding | 15 | 15 | 0,24562 | 1,029966 | 0,641862 | 1 | 1 | 1 |
| Lysosome Vesicle Biogenesis | 15 | 15 | 0,24562 | 1,029966 | 0,641862 | 1 | 1 | 1 |
| Glutathione conjugation | 15 | 15 | 0,24562 | 1,029966 | 0,641862 | 1 | 1 | 1 |
| Metabolism of polyamines | 15 | 15 | 0,24562 | 1,029966 | 0,641862 | 1 | 1 | 1 |
| Endogenous sterols | 15 | 15 | 0,24562 | 1,029966 | 0,641862 | 1 | 1 | 1 |
| HDL-mediated lipid transport | 15 | 15 | 0,24562 | 1,029966 | 0,641862 | 1 | 1 | 1 |
| Initial triggering of complement | 15 | 15 | 0,24562 | 1,029966 | 0,641862 | 1 | 1 | 1 |
| salvage pathways of pyrimidine ribonucleotides | 15 | 15 | 0,24562 | 1,029966 | 0,641862 | 1 | 1 | 1 |
| D-myo-inositol (1,3,4)-trisphosphate biosynthesis | 15 | 15 | 0,24562 | 1,029966 | 0,641862 | 1 | 1 | 1 |
| 1D-myo-inositol hexakisphosphate biosynthesis II (mammalian) | 15 | 15 | 0,24562 | 1,029966 | 0,641862 | 1 | 1 | 1 |
| Sumoylation by RanBP2 regulates transcriptional repression | 15 | 15 | 0,24562 | 1,029966 | 0,641862 | 1 | 1 | 1 |
| Validated nuclear estrogen receptor beta network | 15 | 15 | 0,24562 | 1,029966 | 0,641862 | 1 | 1 | 1 |
| PAR4-mediated thrombin signaling events | 15 | 15 | 0,24562 | 1,029966 | 0,641862 | 1 | 1 | 1 |
| Nucleotide-binding domain, leucine rich repeat containing receptor (NLR) signaling pathways | 42 | 43 | 0,687735 | 1,006018 | 0,642674 | 1 | 1 | 1 |
| FOXA2 and FOXA3 transcription factor networks | 42 | 43 | 0,687735 | 1,006018 | 0,642674 | 1 | 1 | 1 |
| Beta3 integrin cell surface interactions | 42 | 43 | 0,687735 | 1,006018 | 0,642674 | 1 | 1 | 1 |
| Regulation of nuclear beta catenin signaling and target gene transcription | 131 | 135 | 2,145079 | 0,99945 | 0,643417 | 1 | 1 | 1 |
| Chemokine receptors bind chemokines | 41 | 42 | 0,671361 | 1,005448 | 0,65331 | 1 | 1 | 1 |
| FOXM1 transcription factor network | 41 | 42 | 0,671361 | 1,005448 | 0,65331 | 1 | 1 | 1 |
| SEMA3A-Plexin repulsion signaling by inhibiting Integrin adhesion | 14 | 14 | 0,229245 | 1,029966 | 0,66114 | 1 | 1 | 1 |
| Processing of Intronless Pre-mRNAs | 14 | 14 | 0,229245 | 1,029966 | 0,66114 | 1 | 1 | 1 |
| NRIF signals cell death from the nucleus | 14 | 14 | 0,229245 | 1,029966 | 0,66114 | 1 | 1 | 1 |
| FGFR ligand binding and activation | 14 | 14 | 0,229245 | 1,029966 | 0,66114 | 1 | 1 | 1 |
| Cyclin A/B1 associated events during G2/M transition | 14 | 14 | 0,229245 | 1,029966 | 0,66114 | 1 | 1 | 1 |
| Regulation of Insulin-like Growth Factor (IGF) Activity by Insulin-like Growth Factor Binding Proteins (IGFBPs) | 14 | 14 | 0,229245 | 1,029966 | 0,66114 | 1 | 1 | 1 |
| Polymerase switching on the C-strand of the telomere | 14 | 14 | 0,229245 | 1,029966 | 0,66114 | 1 | 1 | 1 |
| Removal of the Flap Intermediate | 14 | 14 | 0,229245 | 1,029966 | 0,66114 | 1 | 1 | 1 |
| Polymerase switching | 14 | 14 | 0,229245 | 1,029966 | 0,66114 | 1 | 1 | 1 |
| Leading Strand Synthesis | 14 | 14 | 0,229245 | 1,029966 | 0,66114 | 1 | 1 | 1 |
| Prefoldin mediated transfer of substrate to CCT/TriC | 14 | 14 | 0,229245 | 1,029966 | 0,66114 | 1 | 1 | 1 |
| Post-translational modification: gamma carboxylation and hypusine formation | 14 | 14 | 0,229245 | 1,029966 | 0,66114 | 1 | 1 | 1 |
| Viral Messenger RNA Synthesis | 14 | 14 | 0,229245 | 1,029966 | 0,66114 | 1 | 1 | 1 |
| Amine-derived hormones | 14 | 14 | 0,229245 | 1,029966 | 0,66114 | 1 | 1 | 1 |
| Xenobiotics | 14 | 14 | 0,229245 | 1,029966 | 0,66114 | 1 | 1 | 1 |
| Synthesis of very long-chain fatty acyl-CoAs | 14 | 14 | 0,229245 | 1,029966 | 0,66114 | 1 | 1 | 1 |
| Glycogen breakdown (glycogenolysis) | 14 | 14 | 0,229245 | 1,029966 | 0,66114 | 1 | 1 | 1 |
| TRAF6 mediated induction of TAK1 complex | 14 | 14 | 0,229245 | 1,029966 | 0,66114 | 1 | 1 | 1 |
| guanosine nucleotides de novo biosynthesis | 14 | 14 | 0,229245 | 1,029966 | 0,66114 | 1 | 1 | 1 |
| pyrimidine deoxyribonucleotides de novo biosynthesis | 14 | 14 | 0,229245 | 1,029966 | 0,66114 | 1 | 1 | 1 |
| S1P4 pathway | 14 | 14 | 0,229245 | 1,029966 | 0,66114 | 1 | 1 | 1 |
| Atypical NF-kappaB pathway | 14 | 14 | 0,229245 | 1,029966 | 0,66114 | 1 | 1 | 1 |
| ID | 14 | 14 | 0,229245 | 1,029966 | 0,66114 | 1 | 1 | 1 |
| RNA Polymerase II Transcription | 98 | 101 | 1,604716 | 0,999376 | 0,661407 | 1 | 1 | 1 |
| Vif-mediated degradation of APOBEC3G | 67 | 69 | 1,097102 | 1,000116 | 0,674679 | 1 | 1 | 1 |
| G-protein mediated events | 39 | 40 | 0,638611 | 1,004223 | 0,674746 | 1 | 1 | 1 |
| amb2 Integrin signaling | 39 | 40 | 0,638611 | 1,004223 | 0,674746 | 1 | 1 | 1 |
| Platelet Adhesion to exposed collagen | 13 | 13 | 0,21287 | 1,029966 | 0,680994 | 1 | 1 | 1 |
| Inhibition of adenylate cyclase pathway | 13 | 13 | 0,21287 | 1,029966 | 0,680994 | 1 | 1 | 1 |
| Adenylate cyclase inhibitory pathway | 13 | 13 | 0,21287 | 1,029966 | 0,680994 | 1 | 1 | 1 |
| NF-kB is activated and signals survival | 13 | 13 | 0,21287 | 1,029966 | 0,680994 | 1 | 1 | 1 |
| p75NTR recruits signalling complexes | 13 | 13 | 0,21287 | 1,029966 | 0,680994 | 1 | 1 | 1 |
| SHC-mediated signalling | 13 | 13 | 0,21287 | 1,029966 | 0,680994 | 1 | 1 | 1 |
| Signaling by TGF beta | 13 | 13 | 0,21287 | 1,029966 | 0,680994 | 1 | 1 | 1 |
| G-protein activation | 13 | 13 | 0,21287 | 1,029966 | 0,680994 | 1 | 1 | 1 |
| Prostanoid metabolism | 13 | 13 | 0,21287 | 1,029966 | 0,680994 | 1 | 1 | 1 |
| N-glycan trimming in the ER and Calnexin/Calreticulin cycle | 13 | 13 | 0,21287 | 1,029966 | 0,680994 | 1 | 1 | 1 |
| Synthesis, Secretion, and Inactivation of Glucose-dependent Insulinotropic Polypeptide (GIP) | 13 | 13 | 0,21287 | 1,029966 | 0,680994 | 1 | 1 | 1 |
| mRNA Decay by 5' to 3' Exoribonuclease | 13 | 13 | 0,21287 | 1,029966 | 0,680994 | 1 | 1 | 1 |
| Death Receptor Signalling | 13 | 13 | 0,21287 | 1,029966 | 0,680994 | 1 | 1 | 1 |
| Extrinsic Pathway for Apoptosis | 13 | 13 | 0,21287 | 1,029966 | 0,680994 | 1 | 1 | 1 |
| Viral dsRNA:TLR3:TRIF Complex Activates RIP1 | 13 | 13 | 0,21287 | 1,029966 | 0,680994 | 1 | 1 | 1 |
| TRAF6 mediated NF-kB activation | 13 | 13 | 0,21287 | 1,029966 | 0,680994 | 1 | 1 | 1 |
| Regulation of IFNG signaling | 13 | 13 | 0,21287 | 1,029966 | 0,680994 | 1 | 1 | 1 |
| superpathway of geranylgeranyldiphosphate biosynthesis I (via mevalonate) | 13 | 13 | 0,21287 | 1,029966 | 0,680994 | 1 | 1 | 1 |
| cholesterol biosynthesis II (via 24,25-dihydrolanosterol) | 13 | 13 | 0,21287 | 1,029966 | 0,680994 | 1 | 1 | 1 |
| cholesterol biosynthesis I | 13 | 13 | 0,21287 | 1,029966 | 0,680994 | 1 | 1 | 1 |
| cholesterol biosynthesis III (via desmosterol) | 13 | 13 | 0,21287 | 1,029966 | 0,680994 | 1 | 1 | 1 |
| TCA cycle variation III (eukaryotic) | 13 | 13 | 0,21287 | 1,029966 | 0,680994 | 1 | 1 | 1 |
| DNA-PK pathway in nonhomologous end joining | 13 | 13 | 0,21287 | 1,029966 | 0,680994 | 1 | 1 | 1 |
| p75 NTR receptor-mediated signalling | 38 | 39 | 0,622237 | 1,003563 | 0,685533 | 1 | 1 | 1 |
| PLC beta mediated events | 38 | 39 | 0,622237 | 1,003563 | 0,685533 | 1 | 1 | 1 |
| Interleukin-1 signaling | 38 | 39 | 0,622237 | 1,003563 | 0,685533 | 1 | 1 | 1 |
| Signaling events mediated by HDAC Class II | 37 | 38 | 0,605862 | 1,002868 | 0,696357 | 1 | 1 | 1 |
| IL8-mediated signaling events | 37 | 38 | 0,605862 | 1,002868 | 0,696357 | 1 | 1 | 1 |
| Interaction between L1 and Ankyrins | 12 | 12 | 0,196496 | 1,029966 | 0,70144 | 1 | 1 | 1 |
| ERK/MAPK targets | 12 | 12 | 0,196496 | 1,029966 | 0,70144 | 1 | 1 | 1 |
| Recruitment of NuMA to mitotic centrosomes | 12 | 12 | 0,196496 | 1,029966 | 0,70144 | 1 | 1 | 1 |
| Serotonin receptors | 12 | 12 | 0,196496 | 1,029966 | 0,70144 | 1 | 1 | 1 |
| Resolution of AP sites via the single-nucleotide replacement pathway | 12 | 12 | 0,196496 | 1,029966 | 0,70144 | 1 | 1 | 1 |
| Tetrahydrobiopterin (BH4) synthesis, recycling, salvage and regulation | 12 | 12 | 0,196496 | 1,029966 | 0,70144 | 1 | 1 | 1 |
| N-glycan antennae elongation in the medial/trans-Golgi | 12 | 12 | 0,196496 | 1,029966 | 0,70144 | 1 | 1 | 1 |
| Calnexin/calreticulin cycle | 12 | 12 | 0,196496 | 1,029966 | 0,70144 | 1 | 1 | 1 |
| Post-chaperonin tubulin folding pathway | 12 | 12 | 0,196496 | 1,029966 | 0,70144 | 1 | 1 | 1 |
| Gamma-carboxylation, transport, and amino-terminal cleavage of proteins | 12 | 12 | 0,196496 | 1,029966 | 0,70144 | 1 | 1 | 1 |
| Bile salt and organic anion SLC transporters | 12 | 12 | 0,196496 | 1,029966 | 0,70144 | 1 | 1 | 1 |
| Hormone-sensitive lipase (HSL)-mediated triacylglycerol hydrolysis | 12 | 12 | 0,196496 | 1,029966 | 0,70144 | 1 | 1 | 1 |
| Facilitative Na+-independent glucose transporters | 12 | 12 | 0,196496 | 1,029966 | 0,70144 | 1 | 1 | 1 |
| Organic cation/anion/zwitterion transport | 12 | 12 | 0,196496 | 1,029966 | 0,70144 | 1 | 1 | 1 |
| PD-1 signaling | 12 | 12 | 0,196496 | 1,029966 | 0,70144 | 1 | 1 | 1 |
| RNA Polymerase III Chain Elongation | 12 | 12 | 0,196496 | 1,029966 | 0,70144 | 1 | 1 | 1 |
| Apoptotic cleavage of cell adhesion proteins | 12 | 12 | 0,196496 | 1,029966 | 0,70144 | 1 | 1 | 1 |
| chondroitin sulfate biosynthesis (late stages) | 12 | 12 | 0,196496 | 1,029966 | 0,70144 | 1 | 1 | 1 |
| stearate biosynthesis I (animals) | 12 | 12 | 0,196496 | 1,029966 | 0,70144 | 1 | 1 | 1 |
| valine degradation I | 12 | 12 | 0,196496 | 1,029966 | 0,70144 | 1 | 1 | 1 |
| purine nucleotides degradation II (aerobic) | 12 | 12 | 0,196496 | 1,029966 | 0,70144 | 1 | 1 | 1 |
| Metabolism of carbohydrates | 89 | 92 | 1,457344 | 0,996383 | 0,720897 | 1 | 1 | 1 |
| SLBP Dependent Processing of Replication-Dependent Histone Pre-mRNAs | 11 | 11 | 0,180121 | 1,029966 | 0,722497 | 1 | 1 | 1 |
| RAF/MAP kinase cascade | 11 | 11 | 0,180121 | 1,029966 | 0,722497 | 1 | 1 | 1 |
| Unblocking of NMDA receptor, glutamate binding and activation | 11 | 11 | 0,180121 | 1,029966 | 0,722497 | 1 | 1 | 1 |
| Acetylcholine Neurotransmitter Release Cycle | 11 | 11 | 0,180121 | 1,029966 | 0,722497 | 1 | 1 | 1 |
| Norepinephrine Neurotransmitter Release Cycle | 11 | 11 | 0,180121 | 1,029966 | 0,722497 | 1 | 1 | 1 |
| Glutamate Neurotransmitter Release Cycle | 11 | 11 | 0,180121 | 1,029966 | 0,722497 | 1 | 1 | 1 |
| Early Phase of HIV Life Cycle | 11 | 11 | 0,180121 | 1,029966 | 0,722497 | 1 | 1 | 1 |
| Retrograde neurotrophin signalling | 11 | 11 | 0,180121 | 1,029966 | 0,722497 | 1 | 1 | 1 |
| Signal attenuation | 11 | 11 | 0,180121 | 1,029966 | 0,722497 | 1 | 1 | 1 |
| S6K1-mediated signalling | 11 | 11 | 0,180121 | 1,029966 | 0,722497 | 1 | 1 | 1 |
| mTORC1-mediated signalling | 11 | 11 | 0,180121 | 1,029966 | 0,722497 | 1 | 1 | 1 |
| CDC6 association with the ORC:origin complex | 11 | 11 | 0,180121 | 1,029966 | 0,722497 | 1 | 1 | 1 |
| PERK regulated gene expression | 11 | 11 | 0,180121 | 1,029966 | 0,722497 | 1 | 1 | 1 |
| Processive synthesis on the C-strand of the telomere | 11 | 11 | 0,180121 | 1,029966 | 0,722497 | 1 | 1 | 1 |
| Unwinding of DNA | 11 | 11 | 0,180121 | 1,029966 | 0,722497 | 1 | 1 | 1 |
| NICD traffics to nucleus | 11 | 11 | 0,180121 | 1,029966 | 0,722497 | 1 | 1 | 1 |
| G-protein beta:gamma signalling | 11 | 11 | 0,180121 | 1,029966 | 0,722497 | 1 | 1 | 1 |
| P2Y receptors | 11 | 11 | 0,180121 | 1,029966 | 0,722497 | 1 | 1 | 1 |
| Notch-HLH transcription pathway | 11 | 11 | 0,180121 | 1,029966 | 0,722497 | 1 | 1 | 1 |
| Purine catabolism | 11 | 11 | 0,180121 | 1,029966 | 0,722497 | 1 | 1 | 1 |
| Recycling of bile acids and salts | 11 | 11 | 0,180121 | 1,029966 | 0,722497 | 1 | 1 | 1 |
| mRNA Decay by 3' to 5' Exoribonuclease | 11 | 11 | 0,180121 | 1,029966 | 0,722497 | 1 | 1 | 1 |
| Caspase-mediated cleavage of cytoskeletal proteins | 11 | 11 | 0,180121 | 1,029966 | 0,722497 | 1 | 1 | 1 |
| TAK1 activates NFkB by phosphorylation and activation of IKKs complex | 11 | 11 | 0,180121 | 1,029966 | 0,722497 | 1 | 1 | 1 |
| The NLRP3 inflammasome | 11 | 11 | 0,180121 | 1,029966 | 0,722497 | 1 | 1 | 1 |
| pyrimidine ribonucleotides de novo biosynthesis | 11 | 11 | 0,180121 | 1,029966 | 0,722497 | 1 | 1 | 1 |
| isoleucine degradation I | 11 | 11 | 0,180121 | 1,029966 | 0,722497 | 1 | 1 | 1 |
| nicotine degradation II | 11 | 11 | 0,180121 | 1,029966 | 0,722497 | 1 | 1 | 1 |
| ALK2 signaling events | 11 | 11 | 0,180121 | 1,029966 | 0,722497 | 1 | 1 | 1 |
| Alpha6 beta4 integrin-ligand interactions | 11 | 11 | 0,180121 | 1,029966 | 0,722497 | 1 | 1 | 1 |
| Role of second messengers in netrin-1 signaling | 10 | 10 | 0,163747 | 1,029966 | 0,744183 | 1 | 1 | 1 |
| Reduction of cytosolic Ca++ levels | 10 | 10 | 0,163747 | 1,029966 | 0,744183 | 1 | 1 | 1 |
| Dissolution of Fibrin Clot | 10 | 10 | 0,163747 | 1,029966 | 0,744183 | 1 | 1 | 1 |
| p130Cas linkage to MAPK signaling for integrins | 10 | 10 | 0,163747 | 1,029966 | 0,744183 | 1 | 1 | 1 |
| ADP signalling through P2Y purinoceptor 1 | 10 | 10 | 0,163747 | 1,029966 | 0,744183 | 1 | 1 | 1 |
| GRB2:SOS provides linkage to MAPK signaling for Intergrins | 10 | 10 | 0,163747 | 1,029966 | 0,744183 | 1 | 1 | 1 |
| SLBP independent Processing of Histone Pre-mRNAs | 10 | 10 | 0,163747 | 1,029966 | 0,744183 | 1 | 1 | 1 |
| DCC mediated attractive signaling | 10 | 10 | 0,163747 | 1,029966 | 0,744183 | 1 | 1 | 1 |
| Regulation of Rheb GTPase activity by AMPK | 10 | 10 | 0,163747 | 1,029966 | 0,744183 | 1 | 1 | 1 |
| E2F-enabled inhibition of pre-replication complex formation | 10 | 10 | 0,163747 | 1,029966 | 0,744183 | 1 | 1 | 1 |
| Removal of the Flap Intermediate from the C-strand | 10 | 10 | 0,163747 | 1,029966 | 0,744183 | 1 | 1 | 1 |
| Adenylate cyclase activating pathway | 10 | 10 | 0,163747 | 1,029966 | 0,744183 | 1 | 1 | 1 |
| Hormone ligand-binding receptors | 10 | 10 | 0,163747 | 1,029966 | 0,744183 | 1 | 1 | 1 |
| Pyruvate metabolism | 10 | 10 | 0,163747 | 1,029966 | 0,744183 | 1 | 1 | 1 |
| Pyrimidine catabolism | 10 | 10 | 0,163747 | 1,029966 | 0,744183 | 1 | 1 | 1 |
| Synthesis of bile acids and bile salts via 24-hydroxycholesterol | 10 | 10 | 0,163747 | 1,029966 | 0,744183 | 1 | 1 | 1 |
| Base-free sugar-phosphate removal via the single-nucleotide replacement pathway | 10 | 10 | 0,163747 | 1,029966 | 0,744183 | 1 | 1 | 1 |
| Removal of aminoterminal propeptides from gamma-carboxylated proteins | 10 | 10 | 0,163747 | 1,029966 | 0,744183 | 1 | 1 | 1 |
| Golgi to ER Retrograde Transport | 10 | 10 | 0,163747 | 1,029966 | 0,744183 | 1 | 1 | 1 |
| COPI Mediated Transport | 10 | 10 | 0,163747 | 1,029966 | 0,744183 | 1 | 1 | 1 |
| Metabolism of porphyrins | 10 | 10 | 0,163747 | 1,029966 | 0,744183 | 1 | 1 | 1 |
| Zinc influx into cells by the SLC39 gene family | 10 | 10 | 0,163747 | 1,029966 | 0,744183 | 1 | 1 | 1 |
| Transport of organic anions | 10 | 10 | 0,163747 | 1,029966 | 0,744183 | 1 | 1 | 1 |
| IRAK2 mediated activation of TAK1 complex upon TLR7/8 or 9 stimulation | 10 | 10 | 0,163747 | 1,029966 | 0,744183 | 1 | 1 | 1 |
| Rap1 signalling | 10 | 10 | 0,163747 | 1,029966 | 0,744183 | 1 | 1 | 1 |
| IRAK2 mediated activation of TAK1 complex | 10 | 10 | 0,163747 | 1,029966 | 0,744183 | 1 | 1 | 1 |
| TRAF3-dependent IRF activation pathway | 10 | 10 | 0,163747 | 1,029966 | 0,744183 | 1 | 1 | 1 |
| mevalonate pathway I | 10 | 10 | 0,163747 | 1,029966 | 0,744183 | 1 | 1 | 1 |
| NAD biosynthesis II (from tryptophan) | 10 | 10 | 0,163747 | 1,029966 | 0,744183 | 1 | 1 | 1 |
| pentose phosphate pathway | 10 | 10 | 0,163747 | 1,029966 | 0,744183 | 1 | 1 | 1 |
| Adaptive Immune System | 229 | 237 | 3,749795 | 0,9952 | 0,746827 | 1 | 1 | 1 |
| Alpha4 beta1 integrin signaling events | 32 | 33 | 0,523989 | 0,998764 | 0,75075 | 1 | 1 | 1 |
| Alpha-synuclein signaling | 32 | 33 | 0,523989 | 0,998764 | 0,75075 | 1 | 1 | 1 |
| Downstream TCR signaling | 31 | 32 | 0,507614 | 0,997789 | 0,76162 | 1 | 1 | 1 |
| Nef mediated downregulation of MHC class I complex cell surface expression | 9 | 9 | 0,147372 | 1,029966 | 0,766515 | 1 | 1 | 1 |
| Nef Mediated CD4 Down-regulation | 9 | 9 | 0,147372 | 1,029966 | 0,766515 | 1 | 1 | 1 |
| Neurofascin interactions | 9 | 9 | 0,147372 | 1,029966 | 0,766515 | 1 | 1 | 1 |
| Role of Abl in Robo-Slit signaling | 9 | 9 | 0,147372 | 1,029966 | 0,766515 | 1 | 1 | 1 |
| GP1b-IX-V activation signalling | 9 | 9 | 0,147372 | 1,029966 | 0,766515 | 1 | 1 | 1 |
| Basigin interactions | 9 | 9 | 0,147372 | 1,029966 | 0,766515 | 1 | 1 | 1 |
| Dopamine Neurotransmitter Release Cycle | 9 | 9 | 0,147372 | 1,029966 | 0,766515 | 1 | 1 | 1 |
| Serotonin Neurotransmitter Release Cycle | 9 | 9 | 0,147372 | 1,029966 | 0,766515 | 1 | 1 | 1 |
| Ca2+ activated K+ channels | 9 | 9 | 0,147372 | 1,029966 | 0,766515 | 1 | 1 | 1 |
| p38MAPK events | 9 | 9 | 0,147372 | 1,029966 | 0,766515 | 1 | 1 | 1 |
| Regulated proteolysis of p75NTR | 9 | 9 | 0,147372 | 1,029966 | 0,766515 | 1 | 1 | 1 |
| AKT phosphorylates targets in the cytosol | 9 | 9 | 0,147372 | 1,029966 | 0,766515 | 1 | 1 | 1 |
| Activation of Chaperones by ATF6-alpha | 9 | 9 | 0,147372 | 1,029966 | 0,766515 | 1 | 1 | 1 |
| Receptor-ligand binding initiates the second proteolytic cleavage of Notch receptor | 9 | 9 | 0,147372 | 1,029966 | 0,766515 | 1 | 1 | 1 |
| Adrenoceptors | 9 | 9 | 0,147372 | 1,029966 | 0,766515 | 1 | 1 | 1 |
| Prostanoid ligand receptors | 9 | 9 | 0,147372 | 1,029966 | 0,766515 | 1 | 1 | 1 |
| Phosphorylation of CD3 and TCR zeta chains | 9 | 9 | 0,147372 | 1,029966 | 0,766515 | 1 | 1 | 1 |
| Leukotriene synthesis | 9 | 9 | 0,147372 | 1,029966 | 0,766515 | 1 | 1 | 1 |
| Displacement of DNA glycosylase by APE1 | 9 | 9 | 0,147372 | 1,029966 | 0,766515 | 1 | 1 | 1 |
| eNOS activation | 9 | 9 | 0,147372 | 1,029966 | 0,766515 | 1 | 1 | 1 |
| N-Glycan antennae elongation | 9 | 9 | 0,147372 | 1,029966 | 0,766515 | 1 | 1 | 1 |
| Folding of actin by CCT/TriC | 9 | 9 | 0,147372 | 1,029966 | 0,766515 | 1 | 1 | 1 |
| Association of TriC/CCT with target proteins during biosynthesis | 9 | 9 | 0,147372 | 1,029966 | 0,766515 | 1 | 1 | 1 |
| Gamma-carboxylation of protein precursors | 9 | 9 | 0,147372 | 1,029966 | 0,766515 | 1 | 1 | 1 |
| ER to Golgi Transport | 9 | 9 | 0,147372 | 1,029966 | 0,766515 | 1 | 1 | 1 |
| COPII (Coat Protein 2) Mediated Vesicle Transport | 9 | 9 | 0,147372 | 1,029966 | 0,766515 | 1 | 1 | 1 |
| Urea cycle | 9 | 9 | 0,147372 | 1,029966 | 0,766515 | 1 | 1 | 1 |
| Regulation of pyruvate dehydrogenase (PDH) complex | 9 | 9 | 0,147372 | 1,029966 | 0,766515 | 1 | 1 | 1 |
| Vitamin B5 (pantothenate) metabolism | 9 | 9 | 0,147372 | 1,029966 | 0,766515 | 1 | 1 | 1 |
| Sodium/Calcium exchangers | 9 | 9 | 0,147372 | 1,029966 | 0,766515 | 1 | 1 | 1 |
| Sodium/Proton exchangers | 9 | 9 | 0,147372 | 1,029966 | 0,766515 | 1 | 1 | 1 |
| Bicarbonate transporters | 9 | 9 | 0,147372 | 1,029966 | 0,766515 | 1 | 1 | 1 |
| ABCA transporters in lipid homeostasis | 9 | 9 | 0,147372 | 1,029966 | 0,766515 | 1 | 1 | 1 |
| DSCAM interactions | 9 | 9 | 0,147372 | 1,029966 | 0,766515 | 1 | 1 | 1 |
| Caspase-8 is formed from procaspase-8 | 9 | 9 | 0,147372 | 1,029966 | 0,766515 | 1 | 1 | 1 |
| Activation of Pro-Caspase 8 | 9 | 9 | 0,147372 | 1,029966 | 0,766515 | 1 | 1 | 1 |
| Activation of IRF3/IRF7 mediated by TBK1/IKK epsilon | 9 | 9 | 0,147372 | 1,029966 | 0,766515 | 1 | 1 | 1 |
| Endosomal/Vacuolar pathway | 9 | 9 | 0,147372 | 1,029966 | 0,766515 | 1 | 1 | 1 |
| Activation of the AP-1 family of transcription factors | 9 | 9 | 0,147372 | 1,029966 | 0,766515 | 1 | 1 | 1 |
| Advanced glycosylation endproduct receptor signaling | 9 | 9 | 0,147372 | 1,029966 | 0,766515 | 1 | 1 | 1 |
| IKK complex recruitment mediated by RIP1 | 9 | 9 | 0,147372 | 1,029966 | 0,766515 | 1 | 1 | 1 |
| Creation of C4 and C2 activators | 9 | 9 | 0,147372 | 1,029966 | 0,766515 | 1 | 1 | 1 |
| heme biosynthesis II | 9 | 9 | 0,147372 | 1,029966 | 0,766515 | 1 | 1 | 1 |
| gamma-glutamyl cycle | 9 | 9 | 0,147372 | 1,029966 | 0,766515 | 1 | 1 | 1 |
| glutathione redox reactions I | 9 | 9 | 0,147372 | 1,029966 | 0,766515 | 1 | 1 | 1 |
| folate transformations | 9 | 9 | 0,147372 | 1,029966 | 0,766515 | 1 | 1 | 1 |
| prostanoid biosynthesis | 9 | 9 | 0,147372 | 1,029966 | 0,766515 | 1 | 1 | 1 |
| choline biosynthesis III | 9 | 9 | 0,147372 | 1,029966 | 0,766515 | 1 | 1 | 1 |
| bile acid biosynthesis, neutral pathway | 9 | 9 | 0,147372 | 1,029966 | 0,766515 | 1 | 1 | 1 |
| pyrimidine ribonucleotides interconversion | 9 | 9 | 0,147372 | 1,029966 | 0,766515 | 1 | 1 | 1 |
| glycine betaine degradation | 9 | 9 | 0,147372 | 1,029966 | 0,766515 | 1 | 1 | 1 |
| glycerol degradation I | 9 | 9 | 0,147372 | 1,029966 | 0,766515 | 1 | 1 | 1 |
| tryptophan degradation III (eukaryotic) | 9 | 9 | 0,147372 | 1,029966 | 0,766515 | 1 | 1 | 1 |
| adenosine nucleotides degradation II | 9 | 9 | 0,147372 | 1,029966 | 0,766515 | 1 | 1 | 1 |
| a4b7 Integrin signaling | 9 | 9 | 0,147372 | 1,029966 | 0,766515 | 1 | 1 | 1 |
| Rapid glucocorticoid signaling | 9 | 9 | 0,147372 | 1,029966 | 0,766515 | 1 | 1 | 1 |
| Effects of Botulinum toxin | 9 | 9 | 0,147372 | 1,029966 | 0,766515 | 1 | 1 | 1 |
| PLC-gamma1 signalling | 29 | 30 | 0,474865 | 0,995645 | 0,783273 | 1 | 1 | 1 |
| Transport of Mature Transcript to Cytoplasm | 53 | 55 | 0,867857 | 0,992519 | 0,785372 | 1 | 1 | 1 |
| Nef and signal transduction | 8 | 8 | 0,130997 | 1,029966 | 0,789515 | 1 | 1 | 1 |
| Sema4D mediated inhibition of cell attachment and migration | 8 | 8 | 0,130997 | 1,029966 | 0,789515 | 1 | 1 | 1 |
| Inactivation of Cdc42 and Rac | 8 | 8 | 0,130997 | 1,029966 | 0,789515 | 1 | 1 | 1 |
| Activation of Rac | 8 | 8 | 0,130997 | 1,029966 | 0,789515 | 1 | 1 | 1 |
| Platelet sensitization by LDL | 8 | 8 | 0,130997 | 1,029966 | 0,789515 | 1 | 1 | 1 |
| Disinhibition of SNARE formation | 8 | 8 | 0,130997 | 1,029966 | 0,789515 | 1 | 1 | 1 |
| Thromboxane signalling through TP receptor | 8 | 8 | 0,130997 | 1,029966 | 0,789515 | 1 | 1 | 1 |
| Activation of Kainate Receptors upon glutamate binding | 8 | 8 | 0,130997 | 1,029966 | 0,789515 | 1 | 1 | 1 |
| Integration of provirus | 8 | 8 | 0,130997 | 1,029966 | 0,789515 | 1 | 1 | 1 |
| NRAGE signals death through JNK | 8 | 8 | 0,130997 | 1,029966 | 0,789515 | 1 | 1 | 1 |
| p75NTR regulates axonogenesis | 8 | 8 | 0,130997 | 1,029966 | 0,789515 | 1 | 1 | 1 |
| Inhibition of replication initiation of damaged DNA by Rb/E2F1 | 8 | 8 | 0,130997 | 1,029966 | 0,789515 | 1 | 1 | 1 |
| Notch receptor binds with a ligand | 8 | 8 | 0,130997 | 1,029966 | 0,789515 | 1 | 1 | 1 |
| Signaling by Rho GTPases | 8 | 8 | 0,130997 | 1,029966 | 0,789515 | 1 | 1 | 1 |
| Rho GTPase cycle | 8 | 8 | 0,130997 | 1,029966 | 0,789515 | 1 | 1 | 1 |
| A third proteolytic cleavage releases NICD | 8 | 8 | 0,130997 | 1,029966 | 0,789515 | 1 | 1 | 1 |
| Calcitonin-like ligand receptors | 8 | 8 | 0,130997 | 1,029966 | 0,789515 | 1 | 1 | 1 |
| G beta:gamma signalling through PI3Kgamma | 8 | 8 | 0,130997 | 1,029966 | 0,789515 | 1 | 1 | 1 |
| Orexin and neuropeptides FF and QRFP bind to their respective receptors | 8 | 8 | 0,130997 | 1,029966 | 0,789515 | 1 | 1 | 1 |
| Lysosphingolipid and LPA receptors | 8 | 8 | 0,130997 | 1,029966 | 0,789515 | 1 | 1 | 1 |
| Androgen biosynthesis | 8 | 8 | 0,130997 | 1,029966 | 0,789515 | 1 | 1 | 1 |
| Beta-oxidation of pristanoyl-CoA | 8 | 8 | 0,130997 | 1,029966 | 0,789515 | 1 | 1 | 1 |
| Base-Excision Repair, AP Site Formation | 8 | 8 | 0,130997 | 1,029966 | 0,789515 | 1 | 1 | 1 |
| Regulation of the Fanconi anemia pathway | 8 | 8 | 0,130997 | 1,029966 | 0,789515 | 1 | 1 | 1 |
| Formation of tubulin folding intermediates by CCT/TriC | 8 | 8 | 0,130997 | 1,029966 | 0,789515 | 1 | 1 | 1 |
| Transport of gamma-carboxylated protein precursors from the endoplasmic reticulum to the Golgi apparatus | 8 | 8 | 0,130997 | 1,029966 | 0,789515 | 1 | 1 | 1 |
| Recycling of eIF2:GDP | 8 | 8 | 0,130997 | 1,029966 | 0,789515 | 1 | 1 | 1 |
| Stabilization of mRNA by HuR | 8 | 8 | 0,130997 | 1,029966 | 0,789515 | 1 | 1 | 1 |
| Lysine catabolism | 8 | 8 | 0,130997 | 1,029966 | 0,789515 | 1 | 1 | 1 |
| Nicotinate metabolism | 8 | 8 | 0,130997 | 1,029966 | 0,789515 | 1 | 1 | 1 |
| Metabolism of folate and pterines | 8 | 8 | 0,130997 | 1,029966 | 0,789515 | 1 | 1 | 1 |
| Import of palmitoyl-CoA into the mitochondrial matrix | 8 | 8 | 0,130997 | 1,029966 | 0,789515 | 1 | 1 | 1 |
| mitochondrial fatty acid beta-oxidation of saturated fatty acids | 8 | 8 | 0,130997 | 1,029966 | 0,789515 | 1 | 1 | 1 |
| Regulation of cytoskeletal remodeling and cell spreading by IPP complex components | 8 | 8 | 0,130997 | 1,029966 | 0,789515 | 1 | 1 | 1 |
| Type I hemidesmosome assembly | 8 | 8 | 0,130997 | 1,029966 | 0,789515 | 1 | 1 | 1 |
| Transport of nucleotide sugars | 8 | 8 | 0,130997 | 1,029966 | 0,789515 | 1 | 1 | 1 |
| Role of DCC in regulating apoptosis | 8 | 8 | 0,130997 | 1,029966 | 0,789515 | 1 | 1 | 1 |
| Interleukin-6 signaling | 8 | 8 | 0,130997 | 1,029966 | 0,789515 | 1 | 1 | 1 |
| NF-kB activation through FADD/RIP-1 pathway mediated by caspase-8 and -10 | 8 | 8 | 0,130997 | 1,029966 | 0,789515 | 1 | 1 | 1 |
| citrulline biosynthesis | 8 | 8 | 0,130997 | 1,029966 | 0,789515 | 1 | 1 | 1 |
| cysteine biosynthesis III (mammalia) | 8 | 8 | 0,130997 | 1,029966 | 0,789515 | 1 | 1 | 1 |
| NAD salvage pathway II | 8 | 8 | 0,130997 | 1,029966 | 0,789515 | 1 | 1 | 1 |
| androgen biosynthesis | 8 | 8 | 0,130997 | 1,029966 | 0,789515 | 1 | 1 | 1 |
| S1P5 pathway | 8 | 8 | 0,130997 | 1,029966 | 0,789515 | 1 | 1 | 1 |
| Thromboxane A2 receptor signaling | 52 | 54 | 0,851482 | 0,991826 | 0,792982 | 1 | 1 | 1 |
| EGFR interacts with phospholipase C-gamma | 28 | 29 | 0,45849 | 0,994462 | 0,794031 | 1 | 1 | 1 |
| Nephrin interactions | 28 | 29 | 0,45849 | 0,994462 | 0,794031 | 1 | 1 | 1 |
| BARD1 signaling events | 28 | 29 | 0,45849 | 0,994462 | 0,794031 | 1 | 1 | 1 |
| Triglyceride Biosynthesis | 27 | 28 | 0,442116 | 0,993194 | 0,804727 | 1 | 1 | 1 |
| Beta2 integrin cell surface interactions | 27 | 28 | 0,442116 | 0,993194 | 0,804727 | 1 | 1 | 1 |
| IL8- and CXCR1-mediated signaling events | 27 | 28 | 0,442116 | 0,993194 | 0,804727 | 1 | 1 | 1 |
| p73 transcription factor network | 75 | 78 | 1,228099 | 0,990357 | 0,808728 | 1 | 1 | 1 |
| Nef Mediated CD8 Down-regulation | 7 | 7 | 0,114623 | 1,029966 | 0,8132 | 1 | 1 | 1 |
| NrCAM interactions | 7 | 7 | 0,114623 | 1,029966 | 0,8132 | 1 | 1 | 1 |
| PECAM1 interactions | 7 | 7 | 0,114623 | 1,029966 | 0,8132 | 1 | 1 | 1 |
| MEK activation | 7 | 7 | 0,114623 | 1,029966 | 0,8132 | 1 | 1 | 1 |
| RAF phosphorylates MEK | 7 | 7 | 0,114623 | 1,029966 | 0,8132 | 1 | 1 | 1 |
| Trafficking of GluR2-containing AMPA receptors | 7 | 7 | 0,114623 | 1,029966 | 0,8132 | 1 | 1 | 1 |
| RSK activation | 7 | 7 | 0,114623 | 1,029966 | 0,8132 | 1 | 1 | 1 |
| 2-LTR circle formation | 7 | 7 | 0,114623 | 1,029966 | 0,8132 | 1 | 1 | 1 |
| Axonal growth inhibition (RHOA activation) | 7 | 7 | 0,114623 | 1,029966 | 0,8132 | 1 | 1 | 1 |
| FGFR2b ligand binding and activation | 7 | 7 | 0,114623 | 1,029966 | 0,8132 | 1 | 1 | 1 |
| FGFR1 ligand binding and activation | 7 | 7 | 0,114623 | 1,029966 | 0,8132 | 1 | 1 | 1 |
| FGFR2 ligand binding and activation | 7 | 7 | 0,114623 | 1,029966 | 0,8132 | 1 | 1 | 1 |
| CREB phosphorylation | 7 | 7 | 0,114623 | 1,029966 | 0,8132 | 1 | 1 | 1 |
| Translocation of ZAP-70 to Immunological synapse | 7 | 7 | 0,114623 | 1,029966 | 0,8132 | 1 | 1 | 1 |
| Beta-catenin phosphorylation cascade | 7 | 7 | 0,114623 | 1,029966 | 0,8132 | 1 | 1 | 1 |
| Pyrimidine salvage reactions | 7 | 7 | 0,114623 | 1,029966 | 0,8132 | 1 | 1 | 1 |
| Purine salvage | 7 | 7 | 0,114623 | 1,029966 | 0,8132 | 1 | 1 | 1 |
| Purine ribonucleoside monophosphate biosynthesis | 7 | 7 | 0,114623 | 1,029966 | 0,8132 | 1 | 1 | 1 |
| Vitamin A uptake in enterocytes | 7 | 7 | 0,114623 | 1,029966 | 0,8132 | 1 | 1 | 1 |
| Vitamin D (calciferol) metabolism | 7 | 7 | 0,114623 | 1,029966 | 0,8132 | 1 | 1 | 1 |
| Homologous DNA pairing and strand exchange | 7 | 7 | 0,114623 | 1,029966 | 0,8132 | 1 | 1 | 1 |
| Attachment of GPI anchor to uPAR | 7 | 7 | 0,114623 | 1,029966 | 0,8132 | 1 | 1 | 1 |
| Cytosolic sulfonation of small molecules | 7 | 7 | 0,114623 | 1,029966 | 0,8132 | 1 | 1 | 1 |
| Heme biosynthesis | 7 | 7 | 0,114623 | 1,029966 | 0,8132 | 1 | 1 | 1 |
| Cation-coupled Chloride cotransporters | 7 | 7 | 0,114623 | 1,029966 | 0,8132 | 1 | 1 | 1 |
| Zinc efflux and compartmentalization by the SLC30 family | 7 | 7 | 0,114623 | 1,029966 | 0,8132 | 1 | 1 | 1 |
| Multifunctional anion exchangers | 7 | 7 | 0,114623 | 1,029966 | 0,8132 | 1 | 1 | 1 |
| Organic anion transporters | 7 | 7 | 0,114623 | 1,029966 | 0,8132 | 1 | 1 | 1 |
| Organic cation transport | 7 | 7 | 0,114623 | 1,029966 | 0,8132 | 1 | 1 | 1 |
| Transport of nucleosides and free purine and pyrimidine bases across the plasma membrane | 7 | 7 | 0,114623 | 1,029966 | 0,8132 | 1 | 1 | 1 |
| Apoptotic factor-mediated response | 7 | 7 | 0,114623 | 1,029966 | 0,8132 | 1 | 1 | 1 |
| Activation of BAD and translocation to mitochondria | 7 | 7 | 0,114623 | 1,029966 | 0,8132 | 1 | 1 | 1 |
| TNF signaling | 7 | 7 | 0,114623 | 1,029966 | 0,8132 | 1 | 1 | 1 |
| JNK (c-Jun kinases) phosphorylation and activation mediated by activated human TAK1 | 7 | 7 | 0,114623 | 1,029966 | 0,8132 | 1 | 1 | 1 |
| Interleukin receptor SHC signaling | 7 | 7 | 0,114623 | 1,029966 | 0,8132 | 1 | 1 | 1 |
| Terminal pathway of complement | 7 | 7 | 0,114623 | 1,029966 | 0,8132 | 1 | 1 | 1 |
| dolichyl-diphosphooligosaccharide biosynthesis | 7 | 7 | 0,114623 | 1,029966 | 0,8132 | 1 | 1 | 1 |
| glycoaminoglycan-protein linkage region biosynthesis | 7 | 7 | 0,114623 | 1,029966 | 0,8132 | 1 | 1 | 1 |
| coenzyme A biosynthesis | 7 | 7 | 0,114623 | 1,029966 | 0,8132 | 1 | 1 | 1 |
| sphingomyelin metabolism | 7 | 7 | 0,114623 | 1,029966 | 0,8132 | 1 | 1 | 1 |
| gamma-linolenate biosynthesis II (animals) | 7 | 7 | 0,114623 | 1,029966 | 0,8132 | 1 | 1 | 1 |
| oleate biosynthesis II (animals) | 7 | 7 | 0,114623 | 1,029966 | 0,8132 | 1 | 1 | 1 |
| inositol pyrophosphates biosynthesis | 7 | 7 | 0,114623 | 1,029966 | 0,8132 | 1 | 1 | 1 |
| dopamine degradation | 7 | 7 | 0,114623 | 1,029966 | 0,8132 | 1 | 1 | 1 |
| ethanol degradation IV (peroxisomal) | 7 | 7 | 0,114623 | 1,029966 | 0,8132 | 1 | 1 | 1 |
| histidine degradation III | 7 | 7 | 0,114623 | 1,029966 | 0,8132 | 1 | 1 | 1 |
| leucine degradation I | 7 | 7 | 0,114623 | 1,029966 | 0,8132 | 1 | 1 | 1 |
| sucrose degradation | 7 | 7 | 0,114623 | 1,029966 | 0,8132 | 1 | 1 | 1 |
| sphingosine and sphingosine-1-phosphate metabolism | 7 | 7 | 0,114623 | 1,029966 | 0,8132 | 1 | 1 | 1 |
| acetone degradation I (to methylglyoxal) | 7 | 7 | 0,114623 | 1,029966 | 0,8132 | 1 | 1 | 1 |
| serotonin degradation | 7 | 7 | 0,114623 | 1,029966 | 0,8132 | 1 | 1 | 1 |
| noradrenaline and adrenaline degradation | 7 | 7 | 0,114623 | 1,029966 | 0,8132 | 1 | 1 | 1 |
| Phospholipase C-mediated cascade | 26 | 27 | 0,425741 | 0,991833 | 0,815349 | 1 | 1 | 1 |
| DAG and IP3 signaling | 26 | 27 | 0,425741 | 0,991833 | 0,815349 | 1 | 1 | 1 |
| Ca-dependent events | 26 | 27 | 0,425741 | 0,991833 | 0,815349 | 1 | 1 | 1 |
| Transport of Mature mRNA derived from an Intron-Containing Transcript | 49 | 51 | 0,802358 | 0,989583 | 0,815407 | 1 | 1 | 1 |
| Metabolism of non-coding RNA | 48 | 50 | 0,785983 | 0,988775 | 0,822731 | 1 | 1 | 1 |
| snRNP Assembly | 48 | 50 | 0,785983 | 0,988775 | 0,822731 | 1 | 1 | 1 |
| M Phase | 152 | 158 | 2,488947 | 0,990855 | 0,823648 | 1 | 1 | 1 |
| Epithelial-to-mesenchymal transition | 178 | 185 | 2,914688 | 0,990996 | 0,825704 | 1 | 1 | 1 |
| NOD1/2 Signaling Pathway | 25 | 26 | 0,409366 | 0,990367 | 0,825881 | 1 | 1 | 1 |
| Calmodulin induced events | 24 | 25 | 0,392992 | 0,988783 | 0,83631 | 1 | 1 | 1 |
| CaM pathway | 24 | 25 | 0,392992 | 0,988783 | 0,83631 | 1 | 1 | 1 |
| Nephrin/Neph1 signaling in the kidney podocyte | 24 | 25 | 0,392992 | 0,988783 | 0,83631 | 1 | 1 | 1 |
| VEGFR3 signaling in lymphatic endothelium | 24 | 25 | 0,392992 | 0,988783 | 0,83631 | 1 | 1 | 1 |
| Regulation of gene expression in late stage (branching morphogenesis) pancreatic bud precursor cells | 6 | 6 | 0,098248 | 1,029966 | 0,837592 | 1 | 1 | 1 |
| Regulation of gene expression in early pancreatic precursor cells | 6 | 6 | 0,098248 | 1,029966 | 0,837592 | 1 | 1 | 1 |
| ADP signalling through P2Y purinoceptor 12 | 6 | 6 | 0,098248 | 1,029966 | 0,837592 | 1 | 1 | 1 |
| ERK activation | 6 | 6 | 0,098248 | 1,029966 | 0,837592 | 1 | 1 | 1 |
| Neurotransmitter Clearance In The Synaptic Cleft | 6 | 6 | 0,098248 | 1,029966 | 0,837592 | 1 | 1 | 1 |
| Presynaptic function of Kainate receptors | 6 | 6 | 0,098248 | 1,029966 | 0,837592 | 1 | 1 | 1 |
| CREB phosphorylation through the activation of CaMKK | 6 | 6 | 0,098248 | 1,029966 | 0,837592 | 1 | 1 | 1 |
| Release of eIF4E | 6 | 6 | 0,098248 | 1,029966 | 0,837592 | 1 | 1 | 1 |
| Activation of TRKA receptors | 6 | 6 | 0,098248 | 1,029966 | 0,837592 | 1 | 1 | 1 |
| FGFR1b ligand binding and activation | 6 | 6 | 0,098248 | 1,029966 | 0,837592 | 1 | 1 | 1 |
| SHC-mediated cascade | 6 | 6 | 0,098248 | 1,029966 | 0,837592 | 1 | 1 | 1 |
| Klotho-mediated ligand binding | 6 | 6 | 0,098248 | 1,029966 | 0,837592 | 1 | 1 | 1 |
| Assembly of the ORC complex at the origin of replication | 6 | 6 | 0,098248 | 1,029966 | 0,837592 | 1 | 1 | 1 |
| Cyclin B2 mediated events | 6 | 6 | 0,098248 | 1,029966 | 0,837592 | 1 | 1 | 1 |
| Telomere Extension By Telomerase | 6 | 6 | 0,098248 | 1,029966 | 0,837592 | 1 | 1 | 1 |
| Telomere C-strand synthesis initiation | 6 | 6 | 0,098248 | 1,029966 | 0,837592 | 1 | 1 | 1 |
| DNA replication initiation | 6 | 6 | 0,098248 | 1,029966 | 0,837592 | 1 | 1 | 1 |
| Vasopressin-like receptors | 6 | 6 | 0,098248 | 1,029966 | 0,837592 | 1 | 1 | 1 |
| Pyrimidine biosynthesis | 6 | 6 | 0,098248 | 1,029966 | 0,837592 | 1 | 1 | 1 |
| Synthesis of bile acids and bile salts via 27-hydroxycholesterol | 6 | 6 | 0,098248 | 1,029966 | 0,837592 | 1 | 1 | 1 |
| Beta-oxidation of very long chain fatty acids | 6 | 6 | 0,098248 | 1,029966 | 0,837592 | 1 | 1 | 1 |
| mitochondrial fatty acid beta-oxidation of unsaturated fatty acids | 6 | 6 | 0,098248 | 1,029966 | 0,837592 | 1 | 1 | 1 |
| Resolution of D-loop structures through Holliday junction intermediates | 6 | 6 | 0,098248 | 1,029966 | 0,837592 | 1 | 1 | 1 |
| Resolution of D-loop structures | 6 | 6 | 0,098248 | 1,029966 | 0,837592 | 1 | 1 | 1 |
| Presynaptic phase of homologous DNA pairing and strand exchange | 6 | 6 | 0,098248 | 1,029966 | 0,837592 | 1 | 1 | 1 |
| ER Quality Control Compartment (ERQC) | 6 | 6 | 0,098248 | 1,029966 | 0,837592 | 1 | 1 | 1 |
| Gap junction trafficking and regulation | 6 | 6 | 0,098248 | 1,029966 | 0,837592 | 1 | 1 | 1 |
| Vitamins | 6 | 6 | 0,098248 | 1,029966 | 0,837592 | 1 | 1 | 1 |
| Glucuronidation | 6 | 6 | 0,098248 | 1,029966 | 0,837592 | 1 | 1 | 1 |
| Methylation | 6 | 6 | 0,098248 | 1,029966 | 0,837592 | 1 | 1 | 1 |
| Glutathione synthesis and recycling | 6 | 6 | 0,098248 | 1,029966 | 0,837592 | 1 | 1 | 1 |
| Thyroxine biosynthesis | 6 | 6 | 0,098248 | 1,029966 | 0,837592 | 1 | 1 | 1 |
| Methionine salvage pathway | 6 | 6 | 0,098248 | 1,029966 | 0,837592 | 1 | 1 | 1 |
| ChREBP activates metabolic gene expression | 6 | 6 | 0,098248 | 1,029966 | 0,837592 | 1 | 1 | 1 |
| Molybdenum cofactor biosynthesis | 6 | 6 | 0,098248 | 1,029966 | 0,837592 | 1 | 1 | 1 |
| Coenzyme A biosynthesis | 6 | 6 | 0,098248 | 1,029966 | 0,837592 | 1 | 1 | 1 |
| Vitamin C (ascorbate) metabolism | 6 | 6 | 0,098248 | 1,029966 | 0,837592 | 1 | 1 | 1 |
| Pentose phosphate pathway (hexose monophosphate shunt) | 6 | 6 | 0,098248 | 1,029966 | 0,837592 | 1 | 1 | 1 |
| TRAIL signaling | 6 | 6 | 0,098248 | 1,029966 | 0,837592 | 1 | 1 | 1 |
| Viral dsRNA:TLR3:TRIF Complex Activates TBK1/IKK epsilon | 6 | 6 | 0,098248 | 1,029966 | 0,837592 | 1 | 1 | 1 |
| activated TAK1 mediates p38 MAPK activation | 6 | 6 | 0,098248 | 1,029966 | 0,837592 | 1 | 1 | 1 |
| Interleukin-1 processing | 6 | 6 | 0,098248 | 1,029966 | 0,837592 | 1 | 1 | 1 |
| Regulation of signaling by CBL | 6 | 6 | 0,098248 | 1,029966 | 0,837592 | 1 | 1 | 1 |
| Classical antibody-mediated complement activation | 6 | 6 | 0,098248 | 1,029966 | 0,837592 | 1 | 1 | 1 |
| fatty acid activation | 6 | 6 | 0,098248 | 1,029966 | 0,837592 | 1 | 1 | 1 |
| arginine biosynthesis IV | 6 | 6 | 0,098248 | 1,029966 | 0,837592 | 1 | 1 | 1 |
| GDP-mannose biosynthesis | 6 | 6 | 0,098248 | 1,029966 | 0,837592 | 1 | 1 | 1 |
| glycogen biosynthesis II (from UDP-D-Glucose) | 6 | 6 | 0,098248 | 1,029966 | 0,837592 | 1 | 1 | 1 |
| chondroitin and dermatan biosynthesis | 6 | 6 | 0,098248 | 1,029966 | 0,837592 | 1 | 1 | 1 |
| leukotriene biosynthesis | 6 | 6 | 0,098248 | 1,029966 | 0,837592 | 1 | 1 | 1 |
| phosphatidylcholine biosynthesis I | 6 | 6 | 0,098248 | 1,029966 | 0,837592 | 1 | 1 | 1 |
| zymosterol biosynthesis | 6 | 6 | 0,098248 | 1,029966 | 0,837592 | 1 | 1 | 1 |
| oxidative ethanol degradation III (microsomal) | 6 | 6 | 0,098248 | 1,029966 | 0,837592 | 1 | 1 | 1 |
| ethanol degradation II (cytosol) | 6 | 6 | 0,098248 | 1,029966 | 0,837592 | 1 | 1 | 1 |
| methionine degradation I (to homocysteine) | 6 | 6 | 0,098248 | 1,029966 | 0,837592 | 1 | 1 | 1 |
| tryptophan degradation X (mammalian, via tryptamine) | 6 | 6 | 0,098248 | 1,029966 | 0,837592 | 1 | 1 | 1 |
| acetyl-CoA biosynthesis (from pyruvate) | 6 | 6 | 0,098248 | 1,029966 | 0,837592 | 1 | 1 | 1 |
| urea cycle | 6 | 6 | 0,098248 | 1,029966 | 0,837592 | 1 | 1 | 1 |
| nicotine degradation III | 6 | 6 | 0,098248 | 1,029966 | 0,837592 | 1 | 1 | 1 |
| pentose phosphate pathway (non-oxidative branch) | 6 | 6 | 0,098248 | 1,029966 | 0,837592 | 1 | 1 | 1 |
| Alternative NF-kappaB pathway | 6 | 6 | 0,098248 | 1,029966 | 0,837592 | 1 | 1 | 1 |
| Signaling by Aurora kinases | 94 | 98 | 1,539217 | 0,98793 | 0,843826 | 1 | 1 | 1 |
| TCR signaling | 45 | 47 | 0,736859 | 0,986147 | 0,844177 | 1 | 1 | 1 |
| APOBEC3G mediated resistance to HIV-1 infection | 5 | 5 | 0,081873 | 1,029966 | 0,862712 | 1 | 1 | 1 |
| CHL1 interactions | 5 | 5 | 0,081873 | 1,029966 | 0,862712 | 1 | 1 | 1 |
| Regulation of gene expression in endocrine-committed (NEUROG3+) progenitor cells | 5 | 5 | 0,081873 | 1,029966 | 0,862712 | 1 | 1 | 1 |
| Extrinsic Pathway | 5 | 5 | 0,081873 | 1,029966 | 0,862712 | 1 | 1 | 1 |
| RAF activation | 5 | 5 | 0,081873 | 1,029966 | 0,862712 | 1 | 1 | 1 |
| Transmission across Electrical Synapses | 5 | 5 | 0,081873 | 1,029966 | 0,862712 | 1 | 1 | 1 |
| Electric Transmission Across Gap Junctions | 5 | 5 | 0,081873 | 1,029966 | 0,862712 | 1 | 1 | 1 |
| Tandem pore domain potassium channels | 5 | 5 | 0,081873 | 1,029966 | 0,862712 | 1 | 1 | 1 |
| GABA A receptor activation | 5 | 5 | 0,081873 | 1,029966 | 0,862712 | 1 | 1 | 1 |
| CREB phosphorylation through the activation of Adenylate Cyclase | 5 | 5 | 0,081873 | 1,029966 | 0,862712 | 1 | 1 | 1 |
| Activation of CaMK IV | 5 | 5 | 0,081873 | 1,029966 | 0,862712 | 1 | 1 | 1 |
| Botulinum neurotoxicity | 5 | 5 | 0,081873 | 1,029966 | 0,862712 | 1 | 1 | 1 |
| S6K1 signalling | 5 | 5 | 0,081873 | 1,029966 | 0,862712 | 1 | 1 | 1 |
| NGF-independant TRKA activation | 5 | 5 | 0,081873 | 1,029966 | 0,862712 | 1 | 1 | 1 |
| Axonal growth stimulation | 5 | 5 | 0,081873 | 1,029966 | 0,862712 | 1 | 1 | 1 |
| Negative regulation of the PI3K/AKT network | 5 | 5 | 0,081873 | 1,029966 | 0,862712 | 1 | 1 | 1 |
| IRS activation | 5 | 5 | 0,081873 | 1,029966 | 0,862712 | 1 | 1 | 1 |
| CaMK IV-mediated phosphorylation of CREB | 5 | 5 | 0,081873 | 1,029966 | 0,862712 | 1 | 1 | 1 |
| Maturation of Notch precursor via proteolytic cleavage | 5 | 5 | 0,081873 | 1,029966 | 0,862712 | 1 | 1 | 1 |
| Tachykinin receptors bind tachykinins | 5 | 5 | 0,081873 | 1,029966 | 0,862712 | 1 | 1 | 1 |
| Signaling by VEGF | 5 | 5 | 0,081873 | 1,029966 | 0,862712 | 1 | 1 | 1 |
| VEGF ligand-receptor interactions | 5 | 5 | 0,081873 | 1,029966 | 0,862712 | 1 | 1 | 1 |
| G beta:gamma signalling through PLC beta | 5 | 5 | 0,081873 | 1,029966 | 0,862712 | 1 | 1 | 1 |
| Relaxin receptors | 5 | 5 | 0,081873 | 1,029966 | 0,862712 | 1 | 1 | 1 |
| Formyl peptide receptors bind formyl peptides and many other ligands | 5 | 5 | 0,081873 | 1,029966 | 0,862712 | 1 | 1 | 1 |
| Dopamine receptors | 5 | 5 | 0,081873 | 1,029966 | 0,862712 | 1 | 1 | 1 |
| Muscarinic acetylcholine receptors | 5 | 5 | 0,081873 | 1,029966 | 0,862712 | 1 | 1 | 1 |
| Leukotriene receptors | 5 | 5 | 0,081873 | 1,029966 | 0,862712 | 1 | 1 | 1 |
| Opsins | 5 | 5 | 0,081873 | 1,029966 | 0,862712 | 1 | 1 | 1 |
| Mineralocorticoid biosynthesis | 5 | 5 | 0,081873 | 1,029966 | 0,862712 | 1 | 1 | 1 |
| Glucocorticoid biosynthesis | 5 | 5 | 0,081873 | 1,029966 | 0,862712 | 1 | 1 | 1 |
| Ketone body metabolism | 5 | 5 | 0,081873 | 1,029966 | 0,862712 | 1 | 1 | 1 |
| Assembly of the RAD51-ssDNA nucleoprotein complex | 5 | 5 | 0,081873 | 1,029966 | 0,862712 | 1 | 1 | 1 |
| Nonhomologous End-joining (NHEJ) | 5 | 5 | 0,081873 | 1,029966 | 0,862712 | 1 | 1 | 1 |
| Recognition and association of DNA glycosylase with site containing an affected pyrimidine | 5 | 5 | 0,081873 | 1,029966 | 0,862712 | 1 | 1 | 1 |
| Depyrimidination | 5 | 5 | 0,081873 | 1,029966 | 0,862712 | 1 | 1 | 1 |
| Cleavage of the damaged pyrimidine | 5 | 5 | 0,081873 | 1,029966 | 0,862712 | 1 | 1 | 1 |
| Chk1/Chk2(Cds1) mediated inactivation of Cyclin B:Cdk1 complex | 5 | 5 | 0,081873 | 1,029966 | 0,862712 | 1 | 1 | 1 |
| Synthesis of GDP-mannose | 5 | 5 | 0,081873 | 1,029966 | 0,862712 | 1 | 1 | 1 |
| Synthesis of UDP-N-acetyl-glucosamine | 5 | 5 | 0,081873 | 1,029966 | 0,862712 | 1 | 1 | 1 |
| N-glycan trimming and elongation in the cis-Golgi | 5 | 5 | 0,081873 | 1,029966 | 0,862712 | 1 | 1 | 1 |
| Host Interactions with Influenza Factors | 5 | 5 | 0,081873 | 1,029966 | 0,862712 | 1 | 1 | 1 |
| Gap junction trafficking | 5 | 5 | 0,081873 | 1,029966 | 0,862712 | 1 | 1 | 1 |
| Eicosanoids | 5 | 5 | 0,081873 | 1,029966 | 0,862712 | 1 | 1 | 1 |
| Miscellaneous substrates | 5 | 5 | 0,081873 | 1,029966 | 0,862712 | 1 | 1 | 1 |
| Glyoxylate metabolism | 5 | 5 | 0,081873 | 1,029966 | 0,862712 | 1 | 1 | 1 |
| Serotonin and melatonin biosynthesis | 5 | 5 | 0,081873 | 1,029966 | 0,862712 | 1 | 1 | 1 |
| AMPK inhibits chREBP transcriptional activation activity | 5 | 5 | 0,081873 | 1,029966 | 0,862712 | 1 | 1 | 1 |
| PKA-mediated phosphorylation of key metabolic factors | 5 | 5 | 0,081873 | 1,029966 | 0,862712 | 1 | 1 | 1 |
| Glycolysis | 5 | 5 | 0,081873 | 1,029966 | 0,862712 | 1 | 1 | 1 |
| Digestion of dietary carbohydrate | 5 | 5 | 0,081873 | 1,029966 | 0,862712 | 1 | 1 | 1 |
| Beta oxidation of octanoyl-CoA to hexanoyl-CoA | 5 | 5 | 0,081873 | 1,029966 | 0,862712 | 1 | 1 | 1 |
| Beta oxidation of hexanoyl-CoA to butanoyl-CoA | 5 | 5 | 0,081873 | 1,029966 | 0,862712 | 1 | 1 | 1 |
| Beta oxidation of decanoyl-CoA to octanoyl-CoA-CoA | 5 | 5 | 0,081873 | 1,029966 | 0,862712 | 1 | 1 | 1 |
| Beta oxidation of lauroyl-CoA to decanoyl-CoA-CoA | 5 | 5 | 0,081873 | 1,029966 | 0,862712 | 1 | 1 | 1 |
| Sodium-coupled sulphate, di- and tri-carboxylate transporters | 5 | 5 | 0,081873 | 1,029966 | 0,862712 | 1 | 1 | 1 |
| Sodium-coupled phosphate cotransporters | 5 | 5 | 0,081873 | 1,029966 | 0,862712 | 1 | 1 | 1 |
| Organic anion transport | 5 | 5 | 0,081873 | 1,029966 | 0,862712 | 1 | 1 | 1 |
| CTLA4 inhibitory signaling | 5 | 5 | 0,081873 | 1,029966 | 0,862712 | 1 | 1 | 1 |
| SMAC binds to IAPs | 5 | 5 | 0,081873 | 1,029966 | 0,862712 | 1 | 1 | 1 |
| SMAC-mediated apoptotic response | 5 | 5 | 0,081873 | 1,029966 | 0,862712 | 1 | 1 | 1 |
| SMAC-mediated dissociation of IAP:caspase complexes | 5 | 5 | 0,081873 | 1,029966 | 0,862712 | 1 | 1 | 1 |
| Activation of caspases through apoptosome-mediated cleavage | 5 | 5 | 0,081873 | 1,029966 | 0,862712 | 1 | 1 | 1 |
| Cytochrome c-mediated apoptotic response | 5 | 5 | 0,081873 | 1,029966 | 0,862712 | 1 | 1 | 1 |
| Activation, myristolyation of BID and translocation to mitochondria | 5 | 5 | 0,081873 | 1,029966 | 0,862712 | 1 | 1 | 1 |
| Activation of BIM and translocation to mitochondria | 5 | 5 | 0,081873 | 1,029966 | 0,862712 | 1 | 1 | 1 |
| FasL/ CD95L signaling | 5 | 5 | 0,081873 | 1,029966 | 0,862712 | 1 | 1 | 1 |
| protein citrullination | 5 | 5 | 0,081873 | 1,029966 | 0,862712 | 1 | 1 | 1 |
| NAD biosynthesis from 2-amino-3-carboxymuconate semialdehyde | 5 | 5 | 0,081873 | 1,029966 | 0,862712 | 1 | 1 | 1 |
| NAD salvage pathway III | 5 | 5 | 0,081873 | 1,029966 | 0,862712 | 1 | 1 | 1 |
| tetrapyrrole biosynthesis II | 5 | 5 | 0,081873 | 1,029966 | 0,862712 | 1 | 1 | 1 |
| folate polyglutamylation | 5 | 5 | 0,081873 | 1,029966 | 0,862712 | 1 | 1 | 1 |
| serotonin and melatonin biosynthesis | 5 | 5 | 0,081873 | 1,029966 | 0,862712 | 1 | 1 | 1 |
| glucocorticoid biosynthesis | 5 | 5 | 0,081873 | 1,029966 | 0,862712 | 1 | 1 | 1 |
| ceramide biosynthesis | 5 | 5 | 0,081873 | 1,029966 | 0,862712 | 1 | 1 | 1 |
| creatine-phosphate biosynthesis | 5 | 5 | 0,081873 | 1,029966 | 0,862712 | 1 | 1 | 1 |
| salvage pathways of pyrimidine deoxyribonucleotides | 5 | 5 | 0,081873 | 1,029966 | 0,862712 | 1 | 1 | 1 |
| urate biosynthesis/inosine 5'-phosphate degradation | 5 | 5 | 0,081873 | 1,029966 | 0,862712 | 1 | 1 | 1 |
| UDP-N-acetyl-D-glucosamine biosynthesis II | 5 | 5 | 0,081873 | 1,029966 | 0,862712 | 1 | 1 | 1 |
| superpathway of N-acetylglucosamine, N-acetylmannosamine and N-acetylneuraminate degradation | 5 | 5 | 0,081873 | 1,029966 | 0,862712 | 1 | 1 | 1 |
| putrescine degradation III | 5 | 5 | 0,081873 | 1,029966 | 0,862712 | 1 | 1 | 1 |
| aspartate degradation II | 5 | 5 | 0,081873 | 1,029966 | 0,862712 | 1 | 1 | 1 |
| phenylalanine degradation IV (mammalian, via side chain) | 5 | 5 | 0,081873 | 1,029966 | 0,862712 | 1 | 1 | 1 |
| tryptophan degradation to 2-amino-3-carboxymuconate semialdehyde | 5 | 5 | 0,081873 | 1,029966 | 0,862712 | 1 | 1 | 1 |
| tyrosine degradation I | 5 | 5 | 0,081873 | 1,029966 | 0,862712 | 1 | 1 | 1 |
| superpathway of melatonin degradation | 5 | 5 | 0,081873 | 1,029966 | 0,862712 | 1 | 1 | 1 |
| superoxide radicals degradation | 5 | 5 | 0,081873 | 1,029966 | 0,862712 | 1 | 1 | 1 |
| pyruvate fermentation to lactate | 5 | 5 | 0,081873 | 1,029966 | 0,862712 | 1 | 1 | 1 |
| PLK3 signaling events | 5 | 5 | 0,081873 | 1,029966 | 0,862712 | 1 | 1 | 1 |
| Deadenylation of mRNA | 21 | 22 | 0,343868 | 0,98317 | 0,866814 | 1 | 1 | 1 |
| Cell death signalling via NRAGE, NRIF and NADE | 20 | 21 | 0,327493 | 0,980943 | 0,876664 | 1 | 1 | 1 |
| RNA Polymerase I Transcription Termination | 20 | 21 | 0,327493 | 0,980943 | 0,876664 | 1 | 1 | 1 |
| Validated transcriptional targets of deltaNp63 isoforms | 61 | 64 | 0,998854 | 0,981693 | 0,884961 | 1 | 1 | 1 |
| superpathway of D-myo-inositol (1,4,5)-trisphosphate metabolism | 19 | 20 | 0,311118 | 0,978493 | 0,886324 | 1 | 1 | 1 |
| Regulation of Commissural axon pathfinding by Slit and Robo | 4 | 4 | 0,065499 | 1,029966 | 0,88858 | 1 | 1 | 1 |
| Prostacyclin signalling through prostacyclin receptor | 4 | 4 | 0,065499 | 1,029966 | 0,88858 | 1 | 1 | 1 |
| Regulation of Signaling by NODAL | 4 | 4 | 0,065499 | 1,029966 | 0,88858 | 1 | 1 | 1 |
| Reuptake of GABA | 4 | 4 | 0,065499 | 1,029966 | 0,88858 | 1 | 1 | 1 |
| Classical Kir channels | 4 | 4 | 0,065499 | 1,029966 | 0,88858 | 1 | 1 | 1 |
| ATP sensitive Potassium channels | 4 | 4 | 0,065499 | 1,029966 | 0,88858 | 1 | 1 | 1 |
| Activation of PKB | 4 | 4 | 0,065499 | 1,029966 | 0,88858 | 1 | 1 | 1 |
| AKT phosphorylates targets in the nucleus | 4 | 4 | 0,065499 | 1,029966 | 0,88858 | 1 | 1 | 1 |
| betaKlotho-mediated ligand binding | 4 | 4 | 0,065499 | 1,029966 | 0,88858 | 1 | 1 | 1 |
| Phosphorylation of proteins involved in G1/S transition by active Cyclin E:Cdk2 complexes | 4 | 4 | 0,065499 | 1,029966 | 0,88858 | 1 | 1 | 1 |
| mRNA Editing | 4 | 4 | 0,065499 | 1,029966 | 0,88858 | 1 | 1 | 1 |
| Mature Notch receptor traffics to plasma membrane | 4 | 4 | 0,065499 | 1,029966 | 0,88858 | 1 | 1 | 1 |
| Transport of Notch receptor precursor to golgi | 4 | 4 | 0,065499 | 1,029966 | 0,88858 | 1 | 1 | 1 |
| Neurophilin interactions with VEGF and VEGFR | 4 | 4 | 0,065499 | 1,029966 | 0,88858 | 1 | 1 | 1 |
| Histamine receptors | 4 | 4 | 0,065499 | 1,029966 | 0,88858 | 1 | 1 | 1 |
| Adenosine P1 receptors | 4 | 4 | 0,065499 | 1,029966 | 0,88858 | 1 | 1 | 1 |
| Free fatty acid receptors | 4 | 4 | 0,065499 | 1,029966 | 0,88858 | 1 | 1 | 1 |
| Pregnenolone biosynthesis | 4 | 4 | 0,065499 | 1,029966 | 0,88858 | 1 | 1 | 1 |
| Alpha-oxidation of phytanate | 4 | 4 | 0,065499 | 1,029966 | 0,88858 | 1 | 1 | 1 |
| Synthesis of Ketone Bodies | 4 | 4 | 0,065499 | 1,029966 | 0,88858 | 1 | 1 | 1 |
| Plasmalogen biosynthesis | 4 | 4 | 0,065499 | 1,029966 | 0,88858 | 1 | 1 | 1 |
| Translesion synthesis by DNA polymerases bypassing lesion on DNA template | 4 | 4 | 0,065499 | 1,029966 | 0,88858 | 1 | 1 | 1 |
| DNA Damage Bypass | 4 | 4 | 0,065499 | 1,029966 | 0,88858 | 1 | 1 | 1 |
| NOSTRIN mediated eNOS trafficking | 4 | 4 | 0,065499 | 1,029966 | 0,88858 | 1 | 1 | 1 |
| G2/M DNA replication checkpoint | 4 | 4 | 0,065499 | 1,029966 | 0,88858 | 1 | 1 | 1 |
| Gap junction assembly | 4 | 4 | 0,065499 | 1,029966 | 0,88858 | 1 | 1 | 1 |
| Amine Oxidase reactions | 4 | 4 | 0,065499 | 1,029966 | 0,88858 | 1 | 1 | 1 |
| Ethanol oxidation | 4 | 4 | 0,065499 | 1,029966 | 0,88858 | 1 | 1 | 1 |
| Catecholamine biosynthesis | 4 | 4 | 0,065499 | 1,029966 | 0,88858 | 1 | 1 | 1 |
| Carnitine synthesis | 4 | 4 | 0,065499 | 1,029966 | 0,88858 | 1 | 1 | 1 |
| Histidine catabolism | 4 | 4 | 0,065499 | 1,029966 | 0,88858 | 1 | 1 | 1 |
| Vitamin B2 (riboflavin) metabolism | 4 | 4 | 0,065499 | 1,029966 | 0,88858 | 1 | 1 | 1 |
| Vitamin B1 (thiamin) metabolism | 4 | 4 | 0,065499 | 1,029966 | 0,88858 | 1 | 1 | 1 |
| LDL-mediated lipid transport | 4 | 4 | 0,065499 | 1,029966 | 0,88858 | 1 | 1 | 1 |
| Localization of the PINCH-ILK-PARVIN complex to focal adhesions | 4 | 4 | 0,065499 | 1,029966 | 0,88858 | 1 | 1 | 1 |
| Proton-coupled monocarboxylate transport | 4 | 4 | 0,065499 | 1,029966 | 0,88858 | 1 | 1 | 1 |
| Proton/oligonucleotide cotransporters | 4 | 4 | 0,065499 | 1,029966 | 0,88858 | 1 | 1 | 1 |
| Class II GLUTs | 4 | 4 | 0,065499 | 1,029966 | 0,88858 | 1 | 1 | 1 |
| Transcription from mitochondrial promoters | 4 | 4 | 0,065499 | 1,029966 | 0,88858 | 1 | 1 | 1 |
| Breakdown of the nuclear lamina | 4 | 4 | 0,065499 | 1,029966 | 0,88858 | 1 | 1 | 1 |
| Activation of NOXA and translocation to mitochondria | 4 | 4 | 0,065499 | 1,029966 | 0,88858 | 1 | 1 | 1 |
| Activation of PUMA and translocation to mitochondria | 4 | 4 | 0,065499 | 1,029966 | 0,88858 | 1 | 1 | 1 |
| superpathway of serine and glycine biosynthesis I | 4 | 4 | 0,065499 | 1,029966 | 0,88858 | 1 | 1 | 1 |
| arginine degradation I (arginase pathway) | 4 | 4 | 0,065499 | 1,029966 | 0,88858 | 1 | 1 | 1 |
| GDP-glucose biosynthesis | 4 | 4 | 0,065499 | 1,029966 | 0,88858 | 1 | 1 | 1 |
| CMP-N-acetylneuraminate biosynthesis I (eukaryotes) | 4 | 4 | 0,065499 | 1,029966 | 0,88858 | 1 | 1 | 1 |
| dermatan sulfate biosynthesis (late stages) | 4 | 4 | 0,065499 | 1,029966 | 0,88858 | 1 | 1 | 1 |
| NAD phosphorylation and dephosphorylation | 4 | 4 | 0,065499 | 1,029966 | 0,88858 | 1 | 1 | 1 |
| NAD biosynthesis III | 4 | 4 | 0,065499 | 1,029966 | 0,88858 | 1 | 1 | 1 |
| trans,trans-farnesyl diphosphate biosynthesis | 4 | 4 | 0,065499 | 1,029966 | 0,88858 | 1 | 1 | 1 |
| heme biosynthesis from uroporphyrinogen-III I | 4 | 4 | 0,065499 | 1,029966 | 0,88858 | 1 | 1 | 1 |
| tetrahydrofolate salvage from 5,10-methenyltetrahydrofolate | 4 | 4 | 0,065499 | 1,029966 | 0,88858 | 1 | 1 | 1 |
| catecholamine biosynthesis | 4 | 4 | 0,065499 | 1,029966 | 0,88858 | 1 | 1 | 1 |
| estrogen biosynthesis | 4 | 4 | 0,065499 | 1,029966 | 0,88858 | 1 | 1 | 1 |
| mineralocorticoid biosynthesis | 4 | 4 | 0,065499 | 1,029966 | 0,88858 | 1 | 1 | 1 |
| Rapoport-Luebering glycolytic shunt | 4 | 4 | 0,065499 | 1,029966 | 0,88858 | 1 | 1 | 1 |
| adenine and adenosine salvage III | 4 | 4 | 0,065499 | 1,029966 | 0,88858 | 1 | 1 | 1 |
| N-acetylglucosamine degradation II | 4 | 4 | 0,065499 | 1,029966 | 0,88858 | 1 | 1 | 1 |
| spermine and spermidine degradation I | 4 | 4 | 0,065499 | 1,029966 | 0,88858 | 1 | 1 | 1 |
| glycerol degradation IV | 4 | 4 | 0,065499 | 1,029966 | 0,88858 | 1 | 1 | 1 |
| branched-chain alpha-keto acid dehydrogenase complex | 4 | 4 | 0,065499 | 1,029966 | 0,88858 | 1 | 1 | 1 |
| 2-ketoglutarate dehydrogenase complex | 4 | 4 | 0,065499 | 1,029966 | 0,88858 | 1 | 1 | 1 |
| glutamate degradation III (via 4-aminobutyrate) | 4 | 4 | 0,065499 | 1,029966 | 0,88858 | 1 | 1 | 1 |
| phenylalanine degradation I (aerobic) | 4 | 4 | 0,065499 | 1,029966 | 0,88858 | 1 | 1 | 1 |
| heme degradation | 4 | 4 | 0,065499 | 1,029966 | 0,88858 | 1 | 1 | 1 |
| glucose and glucose-1-phosphate degradation | 4 | 4 | 0,065499 | 1,029966 | 0,88858 | 1 | 1 | 1 |
| galactose degradation I (Leloir pathway) | 4 | 4 | 0,065499 | 1,029966 | 0,88858 | 1 | 1 | 1 |
| bupropion degradation | 4 | 4 | 0,065499 | 1,029966 | 0,88858 | 1 | 1 | 1 |
| pentose phosphate pathway (oxidative branch) | 4 | 4 | 0,065499 | 1,029966 | 0,88858 | 1 | 1 | 1 |
| Generation of second messenger molecules | 18 | 19 | 0,294744 | 0,975785 | 0,895773 | 1 | 1 | 1 |
| glutathione-mediated detoxification | 18 | 19 | 0,294744 | 0,975785 | 0,895773 | 1 | 1 | 1 |
| Transcription of the HIV genome | 57 | 60 | 0,933355 | 0,978476 | 0,903704 | 1 | 1 | 1 |
| RNA Polymerase II Pre-transcription Events | 55 | 58 | 0,900606 | 0,976701 | 0,912486 | 1 | 1 | 1 |
| superpathway of methionine degradation | 16 | 17 | 0,261994 | 0,969415 | 0,913956 | 1 | 1 | 1 |
| Beta5 beta6 beta7 and beta8 integrin cell surface interactions | 16 | 17 | 0,261994 | 0,969415 | 0,913956 | 1 | 1 | 1 |
| Transport of Mature mRNAs Derived from Intronless Transcripts | 34 | 36 | 0,556738 | 0,972761 | 0,91406 | 1 | 1 | 1 |
| AKT-mediated inactivation of FOXO1A | 3 | 3 | 0,049124 | 1,029966 | 0,91522 | 1 | 1 | 1 |
| Arachidonate production from DAG | 3 | 3 | 0,049124 | 1,029966 | 0,91522 | 1 | 1 | 1 |
| Adrenaline signalling through Alpha-2 adrenergic receptor | 3 | 3 | 0,049124 | 1,029966 | 0,91522 | 1 | 1 | 1 |
| ERK1 activation | 3 | 3 | 0,049124 | 1,029966 | 0,91522 | 1 | 1 | 1 |
| Dopamine clearance from the synaptic cleft | 3 | 3 | 0,049124 | 1,029966 | 0,91522 | 1 | 1 | 1 |
| TWIK related potassium channel (TREK) | 3 | 3 | 0,049124 | 1,029966 | 0,91522 | 1 | 1 | 1 |
| GABA A (rho) receptor activation | 3 | 3 | 0,049124 | 1,029966 | 0,91522 | 1 | 1 | 1 |
| Vpr-mediated induction of apoptosis by mitochondrial outer membrane permeabilization | 3 | 3 | 0,049124 | 1,029966 | 0,91522 | 1 | 1 | 1 |
| Autointegration results in viral DNA circles | 3 | 3 | 0,049124 | 1,029966 | 0,91522 | 1 | 1 | 1 |
| Proteolytic cleavage of SNARE complex proteins | 3 | 3 | 0,049124 | 1,029966 | 0,91522 | 1 | 1 | 1 |
| Signalling to STAT3 | 3 | 3 | 0,049124 | 1,029966 | 0,91522 | 1 | 1 | 1 |
| NFG and proNGF binds to p75NTR | 3 | 3 | 0,049124 | 1,029966 | 0,91522 | 1 | 1 | 1 |
| PDE3B signalling | 3 | 3 | 0,049124 | 1,029966 | 0,91522 | 1 | 1 | 1 |
| Inhibition of TSC complex formation by PKB | 3 | 3 | 0,049124 | 1,029966 | 0,91522 | 1 | 1 | 1 |
| Ceramide signalling | 3 | 3 | 0,049124 | 1,029966 | 0,91522 | 1 | 1 | 1 |
| p75NTR negatively regulates cell cycle via SC1 | 3 | 3 | 0,049124 | 1,029966 | 0,91522 | 1 | 1 | 1 |
| SHC activation | 3 | 3 | 0,049124 | 1,029966 | 0,91522 | 1 | 1 | 1 |
| FGFR1c and Klotho ligand binding and activation | 3 | 3 | 0,049124 | 1,029966 | 0,91522 | 1 | 1 | 1 |
| Cam-PDE 1 activation | 3 | 3 | 0,049124 | 1,029966 | 0,91522 | 1 | 1 | 1 |
| G2 Phase | 3 | 3 | 0,049124 | 1,029966 | 0,91522 | 1 | 1 | 1 |
| Phosphorylation of proteins involved in the G2/M transition by Cyclin A:Cdc2 complexes | 3 | 3 | 0,049124 | 1,029966 | 0,91522 | 1 | 1 | 1 |
| Binding of RNA by Insulin-like Growth Factor-2 mRNA Binding Proteins (IGF2BPs/IMPs/VICKZs) | 3 | 3 | 0,049124 | 1,029966 | 0,91522 | 1 | 1 | 1 |
| VEGF binds to VEGFR leading to receptor dimerization | 3 | 3 | 0,049124 | 1,029966 | 0,91522 | 1 | 1 | 1 |
| ERKs are inactivated | 3 | 3 | 0,049124 | 1,029966 | 0,91522 | 1 | 1 | 1 |
| Utilization of Ketone Bodies | 3 | 3 | 0,049124 | 1,029966 | 0,91522 | 1 | 1 | 1 |
| Processing of DNA ends prior to end rejoining | 3 | 3 | 0,049124 | 1,029966 | 0,91522 | 1 | 1 | 1 |
| Processing of DNA double-strand break ends | 3 | 3 | 0,049124 | 1,029966 | 0,91522 | 1 | 1 | 1 |
| Depurination | 3 | 3 | 0,049124 | 1,029966 | 0,91522 | 1 | 1 | 1 |
| Cleavage of the damaged purine | 3 | 3 | 0,049124 | 1,029966 | 0,91522 | 1 | 1 | 1 |
| Recognition and association of DNA glycosylase with site containing an affected purine | 3 | 3 | 0,049124 | 1,029966 | 0,91522 | 1 | 1 | 1 |
| Synthesis of dolichyl-phosphate mannose | 3 | 3 | 0,049124 | 1,029966 | 0,91522 | 1 | 1 | 1 |
| Progressive trimming of alpha-1,2-linked mannose residues from Man9/8/7GlcNAc2 to produce Man5GlcNAc2 | 3 | 3 | 0,049124 | 1,029966 | 0,91522 | 1 | 1 | 1 |
| NS1 Mediated Effects on Host Pathways | 3 | 3 | 0,049124 | 1,029966 | 0,91522 | 1 | 1 | 1 |
| Inhibition of HSL | 3 | 3 | 0,049124 | 1,029966 | 0,91522 | 1 | 1 | 1 |
| Oligomerization of connexins into connexons | 3 | 3 | 0,049124 | 1,029966 | 0,91522 | 1 | 1 | 1 |
| Fatty acids | 3 | 3 | 0,049124 | 1,029966 | 0,91522 | 1 | 1 | 1 |
| FMO oxidizes nucleophiles | 3 | 3 | 0,049124 | 1,029966 | 0,91522 | 1 | 1 | 1 |
| Formation of the active cofactor, UDP-glucuronate | 3 | 3 | 0,049124 | 1,029966 | 0,91522 | 1 | 1 | 1 |
| Conjugation of carboxylic acids | 3 | 3 | 0,049124 | 1,029966 | 0,91522 | 1 | 1 | 1 |
| Amino Acid conjugation | 3 | 3 | 0,049124 | 1,029966 | 0,91522 | 1 | 1 | 1 |
| Conjugation of benzoate with glycine | 3 | 3 | 0,049124 | 1,029966 | 0,91522 | 1 | 1 | 1 |
| Interconversion of polyamines | 3 | 3 | 0,049124 | 1,029966 | 0,91522 | 1 | 1 | 1 |
| Creatine metabolism | 3 | 3 | 0,049124 | 1,029966 | 0,91522 | 1 | 1 | 1 |
| Heme degradation | 3 | 3 | 0,049124 | 1,029966 | 0,91522 | 1 | 1 | 1 |
| Regulation of Insulin Secretion by Acetylcholine | 3 | 3 | 0,049124 | 1,029966 | 0,91522 | 1 | 1 | 1 |
| Interconversion of 2-oxoglutarate and 2-hydroxyglutarate | 3 | 3 | 0,049124 | 1,029966 | 0,91522 | 1 | 1 | 1 |
| Beta oxidation of butanoyl-CoA to acetyl-CoA | 3 | 3 | 0,049124 | 1,029966 | 0,91522 | 1 | 1 | 1 |
| Beta oxidation of myristoyl-CoA to lauroyl-CoA | 3 | 3 | 0,049124 | 1,029966 | 0,91522 | 1 | 1 | 1 |
| Beta oxidation of palmitoyl-CoA to myristoyl-CoA | 3 | 3 | 0,049124 | 1,029966 | 0,91522 | 1 | 1 | 1 |
| Trafficking of dietary sterols | 3 | 3 | 0,049124 | 1,029966 | 0,91522 | 1 | 1 | 1 |
| Galactose catabolism | 3 | 3 | 0,049124 | 1,029966 | 0,91522 | 1 | 1 | 1 |
| Na+-dependent glucose transporters | 3 | 3 | 0,049124 | 1,029966 | 0,91522 | 1 | 1 | 1 |
| Rhesus glycoproteins mediate ammonium transport. | 3 | 3 | 0,049124 | 1,029966 | 0,91522 | 1 | 1 | 1 |
| Type II Na+/Pi cotransporters | 3 | 3 | 0,049124 | 1,029966 | 0,91522 | 1 | 1 | 1 |
| Transport of fatty acids | 3 | 3 | 0,049124 | 1,029966 | 0,91522 | 1 | 1 | 1 |
| Stimulation of the cell death response by PAK-2p34 | 3 | 3 | 0,049124 | 1,029966 | 0,91522 | 1 | 1 | 1 |
| Mitochondrial transcription initiation | 3 | 3 | 0,049124 | 1,029966 | 0,91522 | 1 | 1 | 1 |
| BH3-only proteins associate with and inactivate anti-apoptotic BCL-2 members | 3 | 3 | 0,049124 | 1,029966 | 0,91522 | 1 | 1 | 1 |
| Activation of BMF and translocation to mitochondria | 3 | 3 | 0,049124 | 1,029966 | 0,91522 | 1 | 1 | 1 |
| Formation of apoptosome | 3 | 3 | 0,049124 | 1,029966 | 0,91522 | 1 | 1 | 1 |
| Lectin pathway of complement activation | 3 | 3 | 0,049124 | 1,029966 | 0,91522 | 1 | 1 | 1 |
| Activation of C3 and C5 | 3 | 3 | 0,049124 | 1,029966 | 0,91522 | 1 | 1 | 1 |
| Alternative complement activation | 3 | 3 | 0,049124 | 1,029966 | 0,91522 | 1 | 1 | 1 |
| The AIM2 inflammasome | 3 | 3 | 0,049124 | 1,029966 | 0,91522 | 1 | 1 | 1 |
| methionine salvage II (mammalia) | 3 | 3 | 0,049124 | 1,029966 | 0,91522 | 1 | 1 | 1 |
| aspartate biosynthesis | 3 | 3 | 0,049124 | 1,029966 | 0,91522 | 1 | 1 | 1 |
| glutamate degradation II | 3 | 3 | 0,049124 | 1,029966 | 0,91522 | 1 | 1 | 1 |
| uracil degradation II (reductive) | 3 | 3 | 0,049124 | 1,029966 | 0,91522 | 1 | 1 | 1 |
| S-adenosyl-L-methionine biosynthesis | 3 | 3 | 0,049124 | 1,029966 | 0,91522 | 1 | 1 | 1 |
| ubiquinone-10 biosynthesis (eukaryotic) | 3 | 3 | 0,049124 | 1,029966 | 0,91522 | 1 | 1 | 1 |
| tetrahydrobiopterin biosynthesis I | 3 | 3 | 0,049124 | 1,029966 | 0,91522 | 1 | 1 | 1 |
| tetrahydrobiopterin biosynthesis II | 3 | 3 | 0,049124 | 1,029966 | 0,91522 | 1 | 1 | 1 |
| acyl-CoA hydrolysis | 3 | 3 | 0,049124 | 1,029966 | 0,91522 | 1 | 1 | 1 |
| biotin-carboxyl carrier protein assembly | 3 | 3 | 0,049124 | 1,029966 | 0,91522 | 1 | 1 | 1 |
| L-carnitine biosynthesis | 3 | 3 | 0,049124 | 1,029966 | 0,91522 | 1 | 1 | 1 |
| 5-aminoimidazole ribonucleotide biosynthesis I | 3 | 3 | 0,049124 | 1,029966 | 0,91522 | 1 | 1 | 1 |
| inosine-5'-phosphate biosynthesis II | 3 | 3 | 0,049124 | 1,029966 | 0,91522 | 1 | 1 | 1 |
| PRPP biosynthesis I | 3 | 3 | 0,049124 | 1,029966 | 0,91522 | 1 | 1 | 1 |
| D-myo-inositol (1,4,5,6)-tetrakisphosphate biosynthesis | 3 | 3 | 0,049124 | 1,029966 | 0,91522 | 1 | 1 | 1 |
| 1D-myo-inositol hexakisphosphate biosynthesis V (from Ins(1,3,4)P3) | 3 | 3 | 0,049124 | 1,029966 | 0,91522 | 1 | 1 | 1 |
| N-acetylglucosamine degradation I | 3 | 3 | 0,049124 | 1,029966 | 0,91522 | 1 | 1 | 1 |
| methylglyoxal degradation III | 3 | 3 | 0,049124 | 1,029966 | 0,91522 | 1 | 1 | 1 |
| lysine degradation II | 3 | 3 | 0,049124 | 1,029966 | 0,91522 | 1 | 1 | 1 |
| lysine degradation V | 3 | 3 | 0,049124 | 1,029966 | 0,91522 | 1 | 1 | 1 |
| glutaryl-CoA degradation | 3 | 3 | 0,049124 | 1,029966 | 0,91522 | 1 | 1 | 1 |
| acetate conversion to acetyl-CoA | 3 | 3 | 0,049124 | 1,029966 | 0,91522 | 1 | 1 | 1 |
| melatonin degradation I | 3 | 3 | 0,049124 | 1,029966 | 0,91522 | 1 | 1 | 1 |
| thyroid hormone metabolism I (via deiodination) | 3 | 3 | 0,049124 | 1,029966 | 0,91522 | 1 | 1 | 1 |
| thyroid hormone metabolism II (via conjugation and/or degradation) | 3 | 3 | 0,049124 | 1,029966 | 0,91522 | 1 | 1 | 1 |
| purine ribonucleosides degradation to ribose-1-phosphate | 3 | 3 | 0,049124 | 1,029966 | 0,91522 | 1 | 1 | 1 |
| guanosine nucleotides degradation III | 3 | 3 | 0,049124 | 1,029966 | 0,91522 | 1 | 1 | 1 |
| thymine degradation | 3 | 3 | 0,049124 | 1,029966 | 0,91522 | 1 | 1 | 1 |
| Class IB PI3K non-lipid kinase events | 3 | 3 | 0,049124 | 1,029966 | 0,91522 | 1 | 1 | 1 |
| Ephrin A reverse signaling | 3 | 3 | 0,049124 | 1,029966 | 0,91522 | 1 | 1 | 1 |
| AlphaE beta7 integrin cell surface interactions | 3 | 3 | 0,049124 | 1,029966 | 0,91522 | 1 | 1 | 1 |
| Hypoxic and oxygen homeostasis regulation of HIF-1-alpha | 76 | 80 | 1,244474 | 0,978474 | 0,917244 | 1 | 1 | 1 |
| Transport of Mature mRNA Derived from an Intronless Transcript | 33 | 35 | 0,540364 | 0,971127 | 0,919575 | 1 | 1 | 1 |
| HIV Life Cycle | 98 | 103 | 1,604716 | 0,979972 | 0,921066 | 1 | 1 | 1 |
| Platelet calcium homeostasis | 15 | 16 | 0,24562 | 0,965633 | 0,922644 | 1 | 1 | 1 |
| Circadian rhythm pathway | 15 | 16 | 0,24562 | 0,965633 | 0,922644 | 1 | 1 | 1 |
| Interactions of Vpr with host cellular proteins | 32 | 34 | 0,523989 | 0,969397 | 0,924929 | 1 | 1 | 1 |
| HIF-2-alpha transcription factor network | 32 | 34 | 0,523989 | 0,969397 | 0,924929 | 1 | 1 | 1 |
| Interactions of Rev with host cellular proteins | 31 | 33 | 0,507614 | 0,967562 | 0,930118 | 1 | 1 | 1 |
| Transport of the SLBP Dependant Mature mRNA | 31 | 33 | 0,507614 | 0,967562 | 0,930118 | 1 | 1 | 1 |
| Peptide hormone biosynthesis | 14 | 15 | 0,229245 | 0,961347 | 0,931031 | 1 | 1 | 1 |
| Mitotic Prometaphase | 94 | 99 | 1,539217 | 0,977952 | 0,932122 | 1 | 1 | 1 |
| Rev-mediated nuclear export of HIV-1 RNA | 30 | 32 | 0,49124 | 0,965613 | 0,935137 | 1 | 1 | 1 |
| Transport of the SLBP independent Mature mRNA | 30 | 32 | 0,49124 | 0,965613 | 0,935137 | 1 | 1 | 1 |
| Mitochondrial Fatty Acid Beta-Oxidation | 13 | 14 | 0,21287 | 0,956449 | 0,939092 | 1 | 1 | 1 |
| triacylglycerol degradation | 13 | 14 | 0,21287 | 0,956449 | 0,939092 | 1 | 1 | 1 |
| Vpr-mediated nuclear import of PICs | 29 | 31 | 0,474865 | 0,963538 | 0,939981 | 1 | 1 | 1 |
| Export of Viral Ribonucleoproteins from Nucleus | 29 | 31 | 0,474865 | 0,963538 | 0,939981 | 1 | 1 | 1 |
| Interactions of Tat with host cellular proteins | 2 | 2 | 0,032749 | 1,029966 | 0,942654 | 1 | 1 | 1 |
| Nef mediated downregulation of CD28 cell surface expression | 2 | 2 | 0,032749 | 1,029966 | 0,942654 | 1 | 1 | 1 |
| Amyloids | 2 | 2 | 0,032749 | 1,029966 | 0,942654 | 1 | 1 | 1 |
| ERK2 activation | 2 | 2 | 0,032749 | 1,029966 | 0,942654 | 1 | 1 | 1 |
| Serotonin clearance from the synaptic cleft | 2 | 2 | 0,032749 | 1,029966 | 0,942654 | 1 | 1 | 1 |
| Enzymatic degradation of Dopamine by monoamine oxidase | 2 | 2 | 0,032749 | 1,029966 | 0,942654 | 1 | 1 | 1 |
| Enzymatic degradation of dopamine by COMT | 2 | 2 | 0,032749 | 1,029966 | 0,942654 | 1 | 1 | 1 |
| Metabolism of serotonin | 2 | 2 | 0,032749 | 1,029966 | 0,942654 | 1 | 1 | 1 |
| Neurotransmitter uptake and Metabolism In Glial Cells | 2 | 2 | 0,032749 | 1,029966 | 0,942654 | 1 | 1 | 1 |
| Astrocytic Glutamate-Glutamine Uptake And Metabolism | 2 | 2 | 0,032749 | 1,029966 | 0,942654 | 1 | 1 | 1 |
| Amplification of signal from unattached kinetochores via a MAD2 inhibitory signal | 2 | 2 | 0,032749 | 1,029966 | 0,942654 | 1 | 1 | 1 |
| Degradation of GABA | 2 | 2 | 0,032749 | 1,029966 | 0,942654 | 1 | 1 | 1 |
| Activation of Na-permeable Kainate Receptors | 2 | 2 | 0,032749 | 1,029966 | 0,942654 | 1 | 1 | 1 |
| Ionotropic activity of Kainate Receptors | 2 | 2 | 0,032749 | 1,029966 | 0,942654 | 1 | 1 | 1 |
| Binding and entry of HIV virion | 2 | 2 | 0,032749 | 1,029966 | 0,942654 | 1 | 1 | 1 |
| Translocation of BoNT Light chain | 2 | 2 | 0,032749 | 1,029966 | 0,942654 | 1 | 1 | 1 |
| Signalling to ERK5 | 2 | 2 | 0,032749 | 1,029966 | 0,942654 | 1 | 1 | 1 |
| TRKA activation by NGF | 2 | 2 | 0,032749 | 1,029966 | 0,942654 | 1 | 1 | 1 |
| FGFR2c ligand binding and activation | 2 | 2 | 0,032749 | 1,029966 | 0,942654 | 1 | 1 | 1 |
| FGFR3 ligand binding and activation | 2 | 2 | 0,032749 | 1,029966 | 0,942654 | 1 | 1 | 1 |
| FGFR3b ligand binding and activation | 2 | 2 | 0,032749 | 1,029966 | 0,942654 | 1 | 1 | 1 |
| FGFR3c ligand binding and activation | 2 | 2 | 0,032749 | 1,029966 | 0,942654 | 1 | 1 | 1 |
| FGFR4 ligand binding and activation | 2 | 2 | 0,032749 | 1,029966 | 0,942654 | 1 | 1 | 1 |
| FGFR1c ligand binding and activation | 2 | 2 | 0,032749 | 1,029966 | 0,942654 | 1 | 1 | 1 |
| Pyrophosphate hydrolysis | 2 | 2 | 0,032749 | 1,029966 | 0,942654 | 1 | 1 | 1 |
| Formation of the Editosome | 2 | 2 | 0,032749 | 1,029966 | 0,942654 | 1 | 1 | 1 |
| mRNA Editing: C to U Conversion | 2 | 2 | 0,032749 | 1,029966 | 0,942654 | 1 | 1 | 1 |
| C6 deamination of adenosine | 2 | 2 | 0,032749 | 1,029966 | 0,942654 | 1 | 1 | 1 |
| Formation of editosomes by ADAR proteins | 2 | 2 | 0,032749 | 1,029966 | 0,942654 | 1 | 1 | 1 |
| mRNA Editing: A to I Conversion | 2 | 2 | 0,032749 | 1,029966 | 0,942654 | 1 | 1 | 1 |
| phospho-PLA2 pathway | 2 | 2 | 0,032749 | 1,029966 | 0,942654 | 1 | 1 | 1 |
| Estrogen biosynthesis | 2 | 2 | 0,032749 | 1,029966 | 0,942654 | 1 | 1 | 1 |
| DNA Damage Recognition in GG-NER | 2 | 2 | 0,032749 | 1,029966 | 0,942654 | 1 | 1 | 1 |
| DNA Damage Reversal | 2 | 2 | 0,032749 | 1,029966 | 0,942654 | 1 | 1 | 1 |
| Reversal of Alkylation Damage By DNA Dioxygenases | 2 | 2 | 0,032749 | 1,029966 | 0,942654 | 1 | 1 | 1 |
| Translesion synthesis by Pol zeta | 2 | 2 | 0,032749 | 1,029966 | 0,942654 | 1 | 1 | 1 |
| NOSIP mediated eNOS trafficking | 2 | 2 | 0,032749 | 1,029966 | 0,942654 | 1 | 1 | 1 |
| Reactions specific to the complex N-glycan synthesis pathway | 2 | 2 | 0,032749 | 1,029966 | 0,942654 | 1 | 1 | 1 |
| Transcriptional activation of p53 responsive genes | 2 | 2 | 0,032749 | 1,029966 | 0,942654 | 1 | 1 | 1 |
| Transcriptional activation of cell cycle inhibitor p21 | 2 | 2 | 0,032749 | 1,029966 | 0,942654 | 1 | 1 | 1 |
| Amplification of signal from the kinetochores | 2 | 2 | 0,032749 | 1,029966 | 0,942654 | 1 | 1 | 1 |
| Synthesis of Dolichyl-phosphate | 2 | 2 | 0,032749 | 1,029966 | 0,942654 | 1 | 1 | 1 |
| Hypusine synthesis from eIF5A-lysine | 2 | 2 | 0,032749 | 1,029966 | 0,942654 | 1 | 1 | 1 |
| Influenza Virus Induced Apoptosis | 2 | 2 | 0,032749 | 1,029966 | 0,942654 | 1 | 1 | 1 |
| Inhibition of Host mRNA Processing and RNA Silencing | 2 | 2 | 0,032749 | 1,029966 | 0,942654 | 1 | 1 | 1 |
| Assembly of Viral Components at the Budding Site | 2 | 2 | 0,032749 | 1,029966 | 0,942654 | 1 | 1 | 1 |
| Viral RNP Complexes in the Host Cell Nucleus | 2 | 2 | 0,032749 | 1,029966 | 0,942654 | 1 | 1 | 1 |
| Virus Assembly and Release | 2 | 2 | 0,032749 | 1,029966 | 0,942654 | 1 | 1 | 1 |
| vRNP Assembly | 2 | 2 | 0,032749 | 1,029966 | 0,942654 | 1 | 1 | 1 |
| Entry of Influenza Virion into Host Cell via Endocytosis | 2 | 2 | 0,032749 | 1,029966 | 0,942654 | 1 | 1 | 1 |
| Regulation of gap junction activity | 2 | 2 | 0,032749 | 1,029966 | 0,942654 | 1 | 1 | 1 |
| c-src mediated regulation of Cx43 function and closure of gap junctions | 2 | 2 | 0,032749 | 1,029966 | 0,942654 | 1 | 1 | 1 |
| Transport of connexons to the plasma membrane | 2 | 2 | 0,032749 | 1,029966 | 0,942654 | 1 | 1 | 1 |
| Monoamines are oxidized to aldehydes by MAOA and MAOB, producing NH3 and H2O2 | 2 | 2 | 0,032749 | 1,029966 | 0,942654 | 1 | 1 | 1 |
| Acetylation | 2 | 2 | 0,032749 | 1,029966 | 0,942654 | 1 | 1 | 1 |
| Polyamines are oxidized to amines, aldehydes and H2O2 by PAOs | 2 | 2 | 0,032749 | 1,029966 | 0,942654 | 1 | 1 | 1 |
| Conjugation of salicylate with glycine | 2 | 2 | 0,032749 | 1,029966 | 0,942654 | 1 | 1 | 1 |
| Conjugation of phenylacetate with glutamine | 2 | 2 | 0,032749 | 1,029966 | 0,942654 | 1 | 1 | 1 |
| Inhibition of Insulin Secretion by Adrenaline/Noradrenaline | 2 | 2 | 0,032749 | 1,029966 | 0,942654 | 1 | 1 | 1 |
| Agmatine biosynthesis | 2 | 2 | 0,032749 | 1,029966 | 0,942654 | 1 | 1 | 1 |
| Proline catabolism | 2 | 2 | 0,032749 | 1,029966 | 0,942654 | 1 | 1 | 1 |
| PP2A-mediated dephosphorylation of key metabolic factors | 2 | 2 | 0,032749 | 1,029966 | 0,942654 | 1 | 1 | 1 |
| Insulin effects increased synthesis of Xylulose-5-Phosphate | 2 | 2 | 0,032749 | 1,029966 | 0,942654 | 1 | 1 | 1 |
| Nicotinamide salvaging | 2 | 2 | 0,032749 | 1,029966 | 0,942654 | 1 | 1 | 1 |
| Vitamins B6 activation to pyridoxal phosphate | 2 | 2 | 0,032749 | 1,029966 | 0,942654 | 1 | 1 | 1 |
| Glycogen synthesis | 2 | 2 | 0,032749 | 1,029966 | 0,942654 | 1 | 1 | 1 |
| Inositol transporters | 2 | 2 | 0,032749 | 1,029966 | 0,942654 | 1 | 1 | 1 |
| Proton-coupled neutral amino acid transporters | 2 | 2 | 0,032749 | 1,029966 | 0,942654 | 1 | 1 | 1 |
| Transport of Glycerol from Adipocytes to the Liver by Aquaporins | 2 | 2 | 0,032749 | 1,029966 | 0,942654 | 1 | 1 | 1 |
| Regulation of PAK-2p34 activity by PS-GAP/RHG10 | 2 | 2 | 0,032749 | 1,029966 | 0,942654 | 1 | 1 | 1 |
| Release of apoptotic factors from the mitochondria | 2 | 2 | 0,032749 | 1,029966 | 0,942654 | 1 | 1 | 1 |
| Activation and oligomerization of BAK protein | 2 | 2 | 0,032749 | 1,029966 | 0,942654 | 1 | 1 | 1 |
| Activation, translocation and oligomerization of BAX | 2 | 2 | 0,032749 | 1,029966 | 0,942654 | 1 | 1 | 1 |
| LPS transferred from LBP carrier to CD14 | 2 | 2 | 0,032749 | 1,029966 | 0,942654 | 1 | 1 | 1 |
| The IPAF inflammasome | 2 | 2 | 0,032749 | 1,029966 | 0,942654 | 1 | 1 | 1 |
| sulfate activation for sulfonation | 2 | 2 | 0,032749 | 1,029966 | 0,942654 | 1 | 1 | 1 |
| hypusine biosynthesis | 2 | 2 | 0,032749 | 1,029966 | 0,942654 | 1 | 1 | 1 |
| alanine biosynthesis II | 2 | 2 | 0,032749 | 1,029966 | 0,942654 | 1 | 1 | 1 |
| taurine biosynthesis | 2 | 2 | 0,032749 | 1,029966 | 0,942654 | 1 | 1 | 1 |
| serine biosynthesis | 2 | 2 | 0,032749 | 1,029966 | 0,942654 | 1 | 1 | 1 |
| beta-alanine biosynthesis IV | 2 | 2 | 0,032749 | 1,029966 | 0,942654 | 1 | 1 | 1 |
| cysteine biosynthesis/homocysteine degradation | 2 | 2 | 0,032749 | 1,029966 | 0,942654 | 1 | 1 | 1 |
| glutamate biosynthesis II | 2 | 2 | 0,032749 | 1,029966 | 0,942654 | 1 | 1 | 1 |
| glycine biosynthesis III | 2 | 2 | 0,032749 | 1,029966 | 0,942654 | 1 | 1 | 1 |
| glycine biosynthesis I | 2 | 2 | 0,032749 | 1,029966 | 0,942654 | 1 | 1 | 1 |
| UDP-D-xylose and UDP-D-glucuronate biosynthesis | 2 | 2 | 0,032749 | 1,029966 | 0,942654 | 1 | 1 | 1 |
| molybdenum cofactor biosynthesis | 2 | 2 | 0,032749 | 1,029966 | 0,942654 | 1 | 1 | 1 |
| lipoate biosynthesis and incorporation II | 2 | 2 | 0,032749 | 1,029966 | 0,942654 | 1 | 1 | 1 |
| geranylgeranyldiphosphate biosynthesis | 2 | 2 | 0,032749 | 1,029966 | 0,942654 | 1 | 1 | 1 |
| glutathione redox reactions II | 2 | 2 | 0,032749 | 1,029966 | 0,942654 | 1 | 1 | 1 |
| glutathione biosynthesis | 2 | 2 | 0,032749 | 1,029966 | 0,942654 | 1 | 1 | 1 |
| thioredoxin pathway | 2 | 2 | 0,032749 | 1,029966 | 0,942654 | 1 | 1 | 1 |
| flavin biosynthesis IV (mammalian) | 2 | 2 | 0,032749 | 1,029966 | 0,942654 | 1 | 1 | 1 |
| pyridoxal 5'-phosphate salvage pathway | 2 | 2 | 0,032749 | 1,029966 | 0,942654 | 1 | 1 | 1 |
| dolichol and dolichyl phosphate biosynthesis | 2 | 2 | 0,032749 | 1,029966 | 0,942654 | 1 | 1 | 1 |
| fatty acid biosynthesis initiation II | 2 | 2 | 0,032749 | 1,029966 | 0,942654 | 1 | 1 | 1 |
| palmitate biosynthesis I (animals) | 2 | 2 | 0,032749 | 1,029966 | 0,942654 | 1 | 1 | 1 |
| uridine-5'-phosphate biosynthesis | 2 | 2 | 0,032749 | 1,029966 | 0,942654 | 1 | 1 | 1 |
| epoxysqualene biosynthesis | 2 | 2 | 0,032749 | 1,029966 | 0,942654 | 1 | 1 | 1 |
| spermine biosynthesis | 2 | 2 | 0,032749 | 1,029966 | 0,942654 | 1 | 1 | 1 |
| glycine degradation (creatine biosynthesis) | 2 | 2 | 0,032749 | 1,029966 | 0,942654 | 1 | 1 | 1 |
| choline degradation I | 2 | 2 | 0,032749 | 1,029966 | 0,942654 | 1 | 1 | 1 |
| spermidine biosynthesis | 2 | 2 | 0,032749 | 1,029966 | 0,942654 | 1 | 1 | 1 |
| D-myo-inositol (3,4,5,6)-tetrakisphosphate biosynthesis | 2 | 2 | 0,032749 | 1,029966 | 0,942654 | 1 | 1 | 1 |
| histamine degradation | 2 | 2 | 0,032749 | 1,029966 | 0,942654 | 1 | 1 | 1 |
| N-acetylneuraminate and N-acetylmannosamine degradation | 2 | 2 | 0,032749 | 1,029966 | 0,942654 | 1 | 1 | 1 |
| 4-aminobutyrate degradation I | 2 | 2 | 0,032749 | 1,029966 | 0,942654 | 1 | 1 | 1 |
| methylglyoxal degradation I | 2 | 2 | 0,032749 | 1,029966 | 0,942654 | 1 | 1 | 1 |
| alanine degradation III | 2 | 2 | 0,032749 | 1,029966 | 0,942654 | 1 | 1 | 1 |
| asparagine degradation I | 2 | 2 | 0,032749 | 1,029966 | 0,942654 | 1 | 1 | 1 |
| beta-alanine degradation I | 2 | 2 | 0,032749 | 1,029966 | 0,942654 | 1 | 1 | 1 |
| glutamate degradation X | 2 | 2 | 0,032749 | 1,029966 | 0,942654 | 1 | 1 | 1 |
| glutamine degradation I | 2 | 2 | 0,032749 | 1,029966 | 0,942654 | 1 | 1 | 1 |
| proline degradation | 2 | 2 | 0,032749 | 1,029966 | 0,942654 | 1 | 1 | 1 |
| L-serine degradation | 2 | 2 | 0,032749 | 1,029966 | 0,942654 | 1 | 1 | 1 |
| formaldehyde oxidation II (glutathione-dependent) | 2 | 2 | 0,032749 | 1,029966 | 0,942654 | 1 | 1 | 1 |
| itaconate degradation | 2 | 2 | 0,032749 | 1,029966 | 0,942654 | 1 | 1 | 1 |
| lactose degradation III | 2 | 2 | 0,032749 | 1,029966 | 0,942654 | 1 | 1 | 1 |
| trehalose degradation II (trehalase) | 2 | 2 | 0,032749 | 1,029966 | 0,942654 | 1 | 1 | 1 |
| mitochondrial L-carnitine shuttle pathway | 2 | 2 | 0,032749 | 1,029966 | 0,942654 | 1 | 1 | 1 |
| ceramide degradation | 2 | 2 | 0,032749 | 1,029966 | 0,942654 | 1 | 1 | 1 |
| anandamide degradation | 2 | 2 | 0,032749 | 1,029966 | 0,942654 | 1 | 1 | 1 |
| melatonin degradation II | 2 | 2 | 0,032749 | 1,029966 | 0,942654 | 1 | 1 | 1 |
| D-glucuronate degradation I | 2 | 2 | 0,032749 | 1,029966 | 0,942654 | 1 | 1 | 1 |
| glutamate dependent acid resistance | 2 | 2 | 0,032749 | 1,029966 | 0,942654 | 1 | 1 | 1 |
| arsenate detoxification I (glutaredoxin) | 2 | 2 | 0,032749 | 1,029966 | 0,942654 | 1 | 1 | 1 |
| glycerol-3-phosphate shuttle | 2 | 2 | 0,032749 | 1,029966 | 0,942654 | 1 | 1 | 1 |
| Glypican 2 network | 2 | 2 | 0,032749 | 1,029966 | 0,942654 | 1 | 1 | 1 |
| PLK2 and PLK4 events | 2 | 2 | 0,032749 | 1,029966 | 0,942654 | 1 | 1 | 1 |
| Nuclear import of Rev protein | 28 | 30 | 0,45849 | 0,961324 | 0,944647 | 1 | 1 | 1 |
| Glycoprotein hormones | 12 | 13 | 0,196496 | 0,950798 | 0,946799 | 1 | 1 | 1 |
| colanic acid building blocks biosynthesis | 12 | 13 | 0,196496 | 0,950798 | 0,946799 | 1 | 1 | 1 |
| D-myo-inositol (1,4,5)-trisphosphate degradation | 12 | 13 | 0,196496 | 0,950798 | 0,946799 | 1 | 1 | 1 |
| NEP/NS2 Interacts with the Cellular Export Machinery | 27 | 29 | 0,442116 | 0,958958 | 0,949131 | 1 | 1 | 1 |
| Transport of Ribonucleoproteins into the Host Nucleus | 27 | 29 | 0,442116 | 0,958958 | 0,949131 | 1 | 1 | 1 |
| Regulation of Glucokinase by Glucokinase Regulatory Protein | 27 | 29 | 0,442116 | 0,958958 | 0,949131 | 1 | 1 | 1 |
| Late Phase of HIV Life Cycle | 87 | 92 | 1,424595 | 0,973995 | 0,949149 | 1 | 1 | 1 |
| Double-Strand Break Repair | 25 | 27 | 0,409366 | 0,9537 | 0,957538 | 1 | 1 | 1 |
| Signal regulatory protein (SIRP) family interactions | 10 | 11 | 0,163747 | 0,936417 | 0,961038 | 1 | 1 | 1 |
| Signaling mediated by p38-gamma and p38-delta | 10 | 11 | 0,163747 | 0,936417 | 0,961038 | 1 | 1 | 1 |
| Formation of HIV-1 elongation complex in the absence of HIV-1 Tat | 39 | 42 | 0,638611 | 0,956414 | 0,967162 | 1 | 1 | 1 |
| Formation of RNA Pol II elongation complex | 39 | 42 | 0,638611 | 0,956414 | 0,967162 | 1 | 1 | 1 |
| RNA Polymerase II Transcription Elongation | 39 | 42 | 0,638611 | 0,956414 | 0,967162 | 1 | 1 | 1 |
| Amino acid synthesis and interconversion (transamination) | 9 | 10 | 0,147372 | 0,927072 | 0,967511 | 1 | 1 | 1 |
| IRAK1 recruits IKK complex upon TLR7/8 or 9 stimulation | 9 | 10 | 0,147372 | 0,927072 | 0,967511 | 1 | 1 | 1 |
| IRAK1 recruits IKK complex | 9 | 10 | 0,147372 | 0,927072 | 0,967511 | 1 | 1 | 1 |
| Metabolism | 791 | 823 | 12,95235 | 0,989919 | 0,96758 | 1 | 1 | 1 |
| HIV-1 Transcription Elongation | 38 | 41 | 0,622237 | 0,954621 | 0,969621 | 1 | 1 | 1 |
| Tat-mediated elongation of the HIV-1 transcript | 38 | 41 | 0,622237 | 0,954621 | 0,969621 | 1 | 1 | 1 |
| Formation of HIV-1 elongation complex containing HIV-1 Tat | 38 | 41 | 0,622237 | 0,954621 | 0,969621 | 1 | 1 | 1 |
| Uncoating of the HIV Virion | 1 | 1 | 0,016375 | 1,029966 | 0,970906 | 1 | 1 | 1 |
| Reverse Transcription of HIV RNA | 1 | 1 | 0,016375 | 1,029966 | 0,970906 | 1 | 1 | 1 |
| Minus-strand DNA synthesis | 1 | 1 | 0,016375 | 1,029966 | 0,970906 | 1 | 1 | 1 |
| Plus-strand DNA synthesis | 1 | 1 | 0,016375 | 1,029966 | 0,970906 | 1 | 1 | 1 |
| TWIK-related spinal cord K+ channel (TRESK) | 1 | 1 | 0,016375 | 1,029966 | 0,970906 | 1 | 1 | 1 |
| Tandem pore domain halothane-inhibited K+ channel (THIK) | 1 | 1 | 0,016375 | 1,029966 | 0,970906 | 1 | 1 | 1 |
| BoNT Light Chain Types B, D, and F cleave VAMP/Synaptobrevin | 1 | 1 | 0,016375 | 1,029966 | 0,970906 | 1 | 1 | 1 |
| BoNT Light Chain Types A, C1, E cleave SNAP-25 | 1 | 1 | 0,016375 | 1,029966 | 0,970906 | 1 | 1 | 1 |
| NGF processing | 1 | 1 | 0,016375 | 1,029966 | 0,970906 | 1 | 1 | 1 |
| Nuclear Receptor transcription pathway | 1 | 1 | 0,016375 | 1,029966 | 0,970906 | 1 | 1 | 1 |
| NADPH regeneration | 1 | 1 | 0,016375 | 1,029966 | 0,970906 | 1 | 1 | 1 |
| Translesion synthesis by HREV1 | 1 | 1 | 0,016375 | 1,029966 | 0,970906 | 1 | 1 | 1 |
| ABH2 mediated Reversal of Alkylation Damage | 1 | 1 | 0,016375 | 1,029966 | 0,970906 | 1 | 1 | 1 |
| ABH3 mediated Reversal of Alkylation Damage | 1 | 1 | 0,016375 | 1,029966 | 0,970906 | 1 | 1 | 1 |
| Translesion synthesis by Pol eta | 1 | 1 | 0,016375 | 1,029966 | 0,970906 | 1 | 1 | 1 |
| Synthesis of dolichyl-phosphate-glucose | 1 | 1 | 0,016375 | 1,029966 | 0,970906 | 1 | 1 | 1 |
| Reactions specific to the hybrid N-glycan synthesis pathway | 1 | 1 | 0,016375 | 1,029966 | 0,970906 | 1 | 1 | 1 |
| Inhibition of PKR | 1 | 1 | 0,016375 | 1,029966 | 0,970906 | 1 | 1 | 1 |
| Gap junction degradation | 1 | 1 | 0,016375 | 1,029966 | 0,970906 | 1 | 1 | 1 |
| Formation of annular gap junctions | 1 | 1 | 0,016375 | 1,029966 | 0,970906 | 1 | 1 | 1 |
| Microtubule-dependent trafficking of connexons from Golgi to the plasma membrane | 1 | 1 | 0,016375 | 1,029966 | 0,970906 | 1 | 1 | 1 |
| Transport of connexins along the secretory pathway | 1 | 1 | 0,016375 | 1,029966 | 0,970906 | 1 | 1 | 1 |
| Aromatic amines can be N-hydroxylated or N-dealkylated by CYP1A2 | 1 | 1 | 0,016375 | 1,029966 | 0,970906 | 1 | 1 | 1 |
| CYP2E1 reactions | 1 | 1 | 0,016375 | 1,029966 | 0,970906 | 1 | 1 | 1 |
| COX reactions | 1 | 1 | 0,016375 | 1,029966 | 0,970906 | 1 | 1 | 1 |
| Regulation of thyroid hormone activity | 1 | 1 | 0,016375 | 1,029966 | 0,970906 | 1 | 1 | 1 |
| Sterols are 12-hydroxylated by CYP8B1 | 1 | 1 | 0,016375 | 1,029966 | 0,970906 | 1 | 1 | 1 |
| Regulation of Insulin Secretion by Free Fatty Acids | 1 | 1 | 0,016375 | 1,029966 | 0,970906 | 1 | 1 | 1 |
| Regulation of Insulin Secretion by Fatty Acids Bound to GPR40 (FFAR1) | 1 | 1 | 0,016375 | 1,029966 | 0,970906 | 1 | 1 | 1 |
| Biotin metabolism | 1 | 1 | 0,016375 | 1,029966 | 0,970906 | 1 | 1 | 1 |
| 5-Phosphoribose 1-diphosphate biosynthesis | 1 | 1 | 0,016375 | 1,029966 | 0,970906 | 1 | 1 | 1 |
| Fructose catabolism | 1 | 1 | 0,016375 | 1,029966 | 0,970906 | 1 | 1 | 1 |
| Mitochondrial ABC transporters | 1 | 1 | 0,016375 | 1,029966 | 0,970906 | 1 | 1 | 1 |
| Passive Transport by Aquaporins | 1 | 1 | 0,016375 | 1,029966 | 0,970906 | 1 | 1 | 1 |
| Mitochondrial transcription termination | 1 | 1 | 0,016375 | 1,029966 | 0,970906 | 1 | 1 | 1 |
| PLC-mediated hydrolysis of PIP2 | 1 | 1 | 0,016375 | 1,029966 | 0,970906 | 1 | 1 | 1 |
| Cross-presentation of particulate exogenous antigens (phagosomes) | 1 | 1 | 0,016375 | 1,029966 | 0,970906 | 1 | 1 | 1 |
| The NLRP1 inflammasome | 1 | 1 | 0,016375 | 1,029966 | 0,970906 | 1 | 1 | 1 |
| 4-hydroxybenzoate biosynthesis | 1 | 1 | 0,016375 | 1,029966 | 0,970906 | 1 | 1 | 1 |
| diphthamide biosynthesis | 1 | 1 | 0,016375 | 1,029966 | 0,970906 | 1 | 1 | 1 |
| alanine biosynthesis III | 1 | 1 | 0,016375 | 1,029966 | 0,970906 | 1 | 1 | 1 |
| tyrosine biosynthesis IV | 1 | 1 | 0,016375 | 1,029966 | 0,970906 | 1 | 1 | 1 |
| asparagine biosynthesis I | 1 | 1 | 0,016375 | 1,029966 | 0,970906 | 1 | 1 | 1 |
| glutamine biosynthesis I | 1 | 1 | 0,016375 | 1,029966 | 0,970906 | 1 | 1 | 1 |
| molybdenum cofactor (sulfide) biosynthesis | 1 | 1 | 0,016375 | 1,029966 | 0,970906 | 1 | 1 | 1 |
| acyl carrier protein metabolism | 1 | 1 | 0,016375 | 1,029966 | 0,970906 | 1 | 1 | 1 |
| lipoate salvage and modification | 1 | 1 | 0,016375 | 1,029966 | 0,970906 | 1 | 1 | 1 |
| hexaprenyl diphosphate biosynthesis | 1 | 1 | 0,016375 | 1,029966 | 0,970906 | 1 | 1 | 1 |
| all-trans-decaprenyl diphosphate biosynthesis | 1 | 1 | 0,016375 | 1,029966 | 0,970906 | 1 | 1 | 1 |
| 1,25-dihydroxyvitamin D3 biosynthesis | 1 | 1 | 0,016375 | 1,029966 | 0,970906 | 1 | 1 | 1 |
| glutamate removal from folates | 1 | 1 | 0,016375 | 1,029966 | 0,970906 | 1 | 1 | 1 |
| thyroid hormone biosynthesis | 1 | 1 | 0,016375 | 1,029966 | 0,970906 | 1 | 1 | 1 |
| pregnenolone biosynthesis | 1 | 1 | 0,016375 | 1,029966 | 0,970906 | 1 | 1 | 1 |
| cardiolipin biosynthesis II | 1 | 1 | 0,016375 | 1,029966 | 0,970906 | 1 | 1 | 1 |
| phosphatidylethanolamine biosynthesis III | 1 | 1 | 0,016375 | 1,029966 | 0,970906 | 1 | 1 | 1 |
| lanosterol biosynthesis | 1 | 1 | 0,016375 | 1,029966 | 0,970906 | 1 | 1 | 1 |
| xanthine and xanthosine salvage | 1 | 1 | 0,016375 | 1,029966 | 0,970906 | 1 | 1 | 1 |
| adenine and adenosine salvage VI | 1 | 1 | 0,016375 | 1,029966 | 0,970906 | 1 | 1 | 1 |
| adenine and adenosine salvage I | 1 | 1 | 0,016375 | 1,029966 | 0,970906 | 1 | 1 | 1 |
| methylthiopropionate biosynthesis | 1 | 1 | 0,016375 | 1,029966 | 0,970906 | 1 | 1 | 1 |
| L-dopachrome biosynthesis | 1 | 1 | 0,016375 | 1,029966 | 0,970906 | 1 | 1 | 1 |
| histamine biosynthesis | 1 | 1 | 0,016375 | 1,029966 | 0,970906 | 1 | 1 | 1 |
| methylglyoxal degradation VI | 1 | 1 | 0,016375 | 1,029966 | 0,970906 | 1 | 1 | 1 |
| L-dopa degradation | 1 | 1 | 0,016375 | 1,029966 | 0,970906 | 1 | 1 | 1 |
| 4-hydroxyproline degradation I | 1 | 1 | 0,016375 | 1,029966 | 0,970906 | 1 | 1 | 1 |
| L-cysteine degradation I | 1 | 1 | 0,016375 | 1,029966 | 0,970906 | 1 | 1 | 1 |
| L-cysteine degradation II | 1 | 1 | 0,016375 | 1,029966 | 0,970906 | 1 | 1 | 1 |
| L-cysteine degradation III | 1 | 1 | 0,016375 | 1,029966 | 0,970906 | 1 | 1 | 1 |
| citrulline degradation | 1 | 1 | 0,016375 | 1,029966 | 0,970906 | 1 | 1 | 1 |
| threonine degradation II | 1 | 1 | 0,016375 | 1,029966 | 0,970906 | 1 | 1 | 1 |
| 2-amino-3-carboxymuconate semialdehyde degradation to glutaryl-CoA | 1 | 1 | 0,016375 | 1,029966 | 0,970906 | 1 | 1 | 1 |
| ascorbate recycling (cytosolic) | 1 | 1 | 0,016375 | 1,029966 | 0,970906 | 1 | 1 | 1 |
| D-mannose degradation | 1 | 1 | 0,016375 | 1,029966 | 0,970906 | 1 | 1 | 1 |
| fatty acid alpha-oxidation | 1 | 1 | 0,016375 | 1,029966 | 0,970906 | 1 | 1 | 1 |
| S-methyl-5'-thioadenosine degradation II | 1 | 1 | 0,016375 | 1,029966 | 0,970906 | 1 | 1 | 1 |
| sulfite oxidation IV | 1 | 1 | 0,016375 | 1,029966 | 0,970906 | 1 | 1 | 1 |
| thiosulfate disproportionation III (rhodanese) | 1 | 1 | 0,016375 | 1,029966 | 0,970906 | 1 | 1 | 1 |
| alpha-tocopherol degradation | 1 | 1 | 0,016375 | 1,029966 | 0,970906 | 1 | 1 | 1 |
| sorbitol degradation I | 1 | 1 | 0,016375 | 1,029966 | 0,970906 | 1 | 1 | 1 |
| acetyl-CoA biosynthesis (from citrate) | 1 | 1 | 0,016375 | 1,029966 | 0,970906 | 1 | 1 | 1 |
| Hexose transport | 37 | 40 | 0,605862 | 0,952737 | 0,971968 | 1 | 1 | 1 |
| Regulation of KIT signaling | 8 | 9 | 0,130997 | 0,915652 | 0,973509 | 1 | 1 | 1 |
| Phenylalanine and tyrosine catabolism | 8 | 9 | 0,130997 | 0,915652 | 0,973509 | 1 | 1 | 1 |
| Homologous Recombination Repair | 20 | 22 | 0,327493 | 0,936375 | 0,975162 | 1 | 1 | 1 |
| Homologous recombination repair of replication-independent double-strand breaks | 20 | 22 | 0,327493 | 0,936375 | 0,975162 | 1 | 1 | 1 |
| Glucose transport | 35 | 38 | 0,573113 | 0,948674 | 0,976326 | 1 | 1 | 1 |
| Activation of DNA fragmentation factor | 7 | 8 | 0,114623 | 0,901381 | 0,978998 | 1 | 1 | 1 |
| Apoptosis induced DNA fragmentation | 7 | 8 | 0,114623 | 0,901381 | 0,978998 | 1 | 1 | 1 |
| TRAF6 mediated IRF7 activation in TLR7/8 or 9 signaling | 7 | 8 | 0,114623 | 0,901381 | 0,978998 | 1 | 1 | 1 |
| 3-phosphoinositide degradation | 17 | 19 | 0,278369 | 0,921605 | 0,983346 | 1 | 1 | 1 |
| Tight junction interactions | 16 | 18 | 0,261994 | 0,915588 | 0,985674 | 1 | 1 | 1 |
| Fanconi anemia pathway | 44 | 48 | 0,720485 | 0,944153 | 0,987723 | 1 | 1 | 1 |
| Elongation arrest and recovery | 28 | 31 | 0,45849 | 0,930324 | 0,988196 | 1 | 1 | 1 |
| Pausing and recovery of HIV-1 elongation | 28 | 31 | 0,45849 | 0,930324 | 0,988196 | 1 | 1 | 1 |
| HIV-1 elongation arrest and recovery | 28 | 31 | 0,45849 | 0,930324 | 0,988196 | 1 | 1 | 1 |
| Pausing and recovery of elongation | 28 | 31 | 0,45849 | 0,930324 | 0,988196 | 1 | 1 | 1 |
| Elevation of cytosolic Ca2+ levels | 5 | 6 | 0,081873 | 0,85859 | 0,988309 | 1 | 1 | 1 |
| cGMP effects | 5 | 6 | 0,081873 | 0,85859 | 0,988309 | 1 | 1 | 1 |
| ATM mediated response to DNA double-strand break | 5 | 6 | 0,081873 | 0,85859 | 0,988309 | 1 | 1 | 1 |
| ATM mediated phosphorylation of repair proteins | 5 | 6 | 0,081873 | 0,85859 | 0,988309 | 1 | 1 | 1 |
| arginine degradation VI (arginase 2 pathway) | 5 | 6 | 0,081873 | 0,85859 | 0,988309 | 1 | 1 | 1 |
| phosphatidylethanolamine biosynthesis II | 5 | 6 | 0,081873 | 0,85859 | 0,988309 | 1 | 1 | 1 |
| Pausing and recovery of Tat-mediated HIV-1 elongation | 27 | 30 | 0,442116 | 0,927003 | 0,989485 | 1 | 1 | 1 |
| Tat-mediated HIV-1 elongation arrest and recovery | 27 | 30 | 0,442116 | 0,927003 | 0,989485 | 1 | 1 | 1 |
| phosphatidylglycerol biosynthesis II (non-plastidic) | 14 | 16 | 0,229245 | 0,9013 | 0,989741 | 1 | 1 | 1 |
| CDP-diacylglycerol biosynthesis I | 13 | 15 | 0,21287 | 0,892728 | 0,991485 | 1 | 1 | 1 |
| proline biosynthesis II (from arginine) | 4 | 5 | 0,065499 | 0,824384 | 0,992055 | 1 | 1 | 1 |
| glycine cleavage complex | 4 | 5 | 0,065499 | 0,824384 | 0,992055 | 1 | 1 | 1 |
| Aurora B signaling | 37 | 41 | 0,605862 | 0,929506 | 0,993676 | 1 | 1 | 1 |
| Platelet homeostasis | 36 | 40 | 0,589487 | 0,926995 | 0,994316 | 1 | 1 | 1 |
| NADE modulates death signalling | 3 | 4 | 0,049124 | 0,773116 | 0,99514 | 1 | 1 | 1 |
| Propionyl-CoA catabolism | 3 | 4 | 0,049124 | 0,773116 | 0,99514 | 1 | 1 | 1 |
| Digestion of dietary lipid | 3 | 4 | 0,049124 | 0,773116 | 0,99514 | 1 | 1 | 1 |
| proline biosynthesis I | 3 | 4 | 0,049124 | 0,773116 | 0,99514 | 1 | 1 | 1 |
| myo-inositol biosynthesis | 3 | 4 | 0,049124 | 0,773116 | 0,99514 | 1 | 1 | 1 |
| 2-oxobutanoate degradation I | 3 | 4 | 0,049124 | 0,773116 | 0,99514 | 1 | 1 | 1 |
| Immunoregulatory interactions between a Lymphoid and a non-Lymphoid cell | 47 | 52 | 0,769609 | 0,930949 | 0,996259 | 1 | 1 | 1 |
| Tryptophan catabolism | 9 | 11 | 0,147372 | 0,842869 | 0,996636 | 1 | 1 | 1 |
| purine nucleotides de novo biosynthesis II | 9 | 11 | 0,147372 | 0,842869 | 0,996636 | 1 | 1 | 1 |
| Mitochondrial tRNA aminoacylation | 18 | 21 | 0,294744 | 0,882898 | 0,997185 | 1 | 1 | 1 |
| methylmalonyl pathway | 2 | 3 | 0,032749 | 0,687784 | 0,997523 | 1 | 1 | 1 |
| Cell-Cell communication | 108 | 117 | 1,768462 | 0,950744 | 0,997955 | 1 | 1 | 1 |
| Sphingolipid metabolism | 25 | 29 | 0,409366 | 0,88795 | 0,998672 | 1 | 1 | 1 |
| Recruitment of repair and signaling proteins to double-strand breaks | 6 | 8 | 0,098248 | 0,772796 | 0,998782 | 1 | 1 | 1 |
| tryptophan degradation XI (mammalian, via kynurenine) | 6 | 8 | 0,098248 | 0,772796 | 0,998782 | 1 | 1 | 1 |
| Hemostasis | 354 | 375 | 5,796627 | 0,972289 | 0,998788 | 1 | 1 | 1 |
| GDP-L-fucose biosynthesis II (from L-fucose) | 1 | 2 | 0,016375 | 0,517545 | 0,999158 | 1 | 1 | 1 |
| GDP-L-fucose biosynthesis I (from GDP-D-mannose) | 1 | 2 | 0,016375 | 0,517545 | 0,999158 | 1 | 1 | 1 |
| Cell junction organization | 62 | 69 | 1,015228 | 0,925491 | 0,999192 | 1 | 1 | 1 |
| Nitric oxide stimulates guanylate cyclase | 9 | 12 | 0,147372 | 0,772689 | 0,999714 | 1 | 1 | 1 |
| Gene Expression | 355 | 378 | 5,813001 | 0,967297 | 0,999767 | 1 | 1 | 1 |
| Aurora C signaling | 3 | 5 | 0,049124 | 0,618802 | 0,999768 | 1 | 1 | 1 |
| Adherens junctions interactions | 22 | 27 | 0,360242 | 0,839302 | 0,999901 | 1 | 1 | 1 |
| MRN complex relocalizes to nuclear foci | 2 | 4 | 0,032749 | 0,516267 | 0,999905 | 1 | 1 | 1 |
| Assembly of the RAD50-MRE11-NBS1 complex at DNA double-strand breaks | 2 | 4 | 0,032749 | 0,516267 | 0,999905 | 1 | 1 | 1 |
| Cell-cell junction organization | 38 | 45 | 0,622237 | 0,869784 | 0,999962 | 1 | 1 | 1 |
| Respiratory electron transport | 65 | 76 | 1,064352 | 0,880911 | 0,999998 | 1 | 1 | 1 |
| Nectin/Necl trans heterodimerization | 3 | 7 | 0,049124 | 0,442253 | 1 | 1 | 1 | 1 |
| Factors involved in megakaryocyte development and platelet production | 103 | 119 | 1,686589 | 0,891494 | 1 | 1 | 1 | 1 |
| Signaling events mediated by HDAC Class I | 97 | 113 | 1,588341 | 0,884143 | 1 | 1 | 1 | 1 |
| triacylglycerol biosynthesis | 15 | 24 | 0,24562 | 0,643889 | 1 | 1 | 1 | 1 |
| Transcription | 146 | 178 | 2,390699 | 0,844814 | 1 | 1 | 1 | 1 |
| Nucleosome assembly | 20 | 35 | 0,327493 | 0,588678 | 1 | 1 | 1 | 1 |
| Deposition of New CENPA-containing Nucleosomes at the Centromere | 20 | 35 | 0,327493 | 0,588678 | 1 | 1 | 1 | 1 |
| RNA Polymerase I Promoter Opening | 3 | 31 | 0,049124 | 0,099974 | 1 | 1 | 1 | 1 |
| tRNA Aminoacylation | 24 | 42 | 0,392992 | 0,588657 | 1 | 1 | 1 | 1 |
| Meiotic Recombination | 25 | 42 | 0,409366 | 0,613174 | 1 | 1 | 1 | 1 |
| RNA Polymerase I Chain Elongation | 20 | 48 | 0,327493 | 0,429277 | 1 | 1 | 1 | 1 |
| Telomere Maintenance | 34 | 49 | 0,556738 | 0,714734 | 1 | 1 | 1 | 1 |
| Signaling events mediated by HDAC Class III | 24 | 39 | 0,392992 | 0,633927 | 1 | 1 | 1 | 1 |
| Meiotic Synapsis | 31 | 47 | 0,507614 | 0,679414 | 1 | 1 | 1 | 1 |
| RNA Polymerase I Promoter Clearance | 26 | 54 | 0,425741 | 0,496008 | 1 | 1 | 1 | 1 |
| RNA Polymerase I Transcription | 27 | 56 | 0,442116 | 0,496686 | 1 | 1 | 1 | 1 |
| Cytosolic tRNA aminoacylation | 6 | 24 | 0,098248 | 0,257813 | 1 | 1 | 1 | 1 |
| Chromosome Maintenance | 53 | 69 | 0,867857 | 0,791168 | 1 | 1 | 1 | 1 |
| Packaging Of Telomere Ends | 6 | 21 | 0,098248 | 0,294626 | 1 | 1 | 1 | 1 |
| RNA Polymerase I, RNA Polymerase III, and Mitochondrial Transcription | 63 | 92 | 1,031603 | 0,705338 | 1 | 1 | 1 | 1 |
| The citric acid (TCA) cycle and respiratory electron transport | 92 | 118 | 1,506468 | 0,803043 | 1 | 1 | 1 | 1 |
| Respiratory electron transport, ATP synthesis by chemiosmotic coupling, and heat production by uncoupling proteins. | 65 | 91 | 1,064352 | 0,735722 | 1 | 1 | 1 | 1 |
| Meiosis | 55 | 72 | 0,900606 | 0,786813 | 1 | 1 | 1 | 1 |


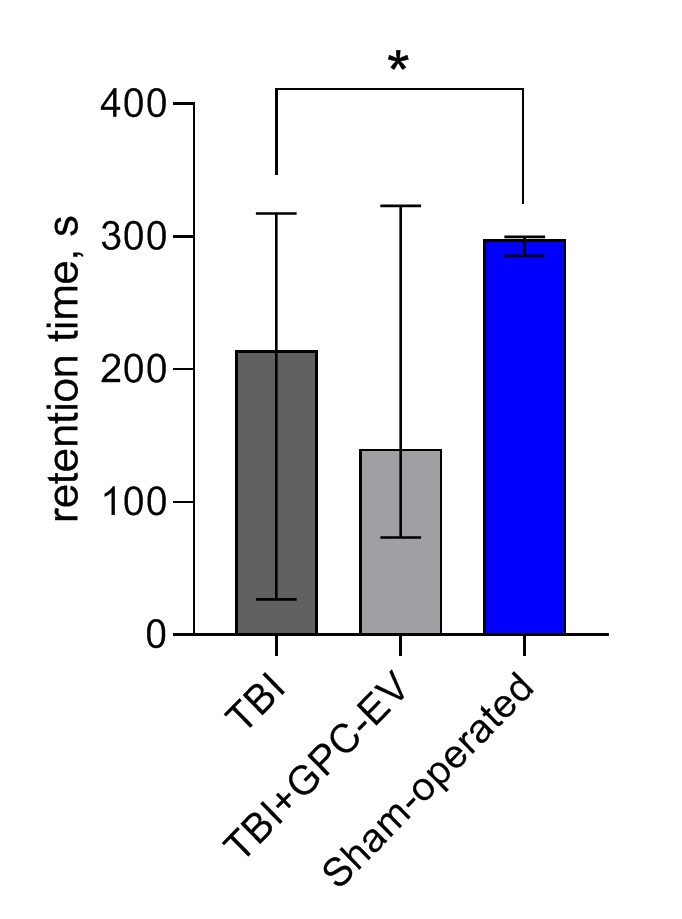


Figure S1. Neurological outcomes of animals during post-TBI recovery. Rotarod test. The data analyzed by Kruskal-Wallis test with post hoc Dunn's test are presented as median with range (* — p≤0.05 compared with the TBI group). TBI group — brain traumatized rats that received 30 µL PBS intranasally (n=10), TBI+GPC-EV group — rats with brain injury, and intranasal administration of GPC-EV (n=10). Sham-operated group — rats that underwent trepanation without TBI (n=6).

**
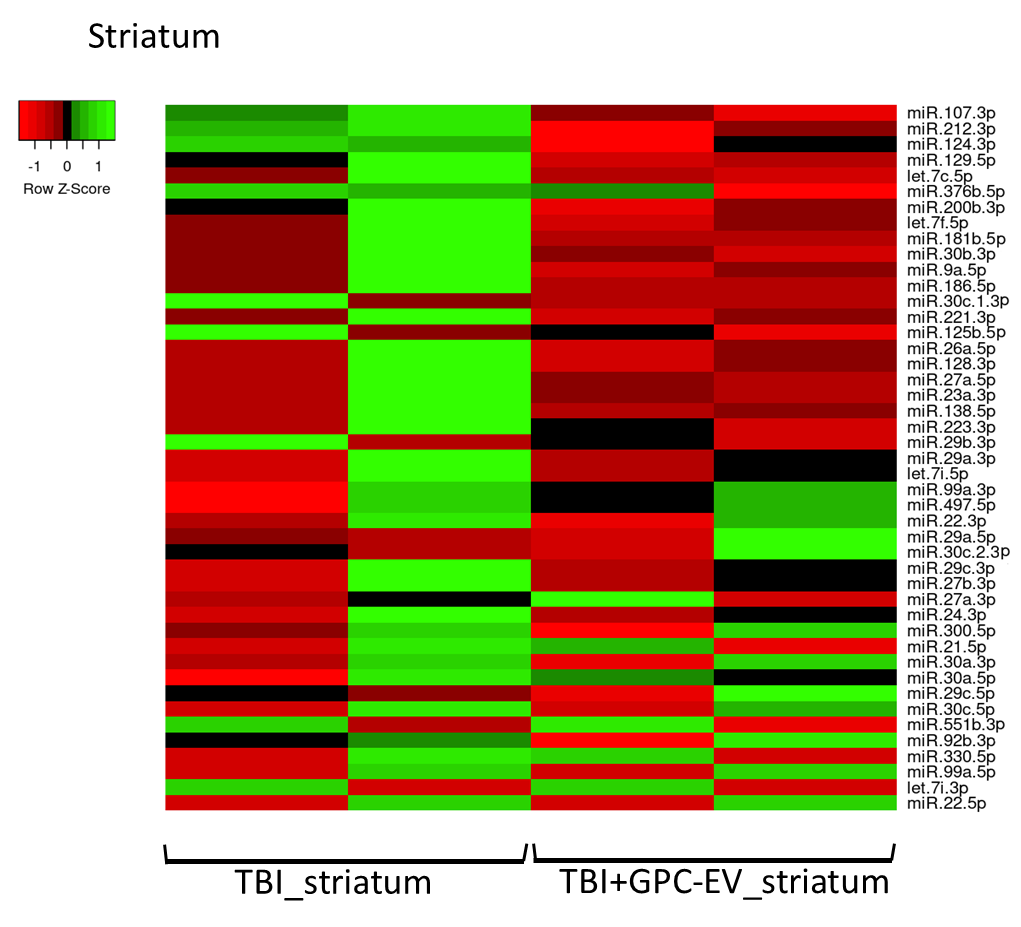
**

Figure S2. miRNA expression level in striatum. Real-time PCR data for miRNA on post-TBI day 7 analyzed by t-test are presented as heatmaps. TBI group — brain traumatized rats that received 30 µL PBS intranasally (n=2), TBI+GPC-EV group— rats with brain injury, and intranasal administration of GPC-EV (n=2).


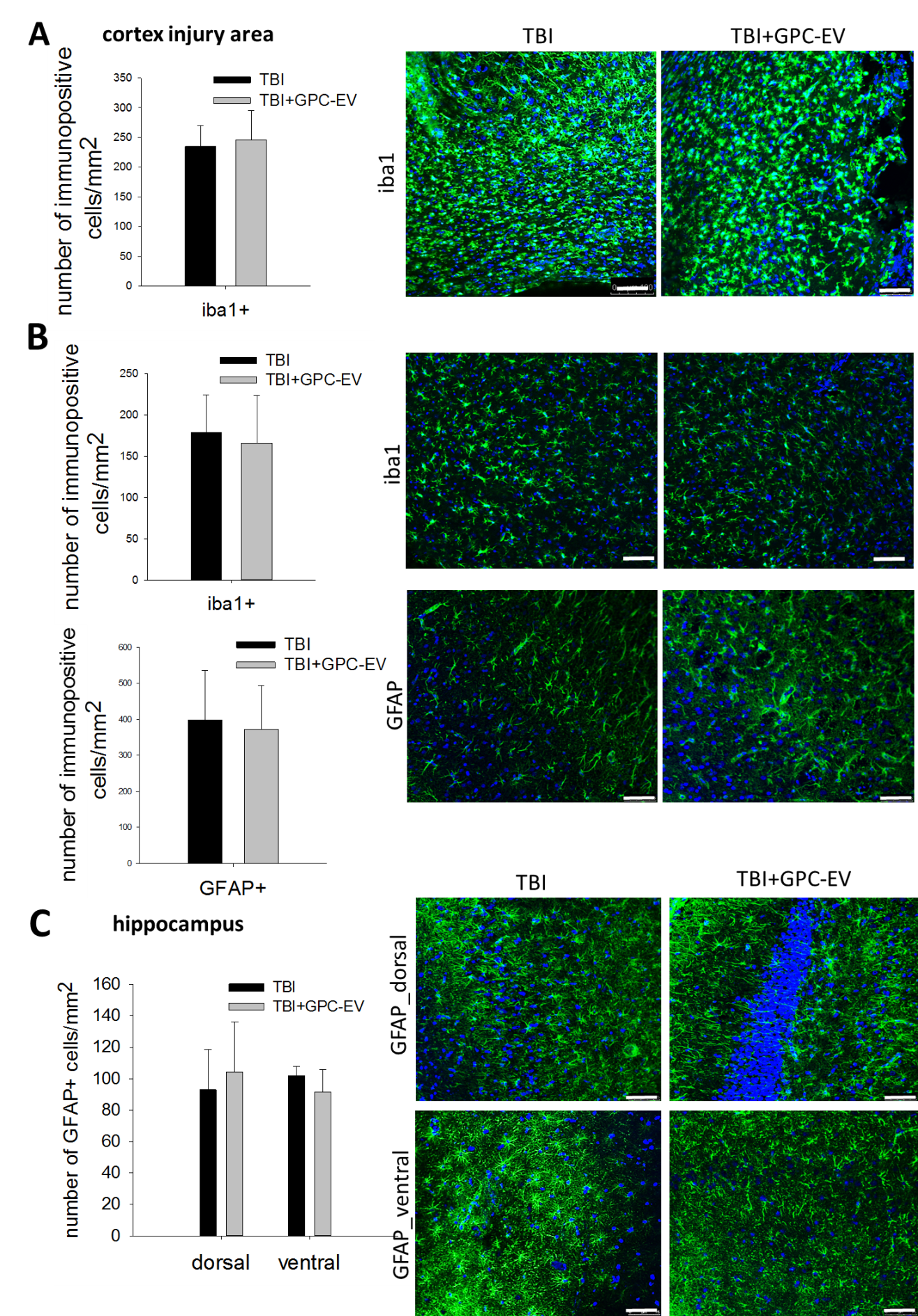


Figure S3. Immunohistochemical study of brain tissues on post-TBI day 14. (A) Visualization and counts of microglia (iba1+, green fluorescence) in cortex. (B) Visualization and counts of microglia (iba1^+^, green fluorescence) and astroglia (GFAP^+^, green fluorescence) in striatum. (C) Visualization and counts of astroglia cells (GFAP^+^, green fluorescence) in hippocampus. The data analyzed by *t*-test are presented as means ± standard deviations (* — p≤0.05 compared with the control). Scale bars, 75 µm. Cell nuclei counterstained with DAPI (blue fluorescence).

For Western Blot analysis were using following antibodies:

| Target | Reactivity | ID | Citations |
| --- | --- | --- | --- |
| Nf-kb | H M R Hm Mk Dg | 8242, cell signaling | https://www.cellsignal.com/products/primary-antibodies/nf-kb-p65-d14e12-xp-rabbit-mab/8242 |
| Tau | H M R | 46687, cell signaling | https://www.cellsignal.com/products/primary-antibodies/tau-d1m9x-xp-rabbit-mab/46687 |
| p-Tau (S396) | H M R | ab32057, abcam | https://www.abcam.com/products/primary-antibodies/tau-phospho-s396-antibody-e178-ab32057.html |
| p-Tau (T205) | H M R | ab254410, abcam | https://www.abcam.com/products/primary-antibodies/tau-phospho-t205-antibody-epr23505-13-ab254410.html |
| caspase-9 | M R | CSB-PA000010, cusabio | https://www.cusabio.com/Polyclonal-Antibody/Cleaved-CASP9-D353-Antibody-11090186.html |


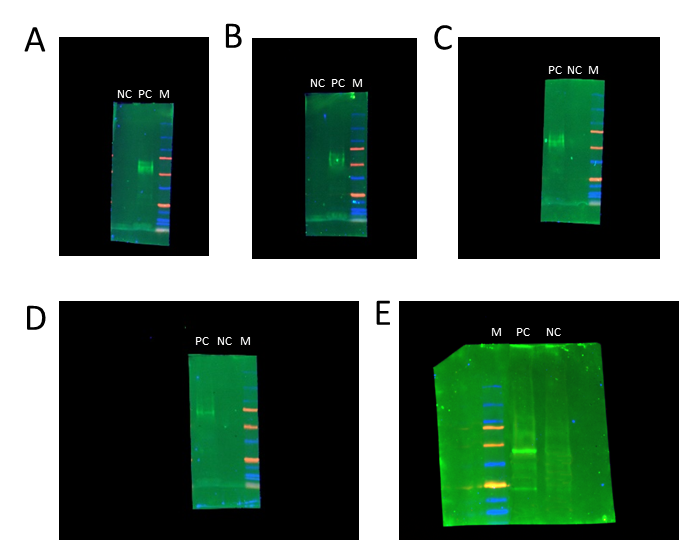


Figure S4. Representative blots reflecting positive and negative controls for Tau (A), p-Tau (Ser396) (B), p-Tau (Thr205) (C), Nf-kb (D), and caspase – 9 (E) using commercial kits: phospho-Tau (Ser356) control lysate (64TS356TDA, 20 µg/Lane), phospho-Tau (Ser202/Th205) control lysate (64TS2TDA, 20 µg/Lane), total Tau control lysate (64NTAUTDA, 20 µg/Lane), total NFkB control lysate (64NFTTDA, 20 µg/Lane) (all CISBIO BIOASSAYS, France) for positive controls; NFkB Knockout Cell Lysate from HeLa (# DAG-KO066, Creative Diagnostics, USA, 40 µg/Lane), lysates from rat liver tissue for negative controls for Tau and their phosphorylated forms, and mouse L929 Apoptosis Cell Lysates (staurosporine) (#9503, cell signaling, UK, 40 µg/Lane) for both controls, NC — negative control, PC — positive control, M — marker.


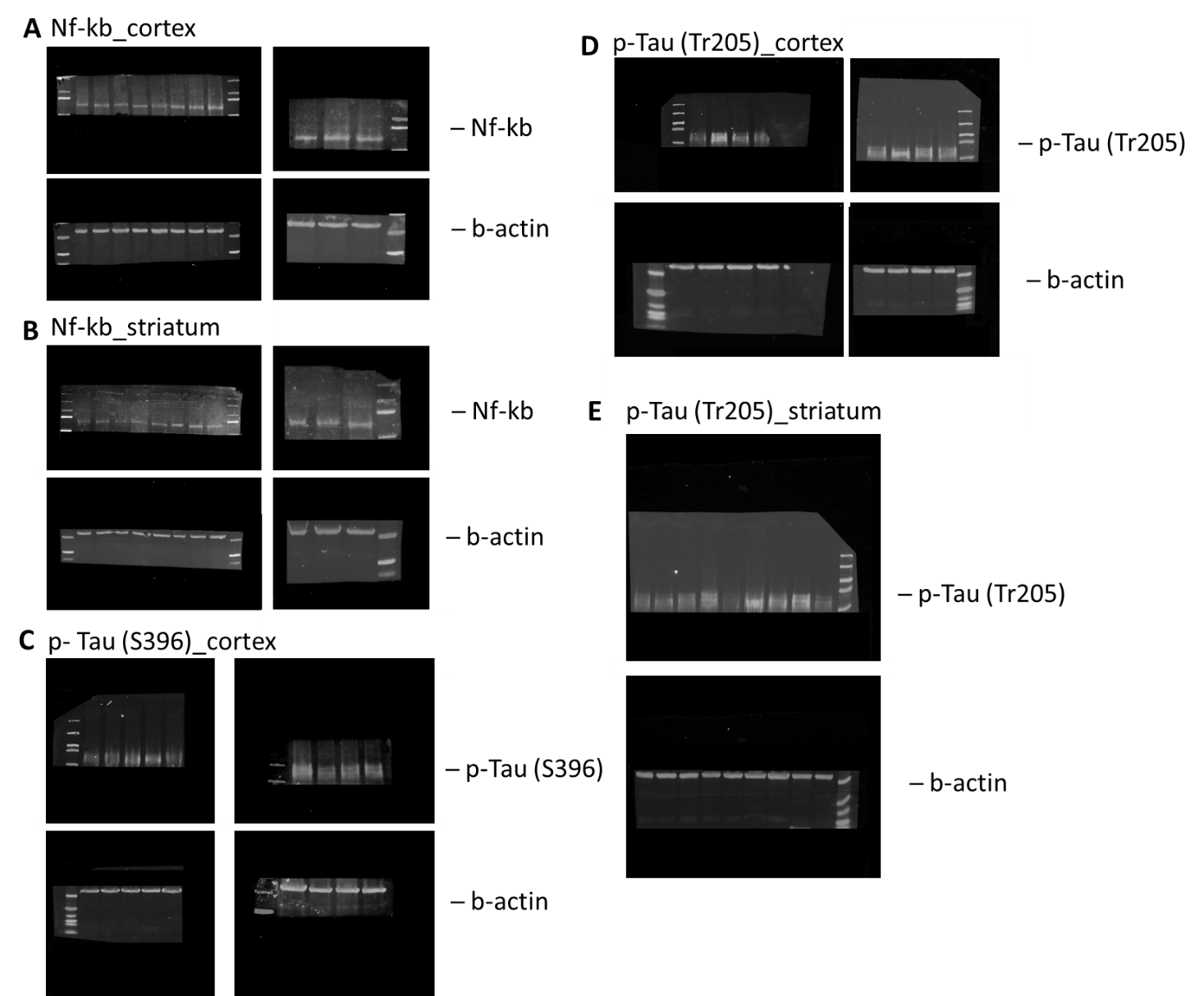


Figure S5. Representative blots reflecting grouping blots from two parts of the same gel for (A) Nf-kb in the cortex, (B) Nf-kb in the striatum, (C) p-Tau (S396) in the cortex, (D) p-Tau (Tr205) in the cortex, and (E) p-Tau (Tr205) in the striatum.
